# A Multi-Compartment Single and Multiple Dose Pharmacokinetic Comparison of Rectally Applied Tenofovir 1% Gel and Oral Tenofovir Disoproxil Fumarate

**DOI:** 10.1371/journal.pone.0106196

**Published:** 2014-10-28

**Authors:** Kuo-Hsiung Yang, Craig Hendrix, Namandje Bumpus, Julie Elliott, Karen Tanner, Christine Mauck, Ross Cranston, Ian McGowan, Nicola Richardson-Harman, Peter A. Anton, Angela D. M. Kashuba

**Affiliations:** 1 Division of Pharmacotherapy and Experimental Therapeutics, UNC Eshelman School of Pharmacy, Chapel Hill, North Carolina, United States of America; 2 Departments of Medicine and Pharmacology, Johns Hopkins University School of Medicine, Baltimore, Maryland, United States of America; 3 Center for HIV Prevention Research, David Geffen School of Medicine at UCLA, Los Angeles, California, United States of America; 4 Department of Biostatistics, UCLA School of Public Health, Los Angeles, California, United States of America; 5 CONRAD, Arlington, Virginia, United States of America; 6 Department of Medicine, University of Pittsburgh, Pittsburgh, Pennsylvania, United States of America; 7 Alpha StatConsult LLC, Damascus, Maryland, United States of America; Imperial College London, United Kingdom

## Abstract

This Phase 1, randomized, two-site (United States), double-blind, placebo-controlled study enrolled 18 sexually abstinent men and women. All received a single 300-mg dose of oral tenofovir disoproxil fumarate (TDF) and were then randomized 2∶1 to receive single and then seven daily rectal exposures of vaginally-formulated tenofovir (TFV) 1% gel or a hydroxyethyl cellulose (HEC) placebo gel. Blood, colonic biopsies and rectal and vaginal mucosal fluids were collected after the single oral TDF, the single topical TFV gel dose, and after 7 days of topical TFV gel dosing for extracellular analysis of TFV and intracellular analysis of the active metabolite tenofovir diphosphate (TFVdp) in peripheral blood mononuclear cells (PBMCs) and isolated mucosal mononuclear cells (MMC), including CD4+ and CD4- cell subsets. With a single rectal dose, TFV plasma concentrations were 24–33 fold lower and half-life was 5 h shorter compared to a single oral dose (p = 0.02). TFVdp concentrations were also undetectable in PBMCs with rectal dosing. Rectal tissue exposure to both TFV and TFVdp was 2 to 4-log_10_ higher after a single rectal dose compared to a single oral dose, and after 7 daily doses, TFVdp accumulated 4.5 fold in tissue. TFVdp in rectal tissue homogenate was predictive (residual standard error, RSE  = 0.47) of tissue MMC intracellular TFVdp concentration, with the CD4+ cells having a 2-fold higher TFVdp concentration than CD4- cells. TFV concentrations from rectal sponges was a modest surrogate indicator for both rectal tissue TFV and TFVdp (RSE  = 0.67, 0.66, respectively) and plasma TFV (RSE  = 0.38). TFV penetrates into the vaginal cavity after oral and rectal dosing, with rectal dosing leading to higher vaginal TFV concentrations (p<0.01).

**Trial Registration:**

ClinicalTrials.gov NCT00984971

## Introduction

Both topical and oral tenofovir (TFV)-containing regimens have demonstrated efficacy in HIV prevention. TFV 1% gel demonstrated 39% protective efficacy in women using the gel within 12 hours before and after sexual activity in the Centre for the AIDS Programme of Research in South Africa (CAPRISA) 004 study [1]. The fixed dose combination of tenofovir disoproxil fumarate (TDF)/emtricitabine (Truvada) prescribed daily in a population of men who have sex with men in the iPrEx study provided 44% protection against HIV infection [2]. Daily dosing of TDF or Truvada provided 62 to 73% protection against HIV transmission in serodiscordant men and women enrolled in the Partners PrEP Study [3]. Furthermore, daily dosing of oral TDF provided 49% reduction in HIV incidence rates among IV drug users [4]. In both CAPRISA 004 and iPrEx, the level of protection was related to drug exposure and adherence [1], [2], [5], [6].

The primary objective of the Phase 1 RMP-02/MTN-006 clinical trial was to evaluate the systemic safety of TFV 1% gel when applied rectally [7]. Built into this study was a comprehensive pharmacokinetic evaluation comparing systemic and compartmental pharmacokinetics among oral TDF and rectal TFV 1% gel users. These novel within-subject pharmacokinetic analyses were also used in an *ex-vivo* biopsy HIV challenge model to correlate TFVdp exposure with protection against *ex vivo* infection (*reported in accompanying paper: Richardson-Harman et al.*) [7]. This is the first study to quantify human rectal mucosal pharmacokinetics after topical administration of tenofovir in multiple compartments concurrently, to compare it to exposure after oral administration and to determine whether less-invasive indicators of TVFdp concentrations in tissue CD4+ cells emerge, potentially playing a future role in large clinical trials.

## Materials and Methods

The protocol for this trial and supporting CONSORT checklist are available as supporting information; see Checklist S1 and Protocol S1.

### Ethics Statement

The trial was IRB-approved at each site (UCLA IRB in Los Angeles, CA; University of Pittsburgh IRB, Committee C in Pittsburgh, PA); all participants provided written informed consent. RMP-02/MTN-006 is registered at ClinicalTrials.gov (#NCT00984971) and is in compliance with the CONSORT 2010 trial reporting recommendations (www.consortstatement.org).

### Study Participants

Study participants were healthy HIV-1-seronegative men and women with a history of consensual rectal anal intercourse, willing to abstain from vaginal and rectal sex during active protocol phases. Female participants were required to be using an acceptable form of contraception (e.g., barrier method, IUD, hormonal contraception, surgical sterilization, or vasectomization of male partner).

### Study Design

**Figure 1:**
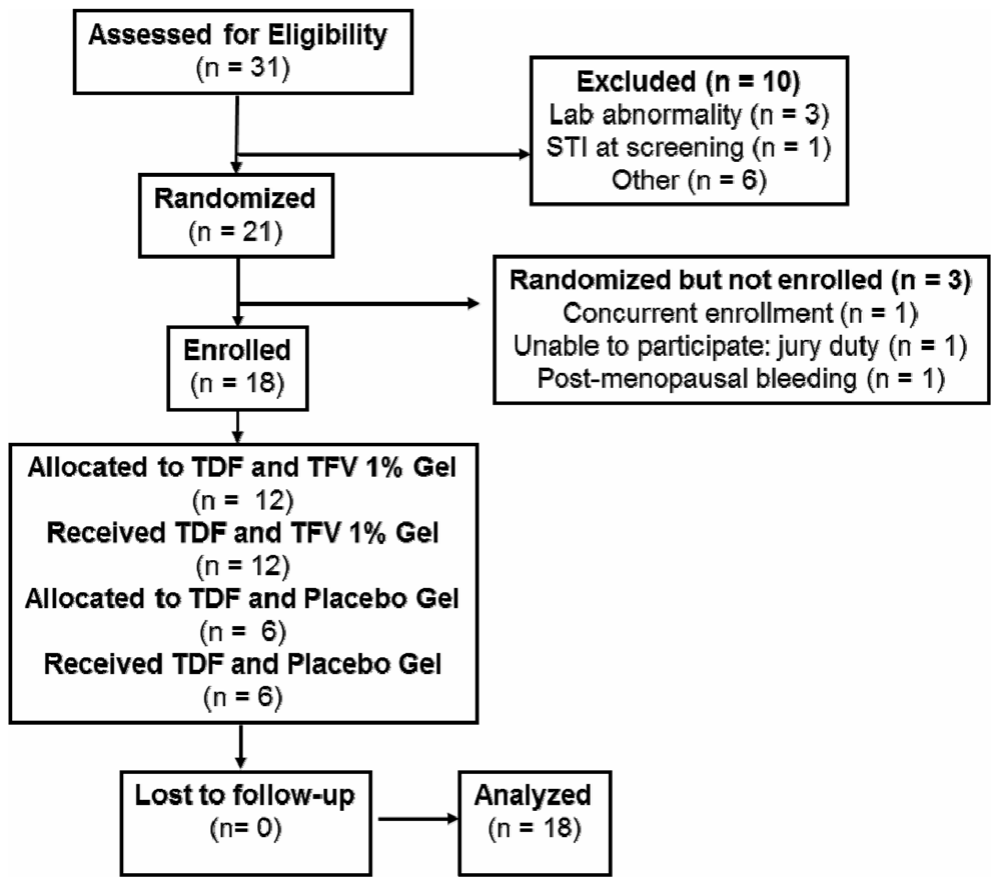
Study Design.

**Figure 2:**
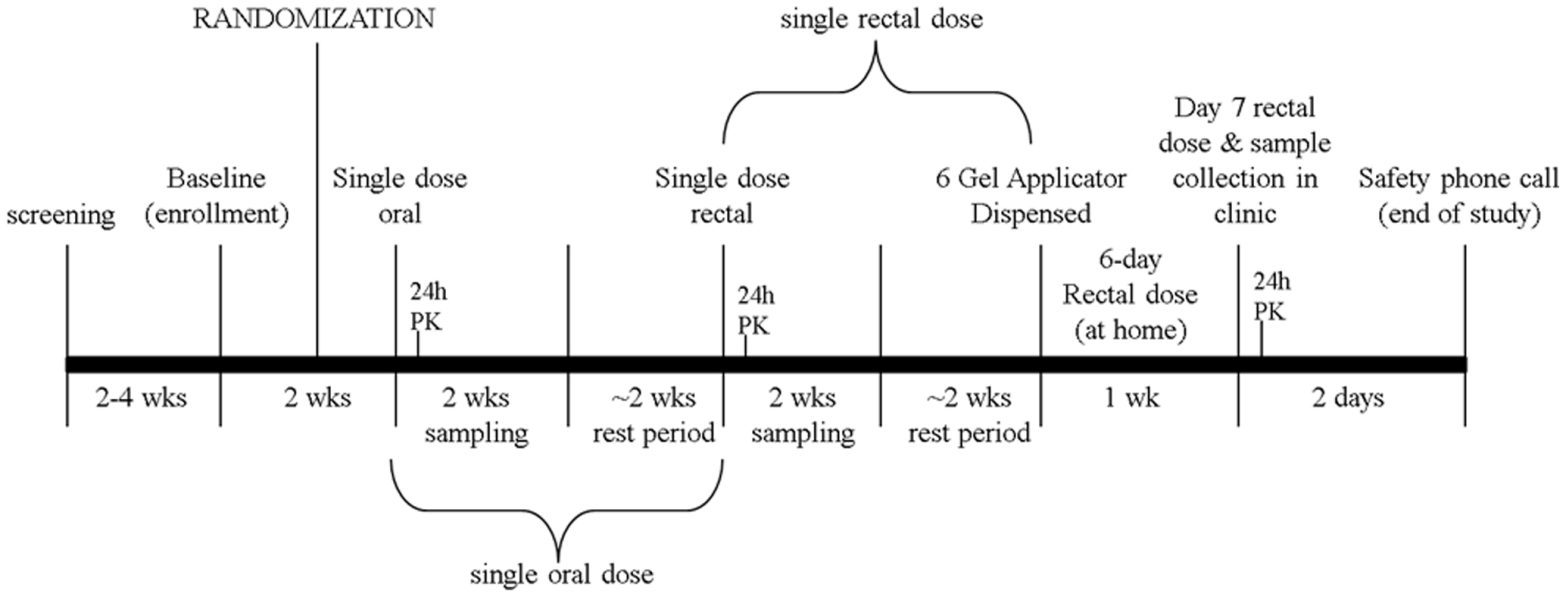
Sample Collection. All 18 trial participants received a single oral dose of 300 mg TDF followed by intensive 24 h PK. After ∼2 week resting period, 12 subjects were randomized to receive a single rectal gel dose of 1% TFV gel with intensive 24 h PK followed, after ∼2 week resting period, by 6 sequential, daily, self-administered rectal 1% TFV gel doses with the 7th dose administered in-clinic with subsequent 24 h intensive PK.

The pharmacokinetic component of the RMP-02/MTN-006 trial was designed to compare systemic and compartmental pharmacokinetics among single oral TDF dosing (300 mg), and single as well as multiple doses of rectally-applied vaginally-formulated TFV 1% gel. This was a Phase 1, double-blind, randomized, placebo-controlled comparison of oral TDF (300 mg), rectally applied TFV 1% gel, and the hydroxyethyl cellulose (HEC) placebo gel, the design of which has been previously published [7], and is illustrated in Figures 1, 2.

Briefly, in this three-stage trial, all participants received a single dose of oral TDF followed 4 weeks later by rectally applied TFV 1% gel or the HEC gel given as a single dose, and 4 weeks later, seven daily doses of the same product previously administered rectally. After enrollment, each subject was assigned to either the treatment or placebo arm (2∶1; TFV 1% gel:HEC gel) and also to one of two post-exposure biopsy sampling arms (groups “A” and “B”) to ensure mucosal safety. This limited the number of sigmoidoscopic procedures per participant to three sigmoidoscopic procedures in each of the first two study stages (single oral, single rectal) with several days delay between biopsy collections for mucosal healing. A single sigmoidoscopy biopsy collection point was used following the 3^rd^ stage (7-day rectal exposures). All subjects provided biopsies (and other compartment samples) 0.5 h after their dose of oral TDF or single/7-day dose of TFV gel. For the single oral and single rectal exposures, in addition to all subjects being sampled at 0.5 h, Group ‘‘A’’ subjects were also biopsied on days 1 (24 hr) and 7; Group ‘‘B’’ subjects were also biopsied on days 4 and 10. Participants were allowed a 2 day visit window for this sampling but nearly all were seen on the first day of their 3-day window. Just prior to each biopsy sample, blood samples were obtained for plasma and peripheral blood mononuclear cell isolation, and rectal and vaginal mucosal fluids were collected by sponge. Each 2 weeks of biopsy sampling was followed by a 2-week resting period. The sample size (N = 18) was based on a similar phase 1 study of topical microbicide UC781. [8] The study was conducted from November 2009 to July 2010.

#### Pharmacokinetic Procedures for Single Oral and Topical Dosing

All participants had blood plasma, PBMCs, and vaginal and rectal fluid obtained before single oral and rectal dosing. Additionally, 30 minutes after the dose, blood plasma, PBMCs, rectal biopsies, rectal fluid and vaginal fluid samples were obtained. At 2, 4, and 24 h after the dose, blood plasma, PBMCs, and rectal/vaginal fluid samples were obtained. Subsequently, on either days 1 and 7 (Group A) or 4 and 10 (Group B) post-dose, blood plasma, PBMCs, rectal biopsies, rectal/vaginal fluid were obtained.

#### Pharmacokinetic Procedures for Multiple Topical Dosing

Thirty minutes after the 7^th^ rectal dose of gel (observed), blood plasma, PBMCs, rectal biopsies, rectal fluid and vaginal fluid samples were obtained. At 2, 4, and 24 h after the dose, blood plasma, PBMCs, rectal fluid, and vaginal fluid samples were obtained.

### Study Products

TDF tablets (300 mg) were supplied by Gilead Sciences (Foster City, CA). TFV 1% gel and HEC gel were supplied by CONRAD (Arlington, VA). TFV 1% gel (weight/weight) is tenofovir (PMPA, 9-[(R)-2-(phosphonomethoxy)propyl]adenine monohydrate), formulated in purified water with edetate disodium, citric acid, glycerin, methylparaben, propylparaben, and hydroxyethyl cellulose with pH adjusted to 4–5 with an osmolarity of 3111 mOsmol/kg. The HEC placebo gel contained hydroxyethyl cellulose as the gel thickener, purified water, sodium chloride, sorbic acid, and sodium hydroxide. The gel was isotonic with a pH of 4.4, osmolarity of 304 mOsmol/kg, 24 and viscosity similar to the other microbicide gel candidates. Both TFV and HEC gels were prefilled into single-use, opaque applicators (HTI Plastics, Lincoln, NE) containing 4 ml of gel.

### Sample Processing

Plasma was collected in tubes containing EDTA anticoagulant. Samples were centrifuged at 800 g for 10 minutes at 4°C, plasma aliquoted into cryovials, and stored at −70°C. Peripheral blood mononuclear cells (PBMCs) were isolated via centrifugation from cell preparation tubes (CPT) at 1,800×g for 25 min at 28°C. PBMCs were collected from the buffy coat and washed twice with normal saline at room temperature (∼21°C). Cells were resuspended in 1 ml normal saline for cell counting. Cell pellets were lysed with 70% methanol and stored at −80°C until analysis.

To release cells for intracellular analysis from colonic tissue, biopsies were incubated with a dissociative enzyme cocktail consisting of collagenase (0.5 mg/ml, Sigma-Aldrich, St. Louis, MO), DNase I (0.083 U/ml, Roche, Indianapolis, IN), elastase (0.07 U/ml, Worthington Biochemicals, Lakewood, NJ), and hyaluronidase (0.4 U/ml, Worthington Biochemicals, Lakewood, NJ). The digestions were carried out in RPMI with 7.5% FBS in 50 ml-conical tubes at 37°C with agitation (Invitrogen, Carlsbad, CA) as previously described. [9], [10] Cells were counted using Guava/Millipore EasyCyte Plus (Millipore, Billerica, MA).

CD4+ T cells from tissue and PBMCs were isolated via positive selection with CD4 microbeads using magnetic affinity column separation (MACS) according to the manufacturer's recommended protocol (Miltenyi Biotec, Auburn, CA). CD4-positive and CD4-negative fractions were collected for cell counting and intracellular drug analysis. We used PBMCs to test for changes in intracellular TFV-DP concentrations as a result of tissue cell extraction and cell subset isolation compared to typical PBMC preparation. Compared to usual PBMC processing, there was no difference in PBMC TFV-DP concentrations resulting from the tissue digestion cocktail, monoclonal antibody incubation, or running cells over the MACS column.

### Sample Analysis

All TFV and TFVdp concentrations were measured using validated LC–MS/MS methods. Total numbers of samples analyzed were as follows: 275 blood plasma, 460 PBMCs, 205 tissue homogenates, 98 isolated mononuclear cells, 99 CD4+ cells, 99 CD4- cells, 264 rectal sponges, 54 vaginal sponges [11]. Briefly, TFV and TFVdp concentrations were determined by previously described LC-MS/MS methods [12], [13] validated for all matrices by the Johns Hopkins Clinical Pharmacology Analytical Laboratory. TFV and TFVdp assays meet the FDA bioanalysis guidance values of ≤ ±15% for precision and accuracy [14]. All calibrators were prepared using analyte calibrator stock solution diluted in the relevant human biological matrix corresponding to the samples to be assayed (plasma, PBMC lysate, cervicovaginal fluid, rectal fluid, homogenized colon or vaginal tissue). Thawed aliquots of plasma and tissue homogenate with ^13^C-TFV internal standard were protein precipitated with methanol. Vaginal and rectal fluid sponges were eluted in 50∶50 methanol:water mixture. Sponges were weighed both before and after. Aliquots, also with ^13^C5-TFV internal standard, underwent solid phase extraction using HLB oasis cartridges. The supernatants and eluants were collected, dried, and reconstituted in 0.5% acetic acid for analysis. For chromatographic separation of samples, a gradient elution with a Zorbax Eclipse XDB-C18 column, with positive electrospray ionization (ESI) was used, with detection via multiple reaction monitoring using a LC-MS/MS system (Waters Acquity UPLC and Agilent 1100 HPLC Applied Biosystems API4000 mass spectrometer). Calibration standards for the TFV assay ranged from 0.31 to 1,280 ng/ml (0.25–50 ng/sample for tissue).

For intracellular TFVdp analysis, tissue homogenates and isolated cell lysates were analyzed using an indirect assay [13]. TFVdp was isolated from cell lysates on a Waters QMA cartridge (Waters Corporation, Milford, MA) over a salt (KCl) gradient. TFV and tenofovir monophosphate (TFVmp) were separated from the cartridge under lower salt concentrations followed by elution of TFVdp with application of 1 M KCl to the cartridge. Isolated TFVdp was then enzymatically dephosphorylated to TFV via phosphatase digestion with incubation with phosphatase and ^13^C-TFV internal standard. TFV was isolated from the KCl solution using trifluoroacetic acid and eluted in methanol. TFV with ^13^C-TFV internal standard was analyzed via UPLC-MS/MS mass spectrometer as described above.

## Pharmacokinetic and Statistical Analysis

### Data Standardization

All values below the limit of quantification (BLQ) were imputed to be 0.01 for noncompartmental pharmacokinetic analysis (NCA) for all arms except rectal administration of placebo. Concentration data during rectal administration for the placebo arm is excluded from this report. All sponge concentrations (ng/sponge) were normalized to fluid weight on sponge (g) by division, resulting in units of ng/g. Data are presented as median (range) unless otherwise noted.

### PK Parameter Calculations

Actual time after dosing was used for NCA. When calculating individual pharmacokinetic (PK) parameters (C_max_, T_max_, AUC_24 h_, half-life), only profiles with: a) greater than two data points, b) last concentration time point less than or equal to 24 h, and c) first concentration time point less than or equal to 12 h were used. A composite PK profile was constructed for rectal tissue homogenates using the median concentrations from all subjects at each nominal time point, regardless of the actual time. In this case, PK parameters were calculated based on this composite profile with nominal times. Composite profiles must be calculated with protocol-specified nominal times because descriptive statistics cannot be calculated with actual times; there were minor deviations in sample collection time during the trial, as it is impossible to collect at the exact protocol-specified time. For calculating AUC_24 h_, linear interpolation was used to interpolate the concentration at 24 h if the actual time was greater than 24 h. If actual time was between 22 h and 24 h, then that concentration was imputed to be the 24 h concentration. Half-life estimation was performed by choosing points during the beta-phase of the elimination slope. (3 single rectal dose plasma PK profiles contained an imputed value of 0.01 at the 24 h timepoint.)

Parameter calculation was performed with Phoenix WinNonlin 6.3.0.395 (Certara/Pharsight). Data manipulation and plotting was performed with R 2.15.10 [15] with libraries: lattice [16], latticeExtra [17], plyr [18] and reshape2 [19].

Accumulation ratio for rectal dosing was defined in one of two ways: either the AUC_24 h_ ratio of multiple dose gel to single dose gel, or if unavailable, the 0.5 h concentration ratio of multiple dose gel to single dose gel.

### Statistical Analysis

Robust linear regression was carried out with robust package for R [20], and fitted to the model: DV = β_0_+β_1_×IV+β_2_×CV+β_3_×IV×CV. Dependent (DV) and independent variables (IV) are continuous, while categorical variable (CV) is either 0 or 1. Any parameter terms with p>0.05 were dropped and excluded from the statistical model, and re-fitted with a simpler model (backward elimination). All robust linear regression was performed with log_10_-transformed concentration data. Robust linear regression allows for differential weights to datapoints in respect to outlier datapoint; outliers were given less weight. This results in a more stable model. [21] All statistical comparisons were performed using paired Wilcoxon signed-rank test with a bonferroni correction for 2 comparisons (oral to single rectal, single to multiple rectal), resulting in a critical p-value of 0.025 (0.05/2). Standard error (SE, standard deviation) was a measure of uncertainty around parameter estimates. Relative standard error (RSE) was a measure of overall goodness of fit (0 would be a perfect fit).

## Results

### Demographics

**Figure 3:**
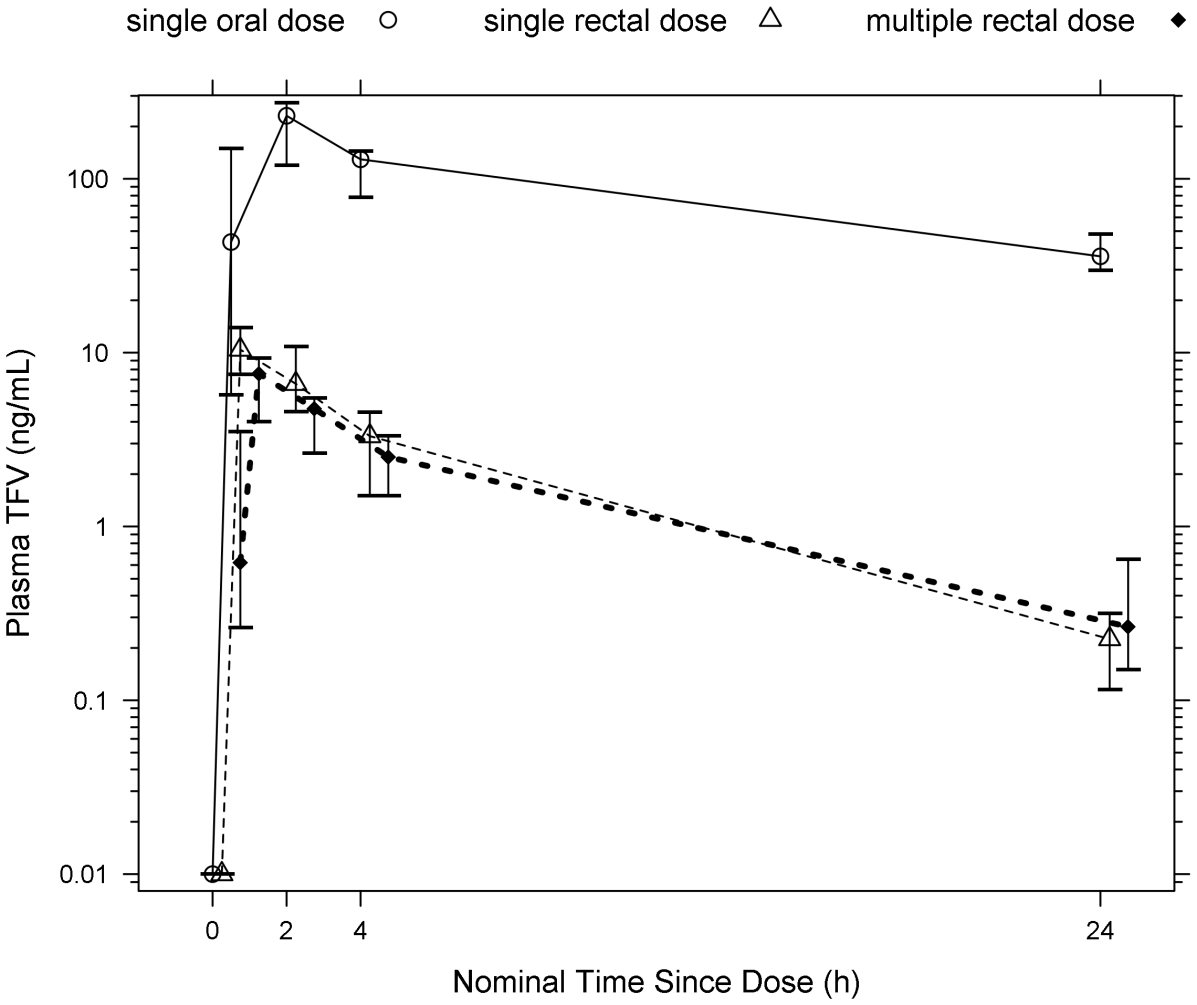
TFV Plasma Half-life is shorter (p = 0.02) during rectal administration. Plasma concentration-time profile is shown (median and interquartile range). Nominal time for single rectal dose was shifted right by 0.250 h and multiple rectal dose by 0.500 h for clarity. N = 18 for oral dose, 12 single rectal dose, 12 multiple rectal dose. (BLQ values are imputed as 0.01.)

**Figure 4:**
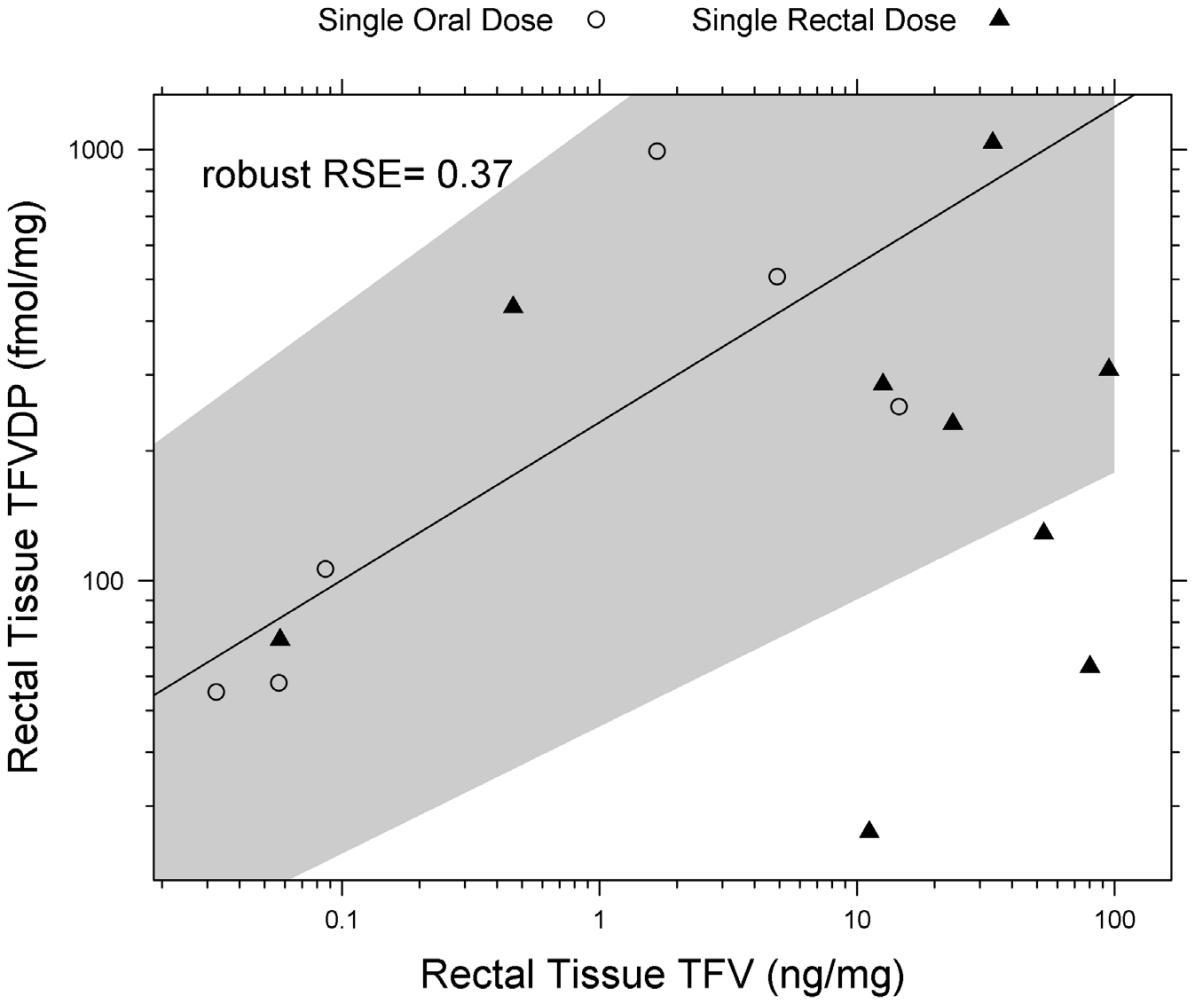
Increasing TFV in Rectal Tissue Homogenate results in increased TFVdp. There is a linear correlation between rectal tissue homogenate TFVdp and TFV (p = 0.04, robust RSE  = 0.37) with oral dosing only (solid line is mean prediction of TFVdp in rectal tissue from oral dosing). Shaded regions are the 10–90% confidence intervals of the mean predictions from robust linear regression model. There is no correlation during rectal dosing.

**Figure 5:**
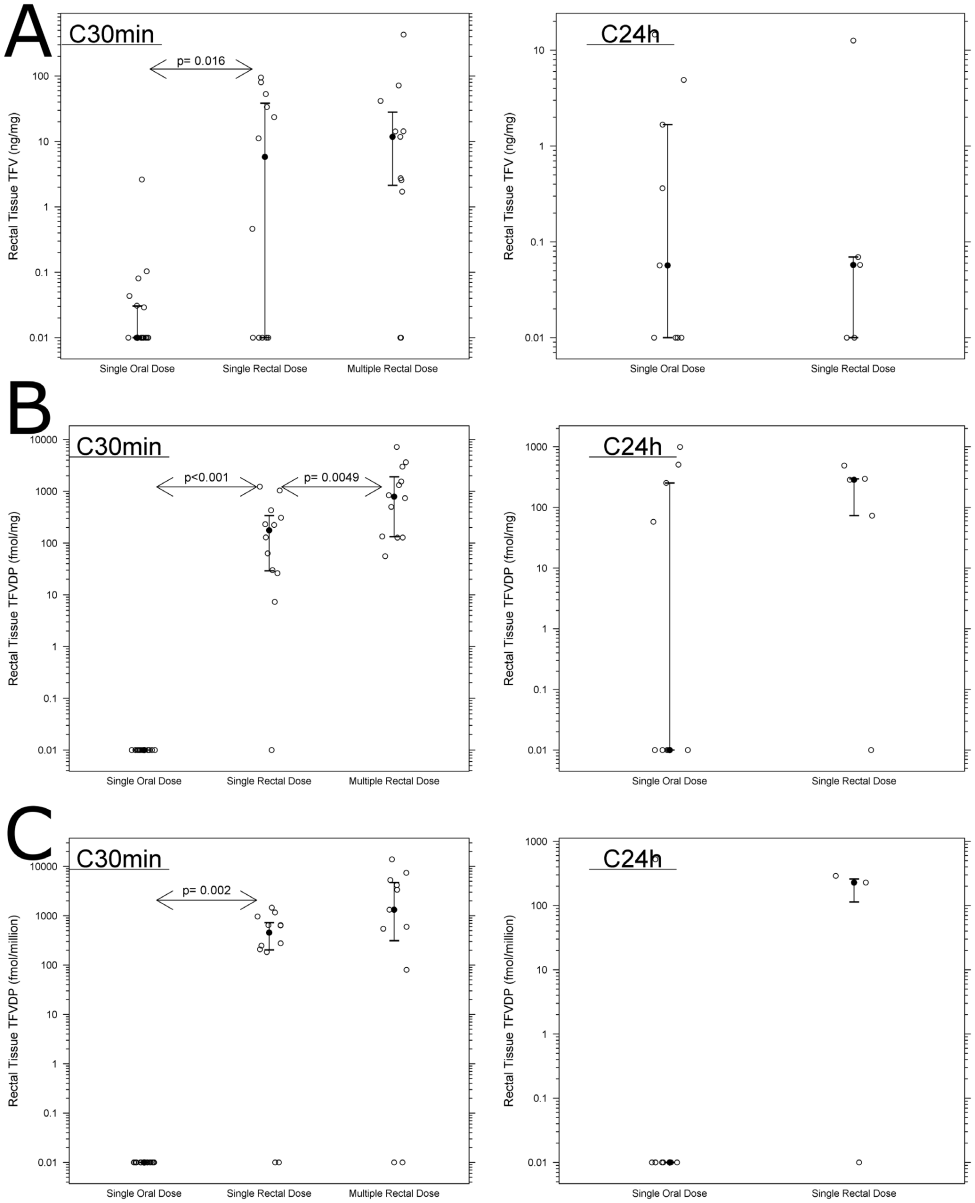
Rectal tissue exposure to TFV and TFVdp (median ± IQR) is higher during rectal dosing with multiple rectal dosing, resulting in accumulation of TFVdp. Each set of figures documents the 30 min drug quantification in the left-side graph and the 24 hr in the right side graph in rectal tissue biopsy homogenate (5A, 5B) and isolated mucosal mononuclear immune cells (MMC) (5C). Comparisons performed with paired Wilcoxon signed-rank test; only a subset of patients gave both C_30 min_ and C_24 h_ samples. Figure S5A  =  TFV_Tissue_; Figure S5B  =  TFVdp_Tissue_; Figure S5C  =  TFVdp_MMC_. There is accumulation of TFV and TFVdp from multiple rectal dosing. Critical p for significance was 0.025 after Bonferroni correction.

**Figure 6:**
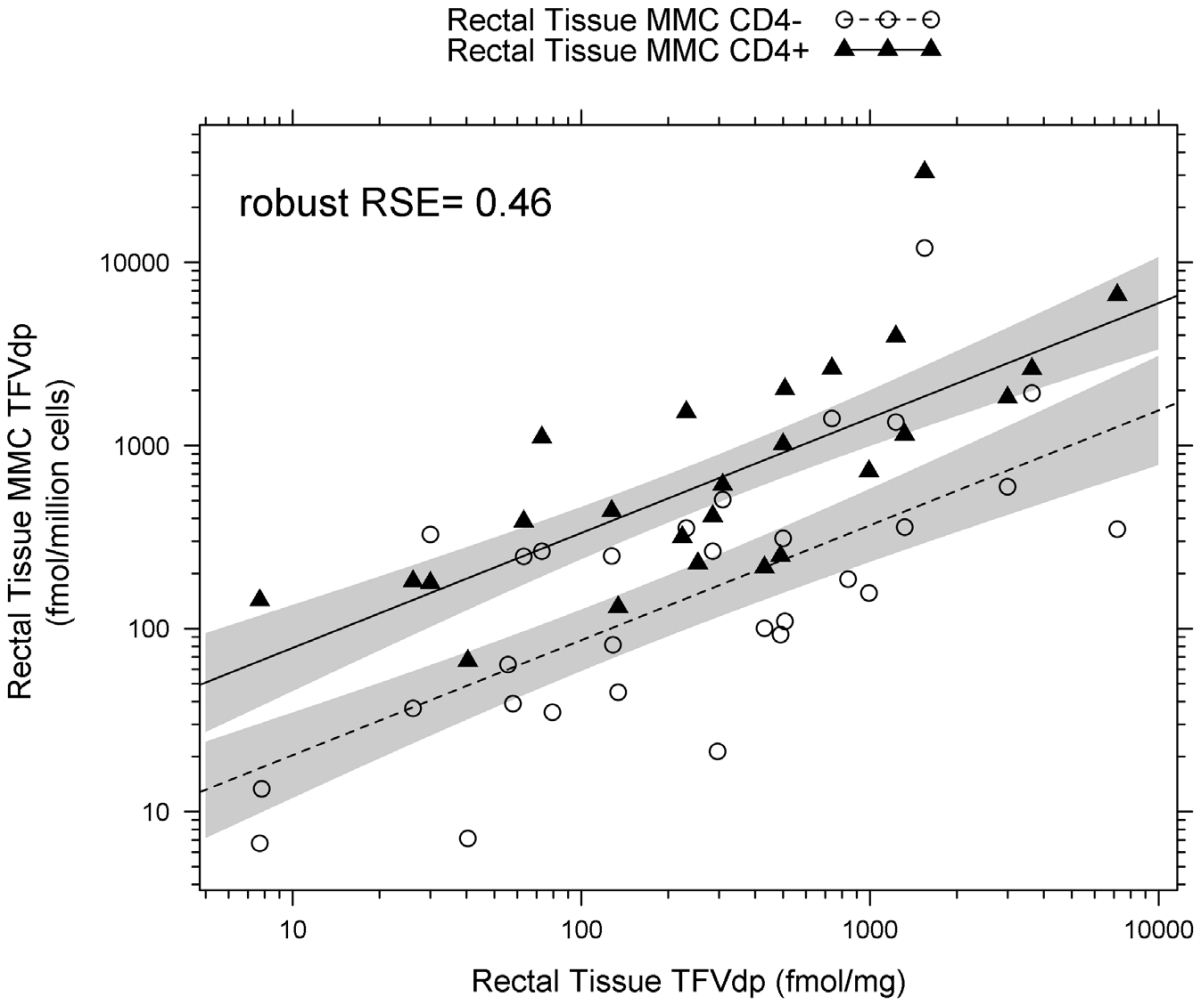
TFVdp in rectal tissue homogenate is predictive of intracellular TFVdp concentration in isolated rectal mucosal mononuclear cells (MMCs), with higher levels of phosphorylation in the CD4+ T cells compared to CD4- T cells. Intracellular TFVdp concentration in isolated rectal mucosal mononuclear cells increases linearly as TFVdp concentration in rectal tissue homogenate increases. (p<0.001, robust RSE  = 0.46) There is higher phosphorylation of TFV in CD4+ cells, seen in its higher y-intercept. The lines are the mean rectal tissue MMC TFVdp concentration predictions from robust linear regression model; solid is CD4+, dashed is CD4-. Shaded regions are the 10–90% confidence intervals of the mean prediction.

**Figure 7:**
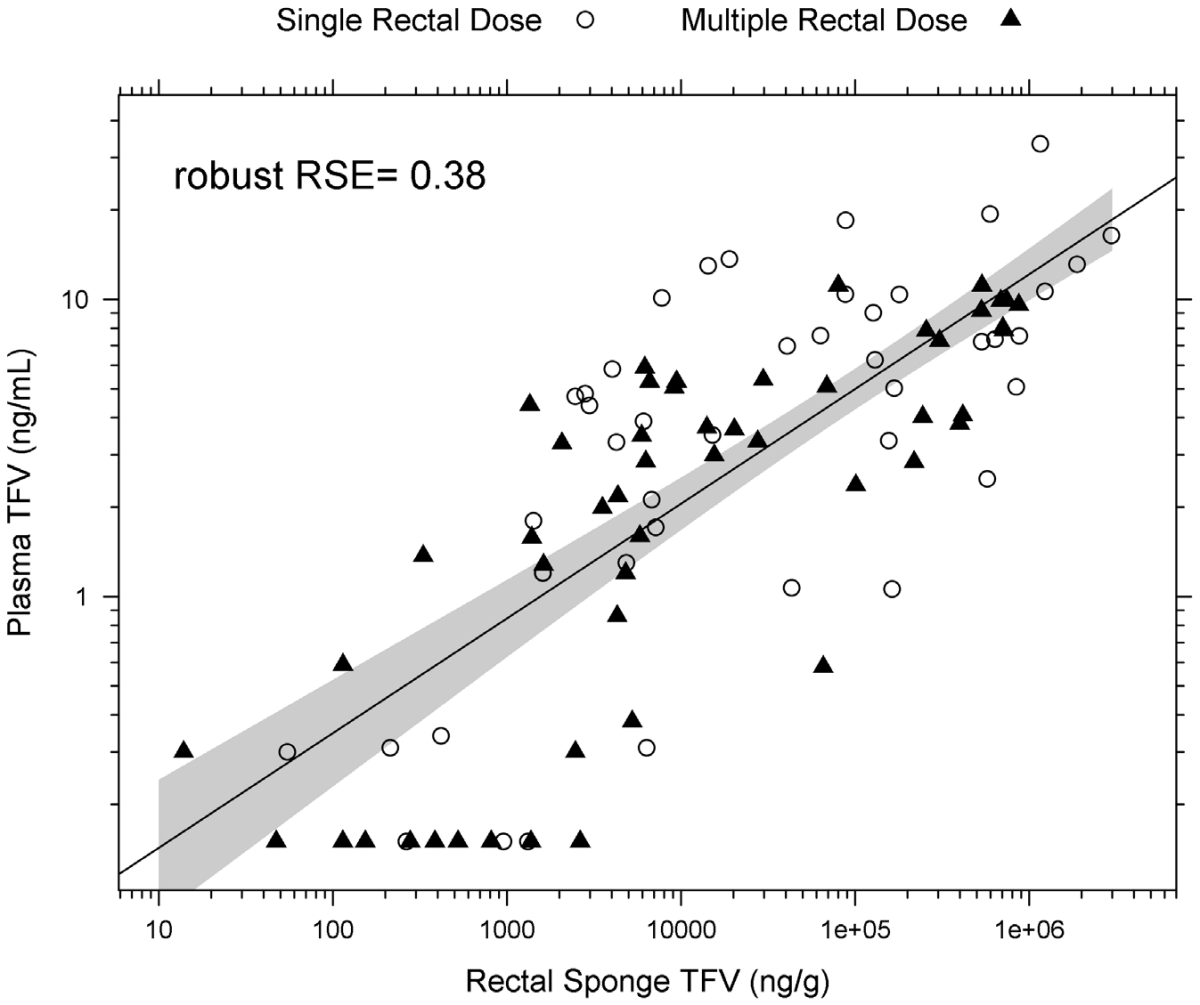
TFV quantification from rectal sponges is predictive of plasma TFV exposure during rectal dosing. Plasma TFV exposure is correlated linearly with rectal sponge TFV exposure. (p<0.001, robust RSE  = 0.38) The linear correlation is the same regardless of number of rectal doses. Shaded regions are the 10–90% confidence intervals of the mean predictions from robust linear regression model.

**Figure 8:**
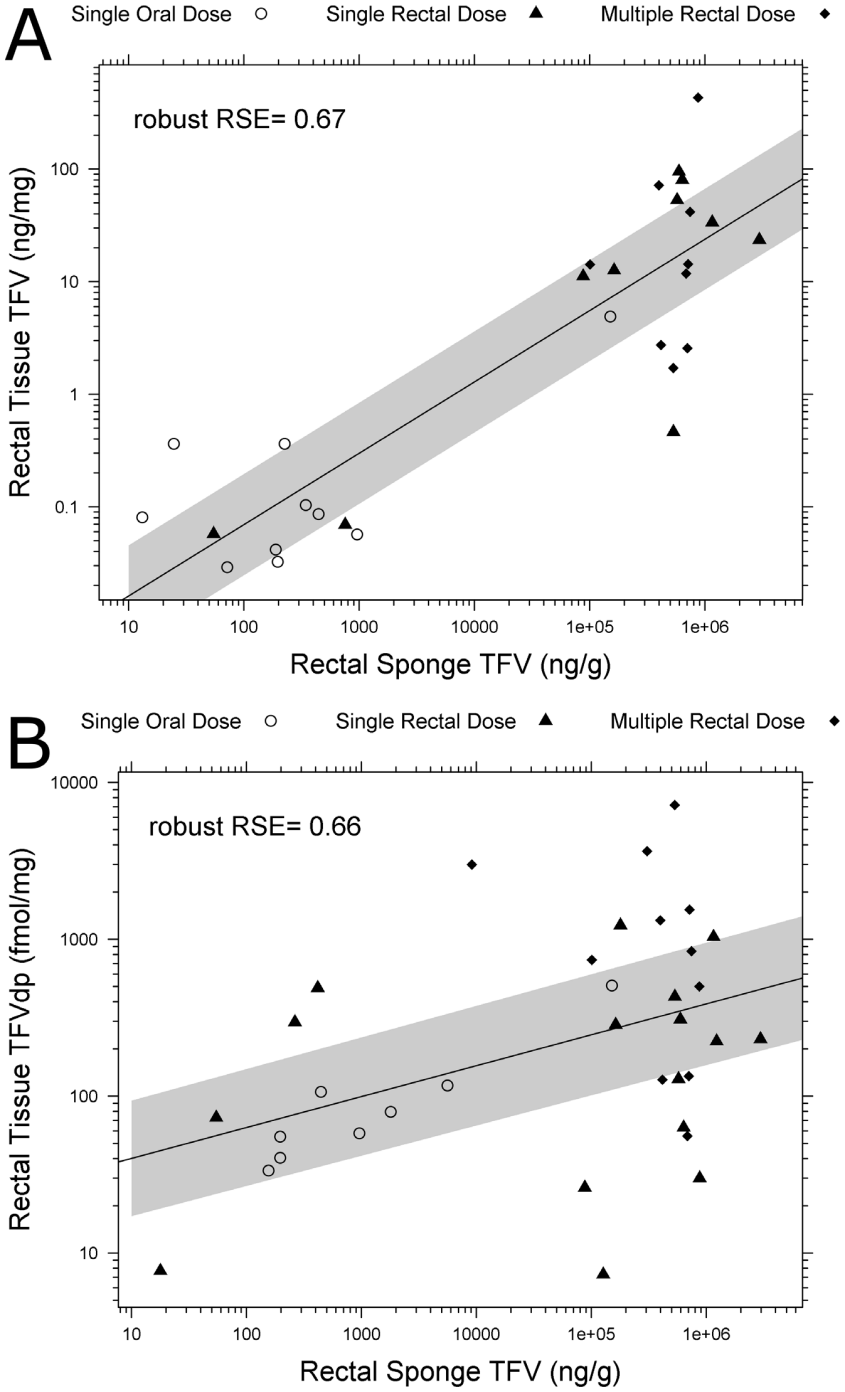
TFV (A) and TFVdp (B) concentrations in rectal tissue homogenate are predicted by Rectal Sponge TFV. (p<0.001, robust RSE_TFV_  = 0.67, RSE_TFVdp_  = 0.66) Shaded regions are the 10–90% confidence intervals of the mean predictions from robust linear regression model. The correlations are consistent regardless of administration route and number of doses.

There were a total of 18 subjects included in this analysis. (Table 1) Mean age was 41, 78% were male, and 84% white. For oral dosing, the following data were used for pharmacokinetic analysis: 139 plasma samples, 131 PBMC samples, 149 rectal tissue homogenates, 72 rectal tissue isolated MMC samples, 31 vaginal sponges, and 135 rectal sponges. For topical dosing, the following data were used for pharmacokinetic analysis: 228 plasma samples, 210 PBMC samples, 144 rectal tissue homogenates, 69 rectal tissue isolate MMC sample, 46 vaginal sponges, and 224 rectal sponges. For the purpose of concentration correlation analysis across matrices only, BLQ samples were excluded. Thus, 163 plasma, 319 PBMC, 208 rectal tissue homogenate, 107 rectal tissue MMC, 42 vaginal sponge, and 130 rectal sponge samples were excluded. All pharmacokinetic data are summarized in Table 2 and Figures 2 and 3. Matrix comparisons are presented in Figures 4–8.

**Table 1:** Demographics.

	Oral tenofovir	Tenofovir Gel	HEC placebo gel
**N**	18	12	6
**Age** (mean, STD)	42.1 (11.4)	41.3 (11.9)	43.7 (10.2)
**Gender**: M	14 (78%)	10 (83%)	4 (67%)
**Gender**: F	4 (22%)	2 (17%)	2 (33%)
**Latino or Hispanic Origin**: Y	5 (28%)	3 (25%)	2 (33%)
**Latino or Hispanic Origin**: N	13 (72%)	9 (75%)	4 (67%)
**Race**: Black or African-American	2 (11%)	1 (8%)	1 (17%)
**Race**: White	15 (83%)	10 (84%)	5 (83%)
**Race**: Other	1 (6%)	1 (8%)	0

**Table 2:** Noncompartmental Pharmacokinetic Parameters (*Insufficient data to perform NCA on CD4- and CD4+ PBMC; see companion publication Richardson-Harmon et.al for exposure-response analysis; **Composite Profile).

Matrix	Analyte, PK parameter	Single Oral Dose, median (min–max); N	Single Rectal Dose median (min–max); N	Multiple Rectal Dose median (min–max); N
Plasma	TFV T1/2 (h)	10.8 (6.82–19.2); 18	4.56 (2.61–62.9); 12	6.62 (4.55–32.1); 10
	TFV AUC24 h (ng/mL ×h)	2210 (1100–2940); 18	66.3 (12.4–114); 12	46.5 (17.1–178); 10
	TFV Cmax (ng/mL)	252 (76.8–387); 18	10.5 (5.08–33.4); 12	8.61 (2.37–11.2); 10
	TFV Tmax (h)	1.93 (0.267–3.92); 18	0.317 (0.183–1.98); 12	0.258 (0.183–2.03); 10
PBMC (total)^1^	TFVdp AUC24 h (fmol/10∧6 cells ×h)	4.26 (0.240–389)	0.24 (0.24–0.24)	0.24 (0.24–0.24)
	TFVdp Cmax (fmol/10∧6 cells)	0.375 (BLQ–38.6)	BLQ (BLQ–0.01)	BLQ (BLQ–0.01)
Rectal Tissue	TFV AUC24 h (ng/mg ×h)*	0.790	70.4	
	TFVdp AUC24 h (fmol/mg ×h)**	0.240	5470	
	TFV C24 h (ng/mg)	0.06 (BLQ–0.360)	0.06 (BLQ–12.6)	
	TFVdp C24 h (fmol/mg)	BLQ (BLQ–991)	285 (BLQ–490)	
	Accumulation Ratio, TFV			2.03
	Accumulation Ratio, TFdp			4.48
	TFV C30 min (ng/mg)		5.81 (BLQ–95.1); 12	11.8 (BLQ–430); 11
	TFVdp C30 min (fmol/mg)		176 (BLQ-1230); 12	789 (55.7–7190); 12
Rectal Mononuclear Cells	TFVdp C30 min (fmol/10∧6 cells)	BLQ (BLQ–BLQ); 18	454 (BLQ–1460); 12	1324 (BLQ–13900); 11
	TFVdp C24 h (fmol/10∧6 cells)	BLQ (BLQ–524); 8	228 (BLQ–290); 3	
	TFVdp CD4+ C30 min (fmol/10∧6 cells)	BLQ (BLQ–BLQ); 18	266 (BLQ–3950); 12	1080 (BLQ–31200); 12
	TFVdp CD4+ C24 h (fmol/10∧6 cells)	26.7 (BLQ–724); 18	250 (BLQ–1110); 3	
	TFVdp CD4- C30 min (fmol/10∧6 cells)	BLQ (BLQ–20); 18	112 (BLQ–1340); 12	330 (BLQ–12000); 12
	TFVdp CD4- C24 h (fmol/10∧6 cells)	28.4 (BLQ–157); 8	92.7 (BLQ–265); 3	
Rectal Fluid	TFV AUC24 h (ng/g ×h, ×10∧5)	1.03 (0.00265–15.1); 16	11.0 (1.56–42.5); 12	7.03 (1.49–32.6); 7
	TFV Cmax (ng/g, ×10∧4)	0.978 (0.00179–15.1); 17	73.9 (8.79–297); 12	41.6 (10.1–87.2); 7
	TFV Tmax (h)	24 (0.330–24.5); 4	0.380 (0.300–2.08); 12	0.330 (0.280–0.370); 7
Vaginal Fluid	TFV AUC24 h (ng/g ×h)	2.33 (0.979–2.72); 4	1.43 (0.587–2.27); 2	11.8; 1
	TFV Cmax (ng/g, ×10∧4)	0.134 (0.0515–0.182); 4	0.313 (0.0432–0.582); 2	0.696; 1
	TFV Tmax (h)	3.01 (1.92–24); 4	2.74 (1.62–3.87); 2	3.58; 1

*Insufficient data to perform NCA on CD4- and CD4+ PBMC; see companion publication Richardson-Harmon et.al for exposure-response analysis.

**Composite Profile.

### Plasma Pharmacokinetics

As expected, systemic TFV exposure, measured by both AUC_24 h_ and C_max_, was 24–33 fold higher after a single oral dose (median AUC_24 h_ 2200 ng/mL ×h, median C_max_ 250 ng/mL) than after a single rectal dose (median AUC_24 h_ 66 ng/mL ×h, median C_max_ 11 ng/mL). An accumulation ratio of 0.73 demonstrated that there was no clinically relevant difference in plasma exposure between single and multiple rectal dosing. A 24-fold lower C_max_ was achieved approximately 1.5 h faster with rectal dosing than with oral dosing (median T_max_ single rectal  = 0.32 h versus single oral  = 1.9 h; Table 2). TFV half-life was noted to be at least 5 h shorter (paired t-test on log-transformed half-lives, p = 0.02) for single and multiple rectal dosing (4.6–6.6 h) compared to oral dosing (11 h). (Table 2; Figure 3) Inter-individual variability of the PK parameters (CV%) ranged from 31–100% during oral dosing, 55–106% during single rectal dosing, and 53–103% during multiple rectal dosing. T_max_ was the most variable PK parameter with CV% consistently above 100%.

### PBMC

No detectable TFVdp was found in total PBMCs after single and multiple rectal dosing. (Table 2) In contrast, PBMC exposure after oral dosing was consistently detected in most (10/18) subjects (median AUC_24 h_ 4.3 fmol/10^6^ cells ×h, median C_max_ 0.38 fmol/10^6^ cells). There was insufficient data to perform NCA on CD4- and CD4+ cell subpopulations; please see companion paper (Richardson-Harmon et.al) for exposure-response analysis.

### Rectal Tissue

There was a linear relationship (p = 0.04) between TFVdp and TFV in rectal tissue homogenates during oral dosing. This relationship can be seen in Figure 4. The resulting model (± standard error) was as follows: Rectal Tissue homogenate TFVdp concentration (fmol/mg)  = 2.37 (±0.168)+0.366 (±0.121)× rectal tissue homogenate TFV concentration (ng/mg). Although rectal data from topical dosing was excluded from the model due to nonsignificance, they are overlaid in Figure 4 for reference.

Population composite AUC showed that topical dosing resulted in 2-log_10_ higher rectal exposure to TFV (single oral vs. single rectal dose AUC_24 h_  = 0.79 vs. 70 ng/mg ×h, Table 2) and a 4-log_10_ higher rectal exposure to TFVdp (single oral vs. rectal dose AUC_24 h_  = 0.24 vs. 5500 fmol/mg ×h). When comparing tissue concentrations 30 minutes post-dose, single topical dosing resulted in 2-fold higher TFV (paired p = 0.016) and 4-fold higher (paired p<0.001) TFVdp concentrations compared to oral dosing (Figures 4A, 4B). Multiple rectal dosing resulted in a non-statistically significant 2-fold accumulation in TFV concentrations compared to single dosing (paired P>0.8), with TFVdp concentrations accumulating 4.5-fold (paired p<0.01). Over 24 h, we observed TFV concentrations continuing to increase after oral dosing, such that 24 h concentrations post-dose were similar between oral and topical dosing: median TFV concentration at 24 h was 0.060 ng/mg after oral dosing vs 0.060 ng/mg after rectal dosing, paired p>0.8). However, median TFVdp concentrations 24 h post topical rectal dose were almost 300-fold higher than after an oral dose, though not statistically significant. (paired p = 0.125). With 4 subjects having missing paired C_24 h_ rectal biopsy samples for rectal dosing in Figure 5B, this may have resulted in biased TFVdp concentrations after topical dosing.

Isolated total mucosal mononuclear cells yielded similar results to tissue homogenates. At 0.5 h post-dose, rectal dosing resulted in 4-log_10_ higher concentration (paired p = 0.002) of TFVdp (median 454 fmol/10^6^ cells) compared to oral administration (median 0.010 fmol/10^6^ cells), and remained 4-log_10_ higher 24 h post dose (median rectal: 230 fmol/10^6^ cells, oral: 0.010 fmol/10^6^ cells, Table 2, Figure 5C, paired p = 0.5). There was also accumulation of TFVdp in these isolated cells after multiple rectal dosing (accumulation ratio: 2.9, paired p = 0.084).

Regardless of dosing route, compared to plasma, there was large inter-individual variability in TFV and TFVdp tissue and cell concentrations at each nominal time. Although no dosing route was found to have more variable exposure than the other. In tissue homogenates, TFV CV% ranged from 112–364%, 0 to 220%, and 236% for single oral, single rectal, and multiple rectal dosing, respectively. The CV% of TFVdp ranged from 0–307%, 85–312%, and 132% for single oral, single rectal, and multiple rectal dosing. The lower CV% of 0 in these groups is a result of all measured values being below the limit of quantification. The variability of TFV and TFVdp in isolated mucosal mononuclear cells was also similarly high regardless of CD4 expression status, and ranged from 0 to 316%.

Robust linear regression analysis demonstrated that TFVdp in isolated mucosal mononuclear cells was positively and linearly correlated with TFVdp in rectal tissue homogenates (Figure 6). The β_3_ term (see methods for the initial model) was dropped due to nonsignificance. Despite a large number of data points (85%) excluded from the analysis for concentrations below the limit of detection, the final model (± standard error) still achieved statistical significance (p<0.001), and was as follows: Cellular TFVdp concentration  = 0.680 (±0.205) +0.628 (± 0.0818) × homogenate TFVdp concentration +0.586 (±0.125) × cell type (cell type  = 0 for CD4- cells and cell type  = 1 for CD4+ cells). Though there is no interaction between cell type and TFVdp, the y-intercept of the CD4+ cells was significantly higher (0.680 vs 1.27; p<0.01) than that of the CD4- cells.

### Rectal Sponge

TFV exposure in rectal fluid was 1–2 log_10_ higher during topical administration (single dose median AUC_0–24 h_: 1,100,000 ng/g ×h, C_max_ 740,000 ng/g, Table 2) compared to oral administration (median AUC_0–24 h_: 100,000 ng/g ×h, C_max_ 9,800 ng/g). As expected, topical administration achieved maximal concentrations nearest the time of application (C_max_  = 0.38 h). However, oral administration achieved maximal concentrations 24 hours after dosing. There was no accumulation of TFV in rectal fluid after multiple rectal dosing (median AUC_24 h_ single dose 11.0×10^5^ ng/g ×h, multiple dose 7.03×10^5^ ng/g ×h). Inter-individual variability was higher for rectal sponge samples compared to direct sampling of other matrices. With oral dosing, single rectal dosing, and multiple rectal dosing, TFV concentration CV% ranged from 150–390%, 92–320%, and 56–150%, respectively.

Robust linear regression demonstrated that plasma TFV concentrations positively and linearly correlated with TFV in rectal fluid after topical dosing (Figure 7). No correlation between plasma concentrations and rectal fluid after oral dosing was noted. Both β_2_ and β_3_ terms were dropped due to nonsignificance, with the following model (± standard error) achieving statistical significance (p<0.001): Plasma TFV  = −1.23 (±0.170) +0.386 (±0.0328) × TFV rectal fluid concentration. The number of rectal doses did not significantly affect these concentration correlations.

Robust linear regression demonstrated that rectal tissue TFV and TFVdp concentrations positively and linearly correlated with TFV in rectal fluid, regardless of administration route (Figure 8). Thus, dose route was dropped as an interaction term. The following model (± standard error) achieving statistical significance (p<0.001): [Rectal Tissue TFV or TFVdp]  = β_0_+β_1_× [Rectal Fluid TFV] For predicting rectal tissue TFV, β_1,TFV_  = 0.634(±0.0777) (p<0.001) and β_0,TFV_  = −2.43(±0.335). For predicting rectal tissue TFVdp, β_1,TFVdp_  = 0.197 (±0.0612) (p<0.001), and β_0,TFVdp_  = 1.41 (±0.272). Dose route and frequency was not found to be significant factors in the correlation.

### Vaginal Sponge

TFV concentrations in the vaginal lumen peaked at approximately 3 hours regardless of route of administration. However, median TFV C_max_ in the vagina was approximately 2-fold higher after a single rectal dose than after a single oral dose (3100 ng/g vs. 1300 ng/g, Table 2). Additionally, from data in one subject only, there was evidence of accumulation in the vaginal lumen after multiple rectal dosing (accumulation ratio  = 5).

Less inter-individual variability was seen in the vaginal sponge data than the rectal sponge data. TFV concentration CV% at each nominal time from oral dosing, single rectal dosing, and multiple rectal dosing ranged from 50–140%, 40–120%, and 110–140%, respectively.

**Figure 9:**
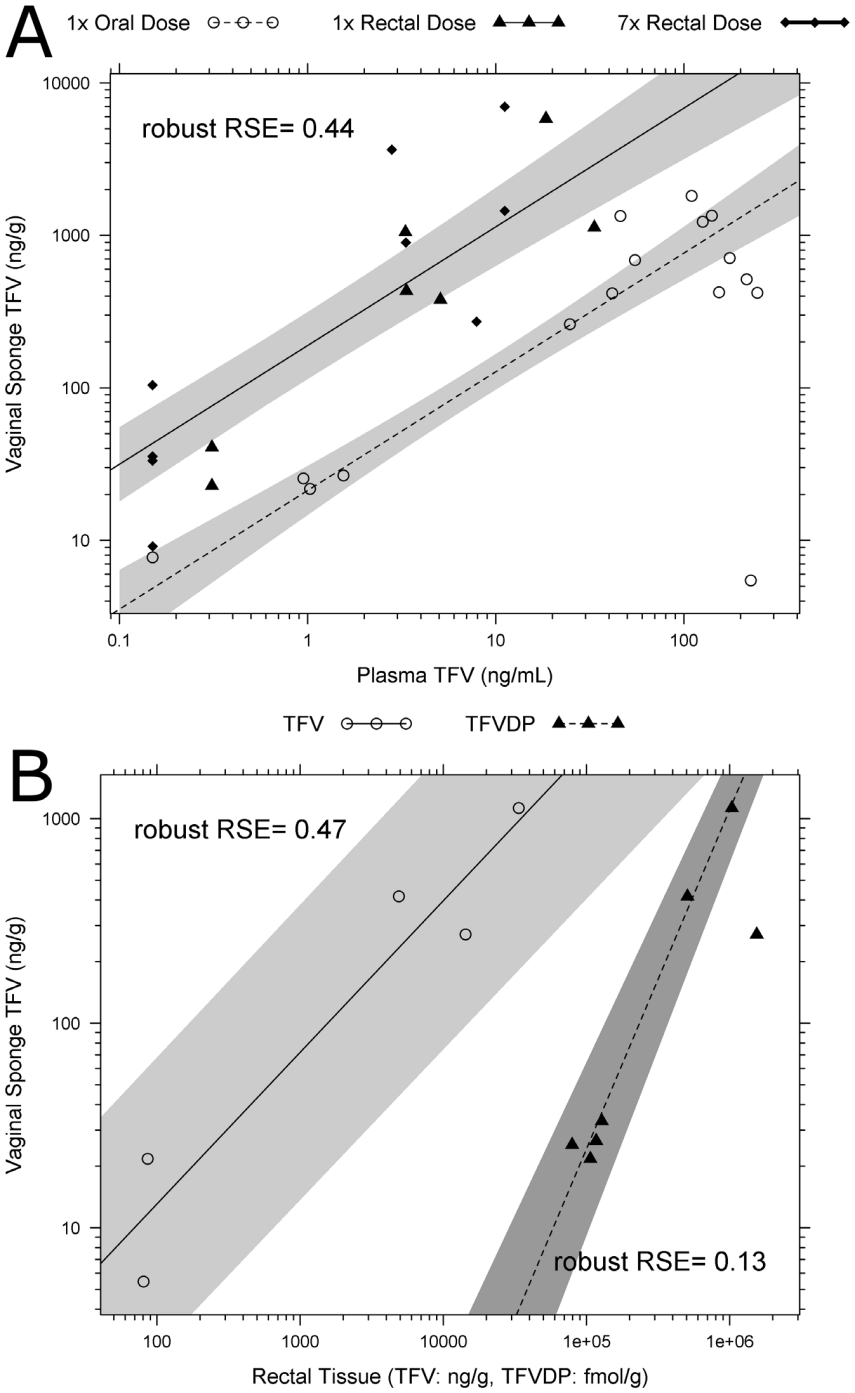
There is vaginal penetration of TFV from both oral and topical rectal exposures. (A) Vaginal fluid detection of both TFV and TFVdp concentration in vaginal fluid is higher following rectal dosing than following single oral dosing TFVdp. There is a linear correlation between vaginal fluid sponge TFV and plasma TFV concentrations (p<0.001, robust RSE  = 0.44). TFV penetration into vaginal fluid is 1-log_10_ higher with rectal administration than oral, seen with higher y-intercept. (p<0.001). (B) There is a linear correlation between vaginal fluid TFV and both rectal tissue TFV and TFVdp (p<0.01, robust RSE_TFV_  = 0.47, RSE_TFVdp_  = 0.13). Shaded regions are the 10–90% confidence intervals of the mean predictions from robust linear regression model. Solid line is mean vaginal fluid TFV concentration, dashed TFVdp.

Robust linear regression demonstrated that TFV concentrations on vaginal sponges are positively and linearly correlated with TFV concentrations in plasma (Figure 9A). The final predictive model (± std error) was: TFV in vaginal fluid  = 1.32 (±0.0948) +0.778 (±0.0704) × [plasma TFV] +0.951 (±0.169) × route. For oral dosing, route  = 0, and for rectal dosing, route  = 1. Though there was no interaction between dose route and TFV in vaginal fluid, the y-intercept of rectal administration was significantly higher (2.27 vs. 1.32; p<0.01) than that of oral administration.

Robust linear regression also demonstrated that TFV concentrations on vaginal sponges are positively and linearly correlated with TFV and TFVdp concentrations in rectal tissue. (Figure 9B) The final model (± std error) was as follows: [vaginal fluid TFV]  = β_0_+β_1_× [TFV or TFVdp in rectal tissue] For TFV in rectal tissue as an independent predictor of vaginal fluid concentrations, β_1,TFV_  = 0.741 (±0.105, p<0.001) and β_0,TFV_  = −0.37 (±0.402). For TFVdp in rectal tissue as an independent predictor of vaginal fluid concentrations, β_1,TFVdp_  = 1.66 (±0.114, p<0.001), and β_0,TFVdp_  = −6.91 (±0.649). TFV and TFVdp evaluations were performed as two separate analyses. The coefficients do not directly compare with each other since the predictor variables are independent and in different units. Nonetheless, this demonstrates that TFV vaginal fluid concentrations are significantly linearly correlated with rectal tissue TFV and TFVdp exposure within the concentration range of 0.10 to 10 ng/mg TFV and 30 to 1000 fmol/mg TFVdp.

## Discussion

This is the first study examining the pharmacokinetic distribution of rectally applied tenofovir gel in various tissues and cells relevant to HIV infection. This study also compares compartment concentrations of rectally applied TFV kinetics to oral dosing. It is important and biologically relevant to quantify TFV and TFVdp exposures in tissues and cells targeted for HIV infection. Measuring these concentrations allows for a better understanding of how drug distributes from the sites of absorption to target tissues, what factors can impact this distribution, and the possible identification of less-invasive surrogate markers of exposure that could be useful in larger clinical trials.

In this study's intra-subject comparison, the TFV exposure (AUC_24 h_) in plasma was, expectedly, more than 30 times higher after oral administration of 136 mg of TFV equivalent than after rectal administration of 44 mg of TFV. Additionally, T_max_ was 1.6 h shorter with rectal administration than with oral dosing. Differences in absorption kinetics between the rectum/lower large intestine and upper small intestine may explain this discrepancy. The extent and rate of absorption is often lower in the rectum compared to the intestines. This can be due to the relatively smaller surface area of the rectum for drug absorption, the inherent differences in the formulation, and the environment surrounding the route of administration, such as pH and fluid content [22]–[24]. In the particular case of TFV gel, the major difference is the formulation. Oral TFV is administered as tenofovir disoproxil fumarate, a di-ester prodrug [25], [26]. In contrast, TFV gel contains only the drug. Thus, TFV gel would not require the additional metabolism step, leading to a faster T_max_. The lower plasma exposure after gel dosing is not completely explained by the lower dose, since the equivalent oral dose was only 3 times higher. This is most likely due to the lower extent of drug absorption in the rectum, and also possibly due to gel leakage.

The half-life of TFV in plasma was 4–6 h longer (p = 0.02) with oral dosing than with rectal administration, and consistently longer in all patients except for one. The plasma half-life observed was 1–3 hours shorter than typically reported [25], [26] most likely due to lack of data in this study during the terminal elimination phase. Nonetheless, the relative difference in half-life between oral and rectal administration is significant. One possible explanation is that the kinetics with rectal dosing may be driven by absorption, leading to flip-flop kinetics [27]. If this is the true, then the half-life would be rate-limited by the rate of absorption instead of elimination. One other explanation may be the saturation of renal elimination processes [28] as a result of higher TFV plasma exposure. Tenofovir is eliminated by filtration and secretion [25]. Although renal filtration is not saturable, tubular secretion is carrier-protein mediated and can exhibit nonlinear, saturable behavior. During tubular secretion, tenofovir is a substrate for the MRP4 transporter in the tubular lumen [29]–[31]. Saturation of this excretion transporter could explain nonlinear elimination.

In the case of topical administration, the decreased half-life and lower plasma exposure during both single and multiple administrations can minimize systemic toxicity. C_max_ of TFVdp after a single oral dose was below limit of detection (LLOQ  = 8 fmol/10^6^ cells); this is significantly lower than what has been previously reported (20 fmol/10^6^ cells [9]), but not unexpected based on inter-individual variability. This is probably also due to lower cell penetration of TFV compared to TFVdp. Due to the low plasma exposure following rectal dosing (median plasma AUC_24 h_ 66 ng/mL ×h), TFVdp exposure was undetectable in PBMCs of all subjects.

As expected, rectal tissue exposure to TFV and TFVdp was 2–4 log_10_ higher with topical administration than with oral dosing. After multiple dosing, TFV does not appear to significantly accumulate in the tissue. Although the accumulation ratio from the median profile showed an approximately 2-fold increase in AUC_24 h_, a paired analysis of concentration within individuals showed no statistically significant changes between the 30 min and 24 h post-dose samples. TFVdp, however, did show significant accumulation in rectal tissue (approximately 5-fold) and in isolated mucosal mononuclear cells (approximately 3-fold). The long intracellular half-life of TFVdp is likely the cause of this tissue accumulation [32].

It has previously been noted that an increase in TFV tissue homogenate concentration yields an increase in TFVdp concentration [9], [32]. We confirmed this at 24 h after oral dosing (Figure 4). The correlation may have been stronger if there would have been more data to analyze. Furthermore, it is possible that the different absorption kinetics of the rectum compared to the intestines could cause variations in this correlation.

To address the question of whether TFVdp in tissue homogenates accurately reflect TFVdp concentrations in target cells for HIV transmission, or whether the heterogeneous mix of cells in mucosal tissue confounds the results, we compared homogenate results to those of isolated mucosal mononuclear cells. Encouragingly, a linear relationship was noted between TFVdp concentration in rectal tissue homogenate and isolated mucosal mononuclear cells. This linear relationship was not influenced by CD4 status: as TFVdp concentration in the homogenate increased by 100%, there was a 63% increase of TFVdp in the isolated mucosal mononuclear cells. However, the data did suggest that there may be differences in phosphorylation based on the CD4 expression status of the cell. When plotting the relationship between TFVdp in tissue homogenates and in CD4+ and CD4- cells, the Y intercept in CD4+ cells was 1.6-fold higher than in CD4- cells. That is, for every observed TFVdp concentration in the homogenate, the TFVdp concentration in the CD4+ cells was 1.6-fold higher than CD4- cells. This difference persisted throughout the 10–10,000 fmol/mg range of TFVdp concentrations, and achieved statistical significance (p<0.01), even with small numbers of samples. The source of this difference is unclear, as some studies suggest that tenofovir is phosphorylated to a similar extent between quiescent and stimulated cells, while others suggest higher phosphorylation in resting cells [33]. Although it is currently unknown whether these concentration differences are of clinical significance, these data are encouraging, as they suggest that TFVdp is found in higher concentrations in the cells that are targets for HIV infection.

In this study, rectal mucosal fluid was collected to determine whether it could be a surrogate for TFV and TFVdp concentration in rectal tissue. TFV concentrations in the rectal fluid linearly correlated with rectal tissue TFVdp. (Figure 8) This relationship remained consistent regardless of dose route or frequency. Despite dramatic differences between oral and rectal absorption characteristics, we still observe a similar linear relationship between these two matrices. Therefore, rectal fluid TFV concentrations collected by sponge are useful in estimating drug concentrations in the target rectal tissue.

We also attempted prediction of plasma TFV exposure from rectal mucosal concentrations. There was high variability in these predictions, so a precise prediction was not possible. However, our data suggest that a rough estimation of high or low plasma TFV based on rectal TFV concentration could be feasible. Therefore, rectal sponge TFV concentration could be used as a non-invasive surrogate for plasma TFV concentrations for safety and toxicity monitoring. With more patient data, prediction variability may be decreased using patient demographic covariates, which could potentially minimize the need for blood sampling in future trials of rectal dosing. We did not observe similar correlation with oral dosing; this is probably due to the high variability in the data compared to the possible strength of the correlation. If we had a higher range of rectal fluid concentration, through either different oral dose levels or sample collection times, then it is possible a correlation may be observed.

Previous data in macaques has demonstrated that 5–7% of tenofovir dosed rectally can be found in vaginal fluid [34]. We evaluated this phenomenon in 4 women, and found that vaginal concentrations were 8.7% of rectal secretion concentrations sampled at the same time, similar to the 1–2 log_10_ difference found previously in animals. Additionally, we noted a 1.6-fold higher exposure of TFV in vaginal secretions with rectal dosing than with oral dosing. Since the slopes of the relationship between vaginal fluid TFV and plasma TFV with the two administration routes are not significantly different, the rate of TFV penetration into the vagina does not vary with administration route. As plasma concentrations with oral dosing are much higher than with rectal dosing, systemic re-distribution into the vaginal fluid cannot be the only mechanism by which TFV reaches the vaginal lumen. Vaginal TFV penetration was further confirmed when we observed a linear relationship between vaginal TFV exposure and rectal tissue exposure. When rectal tissue TFV and TFVdp exposure increased, there was also a linear increase in vaginal exposure to TFV. Due to the low number of data points, we could not discern whether route of administration affected this relationship.

It is interesting to note that there was accumulation of TFV in the vaginal fluid (Table 2, single rectal dose AUC  = 1.4 ng/g ×h, multiple rectal dose  = 11), but not rectal fluid (single rectal dose AUC  = 11 ng/g ×h, multiple rectal dose  = 7.0). There are two possible explanations for this. One is that the rectal site is already saturated due to proximity to administration, whereas vaginal site is more distal and takes time to build up. Another is that there may be differences in fluid turnover between the two sites. Therefore, the kinetics is inherently different between the two sites.

One limitation of this analysis is the treatment of BLQ values and nominal times used for the composite PK profiles in Table 2. BLQ numbers are still valuable because there is a lot of information content in these numbers. Therefore, the treatment of BLQ values depended on the analysis performed. They are imputed as 0.01 for NCA, and ignored for correlation analysis due to statistical difficulties in treating these numbers. Since this was done systematically and consistently, there should be no impact on the overall conclusions. Also, the proportion of BLQ values for plasma was low (2.4%), so this should have little impact on the calculated plasma PK parameters. Similarly, since only datapoints that fell within a specific time window were included in the composite profiles, there should not have been a significant bias in PK parameter estimates.

## Conclusion

This was the first comprehensive pharmacokinetic study of rectally administered tenofovir gel that describes the distribution of TFV and TFVdp into various tissue compartments relevant to HIV infection. The compartments included rectal fluid, rectal tissue and its isolated mucosal mononuclear cells, vaginal fluid, blood plasma and PBMCs. It was expected, and now confirmed, that rectally applied TFV would have lower systemic exposure and higher vaginal exposure compared to oral. There was accumulation of TFVdp in the rectal tissue, due to the long intracellular half-life. An unexpected yet biologically interesting finding was detecting a consistent difference in concentrations of TFVdp in rectal mucosal CD4+ cells, prime targets for new HIV infections. It is useful to know that TFV rectal fluid concentrations may be reasonable bio-indicators of plasma and, importantly, rectal tissue concentrations, making it easier to estimate adherence and TFV concentrations in the target tissue.

We were encouraged to see many concentration correlations across various relevant matrices, such as rectal mucosal mononuclear cells and rectal tissue homogenate TFVdp, and vaginal fluid and plasma. This will enable more advanced population pharmacokinetic modeling methods and developing a single mathematical model that describes the distribution of TFV with more accuracy. Furthermore, if target TFV and TFVdp concentrations are identified, then these data and models will assist in dose/regimen selection that maximizes efficacy and minimizes toxicity.

## Supporting Information

Protocol S1 **Trial Protocol.**
(PDF)

Checklist S1 **CONSORT Checklist.**
(DOC)

## References

[1] Abdool Karim Q, Abdool Karim SS, Frohlich JA, Grobler AC, Baxter C, et al (2010) Effectiveness and safety of tenofovir gel, an antiretroviral microbicide, for the prevention of HIV infection in women. Science 329: 1168–1174 Available: http://www.pubmedcentral.nih.gov/articlerender.fcgi?artid=3001187&tool=pmcentrez&rendertype=abstract Accessed 31 January 2013.. 20643915 10.1126/science.1193748 PMC3001187

[2] Grant RM, Lama JR, Anderson PL, McMahan V, Liu AY, et al (2010) Preexposure chemoprophylaxis for HIV prevention in men who have sex with men. N Engl J Med 363: 2587–2599 Available: http://www.pubmedcentral.nih.gov/articlerender.fcgi?artid=3079639&tool=pmcentrez&rendertype=abstract Accessed 29 January 2013.. 21091279 10.1056/NEJMoa1011205 PMC3079639

[3] Baeten JM, Donnell D, Ndase P, Mugo NR, Campbell JD, et al (2012) Antiretroviral prophylaxis for HIV prevention in heterosexual men and women. N Engl J Med 367: 399–410 Available: http://www.ncbi.nlm.nih.gov/pubmed/22784037 Accessed 22 May 2013.. 22784037 10.1056/NEJMoa1108524 PMC3770474

[4] Choopanya K, Martin M, Suntharasamai P, Sangkum U, Mock PA, et al (2013) Antiretroviral prophylaxis for HIV infection in injecting drug users in Bangkok, Thailand (the Bangkok Tenofovir Study): a randomised, double-blind, placebo-controlled phase 3 trial. Lancet 381: 2083–2090 Available: http://www.ncbi.nlm.nih.gov/pubmed/23769234 Accessed 30 October 2013.. 23769234 10.1016/S0140-6736(13)61127-7

[5] Karim SSA, Kashuba ADM, Werner L, Karim QA (2011) Drug concentrations after topical and oral antiretroviral pre-exposure prophylaxis: implications for HIV prevention in women. Lancet 378: 279–281 Available: http://www.pubmedcentral.nih.gov/articlerender.fcgi?artid=3652579&tool=pmcentrez&rendertype=abstract Accessed 27 May 2013.. 21763939 10.1016/S0140-6736(11)60878-7 PMC3652579

[6] Anderson PL, Glidden D V, Liu A, Buchbinder S, Lama JR, et al (2012) Emtricitabine-tenofovir concentrations and pre-exposure prophylaxis efficacy in men who have sex with men. Sci Transl Med 4: 151ra125 Available: http://www.ncbi.nlm.nih.gov/pubmed/22972843 Accessed 18 July 2013.. 10.1126/scitranslmed.3004006 PMC3721979 22972843

[7] Anton PA, Cranston RD, Kashuba A, Hendrix CW, Bumpus NN, et al (2012) RMP-02/MTN-006: A phase 1 rectal safety, acceptability, pharmacokinetic, and pharmacodynamic study of tenofovir 1% gel compared with oral tenofovir disoproxil fumarate. AIDS Res Hum Retroviruses 28: 1412–1421 Available: http://www.ncbi.nlm.nih.gov/pubmed/22943559 Accessed 31 January 2013.. 22943559 10.1089/aid.2012.0262 PMC3484811

[8] Anton PA, Saunders T, Elliott J, Khanukhova E, Dennis R, et al (2011) First phase 1 double-blind, placebo-controlled, randomized rectal microbicide trial using UC781 gel with a novel index of ex vivo efficacy. PLoS One 6: e23243 Available: http://www.pubmedcentral.nih.gov/articlerender.fcgi?artid=3182160&tool=pmcentrez&rendertype=abstract Accessed 2013 July 18.. 21969851 10.1371/journal.pone.0023243 PMC3182160

[9] Louissaint NA, Cao Y-J, Skipper PL, Liberman RG, Tannenbaum SR, et al. (2013) Single Dose Pharmacokinetics of Oral Tenofovir in Plasma, Peripheral Blood Mononuclear Cells, Colonic Tissue, and Vaginal Tissue. AIDS Res Hum Retroviruses. Available: http://www.ncbi.nlm.nih.gov/pubmed/23600365. Accessed 2013 July 18. 10.1089/aid.2013.0044 PMC3809387 23600365

[10] Louissaint NA, Fuchs EJ, Bakshi RP, Nimmagadda S, Du Y, et al (2012) Distribution of cell-free and cell-associated HIV surrogates in the female genital tract after simulated vaginal intercourse. J Infect Dis 205: 725–732 Available: http://www.ncbi.nlm.nih.gov/pubmed/22279121 Accessed 2013 July 18.. 22279121 10.1093/infdis/jir841 PMC6281406

[11] Beigi R, Noguchi L, Parsons T, Macio I, Kunjara Na Ayudhya RP, et al (2011) Pharmacokinetics and placental transfer of single-dose tenofovir 1% vaginal gel in term pregnancy. J Infect Dis 204: 1527–1531 Available: http://www.pubmedcentral.nih.gov/articlerender.fcgi?artid=3192189&tool=pmcentrez&rendertype=abstract Accessed 2013 July 18.. 21930612 10.1093/infdis/jir562 PMC3192189

[12] Keller MJ, Madan RP, Torres NM, Fazzari MJ, Cho S, et al (2011) A randomized trial to assess anti-HIV activity in female genital tract secretions and soluble mucosal immunity following application of 1% tenofovir gel. PLoS One 6: e16475 Available: http://www.pubmedcentral.nih.gov/articlerender.fcgi?artid=3026837&tool=pmcentrez&rendertype=abstract Accessed 2 February 2013.. 21283552 10.1371/journal.pone.0016475 PMC3026837

[13] King T, Bushman L, Kiser J, Anderson PL, Ray M, et al (2006) Liquid chromatography-tandem mass spectrometric determination of tenofovir-diphosphate in human peripheral blood mononuclear cells. J Chromatogr B Analyt Technol Biomed Life Sci 843: 147–156 Available: http://www.ncbi.nlm.nih.gov/pubmed/16828350 Accessed 2 February 2013.. 10.1016/j.jchromb.2006.05.043 16828350

[14] Hendrix CW, Chen BA, Guddera V, Hoesley C, Justman J, et al (2013) MTN-001: randomized pharmacokinetic cross-over study comparing tenofovir vaginal gel and oral tablets in vaginal tissue and other compartments. PLoS One 8: e55013 Available: http://www.pubmedcentral.nih.gov/articlerender.fcgi?artid=3559346&tool=pmcentrez&rendertype=abstract Accessed 27 May 2013.. 23383037 10.1371/journal.pone.0055013 PMC3559346

[15] R Core Team (2013) R: A Language and Environment for Statistical Computing. Available: http://www.r-project.org/.

[16] Sarkar D (n.d.) Lattice: Multivariate Data Visualization with R (Use R!). Springer. Available: http://www.amazon.com/Lattice-Multivariate-Data-Visualization-Use/dp/0387759689. Accessed 2 February 2013.

[17] Sarkar D, Andrews F (2012) latticeExtra: Extra Graphical Utilities Based on Lattice. Available: http://cran.r-project.org/package=latticeExtra.

[18] Wickham H (2011) The split-apply-combine strategy for data analysis. J Statis. Available: http://citeseerx.ist.psu.edu/viewdoc/download?doi=10.1.1.182.5667&rep=rep1&type=pdf. Accessed 2 February 2013.

[19] Wickham H (2007) Reshaping data with the reshape package. J Stat Softw. Available: http://www.had.co.nz/reshape/introduction.pdf. Accessed 2 February 2013.

[20] Wang J, Zamar R, Marazzi A, Yohai V, Salibian-Barrera M, et al. (2013) robust: Robust Library. Available: http://cran.r-project.org/package=robust.

[21] Hampel FR, Ronchetti EM, Rousseeuw PJ, Stahel WA (2005) Robust Statistics. Hoboken, NJ, USA: John Wiley & Sons, Inc.

[22] Van Hoogdalem E, de Boer AG, Breimer DD (1991) Pharmacokinetics of rectal drug administration, Part I. General considerations and clinical applications of centrally acting drugs. Clin Pharmacokinet 21: 11–26 Available: http://www.ncbi.nlm.nih.gov/pubmed/1717195 Accessed 17 July 2013.. 1717195 10.2165/00003088-199121010-00002

[23] Van Hoogdalem EJ, de Boer AG, Breimer DD (1991) Pharmacokinetics of rectal drug administration, Part II. Clinical applications of peripherally acting drugs, and conclusions. Clin Pharmacokinet 21: 110–128 Available: http://www.ncbi.nlm.nih.gov/pubmed/1884566 Accessed 17 July 2013.. 1884566 10.2165/00003088-199121020-00003

[24] De Boer AG, Moolenaar F, de Leede LG, Breimer DD (n.d.) Rectal drug administration: clinical pharmacokinetic considerations. Clin Pharmacokinet 7: 285–311 Available: http://www.ncbi.nlm.nih.gov/pubmed/6126289 Accessed 17 July 2013.. 10.2165/00003088-198207040-00002 6126289

[25] Fung HB, Stone EA, Piacenti FJ (2002) Tenofovir disoproxil fumarate: a nucleotide reverse transcriptase inhibitor for the treatment of HIV infection. Clin Ther 24: 1515–1548 Available: http://www.ncbi.nlm.nih.gov/pubmed/12462284 Accessed 18 July 2013.. 12462284 10.1016/s0149-2918(02)80058-3

[26] Kearney BP, Flaherty JF, Shah J (2004) Tenofovir disoproxil fumarate: clinical pharmacology and pharmacokinetics. Clin Pharmacokinet 43: 595–612 Available: http://www.ncbi.nlm.nih.gov/pubmed/15217303 Accessed 18 July 2013.. 15217303 10.2165/00003088-200443090-00003

[27] Yáñez JA, Remsberg CM, Sayre CL, Forrest ML, Davies NM (2011) Flip-flop pharmacokinetics—delivering a reversal of disposition: challenges and opportunities during drug development. Ther Deliv 2: 643–672 Available: http://www.pubmedcentral.nih.gov/articlerender.fcgi?artid=3152312&tool=pmcentrez&rendertype=abstract Accessed 16 December 2013.. 21837267 10.4155/tde.11.19 PMC3152312

[28] Van Ginneken CA, Russel FG (1989) Saturable pharmacokinetics in the renal excretion of drugs. Clin Pharmacokinet 16: 38–54 Available: http://www.ncbi.nlm.nih.gov/pubmed/2650954 Accessed 17 July 2013.. 10.2165/00003088-198916010-00003 2650954

[29] Rodríguez-Nóvoa S, Labarga P, Soriano V, Egan D, Albalater M, et al (2009) Predictors of kidney tubular dysfunction in HIV-infected patients treated with tenofovir: a pharmacogenetic study. Clin Infect Dis 48: e108–16 Available: http://www.ncbi.nlm.nih.gov/pubmed/19400747 Accessed 17 July 2013.. 19400747 10.1086/598507

[30] Imaoka T, Kusuhara H, Adachi M, Schuetz JD, Takeuchi K, et al (2007) Functional involvement of multidrug resistance-associated protein 4 (MRP4/ABCC4) in the renal elimination of the antiviral drugs adefovir and tenofovir. Mol Pharmacol 71: 619–627 Available: http://www.ncbi.nlm.nih.gov/pubmed/17110501 Accessed 17 July 2013.. 17110501 10.1124/mol.106.028233

[31] Ray AS, Cihlar T, Robinson KL, Tong L, Vela JE, et al (2006) Mechanism of active renal tubular efflux of tenofovir. Antimicrob Agents Chemother 50: 3297–3304 Available: http://www.pubmedcentral.nih.gov/articlerender.fcgi?artid=1610069&tool=pmcentrez&rendertype=abstract Accessed 30 May 2013.. 17005808 10.1128/AAC.00251-06 PMC1610069

[32] Patterson KB, Prince HA, Kraft E, Jenkins AJ, Shaheen NJ, et al (2011) Penetration of tenofovir and emtricitabine in mucosal tissues: implications for prevention of HIV-1 transmission. Sci Transl Med 3: 112re4 Available: http://www.pubmedcentral.nih.gov/articlerender.fcgi?artid=3483088&tool=pmcentrez&rendertype=abstract Accessed 31 May 2013.. 10.1126/scitranslmed.3003174 PMC3483088 22158861

[33] Robbins BL, Wilcox CK, Fridland A, Rodman JH (2003) Metabolism of tenofovir and didanosine in quiescent or stimulated human peripheral blood mononuclear cells. Pharmacotherapy 23: 695–701 Available: http://www.ncbi.nlm.nih.gov/pubmed/12820810 Accessed 18 July 2013.. 12820810 10.1592/phco.23.6.695.32189

[34] Nuttall J, Kashuba A, Wang R, White N, Allen P, et al (2012) Pharmacokinetics of tenofovir following intravaginal and intrarectal administration of tenofovir gel to rhesus macaques. Antimicrob Agents Chemother 56: 103–109 Available: http://www.pubmedcentral.nih.gov/articlerender.fcgi?artid=3256015&tool=pmcentrez&rendertype=abstract Accessed 18 July 2013.. 21986823 10.1128/AAC.00597-11 PMC3256015

