# Supplementary material for: A Multi-Compartment Single and Multiple Dose Pharmacokinetic Comparison of Rectally Applied Tenofovir 1% Gel and Oral Tenofovir Disoproxil Fumarate
Source: PLoS One. 2014 Oct 28;9(10):e106196. doi: 10.1371/journal.pone.0106196 (PMC4211672; doi:10.1371/journal.pone.0106196)
Supplement: Protocol S1 — Trial Protocol. (PDF) [file pone.0106196.s001.pdf]

PROTOCOL S1

IPCP Rectal Microbicide Program/ Microbicide Trials Network
CLARIFICATION MEMO #03 TO:

RMP-02/MTN-006
A two-site, Phase 1, partially-blinded, placebo-controlled safety, acceptability, and pharmacokinetic trial of topical, vaginally-formulated tenofovir 1% gel applied rectally compared with oral 300 mg tenofovir disoproxil fumarate in HIV-1 seronegative adults

Version 1.0/07 April 2009
DAIDS Document ID #10769
CONRAD IND # 73,382

Date of Clarification Memorandum: 16 November 2009

Section 1: Summary of Clarifications and Rationale

The items clarified in this Clarification Memorandum (CM) have been approved by the NIAID Medical Officer and are to be implemented immediately upon issuance. IRB approval of this CM is not required by the sponsor; however, investigators may submit the CM to the IRB overseeing the study at their site for information. This CM is official RMP-02/MTN-006 documentation and is effective immediately. A copy of this CM must be retained in each study site's Essential Documents file for RMP-02/MTN-006. No change in informed consent is necessitated by or included in this CM.

The primary goal for this CM is to modify the protocol to reflect the allowable windows for post-dose specimen collection timepoints at Visits 3, 7, and 12. Note that efforts will be made to collect the specimens in approximately the same order detailed in the RMP-02/MTN-006 SSP. The goal will be to collect the last sample (tissue biopsies) by the target collection timepoint. A change previously noted in RMP-02/MTN-006, CM #02 is also included in this CM. The Protocol Team Roster is also updated.

Section 2: Implementation

Text to be deleted is noted by ~~strikethrough~~ and text to be added is noted below in **bold**.

1. The following sections of the protocol are updated to reflect allowable windows for post-dose specimen collection timepoints **(+/- 15 minutes** for the 30 minute timepoint and **+/- 30 minutes** for 2- and 4-hour timepoints).

Table 15: Visit 3 and 7 (Study Product #1; Study Product #2 and 30' Sampling for each), Female Pelvic Specimens row, note to first bullet, and *Rectal Specimens* row, notes to fourth and seventh bullets

Table 19: Visit 12 (Sampling Following Once Daily Exposure for 7 Days (7th dose given in clinic)), Female Pelvic Specimens row, note to first bullet, and *Rectal Specimens* row, notes to fourth and seventh bullets

Section 7.11.1, Pharmacokinetic Procedures: Single Oral Dose

Section 7.11.2, Pharmacokinetic Procedures: Single Rectally Applied Dose

*Section 7.~~10~~**11**.3, Pharmacokinetic Procedures: Following 7-Day Rectally Applied Dose*

2. The following individual has been removed from the Protocol Team Roster: Nancy Connolly

3. The following item was previously noted in RMP-02/MTN-006, CM #02, dated 23 September 09:

The protocol is updated to reflect that PK testing will be performed for all compartments at all PK Visits 2, 5, 6, and 9 have been updated to reflect this:

Appendix 1: Schedule of Study Visits and Evaluations is modified accordingly:

	Visit 1	Visit 2	Visit 3	Visit 4	Visit 5	Visit 6	Visit 7	Visit 8	Visit 9	Visit 10	Visit 11	Visit 12	Visit 13/Early Term	F/U Call	Interim
Histology		X	X		X	**X**	X		X	**X**		X			

The above information will be incorporated into the next version of the protocol at a later time if it is amended.

IPCP Rectal Microbicide Program/ Microbicide Trials Network
CLARIFICATION MEMO #02 TO:

RMP-02/MTN-006
A two-site, Phase 1, partially-blinded, placebo-controlled safety, acceptability, and pharmacokinetic trial of topical, vaginally-formulated tenofovir 1% gel applied rectally compared with oral 300 mg tenofovir disoproxil fumarate in HIV-1 seronegative adults

Version 1.0/07 April 2009
DAIDS Document ID #10769
CONRAD IND # 73,382

Date of Clarification Memorandum: 23 September 2009

Section 1: Summary of Clarifications and Rationale

The items clarified in this Clarification Memorandum (CM) have been approved by the NIAID Medical Officer and are to be implemented immediately upon issuance. IRB approval of this CM is not required by the sponsor; however, investigators may submit the CM to the IRB overseeing the study at their site for information. This CM is official RMP-02/MTN-006 documentation and is effective immediately. A copy of this CM must be retained in each study site's Essential Documents file for RMP-02/MTN-006. No change in informed consent is necessitated by or included in this CM.

The primary goal for this CM is to modify the protocol to correctly reflect that PK testing will be performed in all compartments. This CM also clarifies that tenofovir levels will be measured in mucosal mononuclear cells (MMC).

Section 2: Implementation

Text to be deleted is noted by ~~strikethrough~~ and text to be added is noted below in **bold**.

1. The following sections of the protocol are updated to correctly reflect that PK testing will be performed for all compartments at all PK visits. Visits 2, 5, 6, and 9 have been updated to reflect this.

Table 9: Schedule of Study Endpoints:

**Visit 2: Enrollment/Baseline**	Blood, fluids from sponges, stool, endoscopic lavage, biopsies (~17)***	✓***		✓			✓	✓	✓	✓	✓	✓	✓	✓	✓	✓
**Visit 5 A/B: (Day 1-3, or 4-6)**	Blood, fluids from sponges, stool, endoscopic lavage, biopsies (~17)			✓			✓	✓	✓	✓	✓	✓	✓	✓	✓~~*~~	✓
**Visit 9A/B: (Day 1-3, or 4-6)**	Blood, fluids from sponges, stool, endoscopic lavage, biopsies (~17)			✓			✓	✓	✓	✓	✓	✓	✓	✓	✓~~*~~	✓

~~*Visits 5 and 9: No flow will be done at these visits.~~

Table 14: Visit 2 (Enrollment/Baseline Evaluation Visit-All Participants), a sub-bullet is added to blood specimens to reflect specimen collection for **Plasma and PBMC tenofovir levels**. A sub-bullet is also added to the 5th bullet of the Rectal Specimens row to reflect the measurement of **Tenofovir levels in MMC** at this visit.

Table 17: Visits 5 and 6 and Visits 9 and 10 (2-Week Sampling Period), references to ~~(Visits 5 and 9 only)~~ are removed from the 4th bullet, 1st and 3rd sub-bullets.

Appendix 1: Schedule of Study Visits and Evaluations is modified accordingly:

Plasma tenofovir levels		**X**	X	X	X	X	X	X	X	X	X	X	X		
PBMC tenofovir levels		**X**	X	X	X	X	X	X	X	X	X	X	X		
~~CD4 cells (~~TFV levels in MMC~~)~~		**X**	X		X	**X**	X		X	**X**		X			
Histology		X	X		X	**X**	X		X			X			

2. References to CD4 cells ~~(Tenofovir in MMC)~~ are replaced with **Tenofovir levels in MMC** in the following sections of the protocol:

   Table 15: Visit 3 and Visit 7 (Study Product #1; Study Product #2 and 30" sampling) and *Table 19: Visit 12 (Sampling Visit Following Once Daily Exposure for 7 Days (7th dose given in clinic))*, Rectal Specimens, 6th bullet, 2nd (reference to ~~tenofovir in MMC~~ is-deleted) and 3rd sub-bullets (**Tenofovir levels in MMC** is added).

   Table 17: Visits 5 and 6 and Visits 9 and 10 (2-Week Sampling Period), 4th bullet, 3rd sub-bullet.

The above information will be incorporated into the next version of the protocol at a later time if it is amended.

Microbicide Trials Network
LETTER OF AMENDMENT #01 TO:

RMP-02/MTN-006
DAIDS Document ID #10769

A two-site, Phase 1, partially-blinded, placebo-controlled safety, acceptability, and pharmacokinetic trial of topical, vaginally-formulated tenofovir 1% gel applied rectally compared with oral 300 mg tenofovir disoproxil fumarate in HIV-1 seronegative adults

Version 1.0/07 April 2009

CONRAD IND # 73, 382

Letter of Amendment Date: 17 August 2009

Information/Instructions to Study Sites from the Division of AIDS

The information contained in this Letter of Amendment (LoA) impacts the RMP-02/MTN-006 study and must be forwarded to your Institutional Review Board (IRB) and/or Ethics Committee (EC) as soon as possible for their information and review. IRB/EC approval is required before implementation of the revisions contained in this LoA.

The following information will also impact the sample informed consent. Site IRBs/ECs are responsible for assessing whether and how the changes included in this LoA are to be communicated to study participants. All IRB/EC requirements must be followed.

Please file this LoA and all associated IRB/EC correspondence in your essential documents files for RMP-02/MTN-006.

Summary of Revisions and Rationale

This LoA does not impact the overall design and study visit schedule for RMP-02/MTN-006. This LoA provides clarification on the following items:

1. Modifications to Section 8 to update communication pattern regarding adverse event and pregnancy reporting. These changes do not impact the degree or details of safety reporting in RMP-02/MTN-006
2. Inclusion of additional rectal microflora sampling time point per FDA request
3. Elimination references to INR and PTT in Section 5.3, Exclusion Criteria, as they are not included in the Screening Procedures
4. Removal of excessive detail regarding sponge types in the protocol that could potentially be in conflict with the details provided in the RMP-02/MTN-006 SSP manual
5. Clarifications to the informed consent documents to achieve consistency with the protocol

6. Other minor corrections and updates

Section 2: Implementation

Text to be deleted is noted by ~~strikethrough~~ and text to be added is noted below in **bold**.

1. **The following changes are made to Section 8, Assessment of Safety to reflect the guidelines put forth in the RMP-02/MTN-006 Clinical Trials Agreement.**

Section 8.1, Safety Monitoring, second paragraph, first sentence:

First sentence:

The DSMB will be comprised of the Chair, who will be an MD/DO, a representative from UCLA and from Pittsburgh/Magee, one of which may be the Chair, ~~CONRAD MO,~~ and a Biostatistical Representative.

Section 8.1, Safety Monitoring, second paragraph, first sentence:

For all DSMB meetings, there will be an open session accessible to all interested parties, followed by a closed session, limited to DSMB members only. The RMP Regulatory Core will file a summary report of DSMB recommendations with DAIDS, CONRAD, and Gilead after each DSMB meeting.

Section 8.2, Clinical Data Safety Review, second paragraph:

The U19 MDP Regulatory staff (Core B) will review incoming safety data on an ongoing basis. Events identified as questionable, inconsistent, or unexplained will be queried for verification. Adverse event reports requiring expedited handling will be submitted by ~~the Regulatory Core within 3 business days to:~~ **each participating study site to the DAIDS RCC Safety Office (via DAIDS Adverse Events Reporting System DAERS), and RCC will in turn distribute reports to the DAIDS Medical Officer, CONRAD Medical Officer, and Gilead (when the reports are related to the Gilead study product (i.e. oral tablets)), as well as UCLA/Pitt IRBs and RMP staff for review.**

- ~~DAIDS MO~~
- ~~CONRAD~~
- ~~Gilead~~
- ~~Site IRBs~~
- ~~RMP staff~~

When indicated, the FDA will be notified through CONRAD (the IND holder). **See Section 8.4 for complete description of this process.**

Section 8.2, Clinical Data Safety Review, last paragraph:
In the unlikely event that the protocol team or DSMB has serious safety concerns that lead to a decision to permanently discontinue study products for all participants and stop accrual

into the study, the protocol team or DSMB will request a review of the data by the DAIDS **and CONRAD** Medical Officer**s** before recommending that the study be stopped. If at any time, a decision is made to discontinue one or more study products in all participants, ~~DAIDS will notify~~ CONRAD ~~who~~ will notify the US FDA and the site investigators of record will notify the responsible IRBs expeditiously.

Section 8.4, Expedited Adverse Event Reporting Requirements:

Expedited Adverse Event (EAE) Reporting

The adverse events that must be reported in an expedited fashion **to the DAIDS Regulatory Compliance Center (RCC) Safety Office via DAIDS Adverse Events Reporting System (DAERS)** include all serious adverse events (SAEs) as defined by the May 1996 International Conference on Harmonisation of Technical Requirements for Registration of Pharmaceuticals for Human Use (ICH), Good Clinical Practice: Consolidated Guidance (E6) regardless of relationship to the study agent(s). Important medical events that may not be immediately life-threatening or result in death or hospitalization but may jeopardize the patient or may require intervention to prevent one of the outcomes listed in the definition above may also be considered to be serious.[83]

All sites will report all SAEs expeditiously to RCC via the electronic reporting system DAERS established by DAIDS. For those reports related to the Gilead study product (i.e. oral tablets), the RCC Safety Office will send the unprocessed reports directly to Gilead. However, the RCC Office will also prepare the draft safety reports for all study products and send them to the DAIDS Medical Officer and CONRAD MOs for review.

Study sites will be contacted by DAIDS MO if any further information or clarification is needed after the report is evaluated by DAIDS and CONRAD MOs. The RCC office will then prepare the final report which will go to CONRAD for signature and submission to the FDA. Copies of this final report will be filed with Gilead, CONRAD, and the RCC. Additionally, the RCC Safety Office will distribute the final safety reports to all DAIDS funded networks/sites evaluating the study products used in this study.

For all SAEs submitted, sites must file an ~~initial and an~~ **RCC** update ~~to CONRAD and the DAIDS Medical Officer~~ with the final or stable outcome unless the initial EAE submitted had a final or stable outcome ~~noted~~ already **noted**.

EAE Reporting Requirements for this Study

~~Any adverse event that is determined to be serious (whether expected or unexpected) regardless of relationship to the study agent(s) must be immediately reported to CONRAD and the DAIDS Medical Officer (21 CFR 312.64). An Expedited Adverse Event (EAE) Form must be completed and sent to CONRAD and the DAIDS Medical Officer within 3 business days (by 5 PM Eastern Time (ET)) after site awareness that the event has occurred at a reportable level. DAIDS MO will review and discuss the EAE report with CONRAD to address any concerns.~~

~~CONRAD will then notify the FDA of any unexpected serious adverse events associated with the use of the drug as soon as possible, but no later than 7 calendar days after initial receipt of the information from the investigator.~~

~~For unexpected serious adverse events associated with the use of the drug, CONRAD will submit the safety reports provided by the sites to the IND no later than 15 calendar days after the initial receipt of the information and send copies of the submission to the DAIDS MO, the RCC (to be placed in the file) and Gilead.~~ **Sites using the DAERS internet-based reporting system for submission of EAEs to DAIDS will follow the DAERS processes as outlined in the DAERS training information. For questions about DAERS, please contact DAIDS-ES at DAIDS-ESSupport@niaid.nih.gov or from within the DAERS application itself.**

If the site cannot use DAERS to report an AE on an expedited basis, the AE must be documented on the DAIDS Expedited Adverse Event Reporting Form (EAE Reporting Form) available on the RCC website: http://rcc.tech-res-intl.com (and MOP if applicable), and submitted as specified by the DAIDS EAE Manual. For questions about EAE reporting, please continue to contact the RCC. DAIDS EAE forms should be submitted to DAIDS through the RCC Safety Office (rccsafetyoffice@tech-res.com) or call 1-800-537-9979 or 301-897-1709 or fax 1-800-275-7619 or 301-897-1710.]

Section 8.5, Pregnancy and Pregnancy Outcomes, last paragraph:

In addition, the U19 MDP Core C – Data Management and Biostatistics Core – will file a quarterly report of all pregnancies with CONRAD and Gilead, as well as an annual summary report of all adverse events for the annual IND reports (to be submitted by CONRAD).

2. **The protocol is updated to include an additional rectal microflora swab at Visit 11 per a request from the FDA:**

Table 18: Visit 11 (Study Product Visit #3: Dispense Self-Administered Gel Supply):

**Rectal Specimens**	• **Anorectal swabs**<br>o **Rectal microflora**<br>• Rectal Sponges<br>o Rectal tenofovir concentrations ~~(dry sponge)~~

Section 2.11, Justification of Sampling Timepoints, Table 9: Schedule of Study Endpoints

Visit 11: Begin 7-day Topical	Blood, fluids from sponges ***					✓						✓	✓	✓		

Appendix I: Schedule of Study Visits and Evaluations:

Rectal microflora			X				X				**X**	X	X		

3. **Section 5.3, Exclusion Criteria, item 3i is modified. This statement is removed as these labs are not being performed at the Screening Visit:**
   i. History of bleeding problems ~~(i.e. INR > 1.5× the site laboratory ULN or PTT > 1.25× the site laboratory ULN)~~

4. **Excessive detail regarding sponge types (i.e., dry vs. pre-moistened) has been removed from Sections 7.2, 7.3, 7.4, and 7.5 of the protocol.**

5. **The Sample Informed Consent Document (Enrollment) is modified to achieve consistency with the protocol:**

What Do I Have To Do If I Am In This Study? section:

Third paragraph:

Study visits will take about 45 minutes and **can** last up to ~~three~~ **five** hours.

Fourth paragraph, first bullet:

~~Have samples of fluid from your rectum taken to test for gonorrhea and chlamydia~~

Schedule of Study Visits and Evaluations (from Sample Informed Consent Document (Enrollment):

Pregnancy test	X	X	**X**				**X**				X	**X**			▲

Syphilis test	X											**X**			▲

6. **In addition to the changes listed above, the following minor clarifications and corrections have been made to the protocol:**

Section 2.11 Justification of Sampling Time Points, third paragraph, first sentence:

PK time points will consist of a 4-point extracellular and intracellular plasma and rectal secretion sampling strategy (for women, cervicovaginal secretions will also be collected) within the first 24 hours of oral and single and multiple dose topical exposure for all subjects (Visit 3, Visit 7, and Visit 1~~1~~**2**).

All references to urinalysis are corrected to reflect inclusion of testing for nitrites, not nitrates:

Section 7.1, Screening Visit, Table 13: Visit 1 (Screening Visit):

**Urine**	• Collect urine sample<br>o ♀Qualitative hCG<br>o Dipstick Urinalysis (UA) for protein, glucose, nitr~~a~~**i**tes, and leukocyte esterase<br>o Nucleic acid amplification test (NAAT) for GC/CT

Section 7.12.1, Local Laboratory Testing, Clinical: Safety Urine Samples, second sentence:

These will include routine urinalysis (protein, glucose, nitr~~a~~**i**tes, and leukocyte esterase), NAAT for GC/CT as well as pregnancy tests for female participants.

Section 13.9.2, Care for Participants Identified as HIV-Infected, second sentence is updated to reflect that both HIV-infected men and women will be referred to appropriate sources of care:

According to site SOPs, study staff will refer participants found to be HIV-infected to available sources of medical and psychological care, social support, and local research studies for HIV-infected ~~women~~ **adults**.

Appendix 1: Schedule of Study Visits and Evaluations:

Test results		X	X				X				X	X			
Schedule next study visit	▲	X	X	X	X	X	X	X	X	X	X	X	~~X~~	▲	▲
CBC w/ diff and platelets	X	**X**	X				X					X			
Vaginal sponge ~~(dry)~~		X	X	X	X	X	X	X	X	X	X	X	X		
TFV levels in rectal mucosal tissue homogenates		**X**	X		X	X	X		X	X		X			
Rectal tenofovir concentrations		~~X~~	X	X	X	X	X	X	X	X	X	X	X		

The above information will be incorporated into the next version of the protocol at a later time if it is amended.

Microbicide Trials Network
CLARIFICATION MEMO #01 TO:

RMP-02/MTN-006
A two-site, Phase 1, partially-blinded, placebo-controlled safety, acceptability, and pharmacokinetic trial of topical, vaginally-formulated tenofovir 1% gel applied rectally compared with oral 300 mg tenofovir disoproxil fumarate in HIV-1 seronegative adults

Version 1.0/07 April 2009
DAIDS Document ID #10769
CONRAD IND # 73, 382

Date of Clarification Memorandum: 24 April 2009

Section 1: Summary of Clarifications and Rationale

The items clarified in this Clarification Memorandum (CM) have been approved by the NIAID Medical Officer and are to be implemented immediately upon issuance. IRB approval of this CM is not required by the sponsor; however, investigators may submit the CM to the IRB overseeing the study at their site for information. This CM is official RMP-02/MTN-006 documentation and is effective immediately. A copy of this CM must be retained in each study site's Essential Documents file for RMP-02/MTN-006. No change in informed consent is necessitated by or included in this CM.

The primary goal for this CM is to indicate Ross Cranston as the Site Investigator for the Pitt CRS.

Section 2: Implementation

With the exception of the modifications to the Protocol Team Roster, text to be deleted is noted by ~~strikethrough~~ and text to be added is noted below in **bold**.

1. The protocol is updated to reflect Ross Cranston as the Site Investigator for the Pitt CRS:

The Protocol Team Roster is edited as follows:

Ross D. Cranston, MD FRCP
Site Investigator
Division of Infectious Disease
University of Pittsburgh Medical Center
Falk Medical Building, Suite 611
3601 Fifth Avenue
Pittsburgh, PA 15213 USA
Phone: 412-647-4007
Fax: 412-647-5519
Email: rdc27@pitt.edu

Section 1.4, Site Investigators, is updated as follows:

Site Investigator: ~~Ian McGowan, MD, PhD, FRCP~~ **Ross Cranston, MD FRCP**

The above information will be incorporated into the next version of the protocol at a later time if it is amended.

RMP-02/MTN-006

A two-site, Phase 1, partially-blinded, placebo-controlled safety, acceptability and pharmacokinetic trial of topical, vaginally-formulated tenofovir 1% gel applied rectally compared with oral 300 mg tenofovir disoproxil fumarate in HIV-1 seronegative adults

**A Collaboration Between the NIH Integrated Pre-Clinical/Clinical Program (IPCP)
U19 Microbicide Development Program's (MDP's)
Rectal Microbicide Program (RMP), CONRAD, and
the Microbicide Trials Network (MTN)**

<u>Short Title</u>: Phase 1 rectal microbicide safety and acceptability trial of topically applied tenofovir compared with oral tablet

**Funded by:
Division of AIDS, US National Institute of Allergy and Infectious Diseases
US National Institutes of Health**

**Grant #:
5-U19-AI 060614
5-U01-AI068633-03**

DAIDS Protocol #10769

**IND Holder:
CONRAD**

**Additional pharmaceutical product supplied by:
Gilead Sciences, Inc.**

This protocol will be performed under CONRAD IND# 73, 382

**Protocol Chair:
Peter Anton, MD**

Version 1.0

7 April 2009

RMP-02/MTN-006

A two-site, Phase 1, partially-blinded, placebo-controlled safety, acceptability, and pharmacokinetic trial of topical, vaginally-formulated tenofovir 1% gel applied rectally compared with oral 300 mg tenofovir disoproxil fumarate in HIV-1 seronegative adults

TABLE OF CONTENTS

LIST OF ABBREVIATIONS AND ACRONYMS ... v
PROTOCOL TEAM ROSTER ... viii
INVESTIGATOR SIGNATURE FORM ... xii
PROTOCOL SUMMARY ... xiii
1 KEY ROLES ... 16
1.1 Protocol Identification ... 16
1.2 Sponsor and Monitor Identification ... 16
1.3 Medical Officer ... 16
1.4 Site Investigators ... 16
1.5 Clinical Laboratories ... 17
1.6 Data Center ... 17
1.7 Study Operations ... 17
2 INTRODUCTION ... 18
2.1 Background of Microbicide Research ... 18
2.2 Description of Study Products ... 22
2.3 TDF (Tenofovir tablet) ... 22
2.4 Tenofovir 1% Gel (Tenofovir Gel) ... 23
2.5 HEC Placebo Gel ... 23
2.6 *In Vitro* Studies ... 24
2.7 Animal Studies ... 27
2.8 Human Clinical Studies ... 38
2.9 Study Hypotheses and Rationale ... 44
2.10 Justification of Dosing ... 47
2.11 Justification of Sampling Time Points ... 48
3 OBJECTIVES ... 51
3.1 Primary Objective: Safety ... 51
3.2 Secondary Objectives: Immunotoxicity, Pharmacokinetics, and Acceptability 51
3.3 Exploratory Objectives: ... 51
4 STUDY DESIGN ... 51
4.1 Identification of Study Design ... 51
4.2 Summary of Major Endpoints ... 51
4.3 Description of Study Population ... 53
4.4 Time to Complete Enrollment ... 53
4.5 Study Groups ... 53
4.6 Sequence and Duration of Trial Periods ... 53

4.7 Expected Duration of Participation ... 53
4.8 Sites ... 54
5 STUDY POPULATION ... 54
5.1 Selection of the Study Population ... 54
5.2 Inclusion Criteria ... 55
5.3 Exclusion Criteria ... 56
6 STUDY PRODUCT ... 58
6.1 Regimen ... 58
6.2 Administration ... 58
6.3 Study Product Formulation ... 59
6.4 Study Product Supply and Accountability ... 60
6.5 Assessment of Participant Adherence ... 61
6.6 Concomitant Medications ... 62
7 STUDY PROCEDURES ... 62
7.1 Screening Visit ... 65
7.2 Enrollment/Baseline Evaluation Visit ... 67
7.3 Follow-up Visits ... 68
7.4 Product Use End Visit ... 71
7.5 Final Clinic Visit ... 73
7.6 Follow-up Safety Phone Call ... 73
7.7 Follow up Procedures for Participants Who Discontinue Study Product ... 73
7.8 Interim Contacts and Visits ... 75
7.9 Clinical Evaluations and Procedures ... 76
7.10 Behavioral Measures ... 77
7.11 Pharmacokinetic Procedures ... 78
7.12 Laboratory Evaluations ... 80
7.13 Specimen Collection and Processing ... 83
7.14 Specimen Handling ... 83
7.15 Storage of Specimens for Future Use ... 83
7.16 Biohazard Containment ... 84
8 ASSESSMENT OF SAFETY ... 84
8.1 Safety Monitoring ... 84
8.2 Clinical Data Safety Review ... 84
8.3 Adverse Events Definitions and Reporting Requirements ... 85
8.4 Expedited Adverse Event Reporting Requirements ... 88
8.5 Pregnancy and Pregnancy Outcomes ... 89
8.6 Social Harms Reporting ... 89
9 CLINICAL MANAGEMENT ... 90
9.1 Grading System ... 90
9.2 Discontinuation of Study Product(s) in the Presence of Toxicity ... 90
9.3 General Criteria for Discontinuation of Study Product ... 91
9.4 Management of Specific Toxicities ... 92
9.5 Clinical Management of Pregnancy ... 92
9.6 Criteria for Early Termination of Study Participation ... 93
10 STATISTICAL CONSIDERATIONS ... 93
10.1 Overview and General Design ... 93

10.2 Study Endpoints 93
10.3 Study Hypotheses 94
10.4 Sample Size 95
10.5 Randomization Procedures 95
10.6 Participant Accrual, Follow-up, Retention, and Replacement 96
10.7 Data Monitoring and Analysis 97
11 DATA HANDLING AND RECORDKEEPING 105
11.1 Data Management Responsibilities 105
11.2 Source Documents and Access to Source Data/Documents 105
11.3 Quality Control and Quality Assurance 106
11.4 Study Coordination 106
12 CLINICAL SITE MONITORING 106
13 HUMAN SUBJECTS PROTECTIONS 107
13.1 Institutional Review Boards 107
13.2 Protocol Registration 107
13.3 Risk Benefit Statement 108
13.4 Informed Consent Process 111
13.5 Participant Confidentiality 112
13.6 Special Populations 112
13.7 Incentives 113
13.8 Communicable Disease Reporting 113
13.9 Access to HIV-related Care 114
13.10 Study Discontinuation 114
14 PUBLICATION POLICY 114
15 APPENDICES 115
APPENDIX I: SCHEDULE OF STUDY VISITS AND EVALUATIONS 116
APPENDIX II: HIV ANTIBODY TESTING ALGORITHMS 117
APPENDIX III: TOXICITY TABLES 118
APPENDIX IV: MANUAL FOR EXPEDITED REPORTING OF ADVERSE EVENTS TO DAIDS 119
APPENDIX V: RECTAL MICROFLORA 120
APPENDIX VI: HISTOPATHOLOGY SCORING SYSTEM 121
APPENDIX VII: SAMPLE INFORMED CONSENT FORM (SCREENING) 122
APPENDIX VIII: SAMPLE INFORMED CONSENT DOCUMENT (ENROLLMENT) 129
APPENDIX IX: SAMPLE INFORMED CONSENT (STORAGE AND FUTURE TESTING OF SPECIMENS) 142
APPENDIX X: SAMPLE PRODUCT USE LOG DAILY DIARY 146
REFERENCES 147

TABLES

Table 1: Summary of RAI in Surveys of Sexual Behavior 18
Table 2: Tenofovir Bioassay Data 30
Table 3: Rectal Irritation Study 32
Table 4: Use of Topical Tenofovir Gel to Prevent Vaginal Transmission of SIV 34
Table 5: Other Studies of Tenofovir 1% Gel 42

Table 6: PrEP Studies ... 42
Table 7: Range of Human Exposure With Tenofovir 1% Gel[81] ... 48
Table 8: Use and Distribution of Biopsies in 2-Week Follow-Up After Single Topical and Oral Exposure ... 49
Table 10: Summary of Biological Samples Collected In Study ... 52
Table 11: Study Regimen ... 58
Table 12: Study Visit Windows ... 65
Table 13: Visit 1 (Screening Visit) ... 66
Table 14: Visit 2 (Enrollment/Baseline Evaluation Visit-All Participants) ... 67
Table 15: Visit 3 and Visit 7 (Study Product #1; Study Product #2 and 30' sampling for each) ... 68
Table 16: Visits 4, 8, and 13(24-Hour Post-Exposure PK Visit: All Participants) ... 69
Table 17: Visits 5 and 6 and Visits 9 and 10 (2-Week Sampling Period) ... 70
Table 18: Visit 11 (Study Product Visit #3: Dispense Self-Administered Gel Supply) ... 71
Table 19: Visit 12 (Sampling Visit Following Once Daily Exposure for 7 Days (7th dose given in clinic)) ... 72
Table 20: Visit 13 Final Clinic/Early Termination Visit ... 73
Table 21: Follow-up Safety Phone Call ... 73
Table 22: Interim Contacts and Visits ... 76
Table 23: Probability of Events, Selected True Rates of AEs (n=18, oral study) ... 98
Table 24: Probability of Events, Selected True Rates of AEs (n=12, topical study) ... 99
Table 25: Exact 95% Confidence Bounds for True Rates of AEs (n=18, oral study) ... 99
Table 26: Exact 95% Confidence Bounds For True Rate of AEs (n=12, topical study) ... 99

RMP-02/MTN-006
A two-site, Phase 1, partially-blinded, placebo-controlled safety, acceptability, and pharmacokinetic trial of topical, vaginally-formulated tenofovir 1% gel applied rectally compared with oral 300 mg tenofovir disoproxil fumarate in HIV-1 seronegative adults

LIST OF ABBREVIATIONS AND ACRONYMS

3TC	lamivudine
ABC	abacavir
AE	Adverse Event
AIDS	Acquired Immunodeficiency Syndrome
ALT	alanine transaminase
ANCOVA	analysis of covariance
ANOVA	analysis of variance
APV	amprenavir
AST	aspartate aminotransferase
AUC	area under the curve
BMD	bone mineral density
BV	bacterial vaginosis
CAPRISA	Centre for the AIDS Programme of Research in South Africa
CBC	complete blood count
CCR5	chemokine receptor 5
CDC	Centers for Disease Control and Prevention
CFR	Code of Federal Regulations
cGMP	current good manufacturing practices
CLIA	Clinical Laboratory Improvement Amendments
C_{max}	maximum serum concentration
CORE	Coordinating and Operations Center
C-PMPA	radiolabeled tenofovir
CRF	case report form
CRS	clinical research site
CT	*Chlamydia trachomatis*
CVL	cervicovaginal lavage
CXCR4	CXC chemokine receptor 4
d4T	stavudine
DAIDS	Division of AIDS
ddC	zalcitabine
ddI	didanosine
DLV	delavirdine
DMPA	depot medroxyprogesterone acetate
DP	diphosphate
DSMB	Data and Safety Monitoring Board
EFV	efavirenz
ELISA	Enzyme-Linked Immunosorbent Assay
EAE	Expedited Adverse Event

ET	Eastern Time
FDA	Food and Drug Administration
FTC	emtricitabine
GC	*Gonorrhoeae*
GCP	Good Clinical Practices
GLP	Good Laboratory Practices
HEC	hydroxyethylcellulose
HHS	Health and Human Services
HIV	Human Immunodeficiency Virus
HPTN	HIV Prevention Trials Network
HSV	Herpes Simplex Virus
IATA	International Air Transport Association
IDV	indinavir
IL-2	interleukin-2
IND	investigational new drug
IPCP	Integrated Pre-Clinical/Clinical Program
IRB	Institutional Review Board
IGF	insulin-like growth factor
IUD	intrauterine device
IV	intravenous
LL	Local Laboratory
MDP	Microbicides Development Program
MID	median rectal infectious doses
MMC	Mucosal Mononuclear Cells
MRM	multiple reaction monitoring
mRNA	messenger ribonucleic acid
MO	Medical Officer (Division of AIDS)
MQAP-NICHD	Microbicide Quality Assurance Program-National Institute of Child Health and Human Development
MSM	Men who have Sex with Men
MTN	Microbicide Trials Network
MTT	[1-(4,5-dimethylthiazol-2-yl)-3,5-diphenylformazan]
MWRI F	Magee-Womens Research Institute and Foundation
N-9	Nonoxynol-9
NAAT	Nucleic acid amplification test
NFV	nelfinavir
NIAID	National Institute of Allergy and Infectious Diseases
NICHD	National Institute of Child Health and Human Development
NIH	(United States) National Institutes of Health
NL	network laboratory
NOAEL	no observed adverse effect level
NNRTI	non-nucleoside reverse transcriptase inhibitor
NRTI	nucleoside reverse transcriptase inhibitor
NVP	nevirapine
O.D.	optical density
OHRP	Office for Human Research Protections

PBMC	Peripheral blood mononuclear cells
PCR	Polymerase Chain Reaction
PEP	post-exposure prophylaxis
PK	pharmacokinetic(s)
PMPA	9-[(R)-2-(phosphonomethoxy)propyl] adenine monohydrate
PoR	pharmacist of record
PSRT	Protocol Safety Review Team
PSS	polystyrene sulfonate
PTID	Participant Identification Number
PRN	As needed
QD	Daily
RANTES	Regulated on activation normal T cell expressed and secreted
RCC	Regulatory Compliance Center
RAI	Receptive Anal Intercourse (refers to coitus only, does not include manual stimulation or the use of sex toys or purgatives)
RMP	Rectal Microbicide Program
RPR	rapid plasma reagin
RT	Reverse transcriptase
RTV	ritonavir
SAE	Serious Adverse Experience
SADR	Suspected Adverse Drug Reaction
SHIV	simian/human immunodeficiency virus
SIV	Simian Immunodeficiency Virus
SMC	Safety Monitoring Committee
SOP	standard operating procedure
SQV	saquinavir
SSP	Study-specific procedures
STD/ STI	Sexually Transmitted Disease/ Infection
TDF	tenofovir disoproxil fumarate (oral tenofovir)
TER	transepithelial resistance
TERIS	Teratogen Information System
UA	urinalysis
UAI	Unprotected Anal Intercourse
UCLA	University of California, Los Angeles
ULN	upper limit of normal
U Pitt	University of Pittsburgh
UTI	urinary tract infection
VI	virus isolation
VM	vaginal microbicide
ZDV	zidovudine

RMP-02/MTN-006

A two-site, Phase 1, partially-blinded, placebo-controlled safety, acceptability, and pharmacokinetic trial of topical, vaginally-formulated tenofovir 1% gel applied rectally compared with oral 300 mg tenofovir disoproxil fumarate in HIV-1 seronegative adults

PROTOCOL TEAM ROSTER

Peter Anton, MD
MDP Program Director
Protocol Chair
UCLA Center for Prevention Research
10940 Wilshire Blvd., Suite 1250
Los Angeles, CA 90024 USA
Phone: 310-206-5797
Fax: 310-206-8824
Email: PAnton@mednet.ucla.edu

Alex Carballo-Diéguez, PhD
MTN Behavioral Research Committee Representative
HIV Center for Clinical and Behavioral Studies
New York State Psychiatric Institute and Columbia University
1051 Riverside Drive, Unit 15,
New York, NY 10032 USA
Phone: 212-543-5261
Fax: 212-543-6003
Email: ac72@columbia.edu

Roberta Black, PhD
Microbicide Research Branch Chief
Division of AIDS, NIAID
6700 B Rockledge Drive, Room 5135
Bethesda, MD 20817 USA
Phone: 301-496-8199
Fax: 301-402-3684
Email: rblack@niaid.nih.gov

Grace Chow, MS
Biologist
Prevention Research Branch
Division of AIDS
6700 B Rockledge Drive, Room 5119
Bethesda, MD 20892-7628 USA
Phone: 301-435-3747
Fax: 301-402-3684
E-Mail: GChow@niaid.nih.gov

Katherine Bunge, MD
MTN Protocol Safety Physician
Magee-Womens Hospital of UPMC
300 Halket Street
Pittsburgh, PA 15213 USA
Phone: 412-917-9936
Fax: 412-641-6170
Email: kbunge@mail.magee.edu

Richard Clark, MPH
Gilead Sciences, Inc.
333 Lakeside Drive
Foster City, CA 94404 USA
Phone: 650-522-1626
Fax: 650-522-5595
Email: rclark@gilead.com

Marianne Morse Callahan
Deputy Director, Clinical
CONRAD
1911 North Fort Myer Drive, Suite 900
Arlington, VA 22209
Phone: 703-276-3915
Fax: 703-524-4770
Email: mcallahan@conrad.org

Nancy Connolly, MD
MTN Safety Physician
Microbicide Trials Network
7006 43rd Ave., NE
Seattle, WA 98115 USA
Phone: 206 523 1177
Fax: 412-641-6170
Email: nancycsc@gmail.com

RMP-02/MTN-006

A two-site, Phase 1, partially-blinded, placebo-controlled safety, acceptability, and pharmacokinetic trial of topical, vaginally-formulated tenofovir 1% gel applied rectally compared with oral 300 mg tenofovir disoproxil fumarate in HIV-1 seronegative adults

PROTOCOL TEAM ROSTER

Ross D. Cranston, MD FRCP
MTN Safety Physician
Division of Infectious Disease
University of Pittsburgh Medical Center
Falk Medical Building, Suite 611
3601 Fifth Avenue
Pittsburgh, PA 15213 USA
Phone: 412-647-4007
Fax: 412-647-5519
Email: rdc27@pitt.edu

William Cumberland, PhD
Biostatistician
Professor & Chair, Department of Biostatistics
UCLA School of Pubic Health
CHS 51-236B
675 Charles E Young Dr. South
Los Angeles, CA 90095
Phone: 310-206-9621
Fax: 310-267-2113
Email: wgc@ucla.edu

Charlene S. Dezzutti, PhD
MTN Network Laboratory Director
Microbicide Trials Network
204 Craft Avenue
Pittsburgh, PA 15213 USA
Phone: 412-641-3462
Fax: 412-641-6170
Email: dezzuttics@upmc.edu

David R. Friend, PhD
Director, Product Development
CONRAD
1911 N. Fort Myer Drive, Suite 900
Arlington, VA 22209 USA
Phone: 703-276-3906
Fax: 703-524-4770
Email: dfriend@conrad.org

Craig W. Hendrix, M.D.
Laboratory Director and Consultant
Division of Clinical Pharmacology
600 North Wolfe Street
Harvey 502
Baltimore, MD 21287-5554 USA
Phone: 410-955-9707
Fax: 410-955-9708
Email: cwhendrix@jhmi.edu

Sharon Hillier, PhD
MTN Principal Investigator
Microbicide Trials Network
204 Craft Avenue
Pittsburgh, PA 15213 USA
Phone: 412-641-8933
Fax: 412-641-6170
Email: shillier@mail.magee.edu

Cindy Jacobson, PharmD
MTN Director of Pharmacy Affairs
Microbicide Trials Network
204 Craft Avenue
Pittsburgh, PA 15213 USA
Phone: 412-641-8913
Fax: 412-641-6170
Email: cjacobson@mail.magee.edu

Angela D.M. Kashuba, BScPhm, PharmD, DABCP
Consultant/Data Analyst
Director, UNC CFAR Clinical Pharmacology Core
School of Pharmacy, CB #7360
University of North Carolina
Chapel Hill, NC 27599-7360 USA
Phone: 919-966-9998
Fax: 919-962-0644
Email: akashuba@unc.edu

RMP-02/MTN-006

A two-site, Phase 1, partially-blinded, placebo-controlled safety, acceptability, and pharmacokinetic trial of topical, vaginally-formulated tenofovir 1% gel applied rectally compared with oral 300 mg tenofovir disoproxil fumarate in HIV-1 seronegative adults

PROTOCOL TEAM ROSTER

Elena Khanukhova
Clinical Research Manager
MDP Regulatory Core
UCLA Center for Prevention Research
10940 Wilshire Blvd., Suite 1250
Los Angeles, CA 90024 USA
Phone: 310-206-3633
Fax: 310-794-2829
Email: ekhanukhova@mednet.ucla.edu

Lorna Rabe, BS, M(ASCP)
MTN Laboratory Manager
Microbicide Trials Network
204 Craft Avenue Room A530
Pittsburgh PA 15213 USA
Phone: 412-641-6042
Fax: 412-641-6170
Email: rabelk@upmc.edu

Ian McGowan, MD, PhD, FRCP
MTN Co-Principal Investigator
University of Pittsburgh School of Medicine
Magee-Women's Research Institute
204 Craft Avenue
Room B505
Pittsburgh, PA 15213 USA
Phone: 412-641-4710
Fax: 412-641-6170
Email: mcgowanim@mail.magee.edu

James Rooney, MD
Co-Sponsor Medical Monitor
Gilead Sciences, Inc.
333 Lakeside Drive
Foster City, CA 94404 USA
Phone: 650-522-5708
Fax: 650-522-5854
Email: Jim_Rooney@gilead.com

Lisa Noguchi, CNM, MSN
Director of Operations
Microbicide Trials Network
204 Craft Avenue
Pittsburgh, PA 15213 USA
Phone: 412-641-8945
Fax: 412-641-6170
Email: lnoguchi@mail.magee.edu

Janet Schafer
Manager, Clinical Trials/ Regulatory Affairs
CONRAD
1911 Fort Myer Drive
Suite 900
Arlington, VA 22209 USA
Phone: 703-276-4034
Fax: 703-524-4770
Email: jschafer@conrad.org

Jeanna Piper, MD
DAIDS Medical Officer
NIAID
Division of AIDS
6700 B Rockledge Drive, Room 5124
Bethesda, MD 20892 USA
Phone: 301-451-2778
Fax: 301-402-3684
Email: piperj@niaid.nih.gov

Jill Schwartz, MD
Medical Director
CONRAD
1911 Fort Myer Drive
Suite 900
Arlington, VA 22209 USA
Phone: 703-276-3913
Fax: 703-524-4770
Email: jschwartz@conrad.org

Mala Shah, MPH
Protocol Development Manager
Microbicide Trials Network
204 Craft Avenue
Pittsburgh, PA 15213 USA
Phone: 412-641-8999
Fax: 412-641-6170
Email: shahms@mail.magee.edu

Jim A. Turpin, PhD
DAIDS Program Officer
Microbicide Research Branch
Division of AIDS
6700 B Rockledge Drive, Room 4118
Bethesda, MD 2089 USA
Phone: 301-451-2732
Fax: 301-496-8530
Email: JTurpin@niaid.nih.gov

Ana Ventuneac, PhD
HIV Center for Clinical and Behavioral Studies
New York State Psychiatric Institute and
Columbia University
1051 Riverside Drive, Unit 15,
New York, NY 10032 USA
Phone: 212-568-4352
Fax: 212-543-4385
Email: ventune@pi.cpmc.columbia.edu

RMP-02/MTN-006

A two-site, Phase 1, partially-blinded, placebo-controlled safety, acceptability, and pharmacokinetic trial of topical, vaginally-formulated tenofovir 1% gel applied rectally compared with oral 300 mg tenofovir disoproxil fumarate in HIV-1 seronegative adults

INVESTIGATOR SIGNATURE FORM

Version 1.0
7 April 2009

A Collaboration Between the NIH Integrated Pre-Clinical/Clinical Program (IPCP) U19 Microbicide Development Program's (MDP's) Rectal Microbicide Program (RMP) and the Microbicide Trials Network (MTN)

Funded by:
Division of AIDS, US National Institute of Allergy and Infectious Diseases
US National Institutes of Health

IND Holder:
CONRAD

Additional pharmaceutical product supplied by:
Gilead Sciences, Inc.

I, the Investigator of Record, agree to conduct this study in full accordance with the provisions of this protocol. I will comply with all requirements regarding the obligations of investigators as outlined in the Statement of Investigator (Form FDA 1572), which I have also signed. I agree to maintain all study documentation for at least two years following the date of marketing approval for the study gel and/or tablets for the indication in which it was/they were studied. If no marketing application is filed, or if the application is not approved, the records will be retained for two years after the investigation is discontinued and the US Food and Drug Administration is notified. Publication of the results of this study will be governed by MDP/RMP and MTN policies. Any presentation, abstract, or manuscript will be submitted to the MDP/RMP Scientific Review Committee and the MTN Manuscript Review Committee, DAIDS, CONRAD, and Gilead Sciences, Inc. for review prior to submission.

I have read and understand the information in the Investigator's Brochure(s), including the potential risks and side effects of the products under investigation, and will ensure that all associates, colleagues, and employees assisting in the conduct of the study are informed about the obligations incurred by their contribution to the study.

____
Name of Investigator of Record

______________________________ ______________________________
Signature of Investigator of Record Date

RMP-02/MTN-006

A two-site, Phase 1, partially-blinded, placebo-controlled safety, acceptability, and pharmacokinetic trial of topical, vaginally-formulated tenofovir 1% gel applied rectally compared with oral 300 mg tenofovir disoproxil fumarate in HIV-1 seronegative adults

PROTOCOL SUMMARY

Short Title: Phase 1 rectal microbicide safety and acceptability trial of topically applied tenofovir compared with oral tablet

Clinical Phase: Phase 1

IND Sponsor: CONRAD

Protocol Chair: Peter Anton, MD

Sample Size: Approximately 18 evaluable participants

Study Population: Sexually-abstinent HIV-negative men and women

Participating Clinical Research Sites (CRS):

- University of California at Los Angeles, Los Angeles, USA
- Pitt CRS, Pittsburgh, USA

Study Design: Phase 1, randomized, two-site, partially-blinded, placebo controlled study

Study Duration: Approximately 3.5 months per participant, with projected 8.5 calendar months for accrual

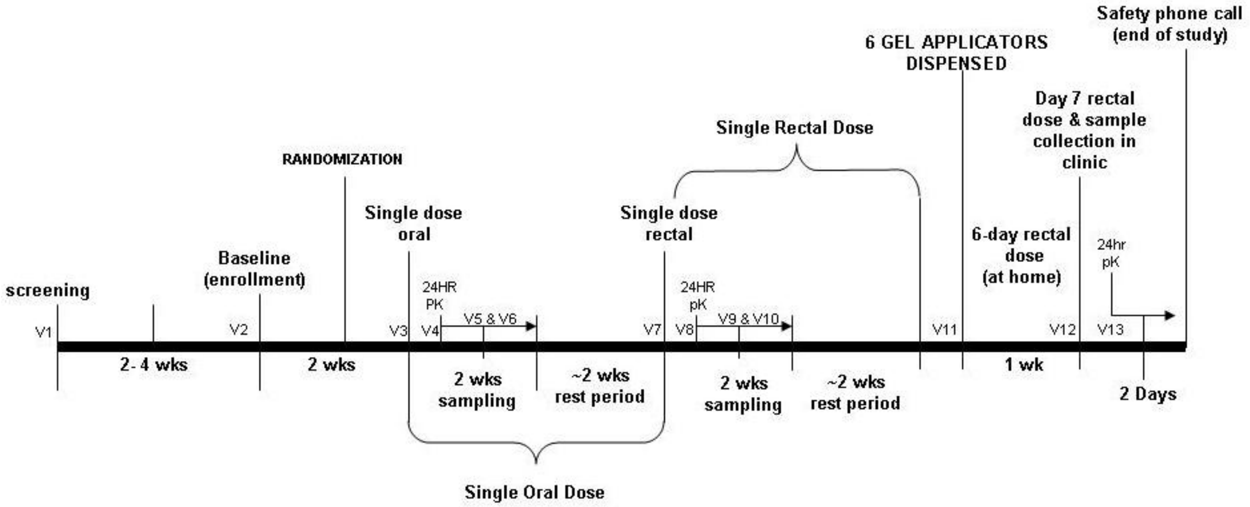

Study Products:

Oral
- Tenofovir Disoproxil Fumarate (TDF) 300mg tablet

Rectal
- Tenofovir 1% gel
- Hydroxyethyl cellulose (HEC) placebo gel

Study Regimen:

	Single Oral Dose	2 WK Sampling Period	2 WK Resting Period (+ 7 days)	Single Rectally-Applied Dose	2 WK Sampling Period	2 WK Resting Period (+ 7 days)	7-day Exposure Dose
**Group A**	Oral TDF	Day 0 (30 min), Day 1 and Day 7		Tenofovir 1% Gel or Placebo Gel	Day 0 (30 min), Day 1 and Day 7		Participants to receive same product as administered during single rectally applied dose
**Group B**	Oral TDF	Day 0 (30 min), Day 4 and Day 10		Tenofovir 1% Gel or Placebo Gel	Day 0 (30 min), Day 4 and Day 10		Participants to receive same product as administered during single rectally applied dose

Primary Objective: Safety

- To evaluate the systemic safety of 1% vaginally-formulated tenofovir gel, applied rectally

Primary Endpoints:

- Grade 2 or higher clinical and laboratory adverse events as defined by the Division of AIDS Table for Grading the Severity of Adult and Pediatric Adverse Events, Version 1.0, Dec 2004 and Addenda 1 and 3 (Female Genital and Rectal Grading Tables for Use in Microbicide Studies) to this table.

Secondary Objectives: Immunotoxicity, Pharmacokinetics, and Acceptability

- To evaluate the immunotoxicity of 1% vaginally formulated tenofovir gel applied rectally
- To compare systemic and compartment pharmacokinetics (PK) among oral TDF, rectally applied tenofovir 1% gel, and placebo gel
- To evaluate the acceptability of tenofovir 1% gel when applied rectally

Secondary Endpoints:

- Fecal calprotectin
- Microflora (see Appendix V)
- Rectal cytokines (secreted)
- Rectal epithelial sloughing
- Rectal histology
- Rectal CD4 cell phenotype/activation
- Tenofovir concentrations
    - o Plasma (tenofovir)
    - o PBMC (intracellular)
    - o Rectal fluid
    - o Vaginal fluid
    - o Rectal mucosal tissue homogenates
    - o Rectal mucosal mononuclear cells (MMC)
- Tenofovir diphosphate concentrations
  - o PBMC
  - o Rectal mucosal tissue homogenates
  - o Rectal mucosal mononuclear cells

- The proportion of participants who at their Final Clinic Visit (Visit 13) report that they would be very likely to use the candidate microbicide during receptive anal intercourse

Exploratory Objectives:

- To assess the preliminary (*ex vivo)* efficacy using biopsy explants of rectally applied tenofovir 1% gel

Exploratory Endpoints:

- Changes in HIV-1 p-24 levels in colorectal explant supernatant

1 KEY ROLES

1.1 Protocol Identification

Protocol Title:	A two-site, Phase 1, partially-blinded, placebo-controlled safety, acceptability, and pharmacokinetic trial of topical, vaginally-formulated tenofovir 1% gel applied rectally compared with oral 300 mg tenofovir disoproxil fumarate in HIV-1 seronegative adults
RMP Protocol Number:	RMP-02
MTN Protocol Number:	MTN-006
Date:	April 7, 2009

1.2 Sponsor and Monitor Identification

Funding Agency:	DAIDS/National Institute of Allergy and Infectious Diseases (NIAID)/National Institutes of Health (NIH)<br>6700 B Rockledge Drive<br>Bethesda, MD 20892 USA
IND Holder:	CONRAD<br>1911 North Fort Myer Drive, Suite 900<br>Arlington, VA 22209 USA
Pharmaceutical Support:	Gilead Sciences, Inc.<br>333 Lakeside Drive<br>Foster City, CA 94404 USA
Monitor:	Pharmaceutical Product Development, Inc. (PPD)<br>929 North Front Street<br>Wilmington, NC 28401-3331 USA

1.3 Medical Officer

**DAIDS Medical Officer:**	Jeanna Piper, MD<br>National Institute of Allergy and Infectious Diseases<br>Division of AIDS<br>6700 B Rockledge Drive, Room 5124<br>Bethesda, MD 20892 USA

1.4 Site Investigators

Site Investigator:	Peter Anton, MD

Site Investigator: Ian McGowan, MD, PhD, FRCP

1.5 Clinical Laboratories

Network Laboratory:	MTN Network Laboratory (NL)<br>Magee-Womens Research Institute<br>204 Craft Avenue<br>Pittsburgh, PA 15213 USA
Pharmacology:	MTN NL Pharmacology Coordinating and Operations Center (CORE)<br>Osler 527<br>600 N. Wolfe St.<br>Johns Hopkins University<br>Baltimore, MD 21287 USA
UCLA Clinical Laboratory:	UCLA Clinical Laboratory<br>200 Medical Plaza, 1st Floor<br>Los Angeles, CA 90095 USA
UCLA MICL Core Laboratory:	675 Charles Young Dr, South<br>MRL Room 1529<br>Los Angeles, CA 90095-7208 USA
Genova Diagnostics:	63 Zillicoa Street<br>Asheville, NC 28801 USA

1.6 Data Center

Data Center:	UCLA Computing Technologies Research Lab<br>1417 Ueberroth Building<br>Los Angeles, CA 90095-7208 USA

1.7 Study Operations

Study Operations:	UCLA Center for Prevention Research<br>10940 Wilshire Blvd., Suite 1250<br>Los Angeles, CA 90024 USA

2 INTRODUCTION

2.1 Background of Microbicide Research

To date, the majority of microbicide research has focused on the assessment of the safety and effectiveness of vaginal microbicides used for the prevention of HIV transmission via the vaginal compartment. Receptive anal intercourse (RAI) is common among men who have sex with men (MSM) and there is increasing evidence that heterosexual women in the developed and developing world also practice anal sex (Table 1). It can therefore be anticipated that once vaginal microbicides are licensed, they will be used in both the vaginal and rectal compartments. As a consequence, there is a need to evaluate both the rectal and vaginal safety profile of candidate microbicides.

Table 1: Summary of RAI in Surveys of Sexual Behavior

Population	Men, Women, or Men and Women	N	Prevalence of AI	Reference
MSM in EXPLORE study	Men	4295	48-54%	Koblin et al. 2003[1]
High risk women	Women	1,268	32%	Gross M et al. 2000[2]
College students	Men and women	210	20%	Civic D 2000[3]
US Survey (15-44 year olds)	Men and women	12, 571	35-40%	Mosher 2005 [4]
Californian residents	Men and women	3,545	6-8%	Erickson PI et al. 1995[5]

The Integrated Preclinical/Clinical Program (IPCP) for HIV topical microbicides within DAIDS at NIH is the primary sponsor of the 5-year parent grant “Microbicide Development Program” (MDP). The MDP award started in 8/04 with the focus of advancing pipeline development of rectal microbicides. The initial efforts have utilized vaginal formulations of various reverse transcriptase (RT)-inhibitor drugs as HIV-1 microbicides as these formulations were already available and in use in vaginal microbicide trials and safety data generated following rectal use would further define the global safety profile of these products. This clinical trial is one of 6 trials being conducted under this Program, and is labeled “RMP-02” for “Rectal Microbicide Program Clinical Trial #2”.

As this trial design is novel and provided the opportunity to investigate many compartmental PK and safety parameters heretofore unknown, as well as expedite the comparison of relative impact of a single topical versus oral dose of tenofovir on these parameters, the NIH IPCP Project Officer and Review Committee supported the inclusion of the topical/oral comparison within this trial. As the Microbicide Trials Network (MTN) was undertaking similar but more diverse efforts, it was proposed to the MTN Executive Committee to consider partnering in the undertaking of this trial. This was agreed upon with mutual benefits being the combined support and submission of the relevant INDs and the use of some extant MTN infrastructure and laboratories.

CONRAD and Gilead are co-sponsors with DAIDS of the trial. This proposal is novel, builds on strengths from both the DAIDS/IPCP programs and MTN's development expertise and portfolio goals. The first-in-kind linkage also benefits and expedites regulatory as well as laboratory consensus for the nearly concurrent trial MTN-007, A Phase 1 Randomized, Double-Blinded, Placebo-Controlled Rectal Safety and Acceptability Study of Tenofovir 1% Gel. A Memorandum of Understanding exists between the MTN and the IPCP's PI. In this protocol and all references/publications, the trial and its' results will be referred to as "RMP-02/MTN-006".

The RMP-02/MTN-006 and MTN-007 clinical protocols have been developed to assess the safety and pharmacology of tenofovir gel when used rectally in men and women as well as address critical questions in rectal microbicides through explorative objectives. Although a single combined study was considered, the complexity of the study and the potential participant burden were considered to be substantial and as a result the protocol teams felt compartmentalization of key activities into two concurrent and mutually supportive clinical studies was a more efficient, less burdensome and safer approach. RMP-02/MTN-006 will concentrate on systemic, mucosal and tissue pharmacology and pharmacodynamics of tenofovir following administration, including ex-vivo challenge of biopsies with HIV as a potential marker for retained tissue anti-HIV activity. MTN-007 will focus on accumulating Phase I safety and acceptability data, while providing in its explorative objectives the data needed to determine maximum and minimum parameters for a suite of potential rectal safety assays using comparisons of the results from the Gynol II® and placebo arms. In order to assure comparability both protocols have been operationally (general scheme, procedures, inclusion and exclusion criteria, etc.) harmonized. The trials also have in common the basic suite of rectal safety assays, which have been developed by the UCLA MDP team, and are implemented at both clinical sites. Together these two trials should act synergistically to determine/establish: (1) Initial safety of tenofovir gel as a rectal microbicide; (2) Pharmacokinetic/pharmacodynamic parameters for rectal tenofovir gel use; and (3) Further validate the proposed rectal safety suite by establishing maximum (Gynol II®) and minimum (HEC Placebo) mucosal responses, while proactively managing participant and clinical site burden. Additionally, the co-sponsorship of the RMP-02/MTN-006 trial (Pittsburgh and UCLA sites) will support the transition of the UCLA site–developed safety suite to the MTN Network Laboratory (RMP-02/MTN-006), which will then disseminate the suite to the MTN-007 sites.

<u>Vulnerability of the Rectal Compartment to HIV Infection</u>

The rectal compartment is highly vulnerable to HIV transmission. A single layer of columnar epithelium separates the intestinal lumen from the lamina propria. The lamina propria is populated with a broad range of HIV target cells including macrophages, dendritic cells, and activated CD4+ T lymphocytes expressing the chemokine receptor 5 (CCR5) and CXC chemokine receptor 4 (CXCR4) HIV-1 coreceptors.[6] It is likely that the immune composition of the rectal mucosa is at least partially responsible for the 10-20 fold increased risk of HIV transmission associated with anal[7, 8] compared to vaginal intercourse[9, 10]. Any product which induces local inflammation is likely further to increase this risk by recruiting and/or activating the immune target cells.

It remains unclear which factors may augment or reduce the risk of HIV transmission via the rectal route. The vulnerability of the rectal epithelium to the trauma of sexual intercourse suggests that the sub-epithelial mucosa may frequently be exposed to infected luminal contents and, therefore, to the risk of systemic infection. The implications are that one cannot rely simply on epithelial integrity and focus on strategies associated with luminal absorption of infectious agents. Consequently, this study will also assess the following sub-epithelial mucosal endpoints.

Epithelial sloughing

Rectal lavage and examination of effluent for shedding of epithelial cells has been used characterize the rectal safety profile of microbicide candidates in murine, non-human primate, and human studies. Using this approach, it is clear that N-9 is associated with transient epithelial disruption. Substantial reconstitution of these changes occurred by 2 hours and microscopically normal epithelium was noted after 24 hours.[11, 12] In contrast, administration of VivaGel®, C31G, Carraguard, or UC781 to non-human primates did not result in epithelial desquamation.[13-16] Since the epithelial sloughing does not have an absolute, quantifiable threshold, the scoring system of 0-to-4 will be used.[14] Each of four petri-dish quadrants is scored as either 0 or 1, indicating either the absence or presence of epithelial sheets. The total score for each preparation can therefore be from 0-4. Changes pre and post treatment will be analyzed.

Intestinal histopathology

Histopathological assessment of intestinal tissue is a routine method of demonstrating mucosal abnormality associated with gastrointestinal diseases such as ulcerative colitis, Crohn's disease, and gluten enteropathy (celiac disease). In general, mucosal change in these diseases can be quite dramatic and microbicide induced changes may be quite subtle. As a consequence we propose to use a qualitative scoring system developed by the inflammatory bowel disease community[17] and adapted for use in HIV Prevention Trials Network (HPTN) 056, Characterization of Baseline Mucosal Indices of Injury and Inflammation in Men for Use in Rectal Microbicide Trials (please see Appendix VI).[18] Prior to the HPTN 056 study, one rectal microbicide study using histological data[19], employed a simple scoring system of normal, slightly abnormal, or abnormal. Using this histological system 69% of the placebo recipients and 89%of the N-9 recipients had slightly abnormal or abnormal rectal biopsies. The scoring system developed for the HPTN 056 study might provide better discrimination between abnormal and normal histology.

Intestinal mucosal mononuclear cell phenotype

Enzymatic digestion of intestinal biopsies and flow cytometric analysis of T cell populations[20] will be used to determine if product administration is associated in changes in mucosal T cell populations, co-receptor expression, or T cell activation. Co-receptor expression (e.g., CCR5, CXCR4, etc.) on exposed mucosal immunocytes is important for HIV-1 entry. In healthy HIV-1 seronegative individuals, the expression level of CCR5 is increased seven-fold in mucosal mononuclear cells (MMC) compared to peripheral blood mononuclear cells (PBMC)[6]. CXCR4, however, is expressed on

CD45RO+ T cells in similar levels in MMC and PBMC. It was recently shown that MMC are more easily infected with HIV-1 than PBMC.[21, 22] Explanations for the high susceptibility to HIV-1 of MMC may include the increased expression of HIV-1 co-receptors, especially CCR5, as well as the activation status of the MMC. The expression of CCR5 has been shown to be up-regulated by pro-inflammatory and T helper (Th)-1 cytokines, while Th-2 cytokines up-regulate CXCR4.[23, 24] This suggests that expression of CCR5 and CXCR4 is partly controlled by Th1/Th2 type of cytokines, which have been shown to be up-regulated in rectal mucosa from HIV-infected patients.[18] It will be important to ascertain whether microbicidal agents trigger similar responses and associated increased vulnerability to HIV infection.

Cytokine profile in rectal secretions

As discussed above, measurement of cytokines or chemokines in mucosal tissue or local secretions may provide important information about the potential for a candidate microbicide to induce mucosal toxicity. In addition to the messenger RNA (mRNA) analysis of intestinal tissue biopsies we will also quantify cytokine levels in rectal secretions using the Luminex® technique that can measure multiple cytokines or chemokines in small volumes (< 100 μl) of rectal secretions. We will use Luminex® to measure the following cytokines or chemokines: IL-1β, IFN-γ, TNF-α, IL-6, IL-8, IL-12, MIP-1α, MIP-1β, and RANTES.

Fecal calprotectin

Stool samples will be collected at the time of rectal lavage for the measurement of fecal calprotectin. Calprotectin accounts for 60% of the cytoplasmic protein fraction of polymorphonuclear granulocytes and is also found in monocytes, macrophages, and eosinophils.[25, 26] Calprotectin plays an important role in innate immunity and has antibacterial, antifungal, and immunomodulatory effects *in vivo*. Because intestinal granulocytes end their lifespan by migration through the intestinal wall and granulocyte-derived calprotectin can be found in feces, calprotectin is felt to be a useful indirect index of mucosal inflammation.[27, 28] In fact, fecal calprotectin levels are elevated in inflammatory bowel disease[29, 30] and correlate well with disease activity in Crohn's disease and ulcerative colitis. In addition, fecal calprotectin levels have been found to be significantly elevated in first-degree relatives of patients with Crohn's disease even though all the relatives were clinically asymptomatic.[31] These data suggest that the fecal calprotectin assay may be sufficiently sensitive to respond to subtle increases in mucosal inflammation. Fecal calprotectin has a sensitivity of 96% in discriminating between healthy controls (2mg/l; 95% CI 2-3 mg/l) and subjects with active inflammatory bowel disease (91 mg/l; 95% CI 59-105 mg/l).[30]

Microflora

Assessment of pre/post exposure changes in rectal microflora will be conducted (please see Appendix V). It is currently unknown whether rectal administration of tenofovir 1% gel will prompt a change in the rectal microflora. Transient reductions in vaginal lactobacilli have been noted with the administration of candidate microbicides. There are no rectal microflora data from human microbicides although non-human primate

studies have not demonstrated significant changes in rectal microflora following rectal administration of vaginal microbicides.

Assessing Acceptability of Rectally-Administered Microbicides

Prevention tools are effective only if used. The limited use of condoms by many at-risk individuals illustrates the importance of a product's acceptability and perceived need, i.e., the willingness of the users of the product to use it correctly and consistently. This study will explore the acceptability of tenofovir 1% gel for rectal use by means of a behavioral assessment that includes both quantitative and qualitative methods. This assessment, administered to all (placebo and drug-exposed) participants, will evaluate not only product acceptability, but also the acceptability of a vaginal applicator for rectal product application. The behavioral assessment consists of three elements: 1) a Baseline Behavioral Questionnaire 2) a Product Acceptability Questionnaire, and 3) an in-depth phone interview. The quantitative questionnaires were developed based on in-depth qualitative interviews of 20 participants in the first phase of R01 HD046060 "Topical Microbicide Acceptability," (Carballo-Diéguez, PI), a study that focused on acceptability of rectal microbicides among men and women.[32, 33] Subsequently, the questionnaires were administered to more than 100 MSM in Boston and New York, and to 36 men and women participating in P4 Aim 1 of this rectal microbicide study (Peter Anton, PI). These studies showed that there were no comprehension problems or other difficulties. There are advantages to using the same product-acceptability instrument across studies, since this allows more valid post-hoc comparisons across studies.

2.2 Description of Study Products

2.3 TDF (Tenofovir tablet)

Tenofovir disoproxil fumarate (TDF) is currently approved under the trade name Viread® for the treatment of HIV-1 infection in adults. Tenofovir disoproxil fumarate is the oral pro-drug of tenofovir (PMPA, 9-[(R)-2-(phosphonomethoxy)propyl] adenine monohydrate), an acyclic nucleotide analog with activity *in vitro* against retroviruses, including HIV-1 and HIV-2, as well as hepadnaviruses. Further information on TDF is available in the current version of the Viread® Package Insert.

2.3.1 Mechanism of Action

Once absorbed, TDF is rapidly converted by diester hydrolysis to tenofovir (PMPA). Once inside the cell, the tenofovir is then phosphorylated by cellular enzymes to tenofovir diphosphate, a competitive inhibitor of HIV-1 reverse transcriptase (RT) that terminates the growing deoxyribonucleic acid (DNA) chain. Tenofovir diphosphate is a weak inhibitor of mammalian DNA polymerases α, β, and mitochondrial DNA polymerase γ.

2.3.2 Strength of Study Product

The strength of the TDF tablets will be the dose approved by the Food and Drug Administration (FDA) for the indication of treatment of HIV-1 infection in adults (300 mg). For the treatment of HIV-1 infection, TDF is administered once daily as one orally administered 300 mg tablet and has excellent activity against wild type and many drug resistant viruses.

2.4 Tenofovir 1% Gel (Tenofovir Gel)

Tenofovir gel contains approximately 1.0 g/100mL of tenofovir (PMPA). Further information is available in the current version of the tenofovir gel investigator's brochure. Please note that PMPA is referred to as tenofovir 1% gel throughout the remainder of the protocol.

2.4.1 Mechanism of Action

Tenofovir is an acyclic nucleotide analogue of adenosine monophosphate. Once inside the cell tenofovir is phosphorylated by cellular enzymes to form tenofovir diphosphate. Tenofovir diphosphate is a competitive inhibitor of HIV-1 RT that terminates that growing DNA chain.

2.4.2 Strength of Study Product

The strength of the tenofovir gel will be the strength (1%) previously tested in HPTN 050 (IND 55,690), CONRAD A04-095 (IND 73,382) and A04-099 (IND 73,382), HPTN 059 (IND 55,690), MTN-001 (IND 55,690), and MTN-002 (IND 55,690). From the current good manufacturing practices (cGMP) formulators (DPT Pharmaceuticals), the density of the gel is 1.06 g/mL which has been rounded up to 1.1 grams/mL of gel. Each gram of gel contains 10 mg of tenofovir, resulting in a total of 44 mg of tenofovir delivered in each application (or 0.044 grams of tenofovir).

2.5 HEC Placebo Gel

The placebo gel is the hydroxyethylcellulose (HEC) or Universal placebo[34], a vaginal product which contains HEC as the thickener, purified water, sodium chloride, sorbic acid and sodium hydroxide. HEC is used to approximate the viscosity of other microbicide gel candidates.

2.5.1 Mechanism of Action

HEC gel contains hydroxyethylcellulose as the gel thickener, purified water, sodium chloride, sorbic acid and sodium hydroxide[34]. The gel is isotonic and formulated at a pH of 4.4 to avoid disrupting the normal rectal pH (which physiologically has a broad normal range).

2.5.2 Strength of Study Product

2.7% w/w HEC gel will be used in this study.

2.6 *In Vitro* Studies

2.6.1 Tenofovir

Formulation Testing

The physiologic properties evaluated included osmolarity, viscosity, pH, and *in vitro* release. Tenofovir 1% gel and its placebo were 11.5-fold and 11-fold, respectively, greater than iso-osmolar conditions implying that this formulation is hyper-osmolar. Both gels were approximately pH 4.4, which is similar to the vaginal environment. Viscosity evaluations were conducted for both tenofovir 1% gel and its placebo gel. The viscosity of the tenofovir 1% gel and its placebo at 30 rpm showed reproducible results in 3 trials. Both gels were found to be shear thinning in nature. Thinning viscosity indicates that it is “flowable” which allows for even spread across mucosal surfaces.

Safety Testing in Cell Lines

Tenofovir gel (1%) and its placebo gel were evaluated for its effect on the viability of colorectal Caco-2 epithelial cell line. Viability of the Caco-2 epithelial cell line after a 24-hour exposure to tenofovir 1% or placebo gel showed minimal reduction; a 1:10 dilution of both gels yielded ≥ 60% viability. To put this into perspective, the over-the-counter preparations of N-9 (3%) and KY® jelly (Johnson & Johnson, New Brunswick, NJ) need to be diluted a minimum of 1:1000 and 1:100, respectively, of their original formulation to yield ≥ 60% viable epithelial cultures.[35] Using the 1:10 dilution, a “2 hours per day for 5 days” exposure experiment was performed to evaluate the impact of extended use on Caco-2 cell viability. No reduction in Caco-2 viability was noted after the 5-day exposure, indicating that the 1:10 dilutions of both gels were stable concentrations for use in further analysis.

The ability of mucosal epithelial cells to maintain an intact, polarized monolayer in the presence of a microbicide is a possible predictor of that product’s safety on colorectal tissues because the epithelial layer is integral in the protection against sexually transmitted infections including HIV. Therefore, Caco-2 cells were plated in duplicate in transwell plates, and their transepithelial resistance (TER) was measured using the Millicell® ERS meter (Millipore, Billerica, MA) to form a polarized monolayer. When the cells reached plateau TER, a 1:10 dilution of tenofovir 1% or placebo gel and a 1:50 dilution of N-9 were added to the apical side of the transwells. The TER was measured over a 24-hour period. Tenofovir 1% and placebo gel maximally reduced the TER as compared to the control (68% and 59% respectively) after 4 hours. Over the next 20 hours, the TER returned to control TER levels. N-9-treated wells, however, continually declined and reached background levels after 4 hours. These data suggest that hyperosmolar nature of the tenofovir 1% gel formulation resulted in the transient loss of the epithelial monolayer resistance.

Safety Testing in Colorectal Explant Cultures

Tenofovir 1% gel and its placebo were tested for toxicity to colorectal explant cultures. Briefly, duplicate polarized tissues were exposed to product for 18 hours and then washed to remove excess product. One of the duplicate tissues was incubated with MTT, Formazan [1-(4,5-dimethylthiazol-2-yl)-3,5-diphenylformazan] to measure the reduction to formazan and the other was placed in 10% buffered formalin for histology. Up to 5 different tissue donors were used. Tenofovir 1% gel and the placebo did not reduce the viability of the colorectal explants as based on the MTT assay. When assessed for histologic changes, the tenofovir 1% gel and to a lesser extent the placebo treated tissues showed fractured epithelium with an intact lamina propria. This result may be due to the hyper-osmolar formulation of the tenofovir 1% gel and this data would correspond to the changes noted for the epithelial cell line TER.

Efficacy Testing in Colorectal Explant Cultures

The efficacy of the tenofovir 1% and placebo gels were assessed using the polarized colorectal explant culture system[36]. The explants were set-up in duplicate and exposed to HIV-1 without or with 1:5 dilutions of tenofovir 1% or placebo gels on the apical side. The explants were allowed to culture overnight and then washed. The explants were followed for 21 days and HIV-1 replication was assessed by the production of p24 in the basolateral supernatant. The tenofovir 1% gel was effective at preventing HIV-1 infection of the tissue. The placebo was also partially effective at reducing the HIV-1 infection. This has been noted previously for other products that were evaluated .[36]

Anti-HIV-1 Activity

The *in vitro* antiviral activity of unformulated tenofovir against laboratory and clinical isolates of HIV-1 was assessed in lymphoblastoid cell lines, primary monocyte/macrophage cells and peripheral blood lymphocytes.[37, 38] The EC_{50} (50% effective concentration) values for tenofovir were in the range of 0.04 µM - 8.5 µM. In drug combination studies of tenofovir with nucleoside reverse transcriptase inhibitors (NRTI) (abacavir [ABC], didanosine [ddI], lamivudine [3TC], stavudine [d4T], zalcitabine [ddC], zidovudine [ZDV]), NNRTIs (delavirdine [DLV], efavirenz [EFV], nevirapine [NVP]) and protease inhibitors (amprenavir [APV], indinavir [IDV], nelfinavir [NFV], ritonavir [RTV], saquinavir [SQV]), additive/synergistic effects were observed. Tenofovir displayed antiviral activity *in vitro* against HIV-1 clades A, B, C, D, E, F, G, and O (EC_{50} values 0.5 µM - 2.2 µM) and showed strain specific activity against HIV-2 (EC_{50} values ranged from 1.6 µM to 5.5 µM).

Resistance

HIV-1 isolates with reduced susceptibility to tenofovir have been selected *in vitro.*[37, 38] These viruses expressed a K65R mutation in RT and showed a 2–4 fold reduction in susceptibility to tenofovir. Of note, this mutation also confers increased susceptibility to some other NRTIs, and is associated with approximately 50% reduction in the replicative capacity of HIV-1 (potentially resulting in a "less fit" virus).[39] Tenofovir-resistant isolates of HIV-1 have been recovered from some patients treated with Viread® in combination with certain antiretroviral (ARV) agents.[38] In treatment-naïve patients, 8/47 (17%) isolates from patients failing Viread® + 3TC + EFV through week 144 showed >1.4 fold (median 3.7) reduced susceptibility *in vitro* to tenofovir.

Cross-resistance

Cross-resistance among certain NRTIs has been recognized.[37, 38] The M184V/I and/or K65R substitutions selected *in vitro* by the combination of FTC and tenofovir are also observed in some HIV-1 isolates from subjects failing treatment with tenofovir in combination with either 3TC or FTC, and either abacavir, didanosine, or zalcitabine. Therefore, cross-resistance among these drugs may occur in patients whose virus harbors either or both of these amino acid substitutions. In treatment-experienced patients, 14/304 (5%) isolates from patients failing Viread® through week 96 showed >1.4 fold (median 2.7) reduced susceptibility to tenofovir. Genotypic analysis of resistant isolates showed a mutation in the HIV-1 RT gene resulting in the K65R amino acid substitution. HIV-1 isolates from patients (n = 20) whose HIV-1 expressed a mean of 3 ZDV-associated RT amino acid substitutions (M41L, D67N, K70R, L210W, T215Y/F, or K219Q/E/N) showed a 3.1-fold decrease in the susceptibility to tenofovir. Multinucleoside resistant HIV-1 with a T69S double insertion mutation in the RT showed reduced susceptibility to tenofovir.[37]

2.6.2 HEC Placebo Gel

Formulation Testing

Analyses of pH (HEC gel mixed with human seminal plasma, 8.03± 0.26) found that a HEC formulation did not show significant buffering capacity and could not acidify the alkaline pH of seminal plasma, a favorable property for a placebo formulation.[40] *In vitro* assessments of spermicidal activity utilizing human semen from healthy donors showed that HEC gel had no significant deleterious effects on sperm motility, even after 60-minute incubation.

Safety Testing in Cell Lines

Dilutions of the HEC gel in culture medium exhibited negligible toxicity to human vaginal epithelial cells (standard MTT assay), even at the lowest dilution tested (1:2)[34]. Exposure of human vaginal epithelial cells to the HEC gel resulted in minimal IL-1α induction, even at the lowest dilutions tested (lowest dilution, 1:2). Additional studies have shown that HEC gel is safe to peripheral blood mononuclear cells, and colorectal epithelial cell lines.[36, 41] Indeed, no changes in the transepithelial resistance was noted after HEC gel was applied.[41]

Safety Testing in Colorectal Explant Cultures

The HEC gel was applied to colorectal explant tissues using a polarized system.[36] For safety analysis the MTT assay and histology were performed. No observed reduction in the MTT levels or changes in the tissue architecture were noted.

Anti-HIV-1 Activity

Further analysis showed that this gel has no anti-HIV activity as it did not protect peripheral blood mononuclear cells, macrophage, or colorectal explant cultures from infection.[36, 41]

2.7 Animal Studies

2.7.1 Tenofovir and Tenofovir Disoproxil Fumarate (Oral)

Toxicology

Tenofovir and TDF administered orally in toxicology studies to rats, dogs, and monkeys at exposures (based on AUCs) ≥ 6 fold those observed in humans caused bone toxicity. In monkeys, bone toxicity was diagnosed as osteomalacia.[37] Osteomalacia observed in some monkeys appeared to be reversible upon dose reduction or discontinuation of tenofovir. In rats and dogs, bone toxicity manifested as reduced bone mineral density. The mechanism(s) underlying bone toxicity is unknown.

Four gravid rhesus monkeys were administered tenofovir subcutaneously once daily from 20 to 150 days of gestation (30 mg/kg; term: 165 ± 10 days).[42] Fetuses were monitored sonographically, and maternal and fetal blood and urine samples were collected to assess hematologic parameters, clinical chemistry, insulin-like growth factor (IGF) levels, and bone biomarkers. Fetuses were delivered by hysterectomy near term for necropsy and evaluation of bone-related mechanical properties. Results of these studies showed 1) normal fetal development, although overall body weights and crown-rump lengths were less than those for age-matched controls ($p \leq .03$); 2) a significant reduction in circulating IGF-I ($p < .001$); 3) a small reduction in fetal bone porosity ($p \leq .03$); and 4) transient alterations in maternal body weights and bone-related biomarkers during treatment. Results of these studies suggest that chronic fetal exposure to subcutaneous tenofovir at the maternal dose of 30 mg/kg throughout gestation can alter select fetal parameters and transiently affect maternal bone biomarkers.

Evidence of renal toxicity from oral TDF was noted in 4 animal species.[37] Increases in serum creatinine, blood urea nitrogen, glycosuria, proteinuria, phosphaturia, and/or calciuria and decreases in serum phosphate were observed to varying degrees in these animals. These toxicities were noted at exposures (based on AUCs) 2–20 times higher than those observed in humans. The relationship of the renal abnormalities, particularly the phosphaturia, to the bone toxicity is not known.

Carcinogenesis and Mutagenesis

Long-term oral carcinogenicity studies of TDF in mice and rats were carried out at exposures up to approximately 16 times (mice) and 5 times (rats) those observed in humans at the therapeutic dose for HIV infection.[37] At the high dose in female mice, liver adenomas were increased at exposures 16 times that observed in humans. In rats, the study was negative for carcinogenic findings at exposures up to 5 times that observed in humans at the therapeutic dose. TDF was mutagenic in the *in vitro* mouse lymphoma assay, but negative in an *in vitro* bacterial mutagenicity test (Ames test). In an *in vivo* mouse micronucleus assay, TDF was negative when administered to male mice.

Reproductive Toxicity

There were no effects on fertility, mating performance or early embryonic development when TDF was administered to male rats at a dose equivalent to 10 times the human dose based on body surface area comparisons for 28 days prior to mating, and to female rats for 15 days prior to mating through day seven of gestation.[38] There was, however, an alteration of the estrous cycle in female rats. Reproduction studies performed in rats and rabbits at doses up to 14 and 19 times the human dose based on body surface area comparisons revealed no evidence of impaired fertility or fetal harm due to tenofovir. Subcutaneous administration of TDF to pregnant rhesus macaques resulted in a fetal/maternal concentration of 60%, demonstrating that TDF does cross the placenta.[43] Studies in rats have shown that tenofovir passes into breast milk.

Effectiveness

Adult male rhesus macaques were inoculated intra-rectally once weekly for 14 weeks (or until they became infected) with $SHIV_{SF162P3}$ at 10 median tissue culture infective doses (3.8 x 10^5 virus particles) that were approximately five-fold higher than the HIV-1 RNA levels noted in human semen during acute infection.[44] Of the 12 macaques studied, 4 received oral TDF daily, 4 received oral TDF once weekly, and 4 control animals received no TDF. The control animals became infected after receiving a median of 1.5 virus inoculations; macaques receiving TDF daily and those receiving TDF weekly became infected after a median duration of 6.0 and 7.0 weeks, respectively. The animals continued to receive TDF after infection. One macaque in the daily TDF group remained uninfected after 14 weekly inoculations of virus. The K65R mutation was not detected in viral sequences from the infected animals through 31 weeks of the study. Although infection was delayed in treated macaques, compared with control macaques, the differences were not statistically significant (p = .315); however, the study was limited by the small numbers of animals evaluated and the variability in blood TDF levels that resulted from oral dosing. These data demonstrate that treatment with oral TDF provided partial protection against SHIV infection but ultimately did not protect all TDF treated animals against multiple virus challenges.

2.7.2 Tenofovir 1% Gel (Topical)

Pharmacokinetics-Vaginal Administration

Single-dose PK of vaginally administered radiolabeled tenofovir gel in female rabbits has been previously examined (0.5 mL, 1% w/v tenofovir, 5 mg/animal, 50 µCi/kg)[45]. Plasma radioactivity concentrations were highest at the first sample time point (0.5 h) and below the level of quantification at 24 hours. PK parameters including the proportion of dose absorbed systemically could not be estimated, due to the very low plasma concentrations.

In a tissue distribution study using the same radiolabeled tenofovir 1% vaginal gel formulation, dose and strength as the above study, eighteen female rabbits were administered an intravaginal dose using a gavage needle.[46] An additional eighteen rabbits received an intravaginal dose of 3% w/v tenofovir (15 mg per animal). Analysis of vaginal tissue sections found no clear relationship between tissue concentration and

dose, with no consistent pattern of distribution. Very little radioactivity was recovered in non-vaginal tissues. Concentrations in blood (0.002 to 0.047 µg-eq/g of tissue) exemplified the variability of distribution of the product although the effect of oral absorption due to grooming behaviors of the animals may have impacted these results.

The PK, excretion and tissue distribution of ^{14}C-PMPA were evaluated in rats following intravaginal administration of an earlier formulation of tenofovir gel containing propylene glycol.[47] Four female rats received a single intravaginal dose administered as an aqueous gel containing 20 mg tenofovir/g. Plasma concentrations of total radioactivity were highly variable; this was attributed to inconsistent retention of the formulation within the vagina, or possibly oral absorption related to grooming. The apparent maximum serum concentration (C_{max}) for tenofovir occurred at the earliest time point (15 minute), suggesting that absorption from the vagina was relatively rapid. Thereafter, plasma concentrations declined with an approximate half-life of 1.6 hours. The bioavailability of intravaginal tenofovir was estimated by comparison of the observed $AUC_{(0-24)}$ with historical AUC data for an intravenous dose of 10 mg/kg tenofovir in rats (9.71 µg h/mL). The observed systemic bioavailability of intravaginal tenofovir was 7.9%.

In the excretion and distribution study, two groups of four additional rats received a single intravaginal dose of ^{14}C-PMPA (approximately 10 mg/kg, 100 µCi/kg) administered as aqueous gel containing 20 mg tenofovir/g. This study found that much of the dose was lost from the vaginal orifice by leakage. Vaginal tissue contained 0.1% of the dose and less than 0.01% of the dose was recovered in the ovaries and uterus.

The PK of radiolabeled tenofovir gel was evaluated via plasma and vaginal biopsies collected from four rhesus macaques following single-dose intravaginal tenofovir 1% vaginal gel.[46] Radioactivity was detected starting at 15 minutes post application, with peak concentration of tenofovir in vaginal tissue at 8 hours and remaining high at 12 hours. No significant radioactivity was detected in whole blood or plasma.

Systemic and vaginal tissue bioavailability was assessed in female white New Zealand rabbits following single and multiple intravaginal doses (twice a day for 7 or 14 days) of 1 mL of tenofovir 1% gel or a single intravenous (IV) solution of 10 mg tenofovir.[46] Animals that were vaginally and intravenously dosed were sacrificed at the following time points: 1) 8 hours after single intravenous dose; 2) 4 hours after single vaginal dose; 3) 8 hours after single vaginal dose; 4) 4 hours after the 13th twice-daily vaginal dose; and, 5) 4 hours after the 27th twice-daily vaginal dose (see table below). After sacrifice, vaginal tissue was rinsed to remove topical tenofovir, and biopsy samples were taken. Both vaginal rinse and vaginal tissue were analyzed for tenofovir content. System absorption following a single intravaginal dose was barely detectable, and only within the first 30 minutes. Multiple intravaginal administrations of tenofovir1% gel and the single IV administration of 10 mg tenofovir resulted in systemic levels of tenofovir (see Table 2).

Table 2: Tenofovir Bioassay Data

	Mean 1st Rinse Vaginal Surface (ng/mL)	Mean Vaginal Tissue Concentration (ng/g)	C_{max} (ng/mL)	AUC (0-4 h) (ng*h/mL)
Single IV, 8 h	**362** (19-990)	**950** (120-5,019)	**10,221**	**4,013** (3,192-4,503)
Single vaginal, 8 h	**97** (7-415)	**940** (10-7,277)	**3**	--
Single vaginal, 4 h	**1,441** (2-5,100)	**2,817**(35-11,780)	**5**	--
Twice daily x 7d vaginal, 4 h	**1,086** (145-4,369)	**3,146** (448-14,429)	**239** (29-808)	**342** (54-1,037)
Twice daily x 14d vaginal, 4 h	**3,361** (33-8,000)	**11,409** (245-50, 102)	**71** (24-197)	**94** (12-229)

Pharmacokinetics-Rectal Administration

Only preliminary assessments of single dose rectal administration of 1% tenofovir gel (PMPA) have been conducted in the setting of a pilot macaque efficacy trial.[48] Plasma samples were assayed for tenofovir concentration by the Clinical Pharmacology and Analytical Chemistry Core of the University of North Carolina Center for AIDS Research. Drug concentrations in plasma were determined by a validated high pressure, liquid chromatography (HPLC) method with ultraviolet detection.[49] This method utilized a dynamic range of 10-10,000 ng/mL, with intra and inter-day variability of <10% across this range. Total tenofovir concentrations were assayed in tissues using a fully validated HPLC method with mass spectrometry detection.[48]

Analysis of intestinal tissue samples collected at necropsy showed that all tenofovir-dosed animals had measurable concentrations of drug in homogenates of colorectal tissue at concentrations between 20.8 and 54.2 µg/g protein but no drug was detected in homogenates from the small intestine. Tissues from untreated animals acted as negative controls. To indirectly estimate the amount of intracellular phosphorylated tenofovir in tissues, samples were analysed with (to measure the combination of tenofovir + tenofovir monophosphate + tenofovir diphosphate) and without (to measure tenofovir only) phosphatase hydrolysis. Subtracting the concentration of tenofovir obtained from tissue samples without phosphatase, from the concentration of tenofovir obtained from tissue samples with phosphatase, demonstrated that between 46-75% of total tenofovir in tissues was present as the intracellular monophosphate and diphosphate forms. Based on intracellular data describing tenofovir monophosphate : diphosphate ratios,[50-52] it was estimated that approximately 30-60% of total tenofovir in tissues was present as the intracellular diphosphate form. The relatively low rectal dose of tenofovir applied, equating to an average of 10µg/Kg, of which a maximum of 0.19% was detected in plasma 15 minutes later, was far below the dose used in oral pre-exposure prophylaxis.[53]

Toxicology-Vaginal Administration

The preclinical toxicity of tenofovir gel has been evaluated in 14-day rat and 10-day rabbit vaginal irritation and toxicity studies[45, 54]. Daily intravaginal administration of

tenofovir gel produced no vaginal irritation in rats (≤10% tenofovir) and minimal to mild vaginal irritation in rabbits (3% or 10% tenofovir).

14-Day Vaginal Irritation and Toxicity Study of Tenofovir Gel in Rats
Ten female Sprague Dawley rats/group received either 0% (vehicle control), 1%, 3%, or 10% tenofovir gel (2.5% HEC formulation) by intravaginal administration (0.5 mL/dose) once daily for 14 days. There were no mortalities, and no tenofovir-related clinical signs of toxicity or changes in body weight, food consumption, or absolute/relative kidney weights. Individual and mean vaginal (gross) irritation scores for all tenofovir-dosed animals sacrificed at Day 15 were graded as 0 (no erythema or edema); microscopic irritation scores for the vagina, cervix, ovaries, uterine horns, and vulva were graded as 0 (normal histology). No tenofovir-related histopathological effects on the vagina, cervix, ovaries, uterine horns, vulva, or kidneys were observed.

10-Day Vaginal Irritation Study of Tenofovir Gel in Rabbits
The potential irritant effects of tenofovir were evaluated in vaginal tissues of female New Zealand White rabbits using three different gel formulations (2.5% HEC or 1.0 – 2.0% Carbopol® 1342).[55] This study consisted of eleven treatment groups (five rabbits/group) that received either: a sham treatment or Conceptrol® (positive control); 0%, 0.3%, 1.0%, 3.0%, or 10.0% tenofovir formulated in the HEC gel preparation; or 0% or 3.0% tenofovir formulated in a 1.0% or 2.0% Carbopol® 1342 gel preparation. With the exception of the sham dose group, all rabbits received dose formulation (1.0 mL/dose) daily applied topically to the mucosal surface of the vaginal vault for 10 consecutive days. No mortalities and no tenofovir-related clinical signs of toxicity or body weight changes were observed in this study. Group composite vaginal irritation scores for the 10% tenofovir topical gel (HEC formulation), 0% tenofovir (1.0% Carbopol® 1342 formulation), and Conceptrol® (positive control) dose groups were each rated as "mild." Composite vaginal irritation scores rated "minimal" were observed for all other tenofovir, vehicle or sham treatment groups, regardless of the formulation. No unacceptable level of mucosal irritation was observed in any treatment group based on the protocol-derived criteria for this animal model. Generalized erosion and/or ulceration were observed only in animals receiving Conceptrol® positive control (two of five) or the 10% tenofovir topical gel (two of five).

Toxicology-Rectal Administration

14-Day Rectal Irritation Study of Tenofovir Vaginal Gel in Rabbits
Forty (40) New Zealand White rabbits (approximately 10-12 weeks of age and weighing in the range of 2.0 to 2.5 kg at initiation of treatment) were assigned to five dose groups (one sham control, one placebo control and three active test article) consisting of four animals per sex per group under Good Laboratory Practices [(GLP) Pacific BioLabs, Hercules, CA]. The placebo control and active test articles consisted of tenofovir placebo vaginal gel and three different concentrations (1%, 3%, and 10%) of tenofovir vaginal gel respectively. The lubricant for the sham control group was K-Y Jelly from a commercial source.

All female animals were dosed for 14 days and all male animals were dosed for 15 days. Animals in Groups 2 to 5 received 1 mL doses of the respective placebo or test articles via rectal administration for 14/15 consecutive days. A short, soft catheter was attached to a syringe and filled with 1 mL of the appropriate test article. Animals in Group 1, (sham control) underwent the same treatment procedure for 14/15 days with the exception that no dose was administered and the catheter was lubricated with a non-irritating lubricant (K-Y Jelly) prior to insertion. The rectal route of administration was selected as it is the intended clinical route of administration.

Table 3: Rectal Irritation Study

Group	Intervention
1	Sham
2	tenofovir placebo gel
3	tenofovir 1% gel
4	tenofovir 3% gel
5	tenofovir 10% gel

The test article, vaginally formulated tenofovir 1% gel, was well tolerated at dose concentrations (1 mL dose volume) of 1% (10 mg/dose), 3% (30 mg per dose) and 10% (100 mg per dose) when administered as a daily rectal dose for 14 days to female rabbits or 15 days to male rabbits. There was no mortality in this study, and there was only one clinical finding that was potentially study-related: redness at the site of administration in one animal on one day of dosing. There was no evidence of a test article effect on body weight, body weight gain or food consumption over the dose period.

The test article, at the concentrations tested, was without significant effect at the rectal site of administration. Gross pathology at necropsy provided no evidence for tissue damage or inflammation of the rectum or surrounding tissues at the concentrations tested; histopathological evaluation of the rectum and parts of the colon immediately adjacent to the rectum also showed no effect at the concentrations tested. Each rectum sample was subsectioned into proximal, mid and distal sections (in relation to the site of test article application) for histopathological analysis. Within each section, at least 5 subsections were evaluated for inflammation and other types of lesions. As mentioned, no differences were seen.

Rectal administration of the test articles produced little evidence of test article related systemic effects, despite measurable systemic exposures to tenofovir. At necropsy, gross pathology provided no *in situ* evidence for tissue damage or target organ effects. Changes in several hematology, coagulation and clinical chemistry parameters that reached statistical significance were not considered test article related because they were typically sporadic, not dose-related, and were present in only one gender of rabbit on each occasion. Organ weight changes also reached statistical significance on occasion, but these were also considered not to be test article related for the same reasons cited above, i.e., sporadic and not dose-related. No tissues or organs other than the rectum and colon were examined for histopathological changes.

Rectal application of test articles resulted in measurable systemic concentrations of Tenofovir at all dose levels, and after the first dose on Day 1 and the Day 14 dose. Tenofovir exposures were variable on Day 1; however, by Day 14 plasma concentrations were more consistent amongst individual animals and there was a clear dose-related increase in tenofovir exposures in both male and female rabbits. Systemic exposures to tenofovir were comparable in female and male rabbits. Absorption of tenofovir was relatively rapid, with the plasma T_{max} occurring at 1 h on Day 1 (for most dose groups) and at 2 h (female rabbits) and 4 h (male rabbits) on Day 14. Mean C_{max} values on Day 1 ranged from 11.7 ng/mL (Group 4 females) to 59.0 ng/mL (Group 3 females), except Group 3 males where the C_{max} was 1182 ng/mL. Mean C_{max} values on Day 14 ranged from a low of 32.3 ng/mL (Group 3 males) to 265 ng/mL (Group 5 males). The mean T_{max} and C_{max} values for Group 3 males on Day 1 were skewed by one male rabbit with a very high Tenofovir plasma concentration at 24 h post dose of 4210 ng/mL. The elimination half-life for tenofovir could not be determined with accuracy due to the variable exposures on Day 1, and a poorly defined terminal elimination phase on Day 14. For those groups where a half-life could be measured on Day 14, the $t_{1/2}$ for tenofovir ranged from 11.3 to 16.2 hours. It is possible that continued absorption of Tenofovir from the rectal site of administration contributed to the inability to accurately measure half-life on Day 14. Tenofovir plasma concentrations increased in both female and male rabbits with increasing dose. However, the increase in exposure was somewhat less than dose proportional. On Day 14 when tenofovir plasma concentrations were most consistent across individual animals, the decrease in dose-proportional exposure for C_{max}/Dose between Group 3 (10 mg) and Group 5 (100 mg) was 66% and 18% for female and male rabbits, respectively. The decrease for AUC_{last}/Dose between Group 3 and Group 5 was 52% and 32% for female and male rabbits, respectively. There was a marked increase in tenofovir exposure over the 14 days of rectal administration. Accumulation ratios (AUC_{last} Day 14/AUC_{last} Day 1) varied from 7.2 to 23.7 across dose groups.

The No Observed Adverse Effect Level (NOAEL) for rectal administration of test article in this study was greater than the highest concentration tested, i.e., >10% tenofovir in vaginal gel (a 100 mg dose).

Effectiveness-Vaginal Administration

Six independent non-human primate studies provided some degree of evidence for efficacy using 1% or 10% gel vaginally, to reduce/prevent transmission (Table 4). [46] Although these data are limited and a powered statistical determination as to the efficacy of tenofovir 1% gel versus 10% cannot be made, empirical examination of the efficacy data identifies tenofovir 1% gel as the lowest efficacious concentration tested when given within two hours of virus challenge. All studies used SIVmac251, a highly infectious SIV isolate, and Indian-origin rhesus macaques (with the exception of study 6). Study 1 demonstrated protection of all four macaques that received 10% tenofovir gel as compared to no protection in the 2 macaques that received placebo gel. Likewise in study 2, 11 of 15 macaques that received 1% or 10% tenofovir gel were protected as compared to no protection in the 5 untreated control macaques that received no gel product. In studies 3, 4, and 5, <100% of the untreated controls were infected making these data problematic to interpret.

Table 4: Use of Topical Tenofovir Gel to Prevent Vaginal Transmission of SIV

Study*	Number of Exposures	Treatment	Time of Administration	Number Infected	Progesterone Pretreatment
1	2	1 mL vehicle	-24 h, 0 h, 24 h, 48 h	2 of 2	No
		10% tenofovir	-24 h, 0 h, 24 h, 48 h	0 of 4	
2	1	Untreated control	N/A	5 of 5	No
		10% tenofovir	-24 h, -15 m, + 24 h	1 of 5	
		1% tenofovir	-24 h, -15 m, + 24 h	1 of 5	
		1% tenofovir	-15m	2 of 5	
3	1	Untreated control	N/A	2 of 5	No
		Vehicle	-15 m	1 of 5	
		1% tenofovir	-15 m	1 of 5	
		1% tenofovir	-2 h	3 of 5	
		1% tenofovir	-8 h	1 of 5	
4	1	Untreated control	N/A	4 of 5	No
		Vehicle	-15 m	2 of 5	
		1% tenofovir	-15 m	1 of 5	
		1% tenofovir	-2 h	1 of 5	
		1% tenofovir	-8 h	2 of 5	
5	1	Untreated control	N/A	2 of 5	No
		Vehicle	-2 h	2 of 5	
		1% tenofovir	-2 h	0 of 5	
6	1	1% tenofovir	-12 h	5 of 8	Yes
		Vehicle	-12 h	8 of 8	
		1% tenofovir	- 24 h	8 of 8	
		Vehicle	- 24 h	8 of 8	
		Untreated control	N/A	8 of 8	
		1% tenofovir	-72 h, -48 h, -24 h	6 of 8	

* All studies were performed with the SIVmac251 isolate of SIV, and female rhesus macaques were inoculated intravaginally. Virus challenges were performed without progesterone pretreatment in Studies 1-5: macaques in Study 6 were pretreated with 30 mg Depo-Provera 30 days prior to viral challenge. The indicated studies were performed by 3 independent investigators with Studies 2, 3, 4, and 5 being performed by the same laboratory.

Study 6 was different from the first five studies in that Chinese-origin rhesus macaques were used and they were pretreated with progesterone before virus challenge to enhance susceptibility to infection and synchronize reproductive cycles. This study was designed to determine whether topical dosing of tenofovir gel could be disassociated from the coital act while remaining an effective microbicide, in a regimen consistent with the long intracellular half-life of the active metabolite, tenofovir diphosphate. A total of 48 macaques, pretreated with a 30 mg dose of depomedroxyprogesterone acetate (DMPA) 30 days prior to viral challenge, were divided into 6 groups of 8 animals each. Group 1 received one topical vaginal dose of tenofovir 1% gel 12 hours prior to one intravaginal viral challenge with a dilution of SIVmac251 stock representing approximately 50 $TCID_{50}$. In parallel, Group 2 received matched placebo gel. Group 3 received a single dose of tenofovir 1% gel 24 hours prior to viral challenge. The matched placebo gel was administered to Group 4 24 hours prior to viral challenge. Group 5 was an untreated control group receiving only the viral challenge. A single dose of tenofovir 1% gel was administered topically to Group 6 animals at 72, 48, and 24 hours prior to viral challenge. Thus, Group 6 animals received 3 consecutive days of

gel; Group 4 served as the placebo control for Group 6. Based on plasma viral load, all untreated control animals became infected as did all placebo gel-treated macaques. Three animals were protected from infection in Group 1 receiving a single dose of tenofovir 1% gel 12 hours prior to virus exposure. Although no macaques receiving a single dose of tenofovir 1% gel 24 hours prior to virus exposure were protected, two of eight animals in Group 6 receiving multiple doses of tenofovir 1% gel remained uninfected. Infection status was confirmed using virus co-culture, seroconversion and lymph node DNA PCR. These data show 24 of 24 placebo gel-treated or untreated macaques became infected with SIVmac251 while 5 of 24 macaques were protected from SIV infection by vaginally administered tenofovir 1% gel.

Progesterone pretreatment (30 mg DMPA) is used in macaque studies to increase susceptibility to infection by a mechanism thought to involve thinning of the vaginal epithelium. It is generally required to achieve 100% infection in untreated control animals challenged with less infectious SHIV chimeric viruses. Although animals were pretreated with DMPA in this study but not the previous studies (1−5), this pretreatment may not be required for such a highly infectious virus as SIVmac251. In view of the potent infectivity of this virus, the lack of an endpoint in the animal titration of this stock (personal communication), and increased susceptibility resulting from progesterone pretreatment, it is possible that the amount of virus used was too high, thereby masking any protective effect. Further studies are required to understand the factors that impact protection by intravaginal tenofovir gel in the macaque model.

Effectiveness-Rectal Administration

The rectal application of tenofovir was evaluated for protective efficacy against rectal challenge with simian immunodeficiency virus (SIV) in a well established and standardized pre-clinical macaque model.[48] A total of 20 purpose-bred Indian rhesus macaques were used to evaluate the protective efficacy of topical tenofovir. Six animals received tenofovir 1% gel *per rectum* 15 minutes prior to virus challenge and 3 macaques received tenofovir 1% gel per rectum 2 hours prior to virus challenge, whereas 4 macaques received placebo gel and 4 macaques remained untreated. In addition, 3 macaques were given tenofovir gel 2 hours after virus challenge. Following intrarectal instillation of 20 median rectal infectious doses (MID_{50}) of a non-cloned, virulent stock of $SIV_{mac251/32H}$ all animals were analyzed for virus infection, by virus isolation (VI) from peripheral blood mononuclear cells (PBMC), quantitative proviral DNA load in PBMC, plasma vRNA load by sensitive quantitative competitive (qc)-RT PCR and presence of SIV-specific serum antibodies by ELISA. A significant protective effect was seen (p=0.003; Fisher's Exact Probability test) wherein 8 of 9 macaques given tenofovir *per rectum* either 15 minutes or 2 hours prior to virus challenge were protected from infection (n=6) or had modified virus outcomes (n=2) while 4 of 4 untreated macaques and 3 of 4 macaques given placebo gel were infected, as were 2 of 3 animals receiving tenofovir gel after challenge. Moreover, analysis of lymphoid tissues *post mortem* failed to reveal sequestration of SIV in the protected animals.

Colorectal explants from non-SIV challenged tenofovir treated macaques were resistant to infection *ex vivo*, whereas no inhibition was seen in explants from the small intestine.

Tissue-specific inhibition of infection was associated with the intracellular detection of tenofovir. In colo-rectal explants from 3 of 4 animals complete or nearly complete inhibition of virus replication was seen and in the other animal a high level of variability between replicate samples resulted in lower mean inhibition. In contrast, inhibition of virus replication was not seen in explants from the small intestine suggesting that tenofovir was, at least in part, acting on cells at the virus portal of entry.

Analysis of plasma tenofovir concentration at the time of virus challenge, 15 minutes after gel administration, revealed a strong positive association with protective efficacy. The lowest concentration of plasma tenofovir associated with protection was 119.9 ng/mL. Taking into account estimated plasma volume, protection was associated with as little as 0.11% of the total tenofovir applied; however, this is systemic exposure, rather than local exposure. Moreover, an effect upon plasma viremia was observed with as little as 0.06% of applied tenofovir detected in plasma at 15 minutes. In animals given tenofovir 2 hours prior to virus challenge, plasma tenofovir concentrations at the time of challenge ranged between below the 10ng/mL limit of detection to 23.3 ng/mL. These results suggest therefore that drug concentration peaks rapidly after rectal dosing. Interestingly ileum/jejunum tissue taken from dosed macaques remained susceptible to infection, and was confirmed by the lack of detectable drug in these tissues. This suggests that secondary distribution to this site is insignificant and supports the importance of comparing an oral, systemically-delivered dose to a topical, locally-delivered dose.

2.7.3 HEC Placebo Gel

HEC is the thickener in the placebo gel. The results of multiple animal studies have been consistent with the safety of this ingredient. A recently completed rectal study in a macaque model also appears to be consistent with the safety of this ingredient.

Toxicology

Up to 55 intravenous injections of HEC were given to dogs (dose and number not specified) without causing injury other than that typical of the other water-soluble cellulose ethers. [56] Only transitory changes in the blood picture and the deposition of the material on the intima of the blood vessels were noted. Groups of rats maintained for two years on diets containing HEC (n not specified, up to 5%) did not exhibit any adverse effects. HEC has also been administered to rats in single oral doses as high as 23,000 mg/kg without observed toxic effects (n not specified).

Intraperitoneal administration of unformulated HEC to pregnant mice in a 1% and 4% concentration caused an increase in resorptions, but no detectable increase in birth defects.[57] While no epidemiological studies of congenital anomalies in infants born to women exposed to HEC during pregnancy have been reported, the Teratogen Information System (TERIS) considers the magnitude of teratogenic risk to a child born after exposure during gestation to be none.[58]

CF-1 mice (n not specified) pretreated with medroxyprogesterone acetate were administered 0.02 mL of HEC gel vaginally, followed by a 0.01 mL inoculum of 10 intravaginal $dose_{50}$ units of HSV-2 0.3 minutes later.[40] On day 3, vaginal lavage was cultured on human foreskin fibroblasts, and mice were considered infected if a cytopathic effect was observed after 3 days of incubation. Control animals were treated similarly but were not administered the test article. Infection rate following pretreatment with HEC gel (90%) was not significantly different from pretreatment with PBS (80%) or from mice given no treatment (% not specified). HEC gel did not enhance susceptibility of mice to HSV-2 when administered 12 hours before vaginal challenge.[34]

A 10-day rabbit vaginal irritation study (10/arm, 2 arms, HEC gel vs. 0.9% saline control) found that the HEC gel was not irritating to the vaginal mucosa of rabbits when dosed daily for 10 days. One animal in the HEC gel group had an instance of vaginal redness (compared to four animals in the saline group), which did not persist and was not evident at the end of the study. Diarrhea, few feces, and soiling of the anogenital area were noted in that animal. Body weight changes were noted to be normal. In 9 of 10 animals, necropsy results were normal. Anogenital soiling was observed in the animal that exhibited erythema during the in-life phase of the study. Histopathological changes observed were similar to those seen in the control group and likely attributable to those that occur as a result of the repeated insertion of a catheter, rather than due to any effect of the test samples.

HEC gel was used as the placebo comparator in a recent rectal safety study of a combination microbicide in a macaque model.[59, 60] A third study arm received no product and served as a negative control. Rectal safety of the active product and HEC gel was evaluated following four daily applications of study products. Rectal flora, pH, and rectal lavage samples were assessed pre-and post-dosing and showed no evidence of toxicity in the macaques that received HEC gel. The infrequent evidence of epithelial sloughing and rare incidence of associated blood cells in rectal lavage samples was similar in the HEC placebo and no product arms of this study.

<u>Effectiveness</u>

The effect of the placebo gel on vaginal transmission of $SHIV_{162p3}$ (10^3 $TCID_{50}$) to rhesus monkeys was determined in two separate studies (n = 5, n = 3, respectively).[34] Macaques pretreated with medroxyprogesterone acetate were vaginally administered 1 mL of the HEC gel formulation 15 minutes prior to challenge with 0.5 mL $SHIV_{162p3}$. Investigators monitored total RNA load in the animal plasma for a total of 8 weeks by means of a standard quantitative RT-PCR. The first study utilized the HEC gel formulation at pH 6.5; the second study utilized a formulation at pH 4.4. In both studies, all monkeys were infected, as determined by the presence of viral RNA in circulating blood, regardless of the pH of the formulation.

2.8 Human Clinical Studies

2.8.1 Tenofovir Disoproxil Fumarate 300 mg Tablet

Pharmacokinetics

Tenofovir pharmacokinetics (PK) have been evaluated in healthy volunteers and HIV-1 infected individuals.[38] Tenofovir PK are similar between these populations and between males and females. Oral bioavailability of tenofovir from TDF in fasted patients is approximately 25%. *In vitro* binding of tenofovir to human plasma proteins is <0.7% and is independent of concentration over a range of 0.01-25 µg/mL. Following oral administration of one dose of TDF 300 mg to HIV-1 infected patients in the fasted state, maximum serum concentrations are achieved in 1.0 ± 0.4 hrs. Maximum serum concentration and AUC values are 296 ± 90 ng/mL and 2287 ± 685 ng•h/mL, respectively. Approximately 70-80% of the intravenous dose of tenofovir is recovered as unchanged drug in urine. Tenofovir is eliminated by glomerular filtration and active tubular secretion. Following a single oral dose of TDF 300 mg, the terminal elimination half-life of tenofovir is approximately 17 hours. The PK of individual doses of tenofovir are dose proportional over a TDF dose range of 75 to 600 mg and are not affected by repeated dosing.

Safety

Gilead Study 903, a randomized, double-blind trial conducted in the United States, Europe and South America, was designed to compare the efficacy and safety of a treatment regimen of TDF, 3TC and EFV to a regimen of d4T, 3TC and EFV in 600 ARV-naïve HIV-1 infected patients in a 144-week, double-blind phase. Patients who completed the 144-week double-blind phase on TDF were then eligible to roll over to the extension phase (weeks 144-480). In the double-blind phase, the most common (occurring in 2% or greater of tenofovir recipients) adverse events (AE) emerging after treatment with TDF plus EFV and 3TC in HIV-infection treatment naïve adults included whole body (headache, pain, fever, abdominal pain, back pain, asthenia), gastrointestinal (diarrhea, nausea, dyspepsia, vomiting), musculoskeletal (arthralgia, myalgia), nervous system (depression, insomnia, dizziness, anxiety), respiratory (pneumonia), and skin rash. The most frequent laboratory abnormalities were elevations in fasting cholesterol, creatine kinase, amylase, aspartate aminotransferase (AST) or alanine transaminase (ALT), hematuria, and decreased absolute neutrophil count. The frequency of all these events and laboratory abnormalities was similar or lower in the tenofovir treated group compared to the d4T-treated group.

Follow-up data from an interim 288-week analysis of patients who enrolled in the extension phase of the study have recently been reported.[61] Eighty-six patients (62% male, 70% white) initially randomized to the TDF arm continued treatment with TDF. No patient discontinued TDF due to renal events. Mean limb fat increased from 8.0 kg at week 96 to 8.8 kg at week 288. Thus, sustained TDF therapy was not associated with renal AEs or limb fat loss. Tenofovir is eliminated by the renal route, including tubular secretion. Thus, dose-interval adjustments are necessary for TDF in patients with significant renal impairment. TDF-induced nephrotoxicity has been reported in some

series,[62] especially in patients with other medical problems or pre-existing renal dysfunction, although observational prospective studies tend to accord with Gilead Study 903 in a finding of absence or low frequency of significant renal dysfunction;[63] when renal dysfunction occurs, it is generally predictable based on identifiable risk criteria.[64] One study that followed 27 HIV-infected children treated with TDF for 96 weeks found no evidence of impaired glomerular or tubular renal function.[65]

In Gilead Study 903 through 144 weeks, decreases from baseline in bone mineral density (BMD) were seen at the lumbar spine and hip in both arms of the study. At Week 144, there was a significantly greater mean percentage decrease from baseline in BMD at the lumbar spine in patients receiving TDF + 3TC + EFV (-2.2% ± 3.9) compared with patients receiving d4T + 3TC + EFV (-1.0% ± 4.6). Changes in BMD at the hip were similar between the two treatment groups (-2.8% ± 3.5 in the TDF group vs. -2.4% ± 4.5 in the d4T group). In both groups, the majority of the reduction in BMD occurred in the first 24–48 weeks of the study and this reduction was sustained through Week 144. Twenty-eight percent of TDF-treated patients vs. 21% of d4T treated patients lost at least 5% of BMD at the spine or 7% of BMD at the hip. Clinically relevant fractures (excluding fingers and toes) were reported in four patients in the TDF group and six patients in the d4T group. In addition, there were significant alterations in biochemical markers of bone metabolism (serum bone specific alkaline phosphatase, serum osteocalcin, serum C-telopeptide, and urinary N-telopeptide) in the TDF group relative to the d4T group, suggesting increased bone turnover. Serum parathyroid hormone levels and 1.25 Vitamin D levels were also higher in the TDF group. Except for bone specific alkaline phosphatase, these changes resulted in values that remained within normal range. Importantly, changes in BMD at the lumbar spine and hip noted in the first 48 weeks of the study were non-progressive through 288 weeks in the extension phase. However, the effects of TDF-associated changes in BMD and biochemical markers on long-term (>144 weeks) bone health and the risk of future fracture are unknown.

Peterson, et al. evaluated the safety of TDF 300 mg daily versus placebo for prevention of HIV-1 infection in women in a Phase 2 double-blind study conducted at 3 sites in West Africa.[66] The study closed prematurely resulting in insufficient power to evaluate efficacy. In the primary safety analysis, with 428 person-years (p-y) of follow up, there was no significant difference in the rate of safety endpoints (defined as grade 2 or higher serum creatinine, grade 3 or 4 transaminase elevation, or grade 3 or 4 phosphate abnormality). Among the 368 participants on TDF, none had grade 3 or 4 transaminase elevation or grade 2 or higher creatinine. One TDF recipient had self-limited grade 3 phosphate. Additional safety information from clinical studies on the TDF 300 mg tablet is available in the package insert at: http://www.gilead.com/pdf/viread_pi.pdf.

Pregnancy Outcomes

The Antiretroviral Pregnancy Registry is intended to provide an early signal of any major teratogenic effect associated with a prenatal exposure to the products monitored through the Registry. The Registry is a voluntary prospective, exposure-registration,

observational study designed to collect and evaluate data on the outcomes of pregnancy exposures to ARV products. Data through 07/31/07 show 6 defects among 380 first trimester TDF exposures.[67] This rate (1.6%) is not elevated compared to 4/263 (1.5%) after second/third trimester exposure, the 2.67% background rate of defects reported by the Centers for Disease Control Metropolitan Atlanta Congenital Defect Program[68], or the generally accepted background rate for birth defects in the US population (approximately 3 – 4%).

Effectiveness as Pre-Exposure Prophylaxis (PrEP)

In the Peterson study referenced above, HIV seroconversion was observed in 2/427 participants in the TDF group (0.86 per 100 p-y) and 6/432 participants in the placebo group (2.48 per 100 p-y), yielding a rate ratio of 0.35 (95% CI, 0.03-1.93). Because this study was closed prematurely, the number of observed HIV infections was lower than planned; the rates of HIV seroconversion in the two groups were not significantly different. Standard genotypic resistance testing of one of the two participants who seroconverted on TDF revealed no drug resistance mutations.[66]

2.8.2 Tenofovir 1% Gel

RMP-02/MTN-006 and MTN-007 will be the first rectal safety studies of vaginally formulated tenofovir 1% gel. However, a broad range of reproductive tract studies have been completed, or are ongoing, and these data are summarized below.

Pharmacokinetics

A Phase 1 Safety and Acceptability Study of the Vaginal Microbicide Agent PMPA Gel, also known as HPTN 050, is a recently completed study of tenofovir vaginal gel with published data.[72] Eighty-four (60 HIV negative and 24 HIV positive) women applied either 0.3% or tenofovir 1% gel once or twice daily for 14 days. Systemic absorption (using an earlier and less sensitive detection kit) was limited, showing maximum serum levels 3.1-25.8 ng/mL.

Safety

In HPTN 050, the tenofovir 1% gel formulation was well tolerated in both HIV-uninfected and -infected women.[69] Further, 94% of female participants and 81% of male participants indicated they would definitely or probably use tenofovir gel in the future. While a number of participants (92%) reported some type of AE, the majority of them were mild (87%) and limited to pruritus (n = 18), erythema (n = 14), petechiae/ecchymosis (n = 14), vaginal discharge (n = 13), and burning (n = 10). Only four severe AEs were reported, but, of these, only one (lower abdominal pain) was thought to be product-related. Product concentration, sexual activity and HIV status were not associated with a specific AE pattern. No clinically significant systemic toxicity was observed. No serious adverse events (SAEs) were reported.

Of 76 participants in the HPTN 050 study who had bacterial vaginosis (BV) evaluation (by using Nugent's score criteria) at both enrollment and Day 14, 30 women had asymptomatic BV at baseline and 15 of them became BV negative after 14 days of

tenofovir gel use, while one out of 46 women without BV at baseline had BV detected at 14 days. Overall, 40% of the women had asymptomatic BV at baseline compared to 21% of the women after fourteen days of tenofovir gel use ($p = 0.0005$), suggesting that the gel did not increase women's risk of developing BV.

In a male tolerance study (CONRAD A04-099/IND 73,382), tenofovir 1% gel was well tolerated in men following seven days of once daily penile exposure. There were few genital findings observed after product use and all findings were classified as mild, small in size and requiring no treatment. Reported symptoms were mild, of short duration and resolved by the final visit. There were no noticeable differences between signs and symptoms of genital irritation in the circumcised compared to uncircumcised group.[70]

A Phase 2 study of tenofovir 1% gel (HPTN 059) has completed follow up. This study assessed safety and acceptability of, and adherence to a regimen of tenofovir gel for vaginal use in HIV-uninfected women versus a placebo gel. Exploratory objectives included measurement of vaginal flora characteristics, assessment of the effects of gel on genital cytokine and chemokine expression, and the evaluation of cytokine and chemokine expression to correlate expression with evidence of inflammation, epithelial disruption and genital symptoms. The study was a four-arm, three-site, randomized, controlled trial comparing gel used once daily and gel used prior to intercourse, to placebo gel, with 6 months gel exposure and follow-up. The study was conducted among 200 women in Pune, India; Birmingham, Alabama, USA; and New York, New York, USA. Participants were sexually active, HIV-uninfected women between ages 18 and 50, but not menopausal or post menopausal. Participants had six months of study gel exposure and six months of follow-up. They were randomized to either once daily or coitally dependent group, and received either tenofovir or placebo gel. Participants received single use unit dose tubes and single use applicators.

No statistically significant differences were seen between those receiving active and placebo gels in complete blood count, liver function tests, or renal function tests. Among those using a study gel daily, no participants had pelvic exam findings involving generalized erythema or severe edema or deep epithelial disruption at any follow-up visit during the study. At the Week 24 Visit, no participants had exam findings suggestive of vaginitis, cervicitis, superficial disruption, disrupted blood vessels, or intermenstrual bleeding. Adherence to study gel was high, and was supported by PK data, using an enhanced detection kit. 79% of women reporting gel use in past 12 hours had low but detectable plasma tenofovir supporting self-reported adherence data. Daily and coital use was highly acceptable to women. These data suggest a favorable safety and acceptability profile of tenofovir gel, and support routine monitoring for genital findings among women without genital symptoms at six month intervals.[71]

Resistance

In HPTN 050, no new resistance mutations evolved in plasma or cervicovaginal lavage after 14 days of tenofovir gel use, but 3 women had plasma mutations associated with low level tenofovir resistance identified at both Days 0 and 14 (M41L, L210M, ±T215I/Y). [69]

Other Studies of Tenofovir for HIV Prevention

Several other studies of the safety and/or effectiveness of tenofovir as an HIV prevention strategy are summarized below. These include studies in Table 5.

Table 5: Other Studies of Tenofovir 1% Gel

Location	Sponsor	Population	Design
USA, Dominican Republic	CONRAD A04-095/IND 73,382	Sexually abstinent women	PK study; single dose and 14-day once or twice-daily.
South Africa	CAPRISA	Sexually active women	Phase 2B, two-arm, randomized placebo controlled, coitally dependent
South Africa, Uganda, USA	DAIDS/MTN-001/IND 55,690	Sexually active women	Phase 2 Adherence and Pharmacokinetics Study of Oral and Vaginal Preparations of Tenofovir
USA	DAIDS/MTN-002/IND 55,690	Healthy term gravidas	Phase 1 Study of Maternal Single-Dose Pharmacokinetics and Placental Transfer
USA	DAIDS IPCP RMP-02/MTN-006/IND 73,382	Sexually abstinent (for active phases of study and for 5 days following biopsy collection) women and men	Phase 1 Rectal PK and Acceptability
USA	DAIDS/MTN-007/IND 73,382	Receptive anal intercourse (RAI) abstinent (for duration of study) women and men	Phase 1 Rectal Safety and Acceptability

Studies examining the safety and/or effectiveness of oral formulations of tenofovir as a prevention strategy are summarized in Table 6 below.

Table 6: PrEP Studies

Location	Sponsor	Population	PrEP Strategy
**Phase II**			
West Africa (Ghana, Nigeria, Cameroon)	Family Health International	936 high-risk women	TDF
United States	CDC	400 men who have sex with men	TDF
**Phase III**			
Thailand	CDC	2,000 injection drug users (~20% women)	TDF
Botswana	CDC	1,200 men and women	Emtricitabine (FTC)/TDF
Peru, Ecuador, Brazil, Thailand, South Africa, United States	NIH<br><br>(iPrEx Study, IND 71,859)	1,400 men who have sex with men (potential expanded sample size of 3,500)	FTC/TDF
Africa	Family Health International	3,800 high-risk women	FTC/TDF
Africa	University of Washington, Gates Foundation	3,900 HIV-1 seronegative partners within HIV-1 discordant couples	FTC/TDF<br>TDF

2.8.3 HEC Placebo Gel

Unformulated hydroxyethylcellulose is known to be a non-irritating substance in humans (skin sensitization is unusual), with doses less than 2 g/kg by ingestion not expected to be toxic[72]. No inhalation studies have been conducted, but exposure of humans to the dust in manufacturing operations over many years has not led to any known adverse effects.

Safety-Vaginal Administration

The hydroxyethylcellulose placebo formulation was developed and adopted for use in the HPTN 035 microbicide study, the Phase II/IIb Safety and Effectiveness Study of the Vaginal Microbicides Buffer Gel and 0.5% PRO2000/5 Gel (P) for the Prevention of HIV Infection in Women.

A randomized, closed label, Phase I study of daily vaginal HEC placebo exposure was conducted in 2003.[73] In this trial, 30 women were randomized to twice-daily vaginal applications of 3.5 mL of the universal (HEC) placebo or polystyrene sulfonate (PSS) vehicle. The primary objective of this study was to assess and compare the effects of the test articles on symptoms and signs of irritation of the external genitalia, cervix, and vagina as seen on naked eye exam after 7 and 14 days of use including disruption of the epithelium and blood vessels as seen on colposcopy after 14 days of use. Secondary objectives included: an assessment and comparison of differences in vaginal health by evaluating the results of wet mounts, pH, and Gram-stained vaginal smears (Nugent score and neutrophil counts) after 7 and 14 days of use and vaginal cultures after 14 days of use; and an assessment of acceptability of the study products after 14 days of use among participants.

Results of this trial indicated that both gels appear safe for vaginal use twice a day for 14 days in sexually abstinent women. Two out of 14 women (14.3%) randomized to the HEC group reported at least one symptom of mild severity of genital irritation, which included genital burning, soreness and pelvic pain. A lower proportion of women in the HEC group experienced any evidence (signs and/or symptoms) of genital irritation. Three out of 14 women in the HEC group (21.4%) had colposcopic findings that included erythema, petechiae and peeling.[73] No deep genital disruption was observed in either product group. Minimal changes in wet mounts, pH, Nugent scores, neutrophils, and vaginal flora were observed in both product groups.

Safety-Rectal Administration

A 2-period crossover study of commercially available lubricant gels, by Fuchs and colleagues[74] demonstrated that osmolar properties affected epithelial denudation and product absorption. The gels were made into iso-osmolar and hyperosmolar mixtures and compounded with a radio-isotope label to address product absorption. Ten healthy male subjects (all MSMs) including 8 seropositive and 2 seronegative men were recruited for the study. All subjects received a 10 mL rectal dose of hyperosmolar ID Glide (Westridge Laboratories; 3429 mOsm/kg [pH 4.79]) and a 10 mL rectal dose of an iso-osmolar preparation of FemGlide (Cooper Surgical)/ID Glide combination gel (283

mOsm/kg [pH 6.77]). Sigmoidoscopy was performed within 1.5 hours of dosing, with cytobrush sampling at 10 cm and cytobrush and biopsy sampling at 12.5 cm (referred to as 10 cm in results), 40 cm, and 42.5 cm (referred to as 40 cm in results). A paired comparison showed a statistically significant difference in gel concentration between the iso-osmolar and hyperosmolar products at 10 cm. The median isotope concentration in the iso-osmolar gel arm was greater at 10 cm than at 40 cm, whereas there was no significant difference at 10 cm and 40 cm in the hyperosmolar gel arm indicating the hyperosmolar gel induced a luminal influx of liquids. The hyperosmolar gel resulted in Grade 3 denudation of the rectal epithelium at 10 cm while the iso-osmolar gel showed no histological damage. No difference in epithelial structure was observed at 40 cm with either gel. Overall, the results showed epithelial injury is greatest at the site of initial and most concentrated gel exposure which causes mucosal fluid secretion and a dilution of intraluminal gel concentration. While no conclusion could be drawn regarding the timing of repair of epithelial changes in this study, the authors noted that other researchers have reported observations of repair as soon as 2 h after injury and a resumption to baseline histology at 8 hours after insult, prompting the encouragement of future investigation into time-related response.

2.9 Study Hypotheses and Rationale

2.9.1 Study Hypotheses

- Vaginally-formulated tenofovir 1% topical gel when applied rectally will be safe using a combination of clinical and laboratory markers including assays specifically designed to measure mucosal toxicity

- Tenofovir will be detectable at different concentrations in the various anatomic compartments sampled for pharmacokinetics following single and 7-day topical exposures

- Exposure to tenofovir 1% gel will demonstrate prevention of *ex vivo* HIV-1 challenge using *in vivo* drug-exposed tissue as compared to baseline tissue samples

- Orally delivered, single dose, 300 mg tenofovir disoproxil fumarate tablets will have similar safety profiles using routine blood safety indices as have been established in other trials and will show no mucosal safety concerns

- The oral dose will have different multi-compartment concentration kinetics than the topical tenofovir and will also demonstrate preliminary (*ex vivo)* prevention using the explant infectivity assay

- Vaginally formulated tenofovir 1% topical gel applied rectally will be acceptable to participants, as indicated by a score in the upper one third of the 10-point Likert scale on intentionality to use in the product in the future

2.9.2 Rationale

The rationale for conducting phase 1 safety assessment of vaginal microbicides (VM) in the rectal compartment is based on increasing recognition that women in the developed[4, 75] and developing world[76] engage in anal intercourse. It is therefore assumed that VM, once available, will also be used rectally and it will be important to know whether a safe VM will also be safe in the rectal compartment.

Developing Safety Standards for Rectally-Administered Microbicides

There are limited preclinical data evaluating microbicide safety in the rectal compartment and the majority of data focus on the spermicidal gel, Nonoxynol-9 (N-9). The N-9 data have provided important insights concerning the intestinal mucosal response to microbicide induced injury. Phillips et al. demonstrated that rectal application of N-9 in mice resulted in rapid exfoliation of intestinal epithelium within 10 minutes of product exposure. The changes were transient and histological examination of the intestinal biopsy samples collected at 1 hour post N-9 exposure appeared normal. The study also demonstrated an N-9 dose dependent increase in murine susceptibility to anorectal herpes simplex infection.[77] Similar dramatic intestinal exfoliation has been documented in macaques rectally exposed to N-9.[78] A human rectal safety study by Tabet et al. described mild rectal histological changes in participants receiving up to 6 weeks of N-9 or placebo gel[19]. In contrast, marked epithelial exfoliation was seen after brief exposure to N-9 in studies by Phillips et al using rectal lavage and histology as endpoints[11, 12]. These contradictory results probably reflect the timing of sample collection. Epithelial reconstitution can occur within 1-8 hours after exposure to N-9.[11, 77] In the Tabet study samples were collected up to 12 hours after N-9 exposure but after only 15 minutes in the Phillips study. The implication of these early studies is that rectal safety should be assessed after acute (within 1 hour) and chronic (at least 7 days) product exposure.

Histology and/or rectal lavage studies can be helpful in documenting severe microbicide associated mucosal changes. However, there is increasing concern that repeated mucosal exposure to vaginal or rectal microbicides could induce subtle immunological changes in the vaginal or rectal mucosa that might increase the risk of HIV transmission. Increased expression of mucosal inflammatory cytokines could lead to recruitment of target cells to the local mucosa and these changes would probably not be detected using conventional histological techniques. As a consequence, it will be necessary to develop immunological biomarkers of microbicide safety. A first step in this process is the characterization of the biological variability of putative mucosal safety biomarkers. Markers that demonstrate extreme variability will be unhelpful as safety biomarkers in microbicide studies. HPTN 056 is a recently published study investigating the biological variability of safety biomarkers in the colorectal intestinal mucosa[18]. Intestinal biopsies were collected from 16 participants on three occasions over a 4 week period in the absence of any microbicide exposure. Tissue was collected at 15 and 30 cm from the anal margin and evaluated for biological variability of a broad range of parameters including histology, mucosal cytokine gene expression, rectal immunoglobulins, and mucosal T cell phenotype. The study demonstrated that tissue

from both sites was essentially equivalent and that the most stable parameters included mucosal cytokine expression and T cell phenotype. Both of these parameters could have utility in the evaluation of potential microbicide toxicity within phase 1 rectal safety studies.

The first microbicide product to undergo Phase 1 rectal safety assessment with this broader range of safety biomarkers is the non-nucleoside reverse transcriptase inhibitor UC781(RMP-01).[13] The study has recently finished subject participation, unblinded data and analysis is ongoing. In the study, which was conducted at UCLA under the auspices of the NIH IPCP U19 program, participants were screened to exclude anorectal STIs and then baseline mucosal samples were collected. After a one-two week period to allow mucosal healing, the participants received a single dose of UC781. Within 30 minutes of microbicide exposure, the participants underwent mucosal assessment to assess acute mucosal responses to UC781. After a second recovery period, seven daily doses of UC781 were administered followed by final mucosal assessment. The range of safety parameters evaluated in the study includes intestinal histology, rectal lavage for epithelial exfoliation, intestinal cytokine gene expression, mucosal mononuclear T cell phenotype, rectal immunoglobulins, and fecal calprotectin. A unique feature of this study was the evaluation of intestinal tissue explants, exposed to UC781 *in vivo*, to resist HIV infection *in vitro.*[79] This design feature allows for preliminary assessment of microbicide efficacy as well as safety before potentially proceeding to much larger clinical effectiveness studies. Preliminary, unblinded results demonstrate no Grade 3, 4 or procedure-related AEs, 8 Grade 2 AEs (4 in one subject) and no significant changes from baseline values following single or seven day exposure.

2.10 Justification of Dosing

Tenofovir 1% Vaginal Gel

Choice of the tenofovir 1% vaginal gel concentration for RMP-02/MTN-006 is based on both animal and clinical evidence suggesting an appropriate safety profile and potency. The daily dose volume of the 1% tenofovir vaginal gel to be applied rectally is 4 mL (which equals 4.4 grams of gel, in weight). Each gram of gel contains 10 mg of tenofovir resulting in 44 mg of tenofovir delivered in each daily 4 mL application. The proposed human daily dose for a person who weighs 70 kg is 0.607 mg/kg/day.

Animal and human studies have demonstrated minimal vaginal irritation at this concentration. A rabbit vaginal irritation test identified tenofovir 1% gel as being histopathologically identical to sham or control treatment, while on a qualitative basis 3% gel was more irritating to vaginal epithelia.[46] The tolerability of the 1% gel was confirmed in the HPTN 050 Phase 1 study, the Phase 1 dose ranging study of tenofovir gel (0.3% once daily, then 1.0% once daily, then 0.3% twice daily followed by 1% twice daily). In this study, of the two doses and frequencies studied in the dose finding cohort, the 1% gel applied intravaginally twice daily for 14 days was well tolerated and was identified as the highest practical dose and frequency for further study in subsequent cohorts.

The second line of evidence is from vaginal transmission inhibition studies performed in non-human primates[46]. Six separate studies provided evidence for efficacy of the gel over a range of tenofovir concentrations of 1% to 10%. Although the total data are limited and a powered statistical determination as to the efficacy of tenofovir 1% gel versus 0.3% and 10% cannot be made, empirical examination of the efficacy data identifies tenofovir 1% gel as the lowest efficacious concentration tested when given within two hours of infection.

Finally, limited vaginal PK tenofovir data in primates demonstrate that tenofovir gel is broadly distributed in vaginal tissues following vaginal application and can penetrate to epithelial tissues.[80] Comparison of the predicted cervicovaginal concentrations of tenofovir gel delivered to those achieved systemically at the standard treatment dose of 300 mg TDF, and tenofovir's characteristic prolonged intracellular half-life (diphosphate form, nine to 50 hours depending upon cell type), suggest that an initial and potentially durational barrier to HIV transmission may be possible. In terms of weighing potential risks and benefits, the tenofovir 1% gel minimizes the potential risks of vaginal epithelial toxicity while providing the potential benefit of delivering sufficient tenofovir to achieve an initial and possibly durational barrier to infection.

Table 7: Range of Human Exposure With Tenofovir 1% Gel[81]

Tenofovir 1% gel route of administration (vaginally formulated)	Analyte	AUC in blood plasma or PBMCs (h*ng/mL)	C_{max} in blood plasma or PBMCs (ng/mL)	C_{24h} in blood plasma or PBMCs (ng/mL)	AUC in tissues *(h*ng/g)	C_{max} in tissue* (ng/g)	C_{24h} in tissue *(ng/g)
**Vaginal**	tenofovir	BLQ-152	BLQ-20	BLQ-5	1,404,172	449,719	10,000
	tenofovir-DP	NK	NK	NK	NK	NK	NK
**Rectal**	tenofovir	NK	NK	NK	NK	NK	NK
	tenofovir-DP	NK	NK	NK	NK	NK	NK

NK=not known, *= data obtained as a composite of individual samples taken from multiple women over various times post-dose, DP=diphosphate

Tenofovir Disoproxil Fumarate 300 mg Tablets

Choice of the 300 mg strength of the TDF tablet is based upon practical and scientific considerations. The TDF 300 mg tablet, or Viread®, is the medication US FDA approved for the indication of treatment of HIV-1 infection. More than 12,000 people have been treated with TDF alone or in combination with other antiretroviral medications for periods of 28 days to 215 weeks in Phase 1–3 clinical trials and expanded access studies. A total of 1,544 patients have received TDF 300 mg once daily in Phase 1–3 clinical trials; over 11,000 people have received TDF in expanded access studies. A significant body of safety data has been accumulated for daily use of the TDF 300 mg tablet. In addition, data on tenofovir PK and anti-viral activity in humans suggest a reasonable expectation of effectiveness as a prevention strategy.

2.11 Justification of Sampling Time Points

The intent of this trial is to characterize the local and systemic effects of single and multiple exposures to tenofovir 1% gel applied rectally. Evaluations will include both local mucosal tissue and immunotoxicity impact, *ex vivo* effect of inhibiting HIV-1 replication and tenofovir concentrations in multiple compartments over the 2 weeks following single and multiple exposures. There will be a single, post-7 day exposure assessment of these as well. This latter time point will not accurately characterize the T½ of tenofovir or tenofovir diphosphate, but will be used to assess safety indices following repeat exposures and quantify immunotoxicity indices, ex vivo inhibition using explants and tenofovir concentrations.

A novel and pivotal component of this trial's design is the ability, within subjects, to compare the immunotoxicity indices described above from a topical exposure to tenofovir 1% gel with those from a single oral dose of tenofovir 300mg. This will be the first side-by-side comparison of topical and oral exposure in the rectal compartment.

PK time points will consist of a 4-point extracellular and intracellular plasma and rectal secretion sampling strategy (for women, cervicovaginal secretions will also be collected) within the first 24 hours of oral and single and multiple dose topical exposure for all subjects (Visit 3, Visit 7, and Visit 11). These samples will be drawn at 0h (pre-dose), 0.5 h, 2 h and 4 h after the dose. A 24 h sample will also be obtained in 50% of subjects (Group A). To complement and extend our observation of plasma PK, additional plasma

timepoints will be obtained with the 5-6 compartment PK studies described below over the 2 weeks following single oral and single topical exposure.

After a single dose to minimize risk and maximize insights into compartmental drug concentrations and *ex vivo* efficacy correlations over the subsequent two weeks, participants will be divided into two groups. Following Visit 2 (when subjects are randomized to tenofovir gel or placebo 2:1), subjects will also randomized into Group A or Group B with equal numbers of placebo and active drug in each group. While all participants will be seen on Day 1 to complete the 24-hour plasma PK timepoint desired, those in Group A will be seen for complete blood, fluid, and tissue sampling with flexible sigmoidoscopy on Day 1 and Day 7 following the single oral dose of TDF 300mg and single rectally applied dose of tenofovir 1% gel or placebo gel. Participants in Group B will be seen for the 24-hour plasma PK only on Day 1 and then return on Day 4 and Day 10 following the single dose administration of the oral or rectal study products for the full set of sample collections, including flexible sigmoidoscopy.

Ex vivo explant infectivity and inhibition experiments will be conducted at each timepoint. All participants will have baseline explant studies done to ensure infectivity of tissue (set-up in quadruplicate to minimize technical error and assay variability) as well as studies after 0.5 h oral and topical exposure and then again following 7-day exposure. Members of Group A will have these done on days 1 and 7 while Group B will be done on days 4 and 10. This pattern will be the same for all mucosal immunotoxicity panels described above. These groups will provide frequent and close time points as described in Table 8 below.

Table 8: Use and Distribution of Biopsies in 2-Week Follow-Up After Single Topical and Oral Exposure

N=18	30 min	Day 1	Day 4	Day 7	Day 10
**Group A**	**x**	**x**		**x**	
**Group B**	**x**		**x**		**x**
Explant	4	4	4	4	4
Flow Cytometry	4	4	4	0	0
Histology	1	1	1	1	1
PK tissue	2	2	2	2	2
PK MMC	6	6	6	6	6
**TOTAL**	**~17**	**~17**	**~17**	**~13**	**~13**

Explants: here with only high dose viral titer (10^4) in quadruplicate
Flow cytometry: done at 30 minutes and days 1 and 4 (to compare with UC781 (30 min) and to start to see longer effects, if any.
PK: ~8 biopsies dedicated at each sampling point; MMCs will have TruCount® run to know CD4 populations

Table 9: Schedule of Study Endpoints

Visit		Types of Specimens Taken	Safety and Immunotoxicity Endpoints: Blood: CBC, ALT, AST, Creat., Phos.	Safety and Immunotoxicity Endpoints: Blood: RPR, HIV	Vaginal Swab (pH, gram stain)	Rectal Swab STI	Rectal Swab Microflora	Rectal Fluid From Sponge Rectal Cytokines	Stool from Prep Enema Calprotectin	Endoscopic Lavage Epithelial Sloughing	Rectal Biopsies (~1) Histology	Rectal Biopsies CD4 Cells (MMC Phenotyping, ~4 Biopsies)	Tenofovir Concentration Endpoints: Blood Plasma and PBMC	Tenofovir Concentration Endpoints: Vaginal Fluid from Sponge	Tenofovir Concentration Endpoints: Rectal Fluid from Sponge	Tenofovir Concentration Endpoints: Rectal Biopsies (~8) Whole Tissue and MMC	Preliminary Efficacy Endpoint: Rectal Biopsies (~4) HIV Infectability in Explants
Visit 1: Screening			✓**, □	✓	✓	✓											
Visit 2: Enrollment/Baseline		Blood, fluids from sponges, stool, endoscopic lavage, biopsies (~17)***	✓***		✓			✓	✓	✓	✓	✓		✓	✓	✓	✓
Visit 3: Single **Oral Dose**	Before dosing	Blood, fluids from sponges	✓***		✓		✓	✓	✓				✓	✓ baseline	✓ baseline		
	After dosing	Blood, fluids from sponges, stool, endoscopic lavage, biopsies (~17)						✓		✓	✓	✓	✓ (also 2h and 4h)	✓ (also 2h and 4h)	✓ (also 2h and 4h)	✓	✓
Visit 4: 24 Hours After Single Oral Dose		Blood, fluids from sponges											✓	✓	✓		
Visit 5A/B: (Day 1-3, or 4-6)		Blood, fluids from sponges, stool, endoscopic lavage, biopsies (~17)			✓			✓	✓	✓	✓	✓	✓	✓	✓	✓*	✓
Visit 6A/B: (Day 7-9, or 10-12)		Blood, fluids from sponges, stool, endoscopic lavage, biopsies (~13)			✓			✓	✓	✓	✓		✓	✓	✓	✓	✓
Visit 7: Single **Topical Dose**	Before dosing	Blood, fluids from sponges, stool	✓***		✓		✓	✓	✓				✓	✓	✓		
	After dosing	Blood, fluids from sponges, stool, endoscopic lavage, biopsies (~17)						✓		✓	✓	✓	✓ (also 2h and 4h)	✓ (also 2h and 4h)	✓ (also 2h and 4h)	✓	✓
Visit 8: 24 Hours After Single Topical Dose		Blood, fluids from sponges											✓	✓	✓		
Visit 9A/B: (Day 1-3, or 4-6)		Blood, fluids from sponges, stool, endoscopic lavage, biopsies (~17)			✓			✓	✓	✓	✓	✓	✓	✓	✓	✓*	✓
Visit 10A/B: (Day 7-9, or 10-12)		Blood, fluids from sponges, stool, endoscopic lavage, biopsies (~13)			✓			✓	✓	✓	✓		✓	✓	✓	✓	✓
Visit 11: Begin 7-day Topical		Blood, fluids from sponges ***											✓	✓	✓		
Visit 12: Product Use End Visit	Before dosing	Blood, swabs, fluids from sponges, stool, endoscopic lavage, biopsies (~17)	✓***	✓	✓		✓	✓	✓				✓	✓	✓		
	After dosing							✓		✓	✓	✓	✓ (also 2h and 4h)	✓ (also 2h and 4h)	✓ (also 2h and 4h)	✓	✓
Visit 13: Final Visit (24-hour, Post-exposure Sampling)		Blood, fluids from sponges					✓	✓					✓	✓	✓		

□ Visit 1: Urine also collected for qualitative hCG (females only), dipstick urinalysis (UA) for protein, glucose, nitrates and leukocyte esterase, and NAAT for GC/CT
* Visits 5 and 9: No flow will be done at these visits.
** Visit 1: Blood also collected for HBsAg and HSV-1 and -2 serology
*** Visits 2,3, & 7,11: Urine also collected for qualitative hCG (females only)

3 OBJECTIVES

3.1 Primary Objective: Safety

- To evaluate the systemic safety of 1% vaginally-formulated tenofovir gel, applied rectally

3.2 Secondary Objectives: Immunotoxicity, Pharmacokinetics, and Acceptability

- To evaluate the immunotoxicity of 1% vaginally formulated tenofovir gel applied rectally

- To compare systemic and compartment pharmacokinetics (PK) among oral TDF, rectally applied tenofovir 1% gel, and placebo gel

- To evaluate the acceptability of tenofovir 1% gel when applied rectally

3.3 Exploratory Objectives:

- To assess the preliminary efficacy *(ex vivo)* of rectally applied tenofovir 1% gel

4 STUDY DESIGN

4.1 Identification of Study Design

RMP-02/MTN-006 will be a Phase 1, randomized, two-site, partially-blinded, placebo-controlled, three-period safety, acceptability, and PK study of Tenofovir 1% Gel applied rectally compared to single oral 300 mg Tenofovir tablets. Please see Table 10 below for detailed description of the timeline of endpoint assessments.

4.2 Summary of Major Endpoints

- Grade 2 or higher clinical or laboratory adverse events as defined by the Division of AIDS Table for Grading the Severity of Adult and Pediatric Adverse Events, Version 1.0, Dec 2004 and Addenda 1 and 3 (Female Genital and Rectal Grading Tables for Use in Microbicide Studies) to this table.

- Mucosal assays
    - Fecal calprotectin
    - Microflora (see Appendix V)
    - Rectal cytokines (secreted)
    - Rectal epithelial sloughing

- Rectal histology
- Rectal CD4 cells phenotype/activation

- Tenofovir concentrations
  - Plasma (tenofovir)
  - PBMC (intracellular)
  - Rectal fluid
  - Vaginal fluid
  - Rectal mucosal tissue homogenates
  - Rectal mucosal mononuclear cells
- Tenofovir diphosphate concentrations
  - PBMC
  - Rectal mucosal tissue homogenates
  - Rectal mucosal mononuclear cells
- Changes in HIV-1 p-24 levels in colorectal explant supernatant

- The proportion of participants who at their Final Clinic Visit (Visit 13) report that they would be very likely to use the candidate microbicide during receptive anal intercourse.

Table 10: Summary of Biological Samples Collected In Study

Type of Sample	Assay	Details	Endpoint addressed
**Blood**	CBC		Safety
	Metabolic panel (MP)	ALT, AST, creatinine, and phosphate	
	STIs	HIV-1, RPR, HSV-1 and 2 and HBsAg	
	Tenofovir concentration	Plasma and PBMC drug concentration	Drug concentration
**Rectal swab**	STIs	GC, Chlamydia	Safety
	Microflora	See Appendix V for particular species	
**Vaginal swab**	BV	pH and Gram stain	Safety
**Rectal sponge sample**	Rectal cytokines	Quantified by Luminex®	Safety
	Tenofovir concentration	In rectal fluid, eluted from sponge	Drug concentration
**Vaginal sponge**	Tenofovir concentration	In vaginal fluid , eluted from sponge	Drug concentration
**Stool from preparatory enema**	Calprotectin	From stool in effluent fluid acquired from preparatory enema (Company outsource - Genova Diagnostics)	Safety
**Rectal endoscopic lavage**	Rectal epithelial sloughing	From rectal lavage fluid acquired during sigmoidoscopic procedure	Safety
**Rectal biopsies**<br><br>**(All taken at 10-15 cm – max of 17 biopsies)**	Rectal histology	biopsy	Safety
	Rectal CD4 cells	Flow cytometric characterization of CD4+ T lymphocytes' phenotype, activation status and co-receptor expression	
	Rectal whole tissue Tenofovir concentration	biopsies	Drug concentration
	Rectal tissue intracellular Tenofovir concentration (MMCs)	biopsies	
	HIV infectability of colorectal tissue explant cultures	biopsies	Preliminary efficacy
**Urine**	Routine urinalysis (UA)	Check glucose, protein	Safety

4.3 Description of Study Population

The study population will include 18 evaluable generally healthy participants (men and women) age 18 and over who are HIV-uninfected and sexually abstinent as described in Section 5.2. Female participants will not be pregnant, breastfeeding, or at risk for pregnancy.

4.4 Time to Complete Enrollment

The approximate time to complete enrollment is expected to be approximately 8.5 months. The time of total study duration is expected to be a minimum of 11 months including the study follow-up period.

4.5 Study Groups

All 18 participants will be assigned to complete each study period (oral and rectal). Two levels of randomization will be completed for each subject after Visit 2 (enrollment/baseline) and before Visit 3 (single oral dose). One level involves randomization into Group A or B for specimen sampling time points and will be completed in a 1:1 ratio. The second level of randomization involves drug or placebo assignment to topical gel (rectal phases only) and will be completed as 2:1 drug to placebo ratio.

PK as well as mucosal immune responses will be measured at baseline for all participants, and following single oral, single topical, and 7-day exposure. This will permit each participant to not only serve as their own control, but will also enable within-group and between-group analyses.

4.6 Sequence and Duration of Trial Periods

The total duration of participation from the Enrollment Visit to the Follow-Up Safety Phone Call is 3.5 months, including three study periods (a single oral dose, a single rectal dose, and one 7-day rectal exposure dose), 2 two-week sampling periods, and 2 one to two-week resting periods. Visits may be completed within specified windows around target dates. Detailed information regarding visit windows will be thoroughly described in the RMP-02/MTN-006 Study Specific Procedures Manual.

4.7 Expected Duration of Participation

The expected duration of participation for individual enrolled participants is 3.5 months from the Enrollment Visit to the Follow-Up Safety Phone Call.

4.8 Sites

Two study sites are planned for this trial:

- UCLA, Los Angeles, USA
- Pitt CRS, Pittsburgh, USA

5 STUDY POPULATION

5.1 Selection of the Study Population

The inclusion and exclusion criteria outlined in Sections 5.2 and 5.3 will be utilized to ensure the appropriate selection of study participants for RMP-02/MTN-006. The projected length of accrual for 18 study participants is 8.5 calendar months.

5.1.1 Recruitment

Participants will be recruited from a variety of sources, using the following key strategies:

- Clinician-patient referrals
- Use of existing "study registries" that contain the names and phone numbers of individuals who have given informed consent to be reached for future studies for which they may be eligible
- Participant referrals (participants refer their friends or partners who may meet eligibility criteria)
- Passive self-referral: interested individuals see a study poster or brochure advertising the study and call the study site directly

Study staff will meet as needed to discuss current recruitment status, targets, and strategies. Staff also will follow-up with all persons who express an interest in the study to ensure that screening appointments are scheduled and carried out in a timely manner.

5.1.2 Retention

Once participants enroll in this study, the study site will make every effort to retain them for the duration of follow-up in order to minimize possible bias associated with loss-to-follow-up. The study staff is responsible for developing and implementing local standard operating procedures to target this goal. Components of such procedures include:

- Thorough explanation of the study visit schedule and procedural requirements during the informed consent process, and re-emphasis at each study visit
- Thorough explanation of the importance of all three treatment phases to the overall success of the study

- Use of appropriate and timely visit reminder mechanisms (via email and/or telephone)
- Immediate and multifaceted follow-up on missed visits

5.2 Inclusion Criteria

Individuals who meet the following criteria are eligible for inclusion in the study:

1. ≥ Age of 18 at screening

2. Willing and able to provide written informed consent for screening and enrollment

3. HIV-1 uninfected at screening according to the standard DAIDS algorithm in Appendix II

4. Willing and able to communicate in English

5. Willing and able to provide adequate locator information, as defined in site standard operating procedures (SOP)

6. Availability to return for all study visits, barring unforeseen circumstances

7. Per participant report at screening, a history of consensual RAI at least once in the prior year

8. Willing to abstain from insertion of anything rectally including sex toys, other than the study gel during the active phases of the study and for 5 days following biopsy collection

9. Willing to abstain from sexual intercourse (rectal and vaginal) during the active phases of the study and for 5 days following biopsy collection

10. Must agree to use study provided condoms for the duration of the study for vaginal and insertive anal intercourse

11. Must be in general good health

12. Must agree not to participate in other drug trials

In addition to the criteria listed above, female participants must meet the following criteria:

13. Post-menopausal or using (or willing to use) an acceptable form of contraception (e.g., barrier method, IUD, hormonal contraception, surgical sterilization, or vasectomization of male partner). If the female participant has female partners only,

the method of contraception will be noted as a barrier method in the study documentation.

5.3 Exclusion Criteria

Individuals who meet any of the following criteria at screening will be excluded from the study:

1. Abnormalities of the colorectal mucosa, or significant colorectal symptom(s), which in the opinion of the clinician represents a contraindication to biopsy (including but not limited to presence of any unresolved injury, infectious or inflammatory condition of the local mucosa, and presence of symptomatic external hemorrhoids)

2. At screening, clinical or laboratory diagnosis of active rectal or reproductive tract infection requiring treatment per current Centers for Disease Control and Prevention (CDC) guidelines or urinary tract infection (UTI). Infections requiring treatment include symptomatic bacterial vaginosis, symptomatic vaginal candidiasis, other vaginitis, trichomoniasis, Chlamydia (CT), gonorrhea (GC), syphilis, active HSV lesions, chancroid, pelvic inflammatory disease, genital sores or ulcers, cervicitis, or symptomatic genital warts requiring treatment. Note that HSV-2 seropositive with no active lesions is allowed, since treatment is not required.

   Note: Allow one re-screening after documented treatment (30 days) in cases of GC/CT identified at screening

3. At screening:
   a. Positive for hepatitis B surface antigen
   b. Hemoglobin < 10.0 g/dL
   c. Platelet count < 100,000/mm^3
   d. White blood cell count less than 2,000 cells/mm^3 or > than 15,000 cells/mm^3
   e. Calculated creatinine clearance less than 80 mL/min by the Cockcroft-Gault formula where creatinine clearance in mL/min (140- age in years) x (weight in kg) x (0.85 for females)/72 x (serum creatinine in mg/dL)
   f. Serum creatinine > 1.3× the site laboratory upper limit of normal (ULN)
   g. ALT and/or AST > 2.5× the site laboratory ULN
   h. +1 glucose or +1 protein on urinalysis (UA)
   i. History of bleeding problems (i.e. INR > 1.5× the site laboratory ULN or PTT > 1.25× the site laboratory ULN)

4. History of significant gastrointestinal bleeding in the opinion of the investigator

5. Allergy to methylparaben, propylparaben, sorbic acid

6. By participant report at enrollment, history of excessive daily alcohol use (as defined by the CDC as heavy drinking consisting of an average consumption of more than 2

drinks per day for men, and more than 1 drink per day for women), frequent binge drinking or illicit drug use that includes any injection drugs, methamphetamines (crystal meth), heroin, or cocaine including crack cocaine, within the past 12 months

7. Per participant report at screening, anticipated use and/or unwillingness to abstain from the following medications during the period of study participation:
    a. Heparin, including Lovenox®
    b. Warfarin
    c. Plavix® (clopidogrel bisulfate)]
    d. Rectally administered medications (including over-the-counter products)
    e. Aspirin
    f. NSAIDS
    g. Acyclovir
    h. Valacyclovir
    i. TDF
    j. Any other drugs that are associated with increased likelihood of bleeding following mucosal biopsy

8. By participant report at screening, use of systemic immunomodulatory medications, rectally administered medications, rectally administered products (including condoms) containing N-9, or any investigational products within the 4 weeks prior to the Enrollment/Baseline Evaluation Visit

9. History of recurrent urticaria

10. Any other condition or prior therapy that, in the opinion of the investigator, would preclude informed consent, make study participation unsafe, make the individual unsuitable for the study or unable to comply with the study requirements. Such conditions may include, but are not limited to, current or recent history of severe, progressive, or uncontrolled substance abuse, or renal, hepatic, hematological, gastrointestinal, endocrine, pulmonary, neurological, or cerebral disease.

In addition to the criteria listed above, female participants will be excluded if they meet any of the following criteria:

11. Pregnant at Enrollment/Baseline Evaluation Visit

12. Breastfeeding or intent to breastfeed during duration of study

6 STUDY PRODUCT

6.1 Regimen

Study participants will be randomized to Group A or Group B for the PK sampling time points and will then be randomized to either the active or placebo product for the rectal application phases. The oral phase will have no placebo. Each study sequence will consist of three study periods, two two-week sampling periods, and two two-week resting periods (see Table 11).

Study participants will receive the study products, which are tenofovir disoproxil fumarate 300mg tablet and tenofovir 1% gel or placebo gel. All participants will complete a single dose of TDF 300mg, followed by a two-week sampling period and a two-week resting period. Participants will then receive a single rectal dose of tenofovir 1% gel followed by a two-week sampling period, a 1-2 week resting period, and will then be instructed to apply one rectal dose of study gel daily for six consecutive days. The Day 7 dose will be administered in the clinic.

Table 11: Study Regimen

	Single Oral Dose	2 WK Sampling Period	2 WK Resting Period (+ 7 days)	Single Rectally-Applied Dose	2 WK Sampling Period	2 WK Resting Period (+ 7 days)	7-day Exposure Dose
**Group A**	Oral TDF	Day 0 (30 min),<br><br>Day 1 and Day 7		Tenofovir 1% Gel or Placebo Gel	Day 0 (30 min),<br><br>Day 1 and Day 7		Participants to receive same product as administered during single rectally applied dose
**Group B**	Oral TDF	Day 0 (30 min),<br><br>Day 4 and Day 10		Tenofovir 1% Gel or Placebo Gel	Day 0 (30 min),<br><br>Day 4 and Day 10		Participants to receive same product as administered during single rectally applied dose

6.2 Administration

Study staff will instruct participants in proper methods of administering and storing their study product(s).

Administration of the single oral dose and the single rectally applied dose will be observed by the clinician at the site. Daily rectal administration of the gel for the 7-day exposure will be administered by the participant days 1-6 and day 7 will be administered in the clinic. Administration of study product should occur in the morning. If a daily dose is missed, the participant will be instructed to administer the missed dose as soon as possible, unless the next dose is due within 6 hours. If the next dose is due within 6 hours, the missed dose will be skipped and the next dose administered as originally scheduled.

Oral Study Product

Tenofovir Disoproxil Fumarate 300 mg Oral Tablet (Single Dose)
Study participants will be administered a single oral dose observed by the clinician or designee at the site. Pharmacokinetic sampling will occur on Day 1 and Day 7 for Group A and on Day 4 and Day 10 for Group B.

Rectal Study Product
Both groups will be randomized to receive an observed single rectal application of either tenofovir 1% gel or HEC placebo gel. This single dose will be administered by the clinician or designee at the site. Pharmacokinetic sampling will occur on day 1 and day 7 for Group A and on day 4 and day 10 for Group B. Following this 2-week pharmacokinetic sampling period, the gel will be administered by the participant daily for 6 consecutive days and day 7 will be administered in the clinic.

6.3 Study Product Formulation

6.3.1 Tenofovir Disoproxil Fumarate 300 mg Tablets

Tenofovir disoproxil fumarate (Viread®, TDF) oral tablet, is a fumaric acid salt of bis-isopropoxycarbonyloxymethyl ester derivative of tenofovir. Each tablet contains 300 mg of tenofovir disoproxil fumarate, which is equivalent to 245 mg of tenofovir disoproxil. TDF tablets should be stored and dispensed in the original container. Each bottle should contain a silica gel desiccant to protect the product from humidity, and this should remain in the container. TDF should be stored at 25°C (77°F). Excursions permitted between 15°C and 30°C (59°F and 86°F).

6.3.2 Tenofovir 1% Gel

Tenofovir 1% gel (weight/weight) is a gel formulation of tenofovir (PMPA, 9-[(R)-2-(phosphonomethoxy)propyl]adenine monohydrate), formulated in purified water with edetate disodium, citric acid, glycerin, methylparaben, propylparaben, hydroxyethylcellulose, and pH adjusted to 4-5. Tenofovir 1% gel is a transparent, viscous gel that will be filled into applicators to form pre-filled, single-use applicators. Each pre-filled applicator is expected to deliver approximately 4.0mL of tenofovir 1% gel (equal to 4.4g).

Tenofovir 1% gel must be stored at controlled room temperature, 25°C (77°F), at all times. Excursions are permitted between 15°C and 30°C (59°F and 86°F).

6.3.3 Placebo Gel

HEC gel, sometimes called the “universal” placebo gel contains hydroxyethylcellulose as the gel thickener, purified water, sodium chloride, sorbic acid and sodium hydroxide.[34] The gel is isotonic and formulated at a pH of 4.4 to avoid disrupting the normal vaginal pH and has minimal buffering capacity to avoid the inactivation of

sexually transmitted pathogens. Hydroxyethylcellulose, the gelling agent, is used to approximate the viscosity of other microbicide gel candidates. Each pre-filled applicator will contain approximately 4 mL of HEC placebo gel for delivery.

HEC gel should be stored at 25°C (77°F). Excursions are permitted between 15°C and 30°C (59°F and 86°F).

6.4 Study Product Supply and Accountability

6.4.1 Study Product Supply

Tenofovir Disoproxil Fumarate (TDF) 300 mg Tablets
TDF will be supplied by Gilead Sciences, Inc (Foster City, CA). Each site will receive one bottle of tenofovir disoproxil fumarate which will contain 30 tablets. The tablets will be shipped directly from Gilead to each site. The tablets will be stored in the pharmacy and will be dispensed from the pharmacy.

Tenofovir 1% Gel
Tenofovir 1% gel will be supplied by CONRAD (Arlington, VA, USA). Under direction from CONRAD, Patheon Inc., (Cincinnati, OH USA) which is a contract manufacturing facility, will manufacture the tenofovir 1% gel and analyze/release the gels under good manufacturing practices (cGMP). Patheon Inc., will fill the applicators with tenofovir 1% gel to create pre-filled applicators and package each applicator and plunger in a wrapper.

HEC Gel
HEC gel will be supplied by CONRAD (Arlington, VA, USA). Under direction from CONRAD, Patheon Inc., (Cincinnati, OH USA) which is a contract manufacturing facility, will manufacture the HEC gel, and analyze/release the gels under good manufacturing practices (cGMP). Patheon Inc., will fill the applicators with HEC gel to create pre-filled applicators and package each applicator and plunger in a wrapper.

The applicators will be shipped directly to the site pharmacy. The applicators will be stored in the pharmacy and will be dispensed from the pharmacy.

6.4.2 Dispensing

Study products are dispensed only to enrolled participants, upon receipt of a written prescription from an authorized prescriber. Study products will be dispensed from the pharmacy to study staff for an enrolled participant or directly to the enrolled participant upon receipt of a written prescription from an authorized prescriber. The observed doses will be administered by the site investigator using product obtained by the study staff or the participant. Each site will prepare a Chain of Custody SOP which will specifically outline the dispensing process and procedures.

One TDF 300 mg tablet will be dispensed at Visit 3 and administered at the site under the supervision of the clinician or designee.

Depending on the arm of the study to which the participant is randomized, a single applicator of either tenofovir 1% gel or HEC placebo gel will be dispensed at Visit 7 and administered rectally at the site by the clinician or designee.
At Visit 11, participants will receive 7 pre-filled applicators containing the same gel previously administered at Visit 7. Participants will rectally administer one application daily for 6 days. Participants will also receive 2 resealable bags. One bag will be labeled “used applicators” and the second bag will be labeled “unused applicators”.

At Visit 12, one pre-filled applicator containing the same gel previously administered will be dispensed for administration at the site under the supervision of the clinician or designee.

6.4.3 Retrieval of Unused Study Products

Study participants will be instructed to bring any unused applicators back to the clinic at Visit 12. In the event that unused applicators are not returned to the site, study staff members will make attempts to retrieve unused study products.

The Pharmacist of Record (PoR) will document all product returns and store the returned study products in designated areas within the study pharmacy.

6.4.4 Accountability

The PoR or designee at the site is required to maintain a complete record of all study products received from the manufacturer and subsequently dispensed and to monitor product expiration dates. All unused study products are to be returned to the study site. At the end of the study, specific instructions will be provided for the return or destruction of the study products by the study site. All drug returned and destroyed will also be recorded on the site accountability logs.

6.5 Assessment of Participant Adherence

The single-dose administrations of study products will be completed during clinic visits (Visit 3 and Visit 7) under observation of the clinician or designee. Anything of note during this process will be recorded in source documents.

For the seven-day use of the study gel, participants will be instructed to keep a Product Log (Appendix X) where they will record the date and time of the product administration. The directions will encourage participants to always use the study product in the morning and to record the time at which the product was administered. In addition, to assure the accurate recording of product administration at home, it will be arranged that a phone call will be placed by study staff every day at a certain agreed-upon hour. Study coordinator and participants will make the necessary arrangements for this call at

Visit 11. This will be a short daily phone call, with the participant reporting the time of product administration and the coordinator making a daily note of this report. This phone call will also be an agreed-upon reminder to those participants who might otherwise inadvertently miss their daily doses. Participants will also be able to note anything of significance related to the process or the gel. Daily notes from these phone calls, in addition to the participant log, will be reviewed at the Final Clinic/Early Termination Visit (Visit 12/Visit 13)

Furthermore, participants will be asked to return in separate, sealed bags (supplied at Visit 11) both used and unused applicators. Thus we will be able to cross validate self-reports, and applicator counts to assess adherence.

6.6 Concomitant Medications

With the exception of medications listed as prohibited, enrolled study participants may use concomitant medications during study participation. All concomitant medications reported throughout the course of the study will be recorded on case report forms designated for that purpose. Prescription medications, over-the-counter preparations, vitamins and nutritional supplements, recreational drugs, and herbal preparations will all be recorded on forms for concomitant medications.

6.6.1 Prohibited Medications and Procedures

Study participants will be prohibited from using the following medications throughout the study period: rectally administered medications (including over-the-counter products), aspirin, NSAIDS, warfarin, Plavix® (clopidogrel bisulfate), heparin (including Lovenox®), acyclovir, valacyclovir, tenofovir disoproxil fumarate, and other drugs that are associated with increased likelihood of bleeding following mucosal biopsy. Furthermore, study participants will be counseled to avoid use the following products within 4 weeks of the Enrollment Visit or during the study: systemic immunomodulatory medications, rectally administered medications, rectally administered products (including condoms) containing N-9, or any other investigational products. Should participants report use of any of these medications or products, they will be required to discontinue use of study product, but will continue to complete all scheduled study visits.

Participants are not expected to require rectal procedures during follow-up; however, should such a procedure be required, the Investigator of Record (IoR)/designees will consult the Data Safety Monitoring Board (DSMB) regarding ongoing product use by the participant.

7 STUDY PROCEDURES

An overview of the study visits and evaluations schedule is presented in Appendix I. Presented in this section is additional information on visit-specific study procedures. A

detailed instruction guide will be provided in the RMP-02/MTN-006 Study-Specific Procedures Manual. In addition to any Interim Visits that may occur in accordance with guidance outlined in Section 7.6, the following visits should take place for study participants:

- Visit 1 (Screening Visit)
- Visit 2 (Enrollment/Baseline Visit)
- Visit 3 (Oral: Study Product Visit #1 and 30 min Sampling)
- Visit 4 (Oral: 24-h Post-Exposure Sampling Period)*
- Visit 5 (Oral: Sampling Period)*
- Visit 6 (Oral: Sampling Period)
- Visit 7 (Rectal-single dose: Study Product Visit #2 and 30 min Sampling)
- Visit 8 (Rectal-single dose: 24-h Post-Exposure Sampling Period)*
- Visit 9 (Rectal-single dose: Sampling Period)*
- Visit 10 (Rectal-single dose: Sampling Period)
- Visit 11 (Rectal-7 day dose: Study Product Visit #3)
- Visit 12 (Rectal-7 day dose: Product Use End Sampling Visit)
- Visit 13 (Rectal 7 day dose: 24-h Post-Exposure Sampling Period)
- Follow-Up Safety Phone Call

All potential study participants will be prescreened via a phone call and will be mailed the informed consent documents for their review prior to the Screening Visit.

* Study participants will be randomized into two different groups based on sampling time points (Groups A and B) per Section 10.5 of the protocol. Please note that for participants in Group A, Visits 4 and 5a and Visits 8 and 9a, will take place at the same time.

The diagram below provides an outline of study visits:

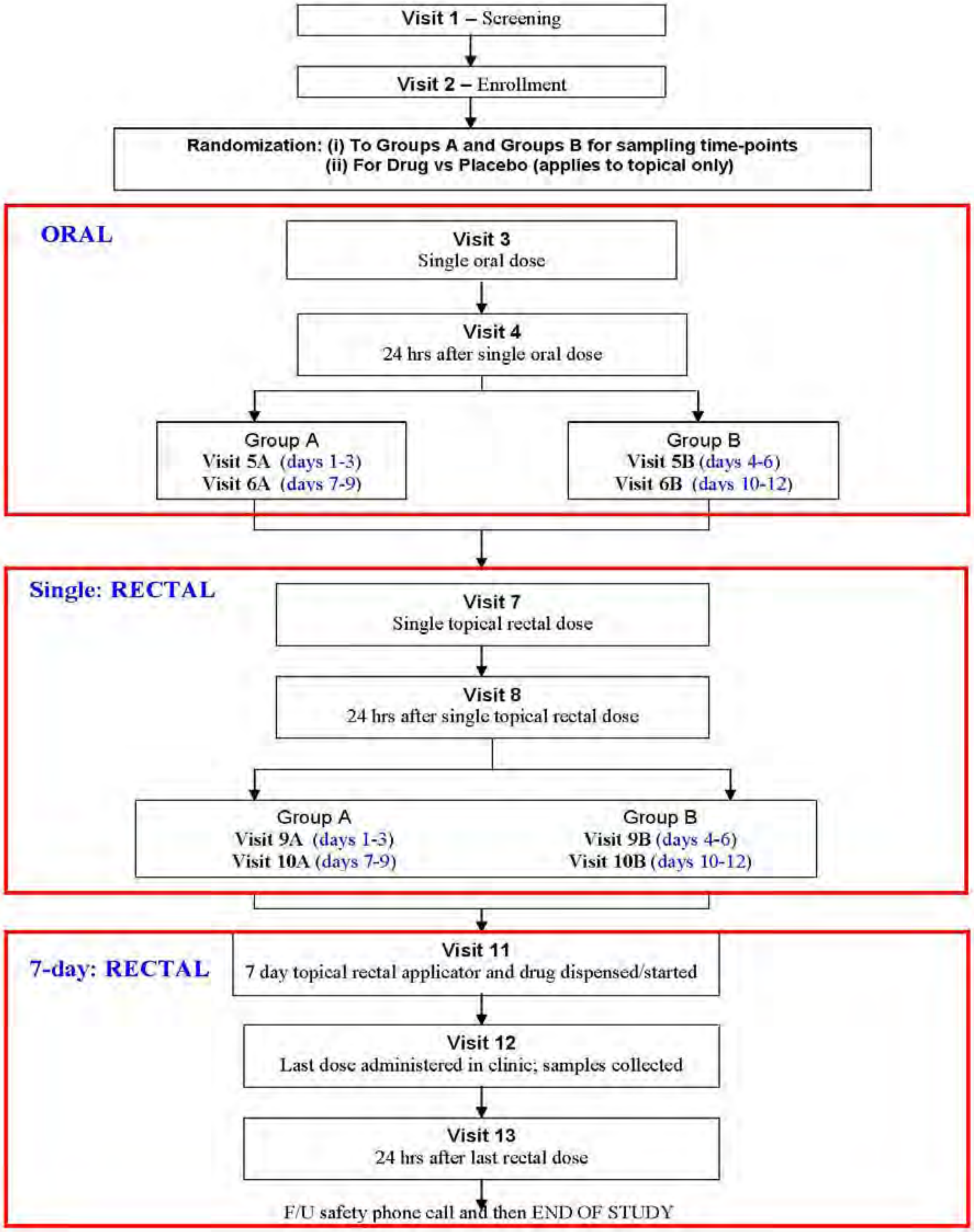

Figure 1: Outline of Study Visits and Study Product Administered

The table below presents an outline of the visit windows:

Table 12: Study Visit Windows

**Visit 1**	Screening	
**Visit 2**	Week 0 – Enrollment/ Baseline	Up to 30 days after Screening
**Visit 3**	Week 2 – Single Oral	+ or – 7 days
**Visit 4**	24 h post Visit 3	At least 18, but no more than 30 hours post Visit 3
**Visit 5**	Group A: 1-day Visit post Visit 3<br>Group B: 4-day Visit post Visit 3	Group A: 1-3 days post Visit 3<br>Group B: 4-6 days post Visit 3
**Visit 6**	Group A: 7-day Visit post Visit 3<br>Group B: 10-day Visit post Visit 3	Group A: 7-9 days post Visit 3<br>Group B: 10-12 days post Visit 3
Resting period		
**Visit 7**	Week 6 – Single Rectal	+ or – 1 week
**Visit 8**	**24 h post Visit 7**	At least 18, but no more than 30 hours post Visit 7
**Visit 9**	Group A: 1-day Visit post Visit 7<br>Group B: 4-day Visit post Visit 7	Group A: 1-3 days post Visit 7<br>Group B: 4-6 days post Visit 7
**Visit 10**	Group A: 7-day Visit post Visit 7<br>Group B: 10-day Visit post Visit 7	Group A: 7-9 days post Visit 7<br>Group B: 10-12 days post Visit 7
Resting period		
**Visit 11**	Week 10 – 7 Day Rectal Dispensed	+ or – 7 days
**Visit 12**	Week 11 – 7 Day Rectal Sample Collection	No window
**Visit 13**	24 h post Visit 12	At least 18, but no more than 30 hours post Visit 12
**N/A**	Follow-up Phone Call	Within 7 days of Visit 12

7.1 Screening Visit

Screening may take place up to 30 days prior to the Enrollment/Baseline Evaluation Visit. Written informed consent will be obtained before any screening procedures are initiated. For participants who do not meet the eligibility criteria, screening will be discontinued once ineligibility is determined.

For participants who are found to be presumptively eligible based on the evaluations listed below at these visits, final eligibility will be confirmed at the Enrollment/Baseline Evaluation Visit.

Table 13: Visit 1 (Screening Visit)

Screening Visit (up to and including 30 days prior to Enrollment Visit)	
**Component**	**Procedure/Analysis**
**Administrative**	• Obtain written informed consent for Screening Visit<br>• Assign participant ID (PTID)<br>• Collect demographic information<br>• Collect locator information<br>• Assess eligibility<br>• Provide reimbursement for study visit (per site standard)<br>• *Schedule next study visit
**Clinical**	• Collect medical/menstrual history (including exclusionary medical conditions and medications)<br>• Review concomitant medications<br>• Perform physical exam<br>• Assessment of smoking status<br>• Perform digital rectal exam<br>• Provide male condoms<br>• Provide counseling<br>o HIV pre-test and post-test<br>o HIV/STI risk reduction<br>o Contraceptive
**Urine**	• Collect urine sample<br>o ♀Qualitative hCG<br>o Dipstick Urinalysis (UA) for protein, glucose, nitrates, and leukocyte esterase<br>o Nucleic acid amplification test (NAAT) for GC/CT
**Blood**	• Collect blood specimens<br>o Complete blood count with differential and platelets<br>o ALT, AST, creatinine, phosphate<br>o HBsAg<br>o Syphilis RPR (confirmatory tests as needed)<br>o HIV-1 serology (confirmatory tests as needed)<br>o HSV-1 and 2 serology
**Female Pelvic Specimens**	• Self-collected samples:<br>o Vaginal pH (Swab)<br>o Vaginal swab for Gram stain, BV assessment (Swab)
**Rectal Specimens**	• Anorectal swabs<br>o Rectal *Chlamydia trachomatis and Neisseria gonorrhoeae by NAAT*

♀ for females of childbearing potential

7.2 Enrollment/Baseline Evaluation Visit

Table 14: Visit 2 (Enrollment/Baseline Evaluation Visit-All Participants)

**Enrollment/Baseline Visit**	
**Component**	**Procedure/Analysis**
**Administrative**	• Obtain written informed consent for Enrollment/Baseline Visit<br>• Collect demographic information<br>• Collect locator information<br>• Confirm eligibility<br>• Provide reimbursement for study visit<br>• Schedule next study visit<br>• Randomization (to be done at the end of Visit 2)
**Clinical**	• Review/update medical and menstrual history<br>• Review/update concomitant medications<br>• Perform physical exam<br>• Record/Update AEs<br>• Provide test results<br>• Provide male condoms<br>• Provide counseling<br>o Adherence (protocol)<br>o HIV pre-test and post-test*<br>o HIV/STI risk reduction<br>o Contraceptive
**Behavioral Assessment**	• Administer Baseline Behavioral Questionnaire (BBQ)<br>o Instruct participant in use of web-based questionnaire
**Urine**	• Collect urine sample<br>o ♀Qualitative hCG
**Blood**	• Collect blood specimens<br>o Complete blood count with differential and platelets<br>o ALT, AST, creatinine, phosphate<br>o HIV-1 serology (confirmatory tests as needed)*<br>o Plasma archive
**Female Pelvic Specimens**	• Self-collected samples:<br>o Vaginal pH<br>o Vaginal swab for Gram stain, BV assessment<br>o Sponge (dry) for baseline PK
**Rectal Specimens**	• Rectal Sponges<br>o Baseline PK<br>o Rectal secreted cytokines<br>• Normosol preparatory enema<br>• Stool from preparatory enema<br>o Calprotectin<br>• Endoscopic lavage<br>o Epithelial sloughing<br>• Flexible sigmoidoscopy (~17 biopsies)<br>o Histology<br>o CD4 cells (MMC phenotyping)<br>o HIV infectability in colorectal tissue explant culture<br>o Tenofovir levels in rectal mucosal tissue homogenates<br>*If indicated

7.3 Follow-up Visits

Table 15: Visit 3 and Visit 7 (Study Product #1; Study Product #2 and 30' sampling for each)

**Study Product #1 (single oral) and Study Product #2 (single topical) + 7 days**	
**Component**	**Procedure/Analysis**
**Administrative**	• Review/update locator information<br>• Provide reimbursement for study visit<br>• Schedule next study visit
**Clinical**	• Review/update medical menstrual history<br>• Review/update concomitant medications<br>• Perform physical exam**<br>• Provide test results<br>• Record/Update AEs<br>• Provide male condoms<br>• Provide counseling<br>o Adherence (protocol and product use)<br>o HIV pre-test and post-test*<br>o HIV/STI risk reduction<br>o Contraceptive
**Urine**	• Collect urine sample<br>o ♀Qualitative hCG
**Blood**	• Collect blood specimens (pre-dosing)<br>o Complete blood count with differential and platelets<br>o ALT, AST, creatinine, phosphate<br>o HIV-1 serology (confirmatory tests as needed)*<br>o Plasma tenofovir levels<br>o PBMC tenofovir levels<br>• Collect blood specimens (30 min, 2 h, and 4 h post-dosing)<br>o Plasma tenofovir levels<br>o PBMC tenofovir levels
**Female Pelvic Specimens**	• Self-collected samples (pre-dosing):<br>o Vaginal pH<br>o Vaginal swab for Gram stain, BV assessment<br>o Vaginal sponge (dry) for tenofovir concentrations<br>*Samples to be taken again 30 min, 2 h, and 4 h post-dosing*<br>• Self-collected samples<br>o Vaginal sponge (dry) for tenofovir concentrations
**Rectal Specimens**	• Normosol preparatory enema<br>• Stool from preparatory enema<br>o Calprotectin<br>*Samples to be taken pre-dosing*<br>• Rectal Sponges (pre-moistened)<br>o Rectal secreted cytokines<br>o Rectal tenofovir concentrations (dry sponge)<br>• Anorectal swabs<br>o Rectal microflora<br>*Samples to be taken again 30 min post-dosing*<br>• Endoscopic lavage<br>o Epithelial sloughing<br>• Flexible sigmoidoscopy (~17 biopsies)<br>o Histology<br>o CD4 cells (MMC phenotyping and Tenofovir in MMC)<br>o HIV infectability in colorectal tissue explant cultures<br>o Tenofovir levels in rectal mucosal tissue homogenates<br>• Rectal Sponges<br>o Rectal secreted cytokines (pre-moistened sponge)<br>o Rectal tenofovir concentrations (dry sponge)<br>*Samples to be taken again 2 and 4 hrs post-dosing*<br>• Rectal Sponges<br>o Rectal tenofovir concentrations (dry sponge)
**Study Product**	• Single dose of TDF 300mg tablet (Visit 3) OR<br>• Single dose of rectally applied study product (tenofovir 1% gel or placebo gel) (Visit 7)<br>*If indicated ** excluding height and weight

Table 16: Visits 4, 8, and 13(24-Hour Post-Exposure PK Visit: All Participants)

24-Hour Post-Exposure Visit (at least 18, but no more than 30 hours post Visits 3, 7, and 12 respectively)	
**Component**	**Procedure/Analysis**
**Administrative**	• Review/update locator information<br>• Provide reimbursement for study visit<br>• Schedule next study visit
**Clinical**	• Review/update medical menstrual history<br>• Review/update concomitant medications<br>• Perform physical exam**<br>• Provide test results<br>• Record/Update AEs<br>• Provide male condoms<br>• Provide counseling<br>o Adherence (protocol)<br>o HIV pre-test and post-test*<br>o HIV/STI risk reduction<br>o Contraceptive
**Blood**	• Collect blood specimens<br>o HIV-1 serology (confirmatory tests as needed)*<br>o Plasma tenofovir levels<br>o PBMC tenofovir levels
**Female Pelvic Specimens**	• Vaginal sponge (dry) for tenofovir concentrations
**Rectal Specimens**	• Rectal Sponges<br>o Rectal tenofovir concentrations (dry sponge)

*If indicated **excluding height and weight

Table 17: Visits 5 and 6 and Visits 9 and 10 (2-Week Sampling Period)

**2-Week Sampling Period<br>(Visits 5 and 9: Group A: 1-3 days post Visits 3 and 7; Group B: 4-6 days post Visits 3 and 7)<br>(Visits 6 and 10: Group A: 7-9 days post Visits 3 and 7; Group B: 10-12 days post Visits 3 and 7)**	
**Component**	**Procedure/Analysis**
**Administrative**	• Review/update locator information<br>• Provide reimbursement for study visit<br>• Schedule next study visit
**Clinical**	• Review/update medical and menstrual history<br>• Review/update concomitant medications<br>• Perform physical exam**<br>• Record/Update AEs<br>• Provide male condoms<br>• Provide counseling<br>o Adherence (protocol)<br>o HIV pre-test and post-test*<br>o HIV/STI risk reduction<br>o Contraceptive
**Blood**	• Collect blood specimens<br>o HIV-1 serology (confirmatory tests as needed)*<br>o Plasma tenofovir levels<br>o PBMC tenofovir levels
**Female Pelvic Specimens**	• Self-collected samples:<br>o Vaginal pH<br>o Vaginal swab for Gram stain, BV assessment<br>• Vaginal sponge (dry) for tenofovir concentrations
**Rectal Specimens**	• Normosol preparatory enema<br>• Stool from preparatory enema<br>o Calprotectin<br>• Endoscopic lavage<br>o Epithelial sloughing<br>• Flexible sigmoidoscopy (~13 biopsies at Visits 6 and 10, ~17 biopsies at Visits 5 and 9)<br>o Histology (Visits 5 and 9 only)<br>o CD4 cells (MMC phenotyping[Visits 5 and 9 only])<br>o CD4 cells (tenofovir in MMC[Visits 5 and 9 only])<br>o HIV infectability in colorectal tissue explant cultures<br>o Tenofovir levels in rectal mucosal tissue homogenates<br>• Rectal Sponges<br>o Rectal tenofovir concentrations (dry sponge)<br>o Rectal secreted cytokines (pre-moistened sponge)<br>*If indicated **excluding height and weight

Table 18: Visit 11 (Study Product Visit #3: Dispense Self-Administered Gel Supply)

Study Product Visit #3 (± 7 days)	
**Component**	**Procedure/Analysis**
**Administrative**	• Review/update locator information<br>• Provide reimbursement for study visit<br>• Schedule next study visit
**Clinical**	• Review/update medical menstrual history<br>• Review/update concomitant medications<br>• Perform physical exam**<br>• Provide test results<br>• Record/Update AEs<br>• Provide male condoms<br>• Provide counseling<br>o Adherence (protocol and product use)<br>o HIV pre-test and post-test*<br>o HIV/STI risk reduction<br>o Contraceptive
**Urine**	• Collect urine sample<br>o ♀Qualitative hCG
**Blood**	• Collect blood specimens<br>o HIV-1 serology (confirmatory tests as needed)*<br>o Plasma tenofovir levels<br>o PBMC tenofovir levels
**Female Pelvic Specimens**	• Vaginal sponge (dry) for tenofovir concentrations
**Rectal Specimens**	• Rectal Sponges<br>o Rectal tenofovir concentrations (dry sponge)
**Study Product**	• Dispense 6-day dose of rectally applied study product (tenofovir 1% gel or placebo gel)<br>• Remind subjects that dosing should be done each morning and recorded in study diary<br>• Arrange for daily product administration verification call<br>*If indicated **excluding height and weight

7.4 Product Use End Visit

The Product Use End Visit will take place the day following the participants apply their last dose of study gel at home. Planned efforts are to have this visit occur at least 18, but no more than 30 hours post Visit 12. The 7th dose will be an observed dose at the clinic. Blood and rectal samples for all endpoints will be taken at this visit.

Table 19: Visit 12 (Sampling Visit Following Once Daily Exposure for 7 Days (7th dose given in clinic))

Product Use End Visit (No window)	
**Component**	**Procedure/Analysis**
**Administrative**	• Review/update locator information<br>• Provide reimbursement for study visit<br>• Schedule next study visit
**Clinical**	• Review/update medical menstrual history<br>• Review/update concomitant medications<br>• Perform physical exam**<br>• Provide test results<br>• Record/Update AEs<br>• Provide male condoms<br>• Provide counseling<br>o Adherence (protocol)<br>o HIV pre-test and post-test<br>o HIV/STI risk reduction<br>o Contraceptive
**Urine**	• Collect urine sample<br>• ♀Qualitative hCG
**Blood**	• Collect Blood specimens (pre-dosing)<br>o Complete blood count with differential and platelets<br>o ALT, AST, creatinine, phosphate<br>o HIV-1 serology (confirmatory tests as needed)<br>o Syphilis RPR (confirmatory tests as needed)<br>o Plasma tenofovir levels<br>o PBMC tenofovir levels<br>• Collect Blood specimens (30 min, 2 h, 4h post-dosing)<br>o Plasma tenofovir levels<br>o PBMC tenofovir levels
**Female Pelvic Specimens**	• Self-collected samples (pre-dosing):<br>o Vaginal pH<br>o Vaginal swab for Gram stain, BV assessment<br>o Vaginal sponge (dry) for tenofovir concentrations<br>*Samples to be taken 30 min, and 2 and 4 h post-dosing*<br>• Self-collected samples<br>o Vaginal sponge (dry) for tenofovir concentrations
**Rectal Specimens**	• Normosol preparatory enema<br>• Stool from preparatory enema<br>o Calprotectin<br>*Samples to be taken pre-dosing*<br>• Rectal Sponges<br>o Rectal tenofovir concentrations (dry sponge)<br>o Rectal secreted cytokines (pre-moistened sponge)<br>• Anorectal swabs<br>o Rectal microflora<br>*Samples to be taken 30 minutes post-dosing*<br>• Endoscopic lavage<br>o Epithelial sloughing<br>• Flexible sigmoidoscopy (~17)<br>o Histology<br>o CD4 cells (MMC phenotyping and Tenofovir levels in MMC)<br>o HIV infectability in colorectal tissue in explant cultures<br>o Tenofovir levels in rectal mucosal tissue homogenates<br>• Rectal Sponges<br>o Rectal tenofovir concentrations (dry sponge)<br>o Rectal secreted cytokines (pre-moistened sponge)<br>*Samples to be taken 2 and 4 h post-dosing*<br>• Rectal Sponges<br>o Rectal tenofovir concentrations (dry sponge)
**Study Product**	• Collect used and unused applicators<br>• Collect study product use log and review daily product administration verification call log<br>• Receive 7th dose of study product in the clinic

** excluding height and weight

7.5 Final Clinic Visit

Participants will be asked to come to the clinic 24 hours after their Product Use End Visit for a 24 h post-exposure sampling.

Table 20: Visit 13 Final Clinic/Early Termination Visit

Final Clinic Visit	
**Component**	**Procedure/Analysis**
**Administrative**	• Provide reimbursement for study visit<br>• Confirm follow-up phone assessment
**Clinical**	• Review/update medical menstrual history<br>• Review/update concomitant medications<br>• Record/Update AEs<br>• Provide male condoms<br>• Provide counseling<br>o HIV/STI risk reduction<br>o Contraceptive
**Behavioral Assessment**	• Administer Product Acceptability Questionnaire (web-based)<br>• In depth phone interview
**Blood**	• Blood specimens<br>o Plasma tenofovir levels<br>o PBMC tenofovir levels
**Female Pelvic Specimens**	• Vaginal sponge (dry) for tenofovir concentrations
**Rectal Specimens**	• Rectal Sponges<br>o Rectal tenofovir concentrations (dry sponge)<br>o Rectal secreted cytokines (premoistened sponge)<br>• Anorectal swabs<br>o Rectal microflora (see Appendix V)
**Study Product**	• Collect used and unused applicators (for Early Termination Visit only)<br>• Collect study product use log and review daily product administration verification call log (for Early Termination Visit only)

7.6 Follow-up Safety Phone Call

Participants will be asked to phone the clinic within 7 days of their Final Clinic Visit to report any AEs they might have experienced following study participation. A clinic visit will be scheduled if indicated.

Table 21: Follow-up Safety Phone Call

Within 7 days of Final Clinic Visit	
**Component**	**Procedure/Analysis**
**Clinical**	• Record/Update AEs<br>• Schedule visit if indicated

7.7 Follow up Procedures for Participants Who Discontinue Study Product

Participants who discontinue study product will be encouraged to remain in the study if they are willing, for safety evaluations according to the study follow-up schedule with the exceptions described below.

7.7.1 Participants Who Seroconvert to HIV

Study staff will capture seroconversions on study case report forms (CRFs) and will also refer participants for additional counseling related to testing or diagnosis if need or requested by the participant. Protocol-specified procedures will continue except:

- HIV serology
- Provision of study product
- Participant education
- Counseling for HIV/STI risk reduction. Counseling will be modified to address primary and secondary HIV/STI prevention for infected individuals.
- Biopsy collection via flexible sigmoidoscopy
- Collection of blood samples
- Collection of urine samples
- Collection of anorectal swabs and sponges
- Collection of vaginal swabs and sponges

7.7.2 Participants Who Become Pregnant

If a participant becomes pregnant products administration will be immediately discontinued. The participant will not be withdrawn from the study and will be followed until the outcome of the pregnancy is known. Every effort will be made to complete protocol-specified visits and procedures with these participants with the following exceptions:

- Provision of study product
- Biopsy collection via flexible sigmoidoscopy
- Collection of anorectal swabs and sponges
- Collection of vaginal swabs and sponges

7.7.3 Participants Who Voluntarily Discontinue Study Gel and/or Tablets

Participants who temporarily or permanently discontinue study gel and/or tablets will not routinely be withdrawn from the study. Rather, every effort will be made to complete all protocol-specified visits and procedures with these participants with the following exceptions:

- Provision of study product
- Participant education
- Biopsy collection via flexible sigmoidoscopy
- Collection of blood samples
- Collection of urine samples
- Collection of anorectal swabs and sponges
- Collection of vaginal swabs and sponges

7.7.4 Participants Who Are Discontinued from Study Gel and/or Tablet Use by the Site Investigator

All protocol-specified study procedures will continue except:

- Provision of study product (permanent discontinuation)
- Participant education (permanent discontinuation)
- Biopsy collection via flexible sigmoidoscopy
- Collection of blood samples
- Collection of urine samples
- Collection of anorectal swabs and sponges
- Collection of vaginal swabs and sponges

7.8 Interim Contacts and Visits

Interim contacts and visits (those between regularly scheduled follow up visits) may be performed at participant request or as deemed necessary by the investigator or designee at any time during the study. All interim contacts and visits will be documented in participants' study records and on applicable case report forms.

Some Interim visits may occur for administrative reasons. For example the participant may have questions for study staff. Other interim contacts and visits may occur in response to AEs experienced by study participants. When interim contacts or visits are completed in response to participant reports of AEs, study staff will assess the reported event clinically and provide or refer the participant to appropriate medical care.

Table 22: Interim Contacts and Visits

Interim Visits	
**Component**	**Procedure/Analysis**
**Administrative**	• Update locator information<br>• Schedule next study visit*
**Clinical**	• Review concomitant medications<br>• Record/update AEs*<br>• Perform physical exam*<br>• Perform rectal exam*<br>• Provide counseling<br>o Adherence (protocol and product use)*<br>o HIV pre-test<br>o HIV/STI risk reduction<br>o Contraceptive
**Urine**	• Collect urine sample<br>o ♀Qualitative hCG*<br>o GC/CT*
**Blood**	• Collect blood specimens<br>o Complete blood count with differential and platelets *<br>o ALT, AST, creatinine, phosphate *<br>o Syphilis RPR (confirmatory tests as needed)*<br>o HIV-1 serology (confirmatory tests as needed)*
**Female Pelvic Specimens**	• Self-collected samples<br>o Vaginal pH*<br>o Vaginal swab for Gram stain, BV assessment*

*if clinically indicated

7.9 Clinical Evaluations and Procedures

The following physical and rectal exam components will be conducted at select visits.

Physical Exam

- Height (may be omitted after the Enrollment Visit)
- Weight (may be omitted after the Enrollment Visit)
- Vital signs
  - Temperature
  - Pulse
  - Blood pressure
- General appearance
- Abdomen
- Other components as indicated by participant symptoms

Medical History

Each participant will be asked about any symptoms or AEs experienced since their previous visit

Rectal Exam

- The participant will be positioned in the left lateral decubitus position
- Digital rectal examination: The examiner will insert a lubricated gloved finger into the anal canal and sweep around the internal anal circumference.
- Rectal STI and sponge collection: A lubricated plastic anoscope will be gently and fully inserted (until the lateral 'wings' touch the anal margin) and the obturator removed. Swabs for GC/CT will be sequentially inserted through the anoscope and placed in contact with the rectal wall, turned through 360 degrees and removed. Next the two sponges will be inserted through the anoscope and placed in contact with rectum and remain there for 5 minutes. The sponges will then be removed and packaged, and then the anoscope will be slowly removed.
- Rectal lavage: A 125 mL Normosol® enema will be inserted through the anus and the contents squeezed into the rectum. The participant will hold the fluid in the rectum for 5 minutes then expel it, including stool, into a collection device placed over a toilet bowl.
- Flexible sigmoidoscopy and biopsy: A flexible sigmoidoscope will be inserted to approximately 10cm-15cm and 13-17 biopsies taken using large-cup biopsy forceps.

Self-Administered Cervicovaginal fluid collection

- Vaginal pH (swab)
- Vaginal fluid (sponge)

7.10 Behavioral Measures

Two sets of behavioral measures will be used in this protocol:

Baseline Behavioral Questionnaire

This will be a Web-based self interview that the participant will complete at the Enrollment Visit at a computer terminal located in the research offices. In addition to demographics, this questionnaire will assess participants' sexual behavior in the prior three months with HIV-negative, positive, or unknown status men and women, including among men, their sexual role—insertive, receptive, or versatile, and frequency of condom use.[82] The assessment will also include questions on use of hyper-osmolar or hypo-osmolar rectal lubricants, rectal douching prior to sexual intercourse, use of lubricants containing N-9, and other behavioral practices that may affect the anal sphincter or rectal compartment. It will also include questions on frequency of alcohol and drug use in the prior three months and frequency of HIV testing. Finally, the assessment will explore participants' attitudes about PrEP and post-exposure prophylaxis (PEP), knowledge about microbicides and likelihood of using a microbicide in the future.

Product Acceptability Questionnaire

This Web-based self-interview will be completed by the participant at the Final Clinic Visit. This questionnaire will include structured and semi-structured questions about the experiences the participant had using the gel rectally, likes and dislikes concerning the

gel, the applicator, and the application process, any changes s/he may have introduced or may wish to introduce in the product used, any problems (e.g., leakage, soiling) s/he may have had, or other product side-effects and how much the participant was bothered by them, and likelihood of using a rectally applied microbicide in the future. This last section has items worded similarly to those of the same section administered at baseline so that we will be able to compare the anticipated likelihood of product use before and after participants become familiar with a product.

In-depth Interview

In-depth research is needed to better characterize the different circumstances in which microbicides may be used that may restrict acceptability and ultimately the use of methods for administration of the product, e.g., with main or regular partners vs. casual partners, physical locations for sex (public settings such as bathrooms vs. private settings), and circumstances in which sex is performed (back alley rush vs. prolonged party weekend vs. at home around sleeping family members). Knowing what women and men do with their sexual partners and the circumstances in which sexual activity happens will allow us to understand how those scenarios affect their willingness to use microbicides and how to better fit microbicide use in that sexual scenario.

The in-depth phone interviews are the richest part of our acceptability assessment. Highly trained qualitative interviewers (male and female so that participants and interviewers can be gender matched) with experience in rectal microbicide research, will interview participants by phone contact with the clinic at which the participant was seen for the last interview. The purpose of this interview is to capture the specific circumstances of each individual's use of the product, in the context of a relationship or lack of it, in different places, times, and the specific cultural frame in which it took place. We want to understand the learning process that occurs in product use and the difficulties that arise; and to record nuances that are not captured in the structured assessments. The interview will be audio-taped and transcribed.

7.11 Pharmacokinetic Procedures

All participants will provide plasma PK samples at 0h (pre-dose) 30mins, 2 h, and 4 h. In addition, all participants will be randomized into either Group A or Group B for specimen-sampling time points. Participants in Group A will be seen for PK sampling on day 1 and day 7 following the single oral dose of TDF 300mg and single rectally applied dose of tenofovir 1% gel or placebo gel, whereas participants in Group B will be seen on day 4 and day 10 following the single dose administration of the oral and rectal study products. Participants will be allowed a 2 day visit window following the originally scheduled PK visit (Please see Table 12 for detailed visit windows). The combination of data from the two Groups will enable insights into compartmental drug concentrations and *ex vivo* efficacy correlations over two weeks from a single dose while minimizing risk.

7.11.1 Pharmacokinetic Procedures: Single Oral Dose

All participants will have blood plasma, PBMCs, and vaginal and rectal fluid obtained before dosing. Additionally, the following PK samples will be taken post-dose:

30 minutes post-dose

- blood plasma (for tenofovir concentrations)
- PBMC tenofovir concentrations (for tenofovir diphosphate concentrations)
- Rectal biopsies: Whole Tissue (for tenofovir and tenofovir diphosphate concentrations)
- Rectal Biopsies: MMC tenofovir levels (for tenofovir diphosphate concentrations)
- Rectal fluid (for tenofovir concentrations)
- Vaginal fluid (in female participants) (for tenofovir concentrations)

2, 4, and 24 hours post-dose

- blood plasma (for tenofovir concentrations)
- PBMCs (for tenofovir diphosphate concentrations)
- Rectal fluid (for tenofovir concentrations)
- Vaginal fluid (in female participants) (for tenofovir concentrations)

Either Days 1 and 7 or Days 4 and 10 (Groups A or B):

- blood plasma (for tenofovir concentrations)
- PBMCs (for tenofovir diphosphate concentrations)
- Rectal biopsies: Whole Tissue (for tenofovir and tenofovir diphosphate concentrations)
- Rectal Biopsies: MMC tenofovir levels (for tenofovir diphosphate concentrations)
- Rectal fluid (for tenofovir concentrations)
- Vaginal fluid (in female participants) (for tenofovir concentrations)

7.11.2 Pharmacokinetic Procedures: Single Rectally Applied Dose

All participants will have blood plasma, PBMCs, and vaginal and rectal fluid obtained before dosing. Additionally, the following PK samples will be taken post-dose:

30 minutes post-dose

- blood plasma (for tenofovir concentrations)
- PBMCs (for tenofovir diphosphate concentrations)
- Rectal biopsies: Whole Tissue (for tenofovir and tenofovir diphosphate concentrations)
- Rectal Biopsies: MMC tenofovir levels (for tenofovir diphosphate concentrations)
- Rectal fluid (for tenofovir concentrations)
- Vaginal fluid (in female participants) (for tenofovir concentrations)

2, 4, and 24 hours post-dose

- blood plasma (for tenofovir concentrations)
- PBMCs (for tenofovir diphosphate concentrations)
- Rectal fluid (for tenofovir concentrations)
- Vaginal fluid (in female participants) (for tenofovir concentrations)

Either days 1 and 7 or days 4 and 10 (Groups A or B):

- blood plasma (for tenofovir concentrations)
- PBMCs (for tenofovir diphosphate concentrations)
- Rectal biopsies: Whole Tissue (for tenofovir and tenofovir diphosphate concentrations)
- Rectal Biopsies: MMC tenofovir levels (for tenofovir diphosphate concentrations)
- Rectal fluid (for tenofovir concentrations)
- Vaginal fluid (in female participants) (for tenofovir concentrations)

7.10.3 Pharmacokinetic Procedures: Following 7-day Rectally Applied Dose

All participants will have blood plasma, PBMCs, and vaginal and rectal fluid obtained before dosing. Additionally, the following PK samples will be taken post-dose:

30 minutes post-dose

- blood plasma (for tenofovir concentrations)
- PBMCs (for tenofovir diphosphate concentrations)
- Rectal biopsies: Whole Tissue (for tenofovir and tenofovir diphosphate concentrations)
- Rectal Biopsies: MMC tenofovir levels (for tenofovir diphosphate concentrations)
- Rectal fluid (for tenofovir concentrations)
- Vaginal fluid (in female participants) (for tenofovir concentrations)

2, 4, and 24 hours post-dose

- blood plasma (for tenofovir concentrations)
- PBMCs (for tenofovir diphosphate concentrations)
- Rectal fluid (for tenofovir concentrations)
- Vaginal fluid (in female participants) (for tenofovir concentrations)

7.12 Laboratory Evaluations

7.12.1 Local Laboratory Testing

Clinical: Safety Bloods

Safety bloods will be done at each site's Clinical Laboratory Improvement Amendments (CLIA) approved labs. These will include complete blood count (CBC) w/Diff and PLT, ALT, AST, Creatinine, calculated Creatinine Clearance, Phosphate, HIV-1 ELISA Ab.

Blood will also be collected for HBsAg, HSV-1, HSV-2, and syphilis serology. If Syphilis RPR is reactive an FTA-ABS serum will be run. Plasma archive will also be collected to confirm HIV serostatus if questions regarding lab results arise after completion of the study.

Clinical: Safety Urine Samples
Safety urine samples will be done at each site's CLIA approved labs. These will include routine urinalysis (protein, glucose, nitrates, and leukocyte esterase), NAAT for GC/CT as well as pregnancy tests for female participants.

Clinical: STI samples
Rectal Swabs for Neisseria *gonorrhoeae* and Chlamydia trachomatis.

Research: Bloods
Research bloods are mainly PK samples (plasma and PBMCs). These will be collected at each site's research laboratory (UCLA, Pittsburgh), prepared as defined in protocol, stored and batched shipped to the MTN Pharmacology Laboratory for quantification (see Laboratory SSP).

Research: Rectal Specimens

Epithelial Sloughing. 50 mL of Normosol® introduced via the endoscope and collected via trap prior to advancing sigmoidoscope for biopsies. Collection and analysis performed at each site.

Rectal Secreted Cytokines
These will be collected from inserted from anorectal swab: collected at each site and isolated per SOP. Samples will be stored for batch processing for Luminex® quantification.

Calprotectin
Samples will be collected at each designated visit at each site and immediately sent to outside laboratory (Genova Diagnostics) from each site for quantification.

Histology
Dedicated biopsy will be prepared at each site, formalin-fixed and paraffin-embedded. Samples will be shipped to UCLA Pathology from both sites for sectioning and quantification by gastrointestinal pathologist

Flow Cytometry
Dedicated biopsies will be received by each site's research laboratory for on-site isolation of mucosal mononuclear cells (MMCs), staining with designated markers and prepared for/analyzed by flow cytometry at each research site.

Rectal Explant Challenge with HIV *ex vivo*
Dedicated biopsies will be collected at each site and explant experiments set up and run for 14 days at each site. Viral titers will be based on common viral stock (sent from UCLA for use at both UCLA and Pittsburgh). All supernatants from explants will be collected, frozen and shipped to UCLA for batch analysis.

Rectal tissue, MMC and fluid samples for PK
Each site will collect rectal sponges for PK to reflect luminal drug concentrations, dedicated rectal biopsies for drug concentration in tissue and dedicated biopsies for isolated of MMCs for quantification of intracellular dug levels. These will be obtained and prepared as designated by the MTN Pharmacology Laboratory, stored frozen and then batch shipped to the MTN Pharmacology Laboratory for processing (see Laboratory SSP).

Rectal Microflora
Samples will be collected by swab at designated visits at each site and samples immediately sent, per SOP, to the MTN Microbiology Laboratory for processing and quantification (see Laboratory SSP).

Research: Vaginal Specimens

Vaginal pH and BV quantification
Samples will be self-collected at designated visits using 2 cotton swabs. One swab will be used to test the pH with a pH indicator strip ranging from 3.6 to 6.1. The second swab will be rolled onto a clean microscope slide, air dried, and sent to the MTN Microbiology Laboratory at the end of the study for Gram stain and assessment for BV using the Nugent criteria.

Vaginal fluid PK
These will be self-collected by participants at each site at designated visits. Each site will elute and prepare the sample per SOP, store frozen and batch ship to the MTN Pharmacology Laboratory for analysis (see Laboratory SSP).

7.12.2 Network Laboratory Testing

Drug Concentration Analysis
All drug-related assays will be performed by an MTN Pharmacology Laboratory. Tenofovir concentrations will be quantified in blood plasma, rectal fluid samples, vaginal fluid samples, and rectal tissue samples. Tenofovir diphosphate concentrations will be quantified in PBMCs, rectal tissue samples, and collagenase-isolated mononuclear cells from rectal tissue samples.

Tenofovir and Tenofovir Diphosphate Analytical Methods
The tenofovir plasma assay uses a combined high performance liquid chromatography-tandem mass spectrometric (HPLC-MS/MS) technique with electrospray ionization on an API4000 mass spectrometer (Applied Biosystems). Analytes and isotopic internal

standards are detected via multiple reaction monitoring (MRM). The assay sensitivity is 0.5 ng/mL and less than 15% coefficient of variation run-to-run. For the intracellular tenofovir diphosphate assay, we use a combined Waters Acquity ultra-performance liquid chromatography-tandem mass spectrometric (UPLC-MS/MS) technique with electrospray ionization on an Applied Biosystems API5000 mass spectrometer also using isotopic internal standards. This direct method does not employ any phosphatase steps, but detects the tenofovir diphosphate moiety to save multiple time consuming column elution steps to isolate the diphosphate moiety, reduce assay variability, and preserve sample mass. The assay limit of detection is 5 fmol/million cells.

Rectal Microflora

The microflora testing will be done at the MTN-Network Laboratory in Pittsburgh. Aerobic and anaerobic organisms will be isolated using conventional culture methods and identified using phenotypic tests. Each organism will be quantified using a semi-quantitative method.

7.13 Specimen Collection and Processing

Each study site will adhere to the standards of good clinical laboratory practice, the HPTN-MTN Network Laboratory Manual (www.mtnstopshiv.org), DAIDS Laboratory Requirements (http://www3.niaid.nih.gov/research/resources/DAIDSClinRsrch/PDF/Lab Policy.pdf), and site standard operating procedures for proper collection, processing, labeling, transport, and storage of specimens at the local laboratories. Specimen collection, testing, and storage at the site laboratories will be documented in the SSP with procedure-specific SOPs. In cases where laboratory results are not available due to administrative or laboratory error, sites are permitted to re-draw specimens that are intended for use in the screening as well as ongoing safety assessments process.

7.14 Specimen Handling

Specimens will be handled in accordance with Requirements for DAIDS Sponsored and/or Funded Laboratories in Clinical Trials (http://www3.niaid.nih.gov/research/resources/DAIDSClinRsrch/Labs/).

7.15 Storage of Specimens for Future Use

The mucosal biopsy samples will be processed for histology, cell isolation and flow cytometry, and RNA isolation. The cells isolated from the gut biopsies will be consumed by the flow cytometry process and there will be no residual cells. The RNA will be used for RT-PCR amplification. After all protocol testing is complete, any residual samples will be stored based on initial consent from the participant. Residual PK samples will be stored at the MTN Pharmacology Laboratory. All other residual samples will be stored at the UCLA MICL Laboratory. If the participant did not give consent to store samples after completion of the study, each site will discard specimens according to institute policy.

7.16 Biohazard Containment

As the transmission of HIV and other blood-borne pathogens can occur through contact with contaminated needles, blood, and blood products, appropriate blood and secretion precautions will be employed by all personnel in the drawing of blood and shipping and handling of all specimens for this study as recommended by the CDC and NIH. All biological specimens will be transported using packaging mandated by Code of Federal Regulations (CFR) 42 Part 72. All dangerous goods materials, including diagnostic specimens and infectious substances, must be transported according to instructions detailed in the International Air Transport Association (IATA) Dangerous Goods Regulations. Biohazardous waste will be contained according to institutional, transportation/carrier, and all other applicable regulations.

8 ASSESSMENT OF SAFETY

8.1 Safety Monitoring

The study site Investigators are responsible for continuous close safety monitoring of all study participants, and for alerting the Protocol Team if unexpected concerns arise. A sub-group of the Protocol Team, including the Protocol co-Chairs, DAIDS Medical Officer (MO), CONRAD MO, UCLA and U Pitt RMP staff, especially including the Regulatory Core (Core B), and Protocol Statistician must be notified. The UCLA RMP Regulatory Core will prepare twice monthly safety data reports for review by the NIH/DAIDS Medical Officer (MO) and CONRAD during the first six months of the study and once a month thereafter. The team will meet, as needed, throughout the period of study implementation to review safety data, discuss product use management and address any potential safety concerns. The content, format and frequency of safety data reports will be agreed upon by the DAIDS Medical Officer and the UCLA RMP Regulatory Core in advance of study implementation.

The DSMB will be comprised of the Chair, who will be an MD/DO, a representative from UCLA and from Pittsburgh/Magee, one of which may be the Chair, CONRAD MO, and a Biostatistical Representative. All members will receive the protocol as well as samples of the reporting formats and the DAIDS Toxicity Tables, including Addendum 3 prior to the trial's start. The DSMB will convene every 6 months if needed, and at the end of the trial. The DSMB will be on call for any emergent concerns.

8.2 Clinical Data Safety Review

A multi-tiered safety review process will be followed for the duration of this study. The study site investigators are responsible for the initial evaluation and reporting of safety information at the participant level, and for alerting the Regulatory Core and the DAIDS MO if unexpected concerns arise. Additional reviews may be conducted at each of these levels as dictated by the occurrence of certain events.

The U19 MDP Regulatory staff (Core B) will review incoming safety data on an ongoing basis. Events identified as questionable, inconsistent, or unexplained will be queried for verification. Adverse event reports requiring expedited handling will be submitted by the Regulatory Core within 3 business days to:

- DAIDS MO
- CONRAD
- Gilead
- Site IRBs
- RMP staff

When indicated, the FDA will be notified through CONRAD (the IND holder).

In addition, accrual will be suspended and the DSMB will be convened if two or more study participants experience an AE ≥ Grade 3. The assembled Data and Safety Monitoring Board will be convened for review as needed. Otherwise, the DSMB will meet every 6 months if needed.

If necessary, external experts representing expertise in the fields of microbicides, biostatistics, HIV transmission and medical ethics may be invited to join the DSMB safety review. A recommendation to stop the trial may be made by the DSMB at this time or at any such time that the team agrees that an unacceptable type and/or frequency of AEs has been observed.

Recommendations regarding permanent discontinuation of one or both study products in the study as a whole may involve sponsor consultation with the US Food and Drug Administration (FDA).

In the unlikely event that the protocol team or DSMB has serious safety concerns that lead to a decision to permanently discontinue study products for all participants and stop accrual into the study, the protocol team or DSMB will request a review of the data by the DAIDS Medical Officer before recommending that the study be stopped. If at any time, a decision is made to discontinue one or more study products in all participants, DAIDS will notify CONRAD who will notify the US FDA and the site investigators of record will notify the responsible IRBs expeditiously.

8.3 Adverse Events Definitions and Reporting Requirements

8.3.1 Adverse Events

An AE is defined as any untoward medical occurrence in a clinical research participant enrolled in a clinical trial, and does not necessarily have a causal relationship with an investigational product or study participation. As such, an AE can be an unfavorable or unintended sign (including an abnormal laboratory finding, for example), symptom or disease temporally associated with the use of an investigational product or study participation, whether or not considered related to the product or study participation.

This definition will be applied beginning from the time of random assignment. The term “investigational product” for this study refers to the applicator, TDF tablets, tenofovir 1% gel and HEC Placebo Gel.

Study participants will be instructed to contact the study site staff to report any AEs they may experience at any time between enrollment and completion of their participation. In the case of a life-threatening event, they will be instructed to seek immediate emergency care. Where feasible and medically appropriate, participants will be encouraged to seek medical care where the study clinician is based, and to request that the clinician be contacted upon their arrival. With appropriate permission of the participant, whenever possible records from all non-study medical providers related to AEs will be obtained and required data elements will be recorded on study case report forms. All participants reporting an AE will be followed clinically until the AE resolves (returns to baseline) or stabilizes.

The site IoR will determine AE resolution or stabilization in their best clinical judgment, but may seek DAIDS MO and/or DSMB medical consultation regarding follow up or additional evaluations of an AE. Study site staff will report on study case report forms all AEs reported by or observed in enrolled study participants from the time of enrollment (random assignment) until study termination, regardless of severity and presumed relationship to study product. The DAIDS AE Grading Table Version 1.0, December 2004, Addenda 1 and 3 (Female Genital and Rectal Grading Tables for Use in Microbicide Studies) will be the primary tool for grading adverse events for this protocol. Adverse events not included in that table will be graded by the DAIDS AE Grading Table, Version 1.0 December 2004. In cases where an AE is covered in multiple tables, Addendum 3 (Rectal Grading Table for Use in Microbicide Studies) will be the grading scale utilized.

Even though sexual abstinence is a requirement during active portions of the trial, participants will be encouraged to report to the study clinician any problems experienced by their partners that might be potentially related to study product. If any such problems are reported, study staff should evaluate and document the occurrence. Should any concerns arise with regard to partner safety, the IoR and the RMP Regulatory Core will advise all study sites on appropriate action.

8.3.2 Serious Adverse Events

Serious adverse events (SAEs) will be defined per CFR 312.32, as AEs occurring at any dose that:

- Results in death
- Is life-threatening
- Results in persistent or significant disability/incapacity
- Is a congenital anomaly/birth defect.
- Requires inpatient hospitalization or prolongation of existing hospitalization

Note: Per ICH SAE definition, hospitalization itself is not an adverse event, but is an outcome of the event. Thus, hospitalization in the absence of an adverse event is not regarded as an AE, and is not subject to expedited reporting. The following are examples of hospitalization that are not considered to be AEs:

- Protocol-specified admission (e.g. for procedure required by study protocol)
- Admission for treatment of target disease of the study, or for pre-existing condition (unless it is a worsening or increase in frequency of hospital admissions as judged by the clinical investigator)
- Diagnostic admission (e.g. for a work-up of an existing condition such as persistent pretreatment lab abnormality)
- Administrative admission (e.g. for annual physical)
- Social admission (e.g. placement for lack of place to sleep)
- Elective admission (e.g. for elective surgery)

Important medical events that may not result in death, be life-threatening, or require hospitalization may be considered a serious adverse drug experience when, based upon appropriate medical judgment, they may jeopardize the patient or subject and may require medical or surgical intervention to prevent one of the outcomes listed above.

8.3.3 Adverse Event Relationship to Study Product

The relationship of all AEs to study product will be assessed per the Manual for Expedited Reporting of Adverse Events to DAIDS (dated 6 May 2004), the tenofovir gel investigator's brochure, the Viread® package insert, HEC Placebo Gel investigator's brochure, and clinical judgment. Per the Manual for Expedited Reporting of Adverse Events to DAIDS, the relationship categories that will be used for this study are:

- *Definitely related*: adverse event and administration of study agent are related in time, and a direct association can be demonstrated with the study agent

- *Probably related*: adverse event and administration of study agent are reasonably related in time, and the adverse event is more likely explained by the study agent than by other causes

- *Possibly related*: adverse event and administration of study agent are reasonably related in time, and the adverse event can be explained equally well by causes other than the study agent

- *Probably not related*: a potential relationship between administration of study agent and adverse event could exist, but is unlikely, and the adverse event is most likely explained by causes other than the study agent

- *Not related*: the adverse event is clearly explained by another cause unrelated to administration of the study agent. Reportable events must have documentation to support the determination of "not related"

8.4 Expedited Adverse Event Reporting Requirements

Expedited Adverse Event (EAE) Reporting

The adverse events that must be reported in an expedited fashion include all serious adverse events (SAEs) as defined by the May 1996 International Conference on Harmonisation of Technical Requirements for Registration of Pharmaceuticals for Human Use (ICH), Good Clinical Practice: Consolidated Guidance (E6) regardless of relationship to the study agent(s). Important medical events that may not be immediately life-threatening or result in death or hospitalization but may jeopardize the patient or may require intervention to prevent one of the outcomes listed in the definition above may also be considered to be serious.[83]

For all SAEs submitted, sites must file an initial and an update to CONRAD and the DAIDS Medical Officer with the final or stable outcome unless the initial EAE submitted had a final or stable outcome noted already.

EAE Reporting Requirements for this Study

Any adverse event that is determined to be serious (whether expected or unexpected) regardless of relationship to the study agent(s) must be immediately reported to CONRAD and the DAIDS Medical Officer (21 CFR 312.64). An Expedited Adverse Event (EAE) Form must be completed and sent to CONRAD and the DAIDS Medical Officer within 3 business days (by 5 PM Eastern Time (ET)) after site awareness that the event has occurred at a reportable level. DAIDS MO will review and discuss the EAE report with CONRAD to address any concerns.

CONRAD will then notify the FDA of any unexpected serious adverse events associated with the use of the drug as soon as possible, but no later than 7 calendar days after initial receipt of the information from the investigator.

For unexpected serious adverse events associated with the use of the drug, CONRAD will submit the safety reports provided by the sites to the IND no later than 15 calendar days after the initial receipt of the information and send copies of the submission to the DAIDS MO, the RCC (to be placed in the file) and Gilead.

Study Agents for Expedited Reporting

The study agents that must be considered in determining relationships of AEs requiring expedited reporting to CONRAD and the DAIDS MO are: tenofovir disoproxil fumarate 300 mg tablet, tenofovir 1% gel, HEC placebo gel, and study gel applicator.

Grading Severity of Events

The Division of AIDS Table for Grading the Severity of Adult and Pediatric Adverse Events, Version 1.0, Dec 2004, Addenda 1 and 3 (Female Genital and Rectal Grading Tables for Use in Microbicide Studies) will be the primary tools for grading adverse events for this protocol. Adverse events not included in those tables will be graded by the DAIDS AE Grading Table Version 1.0, December 2004. In cases where an AE is covered in all tables, the DAIDS AE Grading Table, Version 1.0, Dec 2004, Addendum

3 (Rectal Grading Table for Use in Microbicide Studies) will be the grading scale utilized.

The Division of AIDS Table for Grading the Severity of Adult and Pediatric Adverse Events (DAIDS AE Grading Table), Version 1.0, December, 2004, and Addenda 1 and 3 are available on the RCC website at http://rcc.tech-res.com/eae.htm.

EAE Reporting Periods

SAEs (as defined above) must be reported on an expedited basis during the Protocol-defined EAE Reporting Period, which is the entire study duration for an individual participant (from study enrollment until the Final Clinic Visit/Early Termination Visit).

For each study participant, expedited AE reporting will be undertaken throughout the scheduled duration of follow-up, i.e., from the time of random assignment through completion of the Final Study Visit. In addition, should site staff become aware of any serious, unexpected, clinical suspected adverse drug reactions after the Final Study/Early Termination Visit, such events also will be expeditiously reported.

8.5 Pregnancy and Pregnancy Outcomes

Pregnant subjects are excluded from this study. Urine testing is performed at Visits 1, 2, 3, 7, 11, and as indicated at interim visits. If subjects become pregnant at any time during the course of the study, study agents are discontinued, but subjects will remain in the study and will continue with these assessments: blood tests, UA, and acceptability.

Pregnancy-related data will be collected using the pregnancy CRFs for all pregnancies detected during the study. Pregnancy outcomes will not be expeditiously reported to DAIDS unless there is an associated adverse event in the pregnant subject that meets expedited reporting criteria or the pregnancy results in a congenital anomaly meeting ICH guidelines for expedited reporting. Fetal losses without congenital anomalies or maternal complications that require expedited reporting will not be expeditiously reported, but data will be captured via the pregnancy CRFs.

After the Final Study Visit, pregnancy outcomes that meet criteria for expedited adverse reporting as described above (e.g., maternal complications, congenital anomalies) occurring among participants known to be pregnant at the Final Study Visit will continue be expeditiously reported.

8.6 Social Harms Reporting

Although study sites make every effort to protect participant privacy and confidentiality, it is possible that participants' involvement in the study could become known to others, and that social harms may result (i.e., because participants could become known as HIV-infected or at "high risk" for HIV infection). For example, participants could be treated unfairly or discriminated against, or could have problems being accepted by their families and/or communities. Social harms that are judged by the Investigator of

Record to be serious or unexpected will be reported to responsible site IRB at least annually, or according to their individual requirements. In the event that a participant reports social harm, every effort will be made by study staff to provide appropriate care and counseling to the participant, and/or referral to appropriate resources for the safety of the participant as needed. While maintaining participant confidentiality, study sites may engage their Community Advisory Boards in exploring the social context surrounding instances of social harm.

9 CLINICAL MANAGEMENT

Guidelines for clinical management and product hold/discontinuation are outlined in this section.

In general, the site investigator has the discretion to hold study product at any time if s/he feels that continued product use would be harmful to the participant, or interfere with treatment deemed clinically necessary according to the judgment of the investigator. Unless otherwise specified below, the investigator should immediately consult the DAIDS MO and/or the DSMB medical members for further guidance in restarting study drug(s) or progressing to permanent discontinuation.

9.1 Grading System

The Division of AIDS Table for Grading the Severity of Adult and Pediatric Adverse Events, Version 1.0, Dec 2004 and Addenda 1 and 3 (Female Genital and Rectal Grading Tables for Use in Microbicide Studies) will be used in this study.

These tables are available on the Regulatory Compliance Center (RCC) Web site: http://rcc.tech-res.com/eae.htm.

9.2 Discontinuation of Study Product(s) in the Presence of Toxicity

Grade 1 or 2
In general, participants who develop a Grade 1 or 2 adverse event regardless of relatedness to study product may continue use of both study products per protocol.

Grade 3
Participants who develop a Grade 3 adverse event or toxicity that is judged to be possibly, probably, or definitely related to study product should have that study product held. In general, and unless otherwise decided in consultation with the DSMB Medical members and the DAIDS MO, the investigator should re-evaluate the participant at least weekly up to 2 weeks. If documentation is not available within 2 weeks to show that the adverse event is Grade 2 or less, the current study product must be permanently discontinued for that participant.

If the same Grade 3 adverse event recurs after reintroduction of study product, the current study product must be permanently discontinued if the investigator considers the adverse event probably not, possibly, probably, or definitely related to study product. However if the investigator determines that the toxicity is definitely not related to study product, participants may continue the study product and the DSMB and DAIDS MO must be notified.

Grade 4
Participants who develop a Grade 4 adverse event or toxicity (regardless of relationship to study product(s)) should have the current study product(s) held. If the investigator determines that the toxicity is definitely not related to study product(s), DSMB and DAIDS MO must be consulted to consider restarting study product(s), but product(s) should be held until a recommendation is obtained. The participant should be re-evaluated at least weekly up to 2 weeks. If documentation is not available within 2 weeks to show that the adverse event is Grade 2 or less, the study product(s) must be permanently discontinued. If the same Grade 4 adverse event recurs at either Grade 3 or Grade 4 level after reintroduction of study product(s), study product(s) must be permanently discontinued.

9.3 General Criteria for Discontinuation of Study Product

Participants may voluntarily discontinue use of study gel for any reason at any time. Site IoRs will temporarily hold or permanently discontinue participants from the study gel per protocol for any of the specific criteria below, which may be further clarified in the SSP. Site IoRs also may temporarily hold or permanently discontinue participants from study products for use of prohibited medication (per Section 6.6.1), for reasons not shown here or in the SSP, e.g., to protect participant safety and/or if participants are unable or unwilling to comply with study product use procedures. In such cases, the Site IoRs would temporarily hold product use and provide a written query with a request for permanent study product discontinuation to the DAIDS MO for review. The MO will provide a written response to the site indicating whether s/he has recommended permanent discontinuation of study product(s). Such recommendations regarding permanent discontinuation of study products in individual participants will be made based on careful review of all relevant data.

The criteria for permanent discontinuation of further study product use of one or both study products for an individual participant are:

- Study product-related toxicity requiring permanent discontinuation of study product(s) per Section 9 of this protocol
- Completion of regimen as defined in the protocol
- Request by participant to terminate study product(s)
- Clinical reasons determined by the physician
- HIV infection
- Pregnancy or breastfeeding

9.4 Management of Specific Toxicities

Specific guidance related to product hold is also noted here as it pertains to the clinical management of toxicities.

9.4.1 Hemorrhage following rectal mucosal biopsy

Mucosal bleeding will be directly observed until stopped or stopped with subepithelial injection of diluted epinephrine (standard of care) prior to the end of the biopsy procedure. However if bleeding continues after the procedure that results in the passage of blood clots per rectum and/or continues for more than 3 days and/or is of concern to the participant, the participant will be referred for assessment in the emergency department of the nearest hospital.

9.4.2 Infection following rectal mucosal biopsy

The rate of local or systemic infection following mucosal biopsy is exceedingly low. Any participant presenting with local or systemic features compatible with infection (fever, localized anorectal pain, anal discharge) will be referred to the emergency department of the nearest hospital.

9.4.3 Perforation of rectum following rectal mucosal biopsy

The rate of perforation of a hollow viscus following endoscope biopsy is less than 0.88:1,000.[84] Any participant presenting with local or systemic clinical features suggestive of this condition (abdominal pain, swelling, fever) will be referred to the emergency department of the nearest hospital.

9.5 Clinical Management of Pregnancy

All study participants are required to be using an effective method of contraception according to Section 5.2 at enrollment, and intending to use same method for the duration of study participation. Study staff will provide contraceptive counseling to enrolled participants as needed throughout the duration of study participation and will facilitate access to contraceptive services through direct service delivery and/or active referrals to local service providers.

Participants will be encouraged to report all signs or symptoms of pregnancy to study staff. The site IoR or designee will counsel any participants who become pregnant regarding possible risks to the fetus according to site-specific SOPs. The IoR or designee also will refer the participant to all applicable services; however, sites will not be responsible for paying for pregnancy-related care.

Participants who are pregnant at the termination visit will continue to be followed until the pregnancy outcome is ascertained (or, in consultation with the MO, it is determined that the pregnancy outcome cannot be ascertained). Pregnancy outcomes will be

reported on relevant case report forms; outcomes meeting criteria for EAE reporting also will be reported on EAE forms.

NOTE: Participants who become pregnant during the course of the study will discontinue permanently all study product(s).

9.6 Criteria for Early Termination of Study Participation

Participants may voluntarily withdraw from the study for any reason at any time. Site IoRs may, with the approval of the DSMB withdraw participants before their scheduled termination visit to protect their safety, and/or if participants are unable or unwilling to comply with study procedures. Participants also may be withdrawn if the study sponsors, government or regulatory authorities (including the Office for Human Research Protections (OHRP)), or site IRBs terminate the study prior to its planned end date. Site investigators are required to consult the Protocol Chair and Protocol Biostatistician prior to the termination of any study participant. Study staff will record the reason(s) for all withdrawals in participants' study records. In the event that participants who voluntarily withdraw from the study wish to re-join the study during their planned 21-week follow-up period, they may resume study procedures and follow-up at the investigator's discretion.

10 STATISTICAL CONSIDERATIONS

10.1 Overview and General Design

This will be a two-site, partially-blinded, placebo controlled study in 18 participants of vaginally-formulated tenofovir 1% gel applied topically to the rectum with detailed PK studies comparing multi-compartment tenofovir concentrations (both single and 7-day dosing) within each participant and contrasting these values with single dose oral tenofovir tablets within each participant. This design will enable within-participant, within- and between- group analyses. Safety of the topical gel applications, detailed mucosal immunotoxicity of the topically-applied products and *ex-vivo* efficacy using mucosal explants will be assessed.

10.2 Study Endpoints

Primary Endpoints:

- Grade 2 or higher clinical and laboratory adverse events as defined by the Division of AIDS Table for Grading the Severity of Adult and Pediatric Adverse Events, Version 1.0, Dec 2004 and Addenda 1 and 3 (Female Genital and Rectal Grading Tables for Use in Microbicide Studies) to this table

Secondary Endpoints:

- Mucosal assays
  - Fecal calprotectin
  - Microflora (see Appendix V)
  - Rectal cytokines (secreted)
  - Rectal epithelial sloughing
  - Rectal histology
  - Rectal CD4 cell phenotype/activation
- Tenofovir levels
  - Plasma
  - PBMC (intracellular)
  - Rectal fluid
  - Vaginal fluid
  - Rectal mucosal tissue homogenates
  - Rectal mucosal mononuclear cells
- Tenofovir diphosphate concentrations
  - PBMC
  - Rectal mucosal tissue homogenates
  - Rectal mucosal mononuclear cells

- The proportion of participants who at their Final Clinic Visit (Visit 13) report via the acceptability questionnaire that they would be very likely to use the candidate microbicide during receptive anal intercourse.

Exploratory Endpoints

- Changes in HIV-1 p-24 levels in colorectal explant supernatant

10.3 Study Hypotheses

- Vaginally-formulated Tenofovir 1% topical gel when applied rectally will be safe using a combination of clinical and laboratory markers including assays specifically designed to measure mucosal toxicity

- Tenofovir will be detectable at different concentrations in the various anatomic compartments sampled for pharmacokinetics following single and 7-day topical exposures

- Exposure to tenofovir will demonstrate relative suppression of *ex vivo* HIV-1 infectibility using *in-vivo* drug-exposed tissue as compared to baseline tissue samples

- Orally delivered, single dose, 300 mg Tenofovir tablets will have similar safety profiles using routine blood safety indices as have been established in other trials and will show no mucosal safety concerns

- The oral dose will have different multi-compartment concentration kinetics than the topical tenofovir and will also demonstrate preliminary (*ex vivo)* suppression using the explant infectivity assay

- Vaginally formulated tenofovir 1% topical gel applied rectally will be acceptable to participants, as indicated by a score in the upper one third of the 10-point Likert scale on intentionality to use in the product in the future

10.4 Sample Size

There will be a total of 18 participants, men and women, enrolled at two clinical sites (UCLA, U Pitt). Participants will be HIV-1 seronegative, generally healthy and at least 18 years of age. While the intent will be to enroll equally at both sites, the protocol specifies that recruitment may take place at either site to complete. Based on the prior rectal microbicide trial (RMP-01: UC781), it is anticipated that at least one third of enrolled participants will be women.

10.5 Randomization Procedures

Enrolled participants will undergo two separate randomization procedures for the study as a whole. The two randomization procedures are as follows:

(i) randomized to receive tenofovir 1% gel rectally versus HEC placebo at a 2:1 ratio (drug to placebo); this will apply to the topical gel section of the trial (single and 7-day exposure) only

(ii) randomized to be in sample acquisition Group A (samples acquired at Day 1 and Day 7 post exposure) or Group B (samples acquired at Day 4 and Day 10 post exposure); this will apply to the detailed sampling protocol following single dose oral and single dose topical exposure

For part (i), randomization will be done in blocks of size 3, and each site will enroll participants in multiples of 3. This will ensure balance between control and tenofovir groups between sites. For part (ii), a separate randomization of subjects will be done overall, ensuring equal numbers of controls in Groups A and B, and equal number of tenofovir experimental subjects in Groups A and B – however there will be no guarantee of balance between sites. Randomization codes will be assigned by the Data Management and Biostatistical Core of the MDP/RMP (Core C), based at UCLA under the direction of Dr. William Cumberland. Codes and their actual assignment will be delivered to the manufacturer, who will label the products sequentially starting with 101 at Los Angeles and 201 at Pittsburgh. Each numeric code will also have the designation "A" or "B" indicating which group the subject belongs to. Because of the uncertainty of the actual number of subjects that can be enrolled at each site, 18 labeled packets will be created for each site. Once a total (for both sites) of 9 participants have been enrolled, an assessment and projections of total accrual at planned study end will be made using observed accrual rates at each site, after which a new target total will be

calculated. For example if 2 participants have been enrolled at Pittsburgh and 7 at UCLA, new targets are 18(2/9)=4 and 18(7/9)=14. Since we are using blocks of size 3 these will be rounded to Pittsburgh:3 and UCLA:15 or to Pittsburgh:6 and UCLA:12. The final decision on new targets will be made by the site investigators. After adjustment, the number of subjects enrolled at each site will be a multiple of three, but could be as low as 0 and as high as 18 at one site.

10.6 Participant Accrual, Follow-up, Retention, and Replacement

Recruitment will occur via four main strategies:

- Clinician-patient referrals
- Use of existing "study registries" that contain the names and phone numbers of individuals who have given informed consent to be reached for future studies for which they may be eligible
- Participant referrals (participants refer their friends or partners who may meet eligibility criteria)
- Passive self-referral: interested individuals see a study poster or brochure advertising the study and call the study site directly

Study staff will meet as needed to discuss current recruitment status, targets, and strategies. Staff also will follow-up with all persons who express an interest in the study to ensure that screening appointments are scheduled and carried out in a timely manner.

Participant Retention

Once a participant enrolls in this study, the study site will make every effort to retain him/her for the duration of follow-up in order to minimize possible bias associated with loss-to-follow-up. The study staff is responsible for developing and implementing local standard operating procedures to target this goal. Components of such procedures include:

- Thorough explanation of the study visit schedule and procedural requirements during the informed consent process, and re-emphasis at each study visit
- Thorough explanation of the importance of all three treatment phases to the overall success of the study
- Use of appropriate and timely visit reminder mechanisms (via email and/or telephone)
- Immediate and multifaceted follow-up on missed visits

Participant Withdrawal

Regardless of the participant retention methods just described, participants may voluntarily withdraw from the study for any reason at any time. The Investigator also may withdraw participants from the study in order to protect their safety (e.g. pregnancy, diagnosis of colon cancer, ulcerative colitis) and/or if they are unwilling or unable to comply with required study procedures. Participants also may be withdrawn if the IRB,

study sponsor, government or regulatory authorities terminate the study prior to its planned end date.

Every reasonable effort will be made to complete a final evaluation (as described in Section 7.5) of participants who terminate from the study prior to the final visit, and study staff will record the reason(s) for all withdrawals from the study in participants' study records. Participants who withdraw or are withdrawn prior to receiving study product will be replaced. Participants who withdraw or are withdrawn after receiving study product will not be replaced.

10.7 Data Monitoring and Analysis

10.7.1 Emergency Unblinding Procedures

Subjects may be unblinded for safety or medical treatment purposes. The investigator must, whenever possible, phone the study's designated DSMB medical monitor to unblind the code. The medical monitor will notify the investigator and sponsors that a subject was unblinded without revealing what the treatment assignment was. Appropriate documentation must be submitted to the investigator, MTN, CONRAD, Gilead and the DAIDS MO within 3 working days of unblinding. Any subject that is unblinded must be discontinued from the study.

10.7.2 Primary Analysis

Each participant will serve as their own control with baseline readouts as well as contribute to group comparisons (active product versus placebo). Participants will have sets of study samples acquired four times - once during the screening visits (Visit 1), once for baseline studies (Visit 2), one set following oral dosing (Visits 3-6), one set following single rectal exposure (Visits 7-10) and one set following 7-day exposure (Visit 12) - with a final telephone safety follow-up.

<u>Safety Data</u>
The clinical safety AE reporting will be per the DAIDS AE Grading Table, Version 1.0, December 2004, Addenda 1 and 3 (Female Genital and Rectal Grading Tables for Use in Microbicide Studies). The frequency of $\geq$ Grade 2 adverse events will be used as the primary measure of safety of tenofovir 1% gel and TDF 300 mg. Because of the relatively small sample size, only large differences between placebo and study drug groups will be detectable. For example, assuming a 2% rate in the placebo group (6 participants) and using a one-sided test with alpha=0.10, the power for a Fisher's Exact Test of no difference achieves a power of only .20 when the rate in the active group (12 participants) is 28%, a power of .30 when the active group rate is 32%, and a achieves a power of .80 when the active group rate is 54%. Because we need to be able to determine that excessive AEs are occurring at lower rates in the active group than this, we plan to simply count the number of AEs occurring in a group of 18 participants (for the oral part of the study) and in a group of 12 participants (for the topical part of the study). Table 23 shows for selected true AE rates between .01 and .20 the probability of

one or more, two or more, and three or more AEs occurring in a sample of 18; Table 24 shows the same for a sample of 12. From these tables we can see that we have a probability >.90 of observing one or more AE's when the true rate is >12% in 18 subjects, and a probability >.78 when the true rate is >12% in 12 subjects. We have also included Tables 25 and 26 which give exact 95% confidence intervals for the true rate for selected numbers of AE events from 0 to 5. When the adverse event represents an exacerbation of a baseline condition, the adverse event will be reported as the change in grade from baseline. Serious adverse events will be reported as such, regardless of the baseline condition.

Table 23: Probability of Events, Selected True Rates of AEs (n=18, oral study)

True rate	Zero events	One or more events	Two or more events	Three or more events
**.01**	.835	.165	.014	.001
**.02**	.695	.305	.050	.005
**.03**	.578	.422	.100	.016
**.04**	.480	.520	.161	.033
**.05**	.397	.603	.226	.058
**.06**	.328	.672	.294	.090
**.07**	.271	.729	.362	.127
**.08**	.223	.777	.428	.170
**.09**	.183	.817	.491	.217
**.10**	.150	.850	.550	.266
**.11**	.123	.877	.604	.317
**.12**	.100	.900	.654	.369
**.13**	.082	.918	.699	.421
**.14**	.066	.934	.740	.471
**.15**	.054	.946	.776	.520
**.16**	.043	.957	.808	.567
**.17**	.035	.965	.836	.612
**.18**	.028	.972	.861	.654
**.19**	.023	.977	.882	.693
**.20**	.018	.982	.901	.729

Table 24: Probability of Events, Selected True Rates of AEs (n=12, topical study)

True rate	Zero events	One or more events	Two or more events	Three or more events
**.01**	.886	.114	.006	.000
**.02**	.785	.215	.023	.002
**.03**	.694	.306	.049	.005
**.04**	.613	.387	.081	.011
**.05**	.540	.460	.118	.020
**.06**	.476	.524	.160	.032
**.07**	.419	.581	.203	.047
**.08**	.368	.632	.249	.065
**.09**	.322	.678	.295	.087
**.10**	.282	.718	.341	.111
**.11**	.247	.753	.387	.138
**.12**	.216	.784	.431	.167
**.13**	.188	.812	.475	.198
**.14**	.164	.836	.517	.230
**.15**	.142	.858	.557	.264
**.16**	.123	.877	.595	.299
**.17**	.107	.893	.630	.334
**.18**	.092	.908	.664	.370
**.19**	.080	.920	.696	.406
**.20**	.069	.931	.725	.442

Table 25: Exact 95% Confidence Bounds for True Rates of AEs (n=18, oral study)

Number of events	Estimated rate	Lower bound	Upper bound
**0**	.000	.000	.185
**1**	.056	.016	.273
**2**	.111	.053	.347
**3**	.167	.097	.414
**4**	.222	.143	.476
**5**	.278	.191	.535

Table 26: Exact 95% Confidence Bounds For True Rate of AEs (n=12, topical study)

Number of events	Estimated rate	Lower bound	Upper bound
**0**	.000	.000	.265
**1**	.083	.024	.385
**2**	.167	.080	.484
**3**	.250	.146	.572
**4**	.333	.216	.651
**5**	.417	.290	.723

Mucosal Damage Parameters

Immunotoxicity Assays

Interpretive power for the immunotoxicity assays will be based on 18 before-after comparisons in the setting of oral dosing, and on 12 before-after comparisons in the single rectal dosing and 7-day rectal dosing. We will not compare the changes in the 12 active subjects with changes in the 6 controls, as the power for such a comparison is

extremely low. Because most of these differences using UC781 in the just completed RMP-01 trial showed approximately normal distributions of adverse events, testing for dramatic changes in these variables will be done using paired t-tests on the differences between the two visits. This would be (i) Visit 2 compared to Visit 3; (ii) Visit 2 compared to Visit 7 and (iii) Visit 2 compare to Visit 12.

Because of the small number of participants planned, assessments to look for changes will be done using two-sided tests with α=.10. This assures high power for detecting marked changes, at the cost of increased Type I error probabilities. Hence, any significant results will need to be followed with confirmatory studies and discussion of their clinical relevance. Assuming normally distributed differences, and a paired t-test with a sample size of 12 topical tenofovir participants, there will be 83% power to detect an effect size of 0.8, and 94% power to detect an effect size of 1. To convert an effect size, f, into measured units, we multiply the derived standard deviations for each assay change by f. Thus, for example, based on the results from the RMP-01 UC781 trial, there was a 98% power to detect an average decrease of 6.34 in CD 4-lymphocytes, and higher power for larger differences. This approach will provide sufficient power to detect any changes that we currently believe will be scientifically important.

The associations of five sets of mucosal parameters with the use of study product will be examined as a secondary objective. Five of these parameter sets (Epithelial sloughing, Histopathology, Cell Phenotype, Cytokine profile, Fecal Calprotectin) are collected multiple times per participant: at baseline, pre- and/or post-single oral treatment, pre- and/or post single rectal treatment and pre- and/or post-7 day rectal treatment. For all five parameter panels, the main question of interest is whether the treatment groups (placebo, drug) have systematically different mucosal damage parameters post-single and post 7 day treatments. In particular, the tenofovir gel treatment groups will be assessed to determine if they have evidence of more mucosal damage than the placebo group.

As subjects will be randomized, systematic baseline differences are not expected; however, due to the relatively small study size, it is quite likely that many parameters will be quite different between treatment groups at baseline. All analyses will therefore be conducted both with and without controlling for baseline measurements. For continuous measures, it is well-known that controlling for baseline differences in regression models is more powerful than analyzing changes from baseline.

The basic model for analyzing continuous measures adjusting for baseline differences will be the analysis of covariance (ANCOVA) model regressing the continuous parameter at either post-single oral, post-single rectal or post 7 day rectal treatment on the continuous baseline value and the categorical treatment group membership (with placebo as the reference group). The estimated regression coefficients for the treatment group will then be directly interpretable as the difference between drug versus placebo, after adjusting for baseline differences. This model without the baseline values reduces to the simple one-way analysis of variance (ANOVA) model with treatment group as the predictor. For analyzing binary or ordinal measures, we will use the

logistic regression version of these models. For dichotomous outcomes, we plan to use ordinary logistic regression. For ordinal outcomes, we will use a proportional odds model – which is often referred to as ordinal logistic regression.

As we will be analyzing group differences in a large number of parameters, the question of whether to adjust for multiple hypothesis testing arises. Because the effect of these adjustments is to make it more difficult to find a significant result, and because the safety assessments are made on a small number of subjects, we will not use formal adjustments for the safety part of the trial. For the comparisons of p-24 antigen in the explant studies, we will address this multiple comparisons problem informally by considering differences significant at the 0.05 level or smaller to be flagged as at least suggestive and those with extremely strong p-values (e.g. 0.001 or less) will be carefully examined

We will also investigate longitudinal (three or four time points) and/or multivariate (simultaneous examination of multiple measures in a set) modeling techniques. The longitudinal analyses will most likely not be additionally informative, as there are only three time points for most of the parameters. Multivariate techniques are expected to result in potentially substantial power increases. Our previous studies with phenotype and cytokine panels have shown strong correlations between measures as well as good stability over time (i.e. high intra-subject correlations) in steady state for many of the measures.

<u>Epithelial Sloughing</u>
The simplest analysis for the epithelial sloughing measurements will look at the presence or absence of sloughing. Additionally, we may employ the five point ordinal scale [77,78] which ranges from 0 (no evidence of sloughing) up to 4 (evidence in all four quadrants). For these data, there is baseline measurement will allow screening out of post-treatment false positives.

<u>Histopathology</u>
As in the sloughing data, we will begin by examination of a two point (normal versus abnormal) or three point (normal, slightly abnormal, abnormal) scales. Based on initial analyses of the HPTN 056[57] histopathology panel data, it is not expected that quantitative measures will be substantially more informative than simple qualitative readings.

<u>Mucosal mononuclear cell phenotype</u>
Many of the flow cytometry measurements from the HPTN 056 study are quite stable across time.[57,79,80] Parameters with high intra-subject correlations (RFI measures are log-transformed) include %CD3, %CD4, %CD8, %CD31, CD38RFI on CD4+, CD38RFI on CD8+, %CD38||CD4, and %CD38||CD8 which have intra-subject correlations of between 0.7 and 0.9. CCR5-related parameters had moderate stability (intra-subject correlations of around 0.5).

Mucosal cytokine Profile
The cytokine data in HPTN 056 and earlier work of our group[57] for RANTES, IFN-γ, and IL-10 all showed strong stability (intra-subject correlations between 0.7 or 0.8; analysis is on log-transformed scale for all three of these).

Fecal calprotectin
The fecal calprotectin marker has been shown to be extremely sensitive for discriminating several clinical conditions such as active and inactive Crohn's disease[81,82]. The large majority of measurements for inactive subjects were below normal thresholds and nearly all measurements for active subjects were above normal thresholds. For this study, this suggests that this marker will be particularly helpful in detecting evidence of mucosal damage in the treatment groups.

Statistical Procedures for Microflora Studies
Microflora measures will be graded on a 0 to 4 ordinal scale and recorded at baseline and post-exposure. Depending on the empirical distributions across the points in this scale, statistical procedures will either involve analysis of the actual pre-post differences (ordinal) or dichotomized versions of the pre and post scores (binary). In the ordinal case, we will use one sample and multi-sample signed rank tests to examine whether (1) there is an overall change in microflora levels and (2) whether the treatment groups differ significantly from each other in pre-post change. Similarly, if dichotomization is more appropriate, then the binary baseline and post-exposure data will be analyzed using exact McNemar tests (to examine if levels change significantly pre to post) and Fisher tests (to examine whether the pre and post prevalence levels differ between groups).

Explant Studies
The main question of interest for the explant studies is whether the oral and or topical tenofovir treatment groups have reduced infectivity post-treatment. As virus growth varies according to the day of observation, comparisons will be made when exponential virus growth ("soft endpoint") is achieved using an improved statistical method developed for the Microbicide Quality Assurance Program (MQAP-NICHD).[85] Optical Density (O.D.) data from all p24 assays will be compared to a universal standard curve for O.D. values within a + 95% confidence interval of the plate standards. Differences between experimental groups will be determined using Repeated Measures ANOVA and ANCOVA (adjusting for baseline differences).

Changes in cumulative p-24 antigen will be the primary outcome in the explant studies. Unlike the situation in the immunotoxicity portion of this study, the design here is for efficacy. Consequently, a more conservative two-sided paired t-test with α=.05 will be used. Only one formulation will be used (1%) and only one site (10cm) will be studied, using one viral titer (10^4 $TCID_{50}$). Our previous UC781 study at 10cm showed effect sizes of 1.4 for 0.25% at 10^4 $TCID_{50}$ (corresponding to an average difference of 5240) and an effect size of .8 for 0.1% at 10^4 $TCID_{50}$ (corresponding to an average difference of 1110). However, only roughly 2/3 of those 36 participants infected at baseline with 10^4 titer were infectable with 10^2 viral titer. Given the 12 topical tenofovir participants and the 6 controls here, only the high viral titer will be used in this trial. Using a two-

sided paired t-test with α=.05, we will have 70% power for an effect size of .80 and 99% power for an effect size of 1.4. Comparisons between the control and experimental groups will be done using multi-level models, but given the extremely small sample sizes, this will only be done for powering further studies, as there will not be sufficient power to detect a difference in changes, except for very extreme outcomes.

Pharmacokinetic Data

Pharmacokinetics in 5-6 compartments of single dose tenofovir oral (300 mg), single dose tenofovir 1% topical gel and seven day tenofovir 1% topical gel will be evaluated after rectal administration. This will include the plasma safety tenofovir PK profile of the initial (24 hour) oral and topical single dose (Visit 3 and Visit 7) and following the once-daily self administered dosing (Visit 12).

Single Dose:

The primary pharmacokinetic parameter to be calculated after a single oral and single rectal dose of tenofovir will be the area under the matrix concentration-time curve from 0 to infinity ($0 \rightarrow \infty$). This will be estimated using the log-linear trapezoidal method. Tenofovir area under the curve $(AUC)_{0\rightarrow\infty}$ will be estimated in each subject using 4 samples obtained over 12 days after a single oral or topical tenofovir dose in the following 4 matrices: blood plasma, cervicovaginal fluid, rectal fluid, and rectal tissue. Tenofovir-diphosphate $AUC_{0\rightarrow\infty}$ will be estimated in each subject using 4 samples obtained over 12 days after a single oral or topical tenofovir dose in the following 3 matrices: PBMCs, rectal tissue, and mononuclear cells isolated from rectal tissue.

To perform an extracellular and intracellular concentration comparison between blood plasma and vaginal fluid, rectal fluid, and rectal tissue, a composite approach will be used: a composite concentration-time profile over 12 days will be generated for all matrices. This time profile will be used to calculate a composite $AUC_{0\rightarrow\infty}$, in addition to a CL/F, and a $t_{1/2}$. To compare tissue pharmacokinetics to fluid biological matrices, an estimated tissue density of 1.05 g/mL will be used to convert ng/gm to ng/mL.

Multiple Doses

No AUC will be calculated for this 7-day dosing phase, as samples (plasma, PBMC, rectal fluid, vaginal fluid) from only two time points will be collected (pre-dose #1 and 30 minutes post-dose #7).

Statistical Analyses for PK

1. Dosing Route Comparisons

Intra-individual comparisons for intracellular (PBMCs, rectal tissue, isolated rectal mononuclear cells) and extracellular (plasma, rectal fluid, vaginal fluid, rectal tissue) tenofovir $AUC_{0\rightarrow\infty}$ will be performed to determine differences in exposure between single dosing of oral and rectal tenofovir.

Inter-individual comparisons will be performed on composite AUCs within each single oral and rectal dosing phase to determine the relative extracellular and intracellular penetration of tenofovir in systemic and peripheral compartments.

2. Dosing Frequency Comparisons

Intra-individual comparisons for intracellular (PBMCs, rectal tissue, isolated rectal mononuclear cells) and extracellular (plasma, rectal fluid, vaginal fluid, rectal tissue) tenofovir concentrations will be performed for the 30 minute post-dose timepoint (Visit 3 and Visit 7) and the 24 hour post-dose timepoint (Visit 12) sampled in the single and multiple dose rectal phases of the study.

Acceptability Data Analysis

The quantitative data will be primarily descriptive of demographic variables (ethnic and racial background, age, education, and income), sexual behavior in the prior three months, lubricant and enema use, frequency of HIV testing, and substance use in the prior three months, and will provide descriptive statistics of acceptability, specifically about the product's characteristics, application process, applicator, as well as the degree to which participants are bothered by leakage, soiling, or other problems related to gel use. Consistent with the secondary study objective to evaluate the acceptability of tenofovir 1% gel when applied rectally, the secondary endpoint is to examine the proportion of participants who at their Final Clinic Visit report that they would be very likely to use the candidate microbicide during receptive anal intercourse. We will calculate the proportion of participants who report high intentionality, operationalized as having a rating in the upper one third of the 10-point Likert scale, to use the product in the future every time they have receptive anal intercourse in the Product Acceptability Questionnaire. Furthermore, we will examine intentionality to use the study gel on occasions when they do not use condoms or if they had to wait 30 minutes after application before having receptive anal intercourse with various types of partners (e.g., lovers, one-night stands, or other partners). Because of insufficient statistical power to evaluate whether acceptability assessed at the Final Clinic Visit in the Product Acceptability Questionnaire is different by study condition, we will examine the distributions of each acceptability variable and inspect effect sizes by treatment condition.

The qualitative data will serve to contextualize participants' ratings of acceptability. The in-depth phone interviews will be audio-taped, transcribed, and analyzed for content. In the initial stage of the qualitative data processing, a research assistant will check the accuracy of all transcripts against the actual audio recordings to make sure there is no missing data and two separate investigators will identify categories, themes, and patterns, and develop a coding scheme. Codes generated by the two investigators will be compared and synthesized to end up with shared coding structure that will constitute the preliminary codebook. This codebook will be refined through further coding and discussion between the coders until consensus is reached. Once the transcripts and codebook are completed, they will be entered in NVivo, a software program designed for qualitative data analysis. The analysis of coded material will lead to the progressive

identification of categories, themes, and patterns. This will involve noting regularities in the setting or people under study and organizing them under a conceptual label. As the analysis progresses, hypotheses will be developed and challenged with the rest of the collected qualitative data. In this process, we will be able to integrate the quantitative responses to gain insights about the qualitative data. For example, we will have the opportunity to group participants based on their responses in the quantitative assessment (i.e., inconsistent condom users) and use these as attributes for each case in analyzing the qualitative data. Attributes may prove relevant when comparing and contrasting themes and concepts that emerge from the qualitative data. Ultimately, the qualitative and quantitative data will be integrated with the purpose of creating a full-fledged picture of the different factors affecting acceptability.

10.8 Statistical Procedures for Missing Data

All reasonable efforts will be made to obtain complete data for all participants; however, missing observations will occur due to missed visits, participants lost to follow-up, or noncompliance with the full acceptability assessment or secondary measures. In order to account for this, and to be able to perform intent to treat analysis on the outcomes, multiple imputation will be employed. [86, 87] The imputation will be implemented using the MI procedure in SAS or the IveWARE library.[88] In order to construct these models independently of the data from the current study, imputation model building will be based wherever possible on the data from HPTN 056 as well as RMP-01. Past experience has shown there is high compliance and very little missing data in these studies and we expect the same to be true here. Hence the rate of missing data is expected to be very low. Should this not be the case, we will conduct sensitivity analyses to ascertain how much our results depend on the assumptions made for the multiple imputation.

11 DATA HANDLING AND RECORDKEEPING

11.1 Data Management Responsibilities

Study case report forms will be developed by the UCLA-based RMP Regulatory Core (Core B) and Data Management Core (Core C). Quality control reports and queries routinely will be generated and distributed by the RMP Regulatory Core to the study sites for verification and resolution prior to reporting to the DAIDS MO.

11.2 Source Documents and Access to Source Data/Documents

Source documents and access to source data/documents will be maintained in accordance with the Requirements for Source Documentation in DAIDS Funded and/or Sponsored Clinical Trials. The investigators will maintain, and store securely, complete, accurate and current study records throughout the study. In accordance with U.S. regulations, the investigators will retain all study records on site for at least two years after study closure. Study records will not be destroyed prior to receiving approval for

record destruction from DAIDS. Applicable records include source documents, site registration documents and reports, correspondence, informed consent forms, and notations of all contacts with the participant.

11.3 Quality Control and Quality Assurance

Quality control and quality assurance procedures for RMP-02/MTN-006 will be performed in accordance with Requirements for Clinical Quality Management Plans at DAIDS Funded and/or Supported Clinical Research Sites. As this is a non-network trial, PPD (Wilmington, NC) will monitor the trial while the Regulatory Core at UCLA will internally oversee both clinical research sites and ensure that the sites are appropriately prepared for formal monitoring by PPD.

11.4 Study Coordination

CONRAD holds the IND for this study. Assignment of all sponsor responsibilities for this study will be specified in a Clinical Trials Agreement executed by DAIDS, CONRAD, and Gilead Sciences, Inc. Study site staff will be provided with the DAIDS SOPs for Source Documentation and Essential Documents, the Manual for Expedited Reporting of Adverse Events to DAIDS, and the DAIDS AE Grading Table. Training and written instructions outlining management and reporting, study gel dispensing, product accountability, and other study operations will be provided by the RMP Regulatory Core.

12 CLINICAL SITE MONITORING

Non-network study monitoring will be carried out by PPD (Wilmington, NC). On-site study monitoring will be performed in accordance with Requirements for On-Site Monitoring of DAIDS Funded and/or Sponsored Clinical Trials. Site monitoring visits will be conducted to assess compliance with Health and Human Services (HHS) Regulations 45 Code of Federal Regulations (CFR) Part 46 and 21 CFR Parts 50, 56, and 312. Study monitors will visit the site to:

- Verify compliance with human subjects and other research regulations and guidelines, including confidentiality procedures, informed consent process, and regulatory documentation

- Assess adherence to the study protocol, study-specific procedures manual, and local counseling practices

- Confirm the quality and accuracy of information collected at the study site and entered into the study database, including the validation of data reported on case report forms

- Assess the resolution of any past or ongoing issues identified at previous monitoring visits

Site investigators will allow study monitors to inspect study facilities and documentation (e.g., informed consent forms, clinic and laboratory records, other source documents, case report forms), as well as observe the performance of study procedures. Investigators also will allow inspection of all study-related documentation by authorized representatives of the MTN NL, NIAID, CONRAD, Gilead Sciences, Inc. and US regulatory authorities. A site visit log will be maintained at the study site to document all visits.

13 HUMAN SUBJECTS PROTECTIONS

The investigators will make efforts to minimize risks to human participants. Volunteers and study staff members will take part in a thorough informed consent process. Before beginning the study, the investigators will have obtained IRB approval and the protocol will have been submitted to the FDA. The investigators will permit audits by the NIH, MTN, CONRAD, Gilead Sciences, Inc., the FDA, OHRP, or any of their appointed agents.

13.1 Institutional Review Boards

Each participating institution is responsible for assuring that this protocol and the associated informed consent documents and study-related documents are reviewed by an Institutional Review Board (IRB) prior to implementation of the protocol. Any amendments to the protocol, informed consents, or other study- related documents must be approved by the IRB and study sponsors prior to implementation.

13.2 Protocol Registration

Each study site will complete protocol registration with the DAIDS RCC Protocol Registration Office. Protocol registration material can be sent electronically to epr@tech-res.com. For questions regarding protocol registration, please call (301) 897-1707. For additional information, refer to the protocol registration documents located at http://rcc.tech-res.com/forms.htm.

Protocol registration must occur as a condition for site-specific study activation; no participants may be screened or enrolled in this study prior to obtaining protocol registration approval and completing all other study activation requirements. UCLA RMP staff will notify each study site when all activation requirements have been met by issuing a site-specific study activation notice. Study implementation may not be initiated at a particular site until the activation notice for that site is issued.

The study will be conducted in full compliance with the protocol. The protocol will not be amended without prior written approval by the Protocol Chair and DAIDS Medical Officer.

13.3 Risk Benefit Statement

13.3.1 Risks

<u>General</u>
Phlebotomy may lead to discomfort, feelings of dizziness or faintness, and/or bruising, swelling and/or infection.

<u>Social Harms</u>
Disclosure of STI status may cause sadness or depression in volunteers. Participation in clinical research includes the risks of loss of confidentiality and discomfort with the personal nature of questions.

Although study sites make every effort to protect participant privacy and confidentiality, it is possible that participants' involvement in the study could become known to others, and that social harms may result (i.e., because participants could become known as HIV-infected or at "high risk" for HIV infection). For example, participants could be treated unfairly or discriminated against, or could have problems being accepted by their families and/or communities.

Participants in sites requiring partner notification in response to diagnosed STD or HIV infection could have problems in their relationships with their sexual partners. Participants also could have problems in their partner relationships associated with use or attempted use of study products. In addition, participants could misunderstand the current experimental status of the study gels (i.e., their unknown safety and unproven efficacy) and as a result increase their HIV risk behaviors while in the study.

<u>Risk of Answering the Web-Based Questionnaire</u>
There may be discomfort or embarrassment related to questions dealing with sexual behaviors and personal habits.

<u>Risk of Participating in In-Depth Interview</u>
Participants may feel embarrassed or uncomfortable when answering questions about sexual practices and attitudes. While not anticipated, there is also the potential risk of a violation of the participants' privacy and confidentiality, in the event that someone overhears the telephone conversation.

<u>Risks from flexible sigmoidoscopy with biopsies</u>
Flexible sigmoidoscopy is a commonly practiced medical procedure and the endoscopic procedures done in this trial will not involve any unusual risks or discomforts. The risks associated with these procedures include mild discomfort and the feeling of having a "bloated stomach". Endoscopic biopsies are painless and heal quickly within 3 days or less. On extremely rare occasions, the endoscopic procedure or biopsies may lead to pain, infection (sepsis), bleeding or perforation of the gastrointestinal tract. Perforation occurs approximately once out of every 0.88/1,000 procedures. If this extremely rare complication occurs, antibiotics and surgery to repair the tear may be necessary. The

impact of the rectal application of tenofovir 1% gel on the intestinal mucosa is unknown and may increase the risk of these complications.

Risks of sedation

A flexible sigmoidoscopy is usually preformed without sedation, although the subject may request to have intravenous sedative medication given to control for discomfort. There is a minimal risk of bruising and/or thrombophlebitis at the IV site. Depending upon the sedation used and the amount given the subject may experience temporary drowsiness and/or lightheadedness. If the subject receives sedation, s/he will need to arrange for a ride home. The subject may also feel nauseated and lose his/her appetite for the remainder of the day. Rare, but severe and life-threatening adverse reactions to conscious sedation are aspiration, cardiac arrest, and/or pulmonary arrest.

Risks from enemas

The main risk from having an enema is temporary discomfort. A hollow tube about the thickness of a pencil will be used to put approximately 125mL of Normosol-R pH7.4 into the rectum to stimulate a bowel movement with stool evacuation (a larger volume may be required if the initial volume does not produce results). This may cause a “bloated” or “crampy” feeling. Some air may be pumped into the rectum as well, causing flatulence. The tube is small, but it might cause some anal or rectal discomfort if the subject has any hemorrhoids or other painful conditions. The impact of the rectal application of tenofovir 1% gel on the intestinal mucosa is unknown and may increase the risk of discomfort.

Risks from rectal sponge and swab collection

There is no risk from the rectal sponge and swabs themselves. Subjects may have minor discomfort from the insertion of the anoscope used during the insertion of the rectal sponges/swabs and the lining of the rectum may become irritated. This irritation may last for the remainder of the day. The impact of the rectal application of tenofovir 1% gel on the intestinal mucosa is unknown and may increase the risk of discomfort.

Risk from the applicator

The applicator is small and is made of plastic. It is possible that the subject may feel some discomfort from the applicator. The applicator has been designed for vaginal, not rectal use. To minimize the risk of trauma during applicator insertion, subjects will be given a commercially available lubricant to use in conjunction with the applicator and written instructions, as well as an in-person tutorial, on proper use.

Risks of self-administered sponges for collection of cervicovaginal fluid

The subject could experience discomfort and/or pressure in the vagina and/or pelvis.

Risks of oral TDF tablets

The most common side effects associated with oral TDF in patients with HIV infection are nausea, headache, diarrhea, vomiting, asthenia, flatulence, abdominal distension/pain and anorexia. Less common side effects of TDF include kidney toxicities and low blood phosphate. Other side effects reported in the post-marketing period include weakness, pancreatitis, low blood phosphate, dizziness, shortness of

breath, and rash. In animal studies, tenofovir has been associated with decreased bone mineral density. These effects have not been seen in those taking tenofovir tablets for up to one year. All the above side effects are presumed to be very low in individuals taking a single oral dose, as in this trial.

Risks of tenofovir 1% gel

There is currently no rectal safety data regarding the use of tenofovir 1% gel. Administration of tenofovir gel intravaginally at 0.3% and 1% concentrations in the HPTN 050 Phase1 study resulted in minimal local irritation and little or no systemic adverse effects were identified. Although 92% of participants reported at least 1 AE, 87% of those reported AEs were mild, and 70% of the AEs were limited to the genitourinary tract. Four severe AEs were reported, with only one, lower abdominal pain, thought to be product-related. The risks associated with tenofovir gel are believed to be less than those identified for systemic use. In the HPTN 050 Phase1 study of tenofovir gel, serum PK analysis in a subset of participants demonstrated that there is no clinically significant systemic toxicity. Fourteen of 25 women with PK results had low, but detectable, serum tenofovir levels. In a male tolerance study (CONRAD A04-099/IND 73,382), tenofovir 1% gel was well tolerated in men following seven days of once daily exposure, for 6 to 10 hours, to the penis. There were few reported and observed genital findings after product use including mild pain (burning, irritation, discomfort) and pruritis[46]. All observed findings were classified as mild, small in size and requiring no treatment. Reported symptoms were mild, of short duration and resolved by the final visit. There were no noticeable differences between signs and symptoms of genital irritation in the circumcised compared to uncircumcised group[46, 70].

Given that Phase 1 data demonstrate measurable plasma concentrations of tenofovir in some participants, participants with hepatitis B infection might be at risk for development of tenofovir resistant hepatitis B. However, participants with known hepatitis B infection will not be eligible for enrollment. There is a theoretical risk that tenofovir absorbed systemically from tenofovir gel could result in mutations of the HIV virus in participants who become infected with HIV during the study, or their partner, if the partner is infected with HIV. Limited resistance data from HPTN 050 show no new resistance mutations in plasma or cervicovaginal lavage (CVL) specimens after 14 days of tenofovir gel use. No participant had high level tenofovir mutations (e.g. K65R). Some of the possible side effects of the study gel are dryness, itching, burning, or pain in the genital area.

Participants will also be counseled on the importance of remaining sexually abstinent during the active phases of the study as well as for 5 days after the last biopsy collection to minimize risk to the rectum since the effects of the study gels on the rectum are unknown.

HEC gel

There is currently no rectal safety data regarding the use of HEC gel. Twice daily intravaginal administration of HEC gel over the course of two weeks resulted in mild genital irritation, including genital burning, soreness, and pelvic pain, in 2 out of 14

women (14.3%).[34] [73] Three out of the 14 women (12.4%) had colposcopic findings which included erythema, petechiae and peeling, although no findings with deep disruption were observed during follow-up. HEC gel did not appear to alter vaginal health or shift vaginal flora and no SAEs were reported.

13.3.2 Benefits

Participants in this study may experience no direct benefit. Participants and others may benefit in the future from information learned from this study. Specifically, information learned in this study may lead to the development of safe and effective interventions to prevent HIV transmission. Participants also may appreciate the benefit of earlier diagnosis of STDs in addition to the opportunity to contribute to the field of HIV prevention research and will either receive treatment or be referred to proper medical facilities to receive appropriate treatment for STDs other than HIV. Participants will also be referred for care for any incidental conditions identified during screening and other examinations.

13.4 Informed Consent Process

Written informed consent will be obtained from all potential study participants prior to the initiation of any study-related procedures. In obtaining and documenting informed consent, the investigators and their designees will comply with applicable local and country-specific regulatory requirements and will adhere to Good Clinical Practices (GCP) and to the ethical principles that have their origin in the Declaration of Helsinki. Study staff must document the informed consent process in accordance with the Requirements for Source Documentation in DAIDS Funded and/or Sponsored Clinical Trials. A comprehension checklist will be used to assess participants' comprehension of the enrollment informed consent document. Participants are provided with copies of the informed consent forms if they are willing to receive them. Each study site is responsible for developing study informed consent forms for local use, based on the templates in Appendices VI, VII, and VIII that describe the purpose of screening and of the study, the procedures to be followed, and the risks and benefits of participation, in accordance with all applicable regulations. Prior to the beginning of the trial, site investigators will have IRB written approval of the protocol, informed consent forms, and any other study-related information to be provided to participants.

The informed consent process will give individuals all of the relevant information they need to decide whether to participate, or to continue participation, in this study. Potential research participants will be permitted to ask questions and to exchange information freely with the study investigators. Listed study investigators or their designees will obtain informed consent from potential study participants. The investigators will keep research participants fully informed of any new information that could affect their willingness to continue study participation.

The informed consent process covers all elements of informed consent required by research regulations. In addition, the process specifically addresses the following topics of import to this study:

- The importance of adherence to the study visit and procedures schedule.
- The potential risks of study participation (and what do if such risks are experienced).
- The potential social harms associated with study participation (and what do if such harms are experienced).
- The real yet limited benefits of study participation.
- The distinction between research and clinical care.
- The right to withdraw from the study at any time.

13.5 Participant Confidentiality

All study procedures will be conducted in private, and every effort will be made to protect participant privacy and confidentiality to the extent possible. Each study site will establish a standard operating procedure for confidentiality protection that reflects the local study implementation plan (e.g., whether community-based visits will be conducted) and the input of study staff and community representatives to identify potential confidentiality issues and strategies to address them. In addition to local considerations, the protections described below will be implemented at all sites.

All study-related information will be stored securely at the study site. All participant information will be stored in locked file cabinets in areas with access limited to study staff. Data collection, process, and administrative forms, laboratory specimens, and other reports will be identified by a coded number only to maintain participant confidentiality. All records that contain names or other personal identifiers, such as locator forms and informed consent forms, will be stored separately from study records identified by code number. All local databases will be secured with password-protected access systems. Forms, lists, logbooks, appointment books, and any other listings that link participant ID numbers to other identifying information will be stored in a separate, locked file in an area with limited access. Participants' study information will not be released without their written permission, except as necessary for monitoring (see Section 12).

In addition, a Certificate of Confidentiality from the US Department of Health and Human Services will be obtained for this study. This Certificate protects study staff from being compelled to disclose study-related information by any US Federal, State or local civil, criminal, administrative, legislative or other proceedings. It thus serves to protect the identity and privacy of study participants.

13.6 Special Populations

This section outlines considerations made for the inclusion or exclusion of special populations in this study.

13.6.1 Pregnant Women

Participants who test positive for pregnancy at screening or enrollment visits will not be eligible to participate in this study. Additionally, urine pregnancy tests will be performed on all women at Visits 1, 2, 3, 7, 11, and at interim visits as indicated. Participants who test positive will be taken off product. During the informed consent process, women will be informed that oral tenofovir disoproxil fumarate and tenofovir gel are not methods of contraception and that the effects of oral tenofovir disoproxil fumarate and tenofovir gel on a developing human fetus are unknown.

Oral tenofovir disoproxil fumarate is classified by the FDA as a pregnancy category B drug. Animal studies have failed to demonstrate a risk to the fetus, but there are no adequate and well-controlled studies in pregnant women that have been completed to date.

All potential participants will be required by the Eligibility Criteria for Screening and Enrollment to be currently using a reliable method of contraception, such as hormonal contraception, intrauterine device, or sterilization. Women who become pregnant during the study period following randomization and exposure to study product will discontinue product use; however, their PK assessments but will not be excluded from analysis.

13.6.2 Children

The NIH has mandated that children be included in research trials when appropriate. This study meets “Justifications for Exclusion” criteria for younger children as set forth by the NIH. Specifically, “insufficient data are available in adults to judge potential risk in children” and “children should not be the initial group to be involved in research studies.” Oral TDF is not currently approved for children under 18 years old. This study does not plan to enroll children under 18 years old.

13.7 Incentives

Pending IRB approval, participants will be compensated for their time and effort in this study, and/or be reimbursed for travel to study visits, child care, and time away from work.

13.8 Communicable Disease Reporting

Study staff will comply with all applicable local requirements to report communicable diseases including HIV identified among study participants to local health authorities. Participants will be made aware of all reporting requirements during the study informed consent process.

13.9 Access to HIV-related Care

13.9.1 HIV Counseling and Testing

HIV pretest and post-test counseling will be provided to all potential study participants who consent to undergo HIV screening to determine their eligibility for this study. Participants must receive their HIV test results to take part in this study. Participants who have positive or indeterminate results will have standard post-test counseling as well as limited follow-up confirmatory testing provided by the study. Referral for additional counseling related to testing or diagnosis will occur if needed or requested by the participant.

13.9.2 Care for Participants Identified as HIV-Infected

Study staff will provide participants with their HIV test results in the context of post-test counseling. According to site SOPs, study staff will refer participants found to be HIV-infected to available sources of medical and psychological care, social support, and local research studies for HIV-infected women.

13.10 Study Discontinuation

This study may be discontinued at any time by NIAID, the MTN, CONRAD, Gilead Sciences, Inc., the US FDA, the OHRP, other government or regulatory authorities, or site IRBs

14 PUBLICATION POLICY

The IPCP U19's MDP and RMP policies, DAIDS, MTN, and a Clinical Trial Agreement (CTA) between CONRAD, Gilead Sciences, Inc. and NIAID will govern publication of the results of this study. Any presentation, abstract, or manuscript will be submitted by the Investigators to the MDP/RMP Executive Committee, the MTN Manuscript Review Committee, DAIDS, CONRAD, and Gilead Sciences, Inc., for review prior to submission.

15 APPENDICES

APPENDIX I: SCHEDULE OF STUDY VISITS AND EVALUATIONS

	Visit 1	Visit 2	Visit 3	Visit 4	Visit 5	Visit 6	Visit 7	Visit 8	Visit 9	Visit 10	Visit 11	Visit 12	Visit 13/ Early Term	F/U Call	Interim
	SCR	ENR	Single dose oral phase				Single dose rectal phase				7-day rectal phase				
Informed consent	X	X													
PTID	X														
Demographics	X	X													
Locator information	X	X	X	X	X	X	X	X	X	X	X	X			
Test results		X	X				X				X	X			
Eligibility assessment	X														
Baseline behavioral questionnaire		X													
Confirm eligibility		X													
Adherence (protocol and/or product)		X	X	X	X	X	X	X	X	X	X	X			
Reimbursement	*	X	X	X	X	X	X	X	X	X	X	X	X		
Schedule next study visit	▲	X	X	X	X	X	X	X	X	X	X	X	X	▲	▲
Randomization		X													
Med/menstrual history	X	X	X	X	X	X	X	X	X	X	X	X	X		X
Concomitant meds	X	X	X	X	X	X	X	X	X	X	X	X	X		X
Smoking assessment	X														
Physical exam	X	X	X	X	X	X	X	X	X	X	X	X			▲
Rectal exam	X														▲
Record/update AEs		X	X	X	X	X	X	X	X	X	X	X	X	X	▲
HIV pre-/post-test couns	X	▲	▲	▲	▲	▲	▲	▲	▲	▲	▲	X			▲
HIV/STI risk reduction	X	X	X	X	X	X	X	X	X	X	X	X	X		
Contraceptive couns	X	X	X	X	X	X	X	X	X	X	X	X	X		
Qualitative hCG	♀	♀	♀				♀				♀	♀			▲
Dipstick UA	X														
Urine NAAT for GC/CT	X														
CBC w/ diff and platelets	X		X				X					X			
AST	X	X	X				X					X			
ALT	X	X	X				X					X			
Creatinine	X	X	X				X					X			
Phosphate	X	X	X				X					X			
Plasma archive		X													
Plasma tenofovir levels			X	X	X	X	X	X	X	X	X	X	X		
PBMC tenofovir levels			X	X	X	X	X	X	X	X	X	X	X		
Syphilis RPR	X											X			▲
Confirm syphilis	▲											▲			▲
HIV-1 serology	X	▲	▲	▲	▲	▲	▲	▲	▲	▲	▲	X			▲
Confirm. HIV-1	▲	▲	▲	▲	▲	▲	▲	▲	▲	▲	▲	▲			▲
HSV-1 and -2 serology	X														
HBsAg	X														
Vaginal pH	X	X	X		X	X	X		X	X		X			
Vaginal swab (gram stain, BV assessment)	X	X	X		X	X	X		X	X		X			
Vaginal sponge (dry)		X	X	X	X	X	X	X	X	X	X	X	X		
Rectal GC/CT by NAAT	X														
Normosol-R enema		X	X		X	X	X		X	X		X			
Calprotectin		X	X		X	X	X		X	X		X			
Epithelial sloughing		X	X		X	X	X		X	X		X			
Rectal secreted cytokines		X	X		X	X	X		X	X		X	X		
Rectal microflora			X				X					X	X		
Flexible Sigmoidoscopy		X	X		X	X	X		X	X		X			
Histology		X	X		X		X		X			X			
CD4 cells (MMC phenotype)		X	X		X		X		X			X			
CD4 cells (TFV levels in MMC)			X		X		X		X			X			
HIV infectability		X	X		X	X	X		X	X		X			
TFV levels in rectal mucosal tissue homogenates			X		X	X	X		X	X		X			
Rectal tenofovir concentrations		X	X	X	X	X	X	X	X	X	X	X	X		
Study product			X				X				X	X			
Collect product use log, used/unused product												X	X		
Male condoms	X	X	X	X	X	X	X	X	X	X	X	X	X		
Product admin phone call											X				
Accept. Questionnaire and phone interview													X		
Safety phone call														X	

▲ if indicated * per site standard

APPENDIX II: HIV ANTIBODY TESTING ALGORITHMS

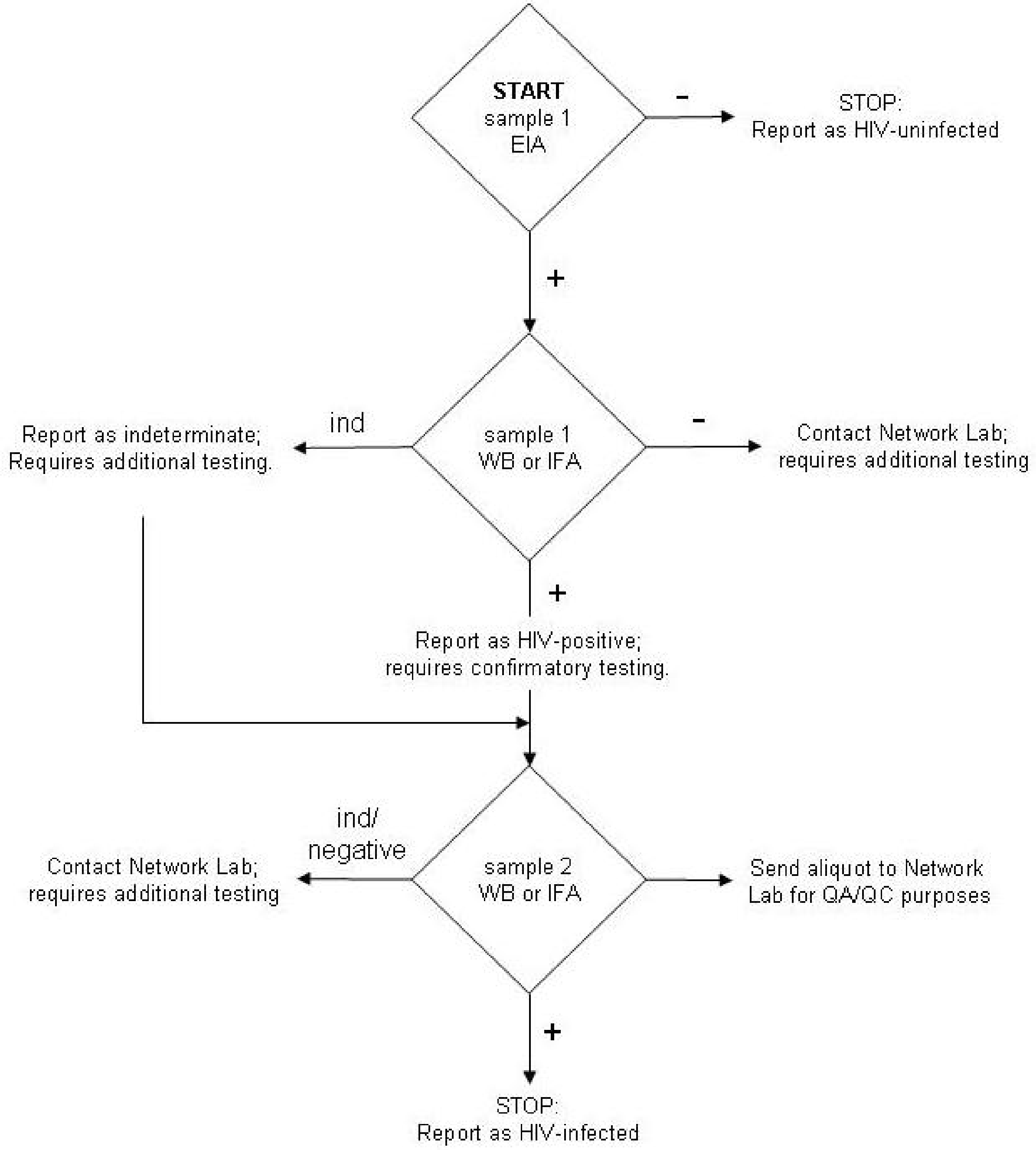

APPENDIX III: TOXICITY TABLES

The DAIDS AE Grading Table Version 1.0, December 2004, Addenda 1 and 3 (Female Genital and Rectal Grading Tables for Use in Microbicide Studies) will be the primary tool for grading adverse events for this protocol. Adverse events not included in that table will be graded by the DAIDS AE Grading Table Version 1.0, December 2004. In cases where an AE is covered in all tables, Addendum 3 (Rectal Grading Table for Use in Microbicide Studies) will be the grading scale utilized.

The Division of AIDS Table for Grading the Severity of Adult and Pediatric Adverse Events (DAIDS AE Grading Table), Version 1.0, December, 2004, is available on the RCC website at http://rcc.tech-res.com/eae.htm.

APPENDIX IV: MANUAL FOR EXPEDITED REPORTING OF ADVERSE EVENTS TO DAIDS

The Manual for Expedited Reporting of Adverse Events to DAIDS, Final 1.0, 6 May 2004 is available at: http://rcc.tech-res.com/eae.htm

APPENDIX V: RECTAL MICROFLORA

FACULTATIVE ISOLATES

Lactobacillus, H_2O_2-producing
Lactobacillus, non-H_2O_2-producing
Gardnerella vaginalis
Diphtheroids
Bacillus
Gram positive rods, other
Group B *Streptococcus*
Enterococcus
Staphylococcus aureus
Staphylococcus, coagulase-negative
Micrococcus
Viridans *Streptococcus*, H_2O_2-producing
Viridans *Streptococcus*, non-H_2O_2 producing
Gram positive cocci, other
Escherichia coli
Proteus
Gram negative rods, other

ANAEROBIC ISOLATES

Gram negative rods, *Bacteroides fragilis* group
Gram negative rods, other non-pigmented
Gram negative rods, pigmented
Gram positive cocci
Gram positive rods, *Clostridium*-like
Gram positive rods, other

APPENDIX VI: HISTOPATHOLOGY SCORING SYSTEM

Participant ID: _________ **Visit No.: _______** **Visit Date: ______**

Biopsy Site: _______

Please Circle the Grade	Please Circle Subgrade	
**Grade 0**<br>No abnormality		
**Grade 1**<br>Mononuclear cell infiltrate	Low	High
**Grade 2**<br>Neutrophilic infiltrate-lamina propria	Low	High
**Grade 3**<br>Neutrophilic infiltrate-epithelium	Low<br>*< 50% of crypts*	High<br>*> 50% of crypts*
**Grade 4**<br>Crypt destruction	Low<br>*probable*	High<br>*unequivocal*
**Grade 5**<br>Erosion or ulceration	Low<br>*Restitution, probable erosion*	High<br>*Unequivocal erosion or ulceration*

APPENDIX VII: SAMPLE INFORMED CONSENT FORM (SCREENING)

A two-site, Phase 1, partially-blinded, placebo-controlled safety, acceptability, and pharmacokinetic trial of topical, vaginally-formulated tenofovir 1% gel applied rectally compared with oral 300 mg tenofovir disoproxil fumarate in HIV-1 seronegative adults

Version 1.0

7 April 2009

**PRINCIPAL INVESTIGATOR:**	**[INSERT NAME]**
**PHONE:**	**[INSERT NUMBER]**
**Short Title for the Study:**	**Phase 1 rectal microbicide safety and acceptability trial of topically applied tenofovir compared with oral tablet**

INTRODUCTION

You are being asked to take part in these screening exams and tests because you are at least 18 years of age and have had at least one experience of receptive anal sex in the past twelve months, and you may be able to join the research study named above. This Rectal Microbicide Program (RMP) and Microbicide Trials Network (MTN) study is sponsored by the U.S. National Institutes of Health (NIH). The person in charge of this study at this site is [INSERT NAME OF PRINCIPAL INVESTIGATOR]. The screening exams and tests include interview questions, urine and blood tests, a physical exam, and an examination of your rectum.

YOUR PARTICIPATION IS VOLUNTARY

This consent form gives information about the screening tests that will be discussed with you. Once you understand the screening tests, and if you agree to take part, you will be asked to sign your name on this form. You will be offered a copy of this form to keep.

Before you learn about the screening tests, it is important that you know the following:

- You do not have to be in this study if you do not want to.
- You may decide not to have the screening tests, or to withdraw from the screening tests at anytime.
- You are only being asked to have the screening tests at this time. Even if you agree to have the screening tests, you do not have to join the research study.
- Some people may not be able to join the research study because of information found during the screening tests.
- You will receive the results of the screening tests even if you are not eligible to join the research study.

WHY ARE THE SCREENING EXAMS AND TESTS BEING DONE?
These exams and tests are being done to see if you can be in this study.

WHAT IS THE PURPOSE OF THE STUDY?
There are two main purposes of this study. The first is to find out whether the study gel causes any side effects when inserted into the rectum or if the study tablet causes any side effects when taken by mouth and to find out if using the study gel can cause changes to the body's cells that might make it easier for people to get HIV. The second purpose is to find out how men and women feel about inserting the study gel into their rectum. Some other studies are being done to see if the study gel, when inserted into the vagina, can be used to prevent the spread of Human Immunodeficiency Virus, or HIV. HIV is the virus that causes Acquired Immunodeficiency Virus, or AIDS. This study is being done so we can make sure the study gel is safe to use before we begin testing it in more people.

The main study product is called tenofovir gel. The side effects from tenofovir will be compared to the side effects from the placebo gel. The tenofovir gel and placebo are not approved for rectal use and the tenofovir gel has not yet been applied rectally in humans. The placebo gel does not have any medicine in it. All participants will also receive one dose of the tablets, called tenofovir tablet.

WHAT DO I HAVE TO DO IF I TAKE PART IN THE SCREENING EXAMS AND TESTS?
The Screening Visit will take about 2 hours. You will be asked to do these things if you decide you want to be in the study:

- Sign this form after you have read it or had it explained to you and had the chance to ask questions about the study
- Answer questions about yourself, such as where you live, your education, your behavior, including your sexual behavior, your medical history, menstrual period history, smoking habits, and any medicines that you may take and how we can contact you
- Have a physical exam
- Have an exam of your rectum
- Hear about
    - different ways to avoid getting pregnant
    - how to avoid infections passed during sex
    - how to use male condoms
- Get treatment for any infections passed during sex or urinary tract infection that you may have, or find out from the study staff where you can get care or treatment
- Provide a urine sample to get tested for pregnancy and urinary tract infections
- Provide samples of fluid from your vagina to check the health of your vagina
- Have samples of fluid from your rectum taken to get tested for gonorrhea, and chlamydia
- Have a blood sample [sites to insert amount] taken to check these things:

- The health of your blood, liver, and kidneys
  - HIV test
  - Syphilis test
  - Herpes
- Receive male condoms from the study staff
- It will take about [INSERT LENGTH OF TIME] to get the results of your screening tests. We will give you the results of these tests when they are available.
- If the results of your screening tests and answers to the screening questions show that you are able to take part in this study, the study staff will schedule an enrollment visit.

WHY WOULD THE DOCTOR STOP THE SCREENING PROCEDURES EARLY?

The study doctor may need to stop the screening exams and tests early without your permission if:

- The study is cancelled by the U.S. Food and Drug Administration (FDA), U.S. National Institutes of Health (NIH), the RMP the MTN, the drug companies supporting this study, the Ethics Committees, the Office for Human Research Protections, the local government or regulatory agency, or the Institutional Review Board (IRB). (An IRB is a committee that watches over the safety and rights of research participants).
- Your exams, tests and answers to the questions show you cannot join the study.
- The study staff feels that having the screening exams and tests would be harmful to you.
- You do not want to find out your HIV test result.
- You are not able to come to the visits or complete the screening exams and tests.
- Other reasons that may prevent you from completing the study.

WHAT ARE THE RISKS OF THE SCREENING VISIT TESTS?

Risk of Blood Draws:

- You may feel discomfort or pain when your blood is drawn.
- You may feel dizzy, faint or lightheaded.
- You may have a bruise, swelling, or infection where the needle goes into your arm.

Risk of Rectal Exams and Anorectal Swabs:

- You may feel discomfort or pressure when your rectum is examined.
- You may experience some discomfort when the swab is inserted into the rectum, and occasionally minor rectal bleeding may occur.

Risks of Vaginal Swabs

- You may experience some discomfort when the swab is inserted into the vagina, and occasionally minor vaginal bleeding will occur.

Other Possible Risks:

- You may become embarrassed, worried, or nervous when discussing personal questions about your sexual behavior, ways to protect against HIV and other infections passed during sex, and your test results.
- You may become worried or nervous while waiting for your test results.
- If you have HIV or other infections, knowing this could make you worried or nervous. A trained counselor will help you deal with any feelings or questions you have.

We will make every effort to protect your privacy while you are having the screening exams and tests. Your visits here will take place in private. However, it is possible that others may learn that you are taking part in the study here. Because of this, they may treat you unfairly or discriminate against you. For example, you could have problems getting or keeping a job, or being accepted by your family or community. Finding out your HIV status could also cause problems between you and your partner.

ARE THERE BENEFITS TO TAKING PART IN THIS STUDY?

You may get no direct benefit from the screening exams and tests. However, you may benefit from the following:

- Physical exam and a rectal exam
- Tests for sexually transmitted infections, other rectal infections, and HIV (which may detect infections that have no symptoms). If you have any of these infections, you will receive treatment or be referred for treatment if needed.
- Tests to check your general health and the health of your liver, kidneys, and blood. This study cannot provide you with medical care, but study staff will refer you to other available sources of care.
- Safer sex counseling and free male condoms
- If your tests show that you are infected with HIV, you will be referred for medical care, counseling, and other services available to you. Medical care for HIV infection will not be part of this study. You will need to get medical care for your HIV infection from your own health care provider or we will provide you with a referral to a center where you can receive care. We will help you to access the right treatment for HIV infection if you need it.

WHAT OTHER CHOICES DO I HAVE BESIDES THIS STUDY?

You do not have to participate in this study, if you choose not to do so. [SITES TO INCLUDE/AMEND THE FOLLOWING IF APPLICABLE: There may be other studies going on here or in the community for which you may be eligible. If you wish, we will inform you about other studies that are being conducted locally. There also may be other places where you can go for HIV counseling and testing. We will tell you about those places if you wish.] Please talk to your doctor about these and other choices that may be available to you.

WHAT ABOUT CONFIDENTIALITY?

This study is being conducted according to ethical guidelines and efforts will be made to keep your personal information private. Your physical and rectal exams will be done in

private. We cannot guarantee absolute confidentiality. In some situations, including emergencies, legal and professional rules may force us to share confidential information about you. If this study is published, your name will not be used and you will not be personally identified. You are encouraged but not required to tell sexual partners about your being in this study.

Your records may be reviewed by:

- The U.S. Food and Drug Administration (FDA)
- U.S. National Institutes of Health (NIH)
- Office for Human Research Protections (OHRP)
- [INSERT NAME OF SITE] IRB
- Study staff
- Study monitors
- The companies that make the gels used in this study
- The company that makes the tablet used in this study

[SITES TO INCLUDE/AMEND THE FOLLOWING IF APPLICABLE:]
[LOCAL/STATE/NATIONAL] regulations require study staff to report the names of people who test positive for HIV and other infections passed during sex to the [LOCAL HEALTH AUTHORITY]. Outreach workers from the [HEALTH AUTHORITY] may then contact you about informing your partners, since they also should be tested. If you do not want to inform your partners yourself, the outreach workers will contact them, according to the confidentiality guidelines of the [HEALTH AUTHORITY].

In addition to the efforts made by the study staff to keep your personal information confidential, a Certificate of Confidentiality has been requested from the U.S. Federal Government for this study. Once obtained, this Certificate will protect study staff from being forced to tell people who are not connected with this study, such as the court system, about your participation or information you give for study purposes. Even with the Certificate of Confidentiality, however, if the study staff learns of possible child abuse and/or neglect or a risk of harm to you or others, they will be required to tell the proper authorities. Having a Certificate of Confidentiality does not prevent you from releasing information about yourself and your participation in the study.

WHAT ARE THE COSTS TO ME?
There is no cost to you for the screening exams and tests.

WILL I RECEIVE ANY PAYMENT?
You will be paid for your time and effort for the screening visit. You will receive [INSERT SITE - SPECIFIC AMOUNT OF MONEY] for each visit. You will also be paid for other costs to you for coming to the screening visit [SUCH AS CHILD CARE, TRAVEL, AND LOSS OF WORK TIME – SITES TO COMPLETE].

WHAT HAPPENS IF I AM INJURED (EXPERIENCE HARM)?

If you are injured as a result of being in this study, the [INSTITUTION] will give you immediate treatment for your injuries. You [will/will not] have to pay for this treatment. You will be told where you can get additional treatment for your injuries. The U.S. National Institutes of Health (NIH) does not have a program to provide money or other forms of compensation for your injuries. Signing this consent form does not change your legal rights.

[SITES TO SPECIFY INSTITUTIONAL POLICY]

WHAT ARE MY RIGHTS AS A RESEARCH PARTICIPANT?

Taking part in the screening exams and tests is completely voluntary. You may choose not to have the screening exams and tests any time. You will be treated the same no matter what you decide.

We will tell you about new information from this or other studies that may affect your health, welfare or willingness to stay in this study. If you want the results of the study, please let the study staff know.

WHAT DO I DO IF I HAVE PROBLEMS OR QUESTIONS?

For questions about the screening exams and tests or if you have a research-related injury, you should contact:

- [SITE INSERT NAME OF THE INVESTIGATOR OR OTHER STUDY STAFF]
- [SITE INSERT TELEPHONE NUMBER AND PHYSICAL ADDRESS OF ABOVE]

For questions about your rights as a research participant, contact:

- [SITE INSERT NAME OR TITLE OF PERSON ON THE INSTITUTIONAL REVIEW BOARD (IRB) OR OTHER ORGANIZATION APPROPRIATE FOR THE SITE]
- [SITE INSERT TELEPHONE NUMBER AND PHYSICAL ADDRESS OF ABOVE]

SIGNATURES
Please carefully read the statements below and think about your choice. No matter what you decide it will not affect your care.

[Insert signature blocks as required by the local IRB:]

______________________	______________________	______________
Participant Name (print)	Participant Signature	Date
______________________	______________________	______________
Study Staff Conducting Consent Discussion (print)	Study Staff Signature	Date
______________________	______________________	______________
Witness Name (print)	Witness Signature	Date

APPENDIX VIII: SAMPLE INFORMED CONSENT DOCUMENT (ENROLLMENT)

A two-site, Phase 1, partially-blinded, placebo-controlled safety, acceptability, and pharmacokinetic trial of topical, vaginally-formulated tenofovir 1% gel applied rectally compared with oral 300 mg tenofovir disoproxil fumarate in HIV-1 seronegative adults

Version 1.0

7 April 2009

PRINCIPAL INVESTIGATOR: **[INSERT NAME]**
PHONE: **[INSERT NUMBER]**
Short Title for the Study: **Phase 1 rectal microbicide safety and acceptability trial of topically applied tenofovir compared with oral tablet**

INTRODUCTION

You are being asked to take part in these enrollment exams and tests because you are at least 18 years of age, have had at least one experience of receptive anal sex in the past twelve months, and have passed the screening for this research study. This Microbicide Trials Network (MTN) study is sponsored by the U.S. National Institutes of Health (NIH). The person in charge of this study at this site is [INSERT NAME OF PRINCIPAL INVESTIGATOR]. Before you decide if you want to join this study, we want you to know about this study.

YOUR PARTICIPATION IS VOLUNTARY

This is an enrollment consent form and gives you information about the study. The study staff will talk with you about this information. You are free to ask questions about this study at any time. If you agree to take part it this study, you will be asked to sign this consent form in front of a witness. You will be offered a copy of this form to keep.

Before you learn about this study, it is important that you know the following:

- You do not have to be in this study if you do not want to.
- You may decide not to have the enrollment tests, or to withdraw from the enrollment tests at anytime.
- Some people may not be able to join the research study because of information found during the enrollment process.
- You will receive the results of your tests even if you are not eligible to join the research study.

WHAT IS THE PURPOSE OF THE STUDY?
There are two main purposes of this study. The first is to find out whether the study gel causes any side effects when inserted into the rectum and to find out if using the study gel can cause changes to the body's cells that might make it easier for people to get HIV. The second purpose is to find out how men and women feel about inserting the study gel into their rectum. Some studies are being done to see if the study gel, when inserted into the vagina, can be used to prevent the spread of Human Immunodeficiency Virus, or HIV. HIV is the virus that causes Acquired Immunodeficiency Virus, or AIDS. This study is being done so we can make sure the study gel is safe to use before we begin testing it in more people.

The main study product is called tenofovir gel. The side effects from tenofovir will be compared to the side effects from the placebo gel. The tenofovir gel and placebo are not approved for rectal use and the tenofovir gel has not yet been applied rectally in humans. The placebo gel does not have any medicine in it. All participants will also receive one dose of the tablets, called tenofovir tablet.

STUDY GROUPS
There are two groups. If you take part in the study, you will be placed in one of the two groups. One group will receive the tenofovir gel rectally and the other group will receive the placebo gel. For every two participants who receive the tenofovir gel, one participant will receive the placebo gel. Your group will be chosen "by lot" [or other equivalent local term, for example, like flipping a coin or throwing dice] to be in one of these groups. You cannot choose your group, and the study staff cannot choose your group for you. You have an equal chance of being placed in either of the two groups. Once you are in a group, you cannot change to another group. Once you are chosen to be in one of the groups, you will be divided into either Timing A or Timing B. The people in Timing A will be asked to come in for their sampling visits on some days and the people in Timing B will be asked to come in on other days. The study procedures will be the same for everyone participating in the study. The study staff and study doctor will not know what group you are in. The only people who know what group you are in are the study statistician, the MTN pharmacist, and the companies who make the study products.

WHAT DO I HAVE TO DO IF I AM IN THIS STUDY?
If you decide to take part in the study, your first visit will continue today, after you read, discuss, understand, and sign this form. Study staff will help you understand the form and answer your questions before you sign this form.

Today, if you decide to sign this form, you will find out which study group you will be in for the study. You will also answer interview questions, including questions about your sexual practices, and have a rectal exam, to make sure you can still join the study. If you decide to join the study, you will also be asked to remain sexually abstinent during the times when you are taking the study products and for at least 5 days after your last biopsy collection.

Study visits will take about 45 minutes and last up to three hours.

At today's visit, you will also:

- Have samples of fluid from your rectum taken to test for gonorrhea and chlamydia
- Have a blood sample [insert amount] taken in case there is a question about your lab results from this visit. After all testing is done, this sample will be destroyed according to the site procedures for getting rid of blood samples that will not be needed after the end of the study.
- Complete a computerized questionnaire about you and your sexual practices, as well as your use of rectal douches, lubricants, alcohol and drugs. This should take about 25 minutes to complete.

You will be in the study for about 3.5 months from the time of your Enrollment Visit (today) up until your follow-up phone call at the end of the study.

At most visits, we will ask you to do the following:

- Let us know if there are any changes in where you live or how we may contact you
- Tell us about any changes in your medical or menstrual history
- Tell us if there have been changes to any medicines you are taking now
- Have a physical exam and will be asked about any changes to your health
- Hear about:
  - Ways to avoid becoming pregnant
  - How to avoid infections passed through sex including HIV
- Provide blood [insert amount] to:
  - Check for the amount of study drug in your blood
  - Check for HIV if the study doctor thinks you need to be checked
- Provide samples of fluid from your vagina to test for the amount of tenofovir in your vagina
- Have samples of fluid taken from your rectum to test for the amount of tenofovir in your rectum
- Learn how to follow the rules of the study and/or about study product rules
- Tell the study staff about any side-effects you think you might be having from the study products or the biopsies
- Receive male condoms from study staff
- Receive your test results
- Schedule your next visit

At some visits, we will ask you to do the following:

- Provide blood [insert amount] to:
  - Check the health of your blood, liver, and kidneys
  - Check for syphilis
- Provide a urine sample for a pregnancy test

In addition, at the Follow-up Visits (Visits 2, 3, 5, 6, 7, 9, 10, and 12), you will:

- Have an examination of your rectum (flexible sigmoidoscopy). This is when a flexible, long hollow tube is placed inside your rectum so that the study doctor can check the health of your rectum and take a sample of rectal tissue
    - Have small tissue samples taken from further inside your rectum (biopsy). The tissue samples will be tested to check the health of your rectum, and will be taken after you receive the study product.
- Have an enema. This is when a liquid is injected into your rectum to promote a bowel movement. The stool and liquid collected from your rectum afterwards will be tested to check the health of your rectum. This will be done before and after you receive the study product

At Visits 3, 7, 11, and 12, you will:

- Receive one tenofovir tablet at Visit 3 and receive either one dose of tenofovir gel OR one dose of placebo gel at Visit 7. The placebo gel does not contain any medicine
- Receive 7 applicators for 6-day supply of either tenofovir gel or placebo gel, be reminded to keep a record of when you use the study gel in the study diary, and schedule a daily phone call from study staff for the 6 days you will be using the study gel at home (Visit 11). The 7th dose of the study gel will be applied by the study doctor at the clinic at Visit 12

At Visit 12 or Visit 13 (if you leave the study early), you will:

- Be asked to return any used or unused study gel to the clinic
- Be asked to bring in your study diary

At Visit 13 , you will:

- Complete a computerized questionnaire about your use of the study gel. This should take about 20 minutes to complete.
- Take part in an interview with one of our researchers over the phone to discuss what your experiences were like in using the gel. The interview should take about an hour.

Follow-up Phone Call

Study staff will call you within 7 days after your last clinic visit. During this phone call, we will ask you to:

- Tell us about any side-effects you might have had from using the study gel

ANY TIME DURING THE STUDY

Please tell the study staff about any medical problems you have during the study. You can contact the study staff between regular visits to report these problems. The study staff will check you as needed and will refer you for medical care. At each study visit, the study staff will update your medical history and information on where you live and how to keep in touch with you.

HOW MANY MEN AND WOMEN WILL BE IN THIS STUDY?

A total of approximately 18 HIV-negative men and women from two study sites will be in this study.

RISKS AND/OR DISCOMFORTS

Risks from Phlebotomy (blood tests)

- You may feel discomfort or pain when your blood is drawn.
- You may feel dizzy or faint.
- You may have a bruise, swelling, small clot, or infection where the needle goes in your arm or finger

Risk of Rectal Exams

- You may feel discomfort or pressure when your rectum is examined.
- You may experience some discomfort when the swab is inserted into the rectum, and occasionally minor rectal bleeding may occur.

Risks from Flexible Sigmoidoscopy with Biopsies

- You may experience some mild discomfort and feel like you have a "bloated stomach"
- Even though the risk is low, you may experience infection, mild rectal irritation and urgency. It is important that you do not put anything in your rectum for 5 days after the biopsies, because you may be at higher risk for getting or spreading an infection until the biopsy site(s) have healed.
- You may experience limited rectal bleeding (1 to 2 days after the procedure) related to the biopsies
- You may experience low blood pressure
- Even though the risk is very rare, there is a very small chance that you may have a hole or a tear in the intestine. The risk of this complication is estimated to be about 1 in a 1,000 people who have flexible sigmoidoscopy with biopsies. If this happens, surgery to repair the tear may be necessary.

Risks from Rectal Swabs

- You may experience some mild discomfort and pressure in your rectum. In some cases, a very small amount of bleeding may occur

Risks from Vaginal Swabs

You may experience some mild discomfort and pressure in your genital and pelvic area

Risks from Enemas

- You may experience some mild discomfort and a bloated or crampy feeling

Risks from the Applicator

- You may experience some discomfort from the applicator since it has been designed for vaginal, not rectal use.

Risks from Tenofovir Gel

If you are in a group that gets tenofovir gel, the gel could cause some bad effects. We do not yet know all the bad effects of the gels. Since we do not know how the gel will affect pregnant women and their unborn babies, it is especially important that you do not become pregnant while on the study. The study staff will tell you about ways to avoid becoming pregnant. We do not know what effects tenofovir gel will have on the rectum, which is also why study staff will require participants to be sexually abstinent (rectal and vaginal) during the active phases of the study and for 5 days after the last biopsy is collected. Some, but not all, women who used the vaginal gels in other studies have had:

- Dryness, itching, burning feeling, or pain in the genital area.
- Vaginal candidiasis (a kind of vaginal infection).
- Discharge from the vagina.
- Irritation in the genital area.

Risks from Tenofovir Tablets

The tablets could cause some bad effects. We do not yet know all the effects of the tablets.

About 5 out of 100 people with HIV taking the tenofovir or a tablet containing tenofovir and another kind of medicine have these occasional side effects:

- Upset stomach, vomiting, gas, loose or watery stools
- Dizziness or headache
- Abdominal pain
- Lack of energy/general body weakness
- Mild problems of kidney function that are only detected by laboratory tests
- Shortness of breath or cough
- Rash, including allergic reaction
- Anxiety
- Joint pain, muscle pain, or other pain syndrome
- Fever
- Low phosphate, a chemical in the blood
- Bone thinning
- Kidney failure
- Inflammation or possible damage to the pancreas

Lactic acidosis (elevated lactic acid levels in the blood) and severe hepatomegaly (enlarged liver) with steatosis (fatty liver) that may result in liver failure, other complications or death have been reported with the use of antiretroviral nucleoside analogues (tenofovir tablet) alone or in combination. The liver complications and death have been seen more often in women on these drug regimens. Some nonspecific symptoms that might indicate lactic acidosis include: unexplained weight loss, stomach discomfort, nausea, vomiting, fatigue, cramps, muscle pain, weakness, dizziness and shortness of breath.

Risks from Phone Interview

The phone interviews will involve discussions on personal matters, such as sexual behavior. Talking about these issues may make you feel uncomfortable. Remember that the interviewers are professionals trained in sexual research who will keep all your information confidential, and that you can choose not to answer specific questions or stop the interview at any time. The phone interview will be recorded using a digital audio recorder. This is done because all the information you may give us is very important to us, and we want a complete record. Audio recording of the interviews is a study requirement. All audio recordings will be kept on a password-protected computer at the researchers' offices in New York, and only the study staff will be able to access them. The audio recordings will be transcribed (put in writing) by the person interviewing you or by another person. Neither the interviewer nor the transcriber will have any identifying information about you. Any names that might be mentioned on the recording will NOT be written down. The audio recordings will be destroyed as soon as they are transcribed and the transcripts have been checked for accuracy, but no later than two months from the time you are interviewed. You should understand, however, that even with all of these procedures in place, there is the potential risk for loss of confidentiality.

Other Possible Risks:

- You may become embarrassed, worried, or nervous when discussing personal questions about your sexual behavior, ways to protect against HIV and other infections passed during sex, and your test results.
- You may become worried or nervous while waiting for your test results.
- If you have HIV or other infections, knowing this could make you worried or nervous. A trained counselor will help you deal with any feelings or questions you have.

We will make every effort to protect your privacy while you are having the screening exams and tests. Your visits here will take place in private. However, it is possible that others may learn that you are taking part in the study here. Because of this, they may treat you unfairly or discriminate against you. For example, you could have problems getting or keeping a job, or being accepted by your family or community. Finding out your HIV status could also cause problems between you and your partner.

Some other studies of HIV prevention have found an unexpected higher risk of getting HIV among study participants. This could happen in any study, including this study. Because of this, the study staff will remind you not to have sex (rectal or vaginal) during the active phases of this study and for at least 5 days after the last biopsy collection. The study staff, will also remind you of the importance of using condoms to protect against HIV.

Very rarely, some of the bad effects listed in this form, such as liver problems, may cause death if they are very severe.

BENEFITS
You may get no direct benefit from being in this study. **We do not know if tenofovir gel or tenofovir tablets work to protect against HIV.** Also, the gel you are getting may be the placebo gel. Because of this, study staff will remind you of the importance not using any over-the-counter rectal medications at any time during the study or having sex (vaginal or rectal), and/or using any kind of sex toy during the resting periods of the study. The study staff will also remind you of the importance of using condoms to protect against HIV.

You or others may benefit in the future from information learned in this study. You also may get some personal satisfaction from being part of research on HIV prevention. This is true no matter what study group you are in.

You will have physical exams and rectal exams. You will have tests to check on the health of your blood, liver, and kidneys. If these tests show that you might have any health problems, you will be referred for medical care and other services available to you.

You will get counseling and testing for HIV. You will get free condoms. If you have infections passed through sex, other than HIV infection, you will be offered medicine to treat them or be referred for treatment, if needed. This study does not provide medication for treatment of HIV/AIDS. If you become infected with HIV, you will be referred for medical care, counseling, and other services available to you.

NEW INFORMATION
You will be told any new information learned during this or other relevant studies that might affect your health or willingness to stay in this study.

WHY YOU MAY HAVE TO STOP TAKING THE STUDY DRUG EARLY
You will have to stop using gel or tablets if you:
- Become infected with HIV.
- Become infected with hepatitis B.
- Become pregnant.
- Are breastfeeding.
- Are taking certain medications that affect your kidneys.
- Are unable or unwilling to follow study procedures or instructions.
- Could be harmed by continuing to take gel or tablets.

WHY YOU MAY BE WITHDRAWN FROM THE STUDY WITHOUT YOUR CONSENT
You may be withdrawn from the study without your consent for the following reasons:
- The study is stopped or canceled.
- The study staff feel that staying in the study would be harmful to you.
- You are not willing to find out your HIV test results.
- You are not willing to follow study procedures
- Other reasons, decided by the study staff.

If you withdraw early from the study, we will ask you to come in for a final visit with all the exams and tests listed above.

ALTERNATIVES TO PARTICIPATION

You do not have to be in this study. The decision to not be in this study will not affect your care in any way.

COSTS TO YOU

There is no cost to you for the study procedures and exams.

REIMBURSEMENT

You will be paid for your time and effort for all regularly scheduled study visits, including today's visit. You will receive [INSERT SITE - SPECIFIC AMOUNT OF MONEY] for each visit. You will also be paid for other costs to you for coming to the screening visit [SUCH AS CHILD CARE, TRAVEL, AND LOSS OF WORK TIME – SITES TO COMPLETE].

CONFIDENTIALITY

Efforts will be made to keep your personal information confidential. However, it is not possible to guarantee confidentiality. Your personal information may be disclosed if required by law. The study staff will use your personal information, if needed, to verify that you are not taking part in any other research studies. This includes other studies conducted by [site name] and studies conducted by other researchers that study staff know about. Any publication of this study will not use your name or identify you personally.

Your records may be reviewed by:

- the United States Food and Drug Administration (FDA)
- the United States National Institutes of Health (NIH)
- the Office for Human Research Protections (OHRP)
- [insert names of applicable IRBs]
- study staff
- study monitors
- the organization that provides the gels used in this study
- the company that makes the tablet used in this study

[Sites to include/amend the following if applicable:] [Local/state/national] regulations require study staff to report the names of people who test positive for [HIV and other infections] passed during sex to the [local health authority]. Outreach workers from the [health authority] may then contact you about informing your partners, since they also should be tested. If you do not want to inform your partners yourself, the outreach workers will contact them, according to the confidentiality guidelines of the [health authority].

In addition to the efforts made by the study staff to keep your personal information confidential, a Certificate of Confidentiality has been requested from the U.S. Federal

Government for this study. Once obtained this Certificate will protect study staff from being forced to tell people who are not connected with this study, such as the court system, about your participation or information you give for study purposes. Even with the Certificate of Confidentiality, however, if the study staff learns of possible child abuse and/or neglect or a risk of harm to you or others, they will be required to tell the proper authorities. Having a Certificate of Confidentiality does not prevent you from releasing information about yourself and your participation in the study.

RESEARCH-RELATED INJURY

[Sites to specify institutional policy:] It is unlikely that you will be injured as a result of study participation. If you are injured, the [institution] will give you immediate necessary treatment for your injuries. You [will/will not] have to pay for this treatment. You will be told where you can get additional treatment for your injuries. There is no program to pay money or give other forms of compensation for such injuries. You do not give up any legal rights by signing this consent form.

PROBLEMS OR QUESTIONS

If you ever have any questions about the study, or if you have a research-related injury, you should contact [insert name of the investigator or other study staff] at [insert telephone number and/or physical address].

If you have questions about your rights as a research participant, you should contact [insert name or title of person on the IRB or other organization appropriate for the site] at [insert physical address and telephone number].

If you have questions about whom to contact at the research site, you should contact [insert name of the investigator or community educator or CAB member [staff will decide which] at [insert physical address and telephone number].

[SITES THAT PREFER NOT TO INCLUDE THE FIGURE SHOWING THE PROTOCOL SCHEMA AND/OR THE TABLE OF STUDY VISITS AND EVALUATIONS ON THE FOLLOWING TWO PAGES MAY DELETE THESE ATTACHMENTS]

SIGNATURE PAGE
[INSERT SIGNATURE BLOCKS AS REQUIRED BY LOCAL IRB]

If you have read the informed consent (or had it read and explained to you), and all your questions have been answered, please sign your name or make your mark below.

Participant's Name (print)	Participant's Signature	Date
Study Staff Conducting Consent Discussion (print)	Study Staff Signature	Date
Witness' Name (print) (As appropriate)	Witness's Signature	Date

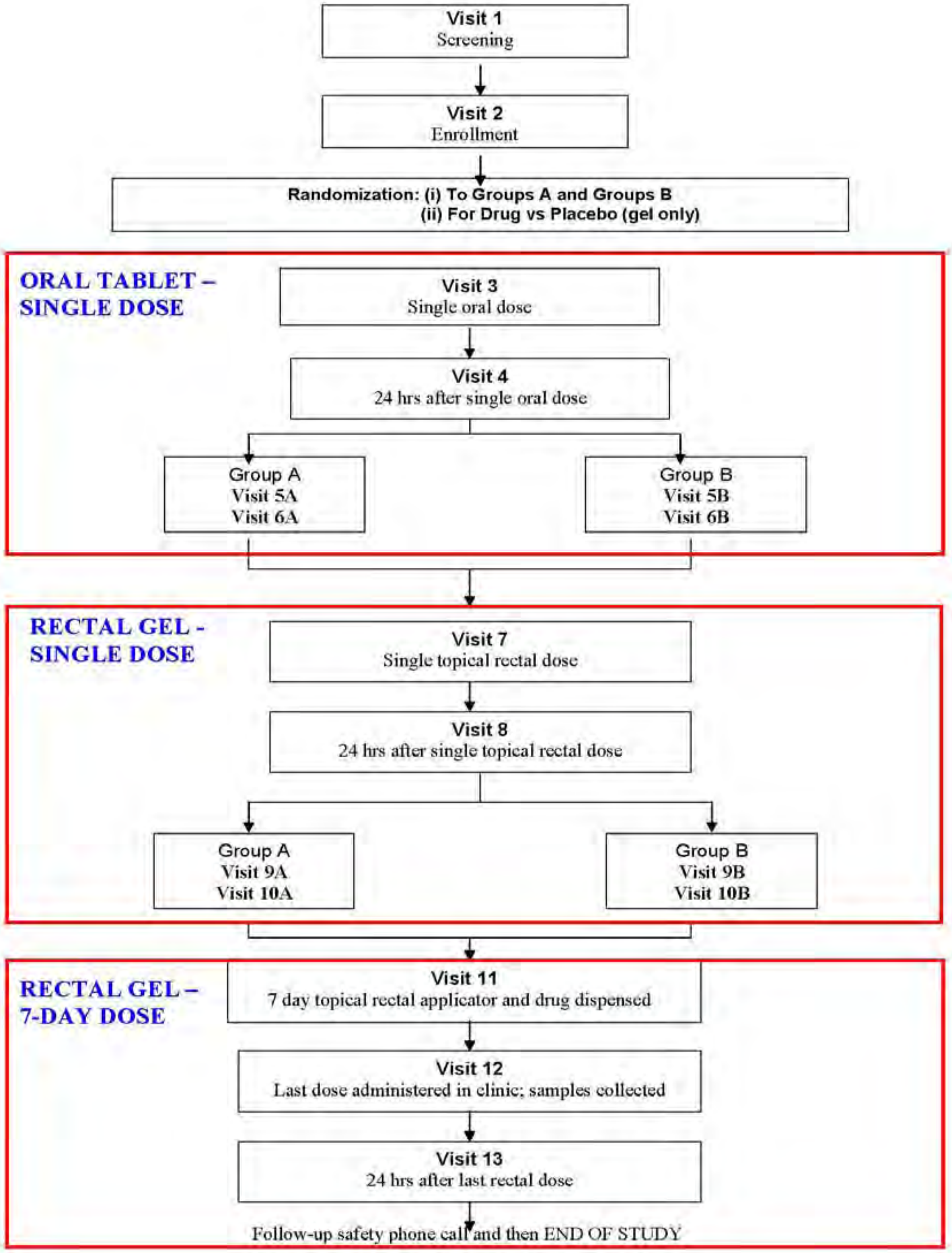
Visit 1
Screening
Visit 2
Enrollment
Randomization: (i) To Groups A and Groups B
(ii) For Drug vs Placebo (gel only)
ORAL TABLET – SINGLE DOSE
Visit 3
Single oral dose
Visit 4
24 hrs after single oral dose
Group A
Visit 5A
Visit 6A
Group B
Visit 5B
Visit 6B
RECTAL GEL - SINGLE DOSE
Visit 7
Single topical rectal dose
Visit 8
24 hrs after single topical rectal dose
Group A
Visit 9A
Visit 10A
Group B
Visit 9B
Visit 10B
RECTAL GEL – 7-DAY DOSE
Visit 11
7 day topical rectal applicator and drug dispensed
Visit 12
Last dose administered in clinic; samples collected
Visit 13
24 hrs after last rectal dose
Follow-up safety phone call and then END OF STUDY

Schedule of Study Visits and Evaluations

	Visit 1	Visit 2	Visit 3	Visit 4	Visit 5	Visit 6	Visit 7	Visit 8	Visit 9	Visit 10	Visit 11	Visit 12	Visit 13/ Early Term	F/U Call	Interim Visit
			Single dose oral phase				Single dose rectal phase				7-day rectal phase				
Informed consent	X	X													
Questions about yourself such as your education	X	X													
Information about where you live and how we can contact you	X	X	X	X	X	X	X	X	X	X	X	X			X
Test results review		X	X				X				X	X			
Questions to see if you can join the study	X	X													
Questions about your sexual practices (on the computer)		X													
Questions about your smoking habits	X														
Review the rules of the study and/or study product rules		X	X	X	X	X	X	X	X	X	X	X			
Schedule next study visit	▲	X	X	X	X	X	X	X	X	X	X	X		▲	▲
Find out which study group your are in		X													
Information about your health and the medicines you are taking	X	X	X	X	X	X	X	X	X	X	X	X	X		X
Physical exam	X	X	X	X	X	X	X	X	X	X	X	X	X		▲
Rectal exam	X														▲
HIV/STI risk reduction, HIV pre- and post test counseling	X	X	X	X	X	X	X	X	X	X	X	X	X		▲
Learn about ways to avoid becoming pregnant	X	X	X	X	X	X	X	X	X	X	X	X	X		X
Receive supply of male condoms	X	X	X	X	X	X	X	X	X	X	X	X	X		
Flexible Sigmoidoscopy		X	X		X	X	X		X	X		X			
Answer questions about your health and how you are feeling and/or any study product side effects		X	X	X	X	X	X	X	X	X	X	X	X	X	▲
Receive study tablet or gel			X				X				X	X			
Return study diary and used and unused applicators to study clinic and review daily verification call log												X		X	
Answer questions about your experience using the study product (on the computer and in a phone interview)													X		
Safety phone call														X	
Lab tests:															
Urine test	X														▲
Pregnancy test	X	X									X				▲
HIV test	X	▲	▲	▲	▲	▲	▲	▲	▲	▲	▲	X	▲		▲
Syphilis test	X														▲
Blood	X	X	X	X	X	X	X	X	X	X	X	X	X		▲
Vaginal swabs/ sponges	X	X	X	X	X	X	X	X	X	X	X	X	X		▲
Rectal swabs/sponges	X	X	X	X	X	X	X	X	X	X	X	X	X		

APPENDIX IX: SAMPLE INFORMED CONSENT (STORAGE AND FUTURE TESTING OF SPECIMENS)

A two-site, Phase 1, partially-blinded, placebo-controlled safety, acceptability, and pharmacokinetic trial of topical, vaginally-formulated tenofovir 1% gel applied rectally compared with oral 300 mg tenofovir disoproxil fumarate in HIV-1 seronegative adults

Version 1.0

7 April 2009

**PRINCIPAL INVESTIGATOR:**	**[INSERT NAME]**
**PHONE:**	**[INSERT NUMBER]**
**Short Title for the Study:**	**Phase 1 rectal microbicide safety and acceptability trial of topically applied tenofovir compared with oral tablet**

INTRODUCTION

You have decided to take part in a Division of AIDS research study. While you are in this research study there may be some samples of tissue, and/or fluid from your rectum taken from you that might be useful for future research. You are being asked to agree to the storage of these samples. This consent form gives you information about the collection, storage and use of your samples. The study staff will talk with you about this information. Please ask any questions, if you have some. If you agree to the storage of your samples, you will be asked to sign or make your mark on this consent form. You will be given a copy of this form copy to keep.

HOW WILL YOU GET THE SAMPLES FROM ME?

The research doctors want to save any extra tissue and/or rectal fluid leftover from your tests during the study. The leftover tissue samples and rectal fluid will be kept and used for future research.

HOW WILL YOU USE MY SAMPLES?

Your samples will be used to look for ways that your body responds to infection (such as cells, proteins, and other chemicals in your body). Tests may also include checking your genes (material passed from parent to child that determines the make-up of the body and mind), since they might affect how your body responds to disease. Your genes might make you more or less likely to get an infection, affect your responses to infection, or make your responses to treatment stronger or weaker. The researchers do not plan to contact you or your regular doctor with any results from tests done on your stored samples. This is because research tests are often done with experimental procedures, so the results from one research study are generally not useful for your medical care. If a rare situation came up where the researchers decided that a test result would provide important information for your health, the researchers would tell your study doctor and your study doctor would try to contact you. If you wish to be

contacted with this type of test result, you must give the study doctor or nurse any change to your address and/or phone number. If you want your regular doctor to be told about this type of test result, you must provide the study doctor or nurse with your regular doctor's name, address and phone number. Your samples will not be sold or used directly to produce products that can be sold for profit.

Research studies using your samples will be reviewed by the National Institutes of Health and a special committee at the researcher's institution (an Institutional Review Board) whose purpose is to protect you as a research participant.

HOW LONG WILL YOU KEEP MY SAMPLES?
There is no time limit on how long your samples will be stored.

HOW WILL MY SAMPLES BE STORED?
Some of your samples will be stored at special facilities at the UCLA MICL Laboratory and at the MTN Pharmacology Laboratory that are designed to store samples securely. The storage facilities are made so that only approved researchers will have access to the samples. An Institutional Review Board will oversee the storage facilities to protect you and other research volunteers from harm.

DOES STORAGE OF MY SAMPLES BENEFIT ME?
There are no direct benefits to you.

WHAT ARE THE RISKS?
There are few risks related to storing your samples. When tests are done on the stored samples there is a small but possible risk to your privacy. It is possible that if others found out information about you from tests (such as information about your genes) it could cause you problems with your family (having a family member learn about a disease that may be passed on in families or learning who is the biological parent of a child) or problems getting a job or insurance.

WHAT ABOUT CONFIDENTIALITY?
To keep your information private, your samples will be labeled with a code that can only be traced back to your research clinic. Your personal information (name, address, phone number) will be protected by the research clinic. When researchers are given your stored samples to study they will not be given your personal information. The results of future tests will not be included in your health records.

Your records may be reviewed by:
- the United States Food and Drug Administration (FDA)
- the United States National Institutes of Health (NIH)
- the Office for Human Research Protections (OHRP)
- [insert names of applicable IRBs]
- study staff
- study monitors
- the organization that provides the gels used in this study

- the company that makes the tablet used in this study

In addition to the efforts made by the study staff to keep your personal information confidential, a Certificate of Confidentiality has been requested from the U.S. Federal Government for this study. Once obtained this Certificate will protect study staff from being forced to tell people who are not connected with this study, such as the court system, about your participation or information you give for study purposes. Even with the Certificate of Confidentiality, however, if the study staff learns of possible child abuse and/or neglect or a risk of harm to you or others, they will be required to tell the proper authorities. Having a Certificate of Confidentiality does not prevent you from releasing information about yourself and your participation in the study.

WHAT ARE MY RIGHTS?

Allowing your samples to be stored is completely voluntary. You may decide not to have any samples stored other than what is needed to complete this study and still be in this research study or any future study. If you decide now that your samples can be stored for future research, you may change your mind at any time. You must contact your study doctor or nurse and let them know that you do not want your samples used for future research. Your samples will then not be used and will be destroyed.

WHAT DO I DO IF I HAVE QUESTIONS?

For questions about the storage of your samples, contact (*insert the name of the investigator*) at *(insert telephone number)*.

For questions about your rights related to the storage of your samples for research, contact (*insert the name or title of person on the Institutional Review Board) at (insert telephone number)*.

SIGNATURE PAGE
[INSERT SIGNATURE BLOCKS AS REQUIRED BY LOCAL IRB]

If you have read the informed consent (or had it read and explained to you), and all your questions have been answered and you agree to this specimen storage and future testing, please sign your name or make your mark below.

____________________ Participant's Name (print)	____________________ Participant's Signature	Date
____________________ Study Staff Conducting Consent Discussion (print)	____________________ Study Staff Signature	Date
____________________ Witness' Name (print) (As appropriate)	____________________ Witness's Signature	Date

APPENDIX X: SAMPLE PRODUCT USE LOG DAILY DIARY

A two-site, Phase 1, partially-blinded, placebo-controlled safety, acceptability, and pharmacokinetic trial of topical, vaginally-formulated tenofovir 1% gel applied rectally compared with oral 300 mg tenofovir disoproxil fumarate in HIV-1 seronegative adults

Version 1.0

7 April 2009

PRINCIPAL INVESTIGATOR: **[INSERT NAME]**
PHONE: **[INSERT NUMBER]**
Short Title for the Study: **Phase 1 rectal microbicide safety and acceptability trial of topically applied tenofovir compared with oral tablet**

Participant ID #___________
Date Dispensed____________

- **Please store applicators at room temperature away from direct sunlight.**
- **You are encouraged to use <u>each Gel applicator daily in the a.m.</u> at approximately the same time everyday.**
- **Please <u>return all applicators used and unused</u> in the storage bags provided with the daily diary.**
- **If you require additional space for comments use back side of this log**

Day	Date	Time	Comments
1			
2			
3			
4			
5			
6			

DAY 7 GIVEN IN CLINIC

	Date	Time	Comment
7			

Date Returned________________ Staff Signature______________________________

Please return all applicators used & unused (in the plastic bags provided) on day 7

REFERENCES

1. Koblin BA, Chesney MA, Husnik MJ, et al. High-risk behaviors among men who have sex with men in 6 US cities: baseline data from the EXPLORE Study. Am J Public Health 2003;93(6):926-32.
2. Gross M, Holte SE, Marmor M, Mwatha A, Koblin BA, Mayer KH. Anal sex among HIV-seronegative women at high risk of HIV exposure. The HIVNET Vaccine Preparedness Study 2 Protocol Team. J Acquir Immune Defic Syndr 2000;24(4):393-8.
3. Civic D. College students' reasons for nonuse of condoms within dating relationships. J Sex Marital Ther 2000;26(1):95-105.
4. Mosher WD, Chandra A, Jones J. Sexual behavior and selected health measures: men and women 15-44 years of age, United States, 2002. Advance Data 2005(362):1-55.
5. Erickson PI, Bastani R, Maxwell AE, Marcus AC, Capell FJ, Yan KX. Prevalence of anal sex among heterosexuals in California and its relationship to other AIDS risk behaviors. AIDS Educ Prev 1995;7(6):477-93.
6. Poles MA, Elliott J, Taing P, Anton PA, Chen IS. A preponderance of CCR5(+) CXCR4(+) mononuclear cells enhances gastrointestinal mucosal susceptibility to human immunodeficiency virus type 1 infection. J Virol 2001;75(18):8390-9.
7. Leynaert B, Downs AM, de Vincenzi I. Heterosexual transmission of human immunodeficiency virus: variability of infectivity throughout the course of infection. European Study Group on Heterosexual Transmission of HIV. American Journal of Epidemiology 1998;148(1):88-96.
8. Vittinghoff E, Douglas J, Judson F, McKirnan D, MacQueen K, Buchbinder SP. Per-contact risk of human immunodeficiency virus transmission between male sexual partners. American Journal of Epidemiology 1999;150(3):306-11.
9. Gray RH, Wawer MJ, Brookmeyer R, et al. Probability of HIV-1 transmission per coital act in monogamous, heterosexual, HIV-1-discordant couples in Rakai, Uganda. Lancet 2001;357(9263):1149-53.
10. Padian NS, Shiboski SC, Glass SO, Vittinghoff E. Heterosexual transmission of human immunodeficiency virus (HIV) in northern California: results from a ten-year study. American Journal of Epidemiology 1997;146(4):350-7.
11. Phillips DM, Sudol KM, Taylor CL, Guichard L, Elsen R, Maguire RA. Lubricants containing N-9 may enhance rectal transmission of HIV and other STIs. Contraception 2004;70(2):107-10.
12. Phillips DM, Taylor CL, Zacharopoulos VR, Maguire RA. Nonoxynol-9 causes rapid exfoliation of sheets of rectal epithelium. Contraception 2000;62(3):149-54.
13. Anton PA. A phase I safety and acceptability study of the UC-781 microbicide gel applied rectally in HIV seronegative adults: an interim safety report at 50% completion. In: Microbicides 2008; 2008; New Delhi, India; 2008.
14. Patton DL, Cosgrove Sweeney YT, McCarthy TD, Hillier SL. Preclinical safety and efficacy assessments of dendrimer-based (SPL7013) microbicide gel formulations in a nonhuman primate model. Antimicrobial Agents & Chemotherapy 2006;50(5):1696-700.

15. Patton DL, Sweeney YC, Cummings PK, Meyn L, Rabe LK, Hillier SL. Safety and efficacy evaluations for vaginal and rectal use of BufferGel in the macaque model. Sex Transm Dis 2004;31(5):290-6.
16. Patton DL, Sweeney YT, Balkus JE, Hillier SL. Vaginal and rectal topical microbicide development: safety and efficacy of 1.0% Savvy (C31G) in the pigtailed macaque. Sexually Transmitted Diseases 2006;33(11):691-5.
17. Geboes K, Riddell R, Ost A, Jensfelt B, Persson T, Lofberg R. A reproducible grading scale for histological assessment of inflammation in ulcerative colitis.[see comment]. Gut 2000;47(3):404-9.
18. McGowan I, Elliott J, Cortina G, et al. Characterization of baseline intestinal mucosal indices of injury and inflammation in men for use in rectal microbicide trials (HIV Prevention Trials Network-056). J Acquir Immune Defic Syndr 2007;46(4):417-25.
19. Tabet SR, Surawicz C, Horton S, et al. Safety and toxicity of nonoxynol-9 gel as a rectal microbicide. Sex Transm Dis 1999;26(10):564-71.
20. Shacklett BL, Yang O, Hausner MA, et al. Optimization of methods to assess human mucosal T-cell responses to HIV infection. Journal of Immunological Methods 2003;279(1-2):17-31.
21. Anton PA, Elliott J, Poles MA, et al. Enhanced levels of functional HIV-1 co-receptors on human mucosal T cells demonstrated using intestinal biopsy tissue. AIDS 2000;14(12):1761-5.
22. Lapenta C, Boirivant M, Marini M, et al. Human intestinal lamina propria lymphocytes are naturally permissive to HIV-1 infection. European Journal of Immunology 1999;29(4):1202-8.
23. Patterson BK, Czerniewski M, Andersson J, et al. Regulation of CCR5 and CXCR4 expression by type 1 and type 2 cytokines: CCR5 expression is downregulated by IL-10 in CD4-positive lymphocytes. Clinical Immunology 1999;91(3):254-62.
24. Weissman D, Dybul M, Daucher MB, Davey RT, Jr., Walker RE, Kovacs JA. Interleukin-2 up-regulates expression of the human immunodeficiency virus fusion coreceptor CCR5 by CD4+ lymphocytes in vivo. Journal of Infectious Diseases 2000;181(3):933-8.
25. Bjerke K, Halstensen TS, Jahnsen F, Pulford K, Brandtzaeg P. Distribution of macrophages and granulocytes expressing L1 protein (calprotectin) in human Peyer's patches compared with normal ileal lamina propria and mesenteric lymph nodes. Gut 1993;34(10):1357-63.
26. Johne B, Fagerhol MK, Lyberg T, et al. Functional and clinical aspects of the myelomonocyte protein calprotectin. Molecular Pathology 1997;50(3):113-23.
27. Brun JG, Ulvestad E, Fagerhol MK, Jonsson R. Effects of human calprotectin (L1) on in vitro immunoglobulin synthesis. Scandinavian Journal of Immunology 1994;40(6):675-80.
28. Yui S, Mikami M, Yamazaki M. Induction of apoptotic cell death in mouse lymphoma and human leukemia cell lines by a calcium-binding protein complex, calprotectin, derived from inflammatory peritoneal exudate cells. Journal of Leukocyte Biology 1995;58(6):650-8.
29. Roseth AG, Aadland E, Jahnsen J, Raknerud N. Assessment of disease activity in ulcerative colitis by faecal calprotectin, a novel granulocyte marker protein. Digestion 1997;58(2):176-80.

30. Tibble J, Teahon K, Thjodleifsson B, et al. A simple method for assessing intestinal inflammation in Crohn's disease. Gut 2000;47(4):506-13.
31. Thjodleifsson B, Sigthorsson G, Cariglia N, et al. Subclinical intestinal inflammation: an inherited abnormality in Crohn's disease relatives?[see comment]. Gastroenterology 2003;124(7):1728-37.
32. Carballo-Dieguez A, Dolezal C, Bauermeister J, Ventuneac A, O'Brien W, Mayer K. Preference for gel over suppository as delivery vehicle for a rectal microbicide: results of a randomised, crossover acceptability trial among men who have sex with men. Sexually Transmitted Infections 2008;84:483-7.
33. Carballo-Dieguez A, Exner T, Dolezal C, Pickard R, Lin P, Mayer KH. Rectal microbicide acceptability: results of a volume escalation trial. Sexually Transmitted Diseases 2007;34(4):224-9.
34. CONRAD. Investigator's Brochure: Universal Placebo Gel. 2005;Version 1.0.
35. Dezzutti CS. Personal Communication. In; 2008.
36. Abner SR, Guenthner PC, Guarner J, et al. A human colorectal explant culture to evaluate topical microbicides for the prevention of HIV infection. Journal of Infectious Diseases 2005;192(9):1545-56.
37. Gilead, Sciences. Viread Investigator's Brochure. September 2006.
38. Gilead, Sciences. Viread® (tenofovir disoproxil fumarate) tablets. Package Insert November 2008.
39. Weber J, Chakraborty B, Weberova J, Miller MD, Quinones-Mateu ME. Diminished replicative fitness of primary human immunodeficiency virus type 1 isolates harboring the K65R mutation. Journal of Clinical Microbiology 2005;43(3):1395-400.
40. Tien D, Schnaare RL, Kang F, et al. In vitro and in vivo characterization of a potential universal placebo designed for use in vaginal microbicide clinical trials. AIDS Research & Human Retroviruses 2005;21(10):845-53.
41. Dezzutti CS, James VN, Ramos A, et al. In vitro comparison of topical microbicides for prevention of human immunodeficiency virus type 1 transmission. Antimicrobial Agents & Chemotherapy 2004;48(10):3834-44.
42. Tarantal AF, Castillo A, Ekert JE, Bischofberger N, Martin RB. Fetal and maternal outcome after administration of tenofovir to gravid rhesus monkeys (Macaca mulatta). Journal of Acquired Immune Deficiency Syndromes: JAIDS 2002;29(3):207-20.
43. Van Rompay KK, Marthas ML, Lifson JD, et al. Administration of 9-[2-(phosphonomethoxy)propyl]adenine (PMPA) for prevention of perinatal simian immunodeficiency virus infection in rhesus macaques. AIDS Research & Human Retroviruses 1998;14(9):761-73.
44. Subbarao S, Otten RA, Ramos A, et al. Chemoprophylaxis with tenofovir disoproxil fumarate provided partial protection against infection with simian human immunodeficiency virus in macaques given multiple virus challenges.[see comment]. Journal of Infectious Diseases 2006;194(7):904-11.
45. Creek MR. Pharmacokinetics and tissue distribution study of PMPA (GS-1278) following intravaginal administration in female New Zealand White Rabbits. SRI Report No B010-97.
46. CONRAD. Investigator's Brochure: Tenofovir Gel (GS-1278). Third Edition.

47. Lund B, Weber S. Pharmacokinetics and distribution of intravaginal [14C] PMPA (GS-1278) following single dose administration to rats. Gilead Sciences Report No 96-DDM-1278-004.
48. Cranage M, Sharpe S, Herrera C, et al. Prevention of SIV Rectal Transmission and Priming of T Cell Responses in Macaques after Local Pre-exposure Application of Tenofovir Gel. PLoS Medicine 2008;5(8):1-13.
49. Rezk NL, Crutchley RD, Kashuba AD. Simultaneous quantification of emtricitabine and tenofovir in human plasma using high-performance liquid chromatography after solid phase extraction. Journal of Chromatography B: Analytical Technologies in the Biomedical & Life Sciences 2005;822(1-2):201-8.
50. Balzarini J, Van Herrewege Y, Vanham G. Metabolic activation of nucleoside and nucleotide reverse transcriptase inhibitors in dendritic and Langerhans cells. AIDS 2002;16(16):2159-63.
51. Delaney WEt, Ray AS, Yang H, et al. Intracellular metabolism and in vitro activity of tenofovir against hepatitis B virus. Antimicrobial Agents & Chemotherapy 2006;50(7):2471-7.
52. Robbins BL, Srinivas RV, Kim C, Bischofberger N, Fridland A. Anti-human immunodeficiency virus activity and cellular metabolism of a potential prodrug of the acyclic nucleoside phosphonate 9-R-(2-phosphonomethoxypropyl)adenine (PMPA), Bis(isopropyloxymethylcarbonyl)PMPA. Antimicrobial Agents & Chemotherapy 1998;42(3):612-7.
53. Van Rompay KK, Kearney BP, Sexton JJ, et al. Evaluation of oral tenofovir disoproxil fumarate and topical tenofovir GS-7340 to protect infant macaques against repeated oral challenges with virulent simian immunodeficiency virus. J Acquir Immune Defic Syndr 2006;43(1):6-14.
54. Fairchild D. 14-day vaginal tissue irritation and toxicity study of PMPA in rats. Report No M016-97.
55. Rush RE. A vaginal irritation study in rabbits with PMPA (GS-1278) topical gel. . Gilead Sciences Report No 96-TOX-1278-003.
56. Patty's Industrial Hygiene and Toxicology Online. http://www3intersciencewileycom/cgi-bin/mrwhome/104554795/HOME; accessed 4/25/08:Section 43.4.
57. Guttner J, Klaus S, Heinecke H. Embryotoxizitat intraperitoneal applizierter Hydroxyethylcellulose bei der Maus. Anatomischer Anzeiger 1981;149(3):282-5.
58. http://depts.washington.edu/terisweb/teris/index.html. In; accessed January 8, 2008.
59. Patton DL. A Combination Microbicide Shows Differential Toxicity Compared With Standalone Products. In: Microbicides 2008; New Delhi.
60. Patton DL, Cosgrove Sweeney YT, Paul KJ. A summary of preclinical topical microbicide rectal safety and efficacy evaluations in a pigtailed macaque model. In Press: Sexually Transmitted Diseases 2008.
61. Cassetti I, Madruga JV, Suleiman JM, et al. The safety and efficacy of tenofovir DF in combination with lamivudine and efavirenz through 6 years in antiretroviral-naive HIV-1-infected patients. HIV Clinical Trials 2007;8(3):164-72.
62. Gitman MD, Hirschwerk D, Baskin CH, Singhal PC. Tenofovir-induced kidney injury. Expert Opinion on Drug Safety 2007;6(2):155-64.

63. Fux CA, Christen A, Zgraggen S, Mohaupt MG, Furrer H. Effect of tenofovir on renal glomerular and tubular function. AIDS 2007;21(11):1483-5.
64. Nelson MR, Katlama C, Montaner JS, et al. The safety of tenofovir disoproxil fumarate for the treatment of HIV infection in adults: the first 4 years. AIDS 2007;21(10):1273-81.
65. Vigano A, Zuccotti GV, Martelli L, et al. Renal safety of tenofovir in HIV-infected children: a prospective, 96-week longitudinal study. Clinical Drug Investigation 2007;27(8):573-81.
66. Peterson L, Taylor D, Roddy R, et al. Tenofovir disoproxil fumarate for prevention of HIV infection in women: a phase 2 double-blind randomized placebo-controlled trial. . PLoS Clinical Trials;2(5:e27.doi:10.1371/journal.pctr.0020027).
67. www.apregistry.com/forms/interim_report.pdf. accessed April 30, 2008.
68. www.cdc.gov/ncbddd/bd/macdp.htm. accessed April 30, 2008.
69. Mayer KH, Maslankowski LA, Gai F, et al. Safety and tolerability of tenofovir vaginal gel in abstinent and sexually active HIV-infected and uninfected women. AIDS 2006;20(4):543-51.
70. CONRAD. Protocol #A04-099 (Study Report), Male Tolerance Study.
71. Hillier SL. Safety and acceptability of daily and coitally dependent use of 1% tenofovir over six months of use. In: Microbicides 2008; New Delhi.
72. Dreisbach R. Handbook of Poisoning. 9 ed. Los Altos, CA: Lang Medical Publications; 1977.
73. Schwartz JL, Ballagh SA, Kwok C, et al. Fourteen-day safety and acceptability study of the universal placebo gel. Contraception 2007;75(2):136-41.
74. Fuchs EJ, Lee LA, Torbenson MS, et al. Hyperosmolar sexual lubricant causes epithelial damage in the distal colon: potential implication for HIV transmission. Journal of Infectious Diseases 2007;195(5):703-10.
75. Misegades L, Page-Shafer K, Halperin D, McFarland W, Survey YWSSIGYWs. Anal intercourse among young low-income women in California: an overlooked risk factor for HIV? AIDS 2001;15(4):534-5.
76. Karim SS, Ramjee G. Anal sex and HIV transmission in women. American Journal of Public Health 1998;88(8):1265-6.
77. Phillips DM, Zacharopoulos VR. Nonoxynol-9 enhances rectal infection by herpes simplex virus in mice. Contraception 1998;57(5):341-8.
78. Patton DL, Cosgrove Sweeney YT, Rabe LK, Hillier SL. Rectal applications of nonoxynol-9 cause tissue disruption in a monkey model. Sex Transm Dis 2002;29(10):581-7.
79. Elliott J. HIV-1 challenge of colorectal explants may be an important predictor of microbicidal effectiveness in vivo. Early blinded results from a phase I rectal microbicide trial of UC-781. In: Microbicides 2008; 2008; New Delhi, India; 2008.
80. NIAID Study 97-101:Pharmacokinetic Study in Three Monkeys.
81. Schwartz J, Kashuba A, Rezk N, et al. Preliminary Results From a Pharmacokinetic Study of the Candidate Vaginal Microbicide Agent 1% Tenofovir Gel. Abstr #610. In: Microbicides 2008; New Delhi, India.
82. Carballo-Dieguez A, Dolezal C, Ventuneac A. Sexual Practices Assessment Schedule for Men who have Sex with Men (SPAS MSM 2002). Available from first author.

83. ICH Guidance (E2A), Clinical Safety Data Management: Definitions and Standards for Expediting Reporting. 1994.
84. Gatto NM, Frucht H, Sundararajan V, Jacobson JS, Grann VR, Neugut AI. Risk of perforation after colonoscopy and sigmoidoscopy: a population-based study.[see comment]. Journal of the National Cancer Institute 2003;95(3):230-6.
85. Richardson-Harman N, Anton P, Bremer J, et al. A standardized pre-clinical evaluation of HIV-1 growth and microbicide efficacy in tissue explants. TA-395. In: Microbicides 2008; New Delhi, India.
86. Little R, Rubin D. Statistical Analysis with Missing Data. 2nd ed. New York: John Wiley & Sons; 2002.
87. Schafer JL. Analysis of Incomplete Multivariate Data: Chapman and Hall/CRC Press; 1997.
88. Raghunathan TE, Lepkowski JM, Van Hoewyk J, Wolenberger P. A multivariate technique for multiplying missing values a sequence of regression models. Survey Methodology 2001;27:85-95.
